# Sample Size Calculations for Partially Clustered Trials

**DOI:** 10.1002/sim.70172

**Published:** 2025-07-15

**Authors:** Kylie M. Lange, Jessica Kasza, Thomas R. Sullivan, Lisa N. Yelland

**Affiliations:** ^1^ School of Public Health The University of Adelaide Adelaide South Australia Australia; ^2^ Women and Kids Theme South Australian Health and Medical Research Institute Adelaide South Australia Australia; ^3^ School of Public Health and Preventive Medicine Monash University Melbourne Victoria Australia

**Keywords:** clustered data, generalized estimating equations, power, re‐randomization design, sample size, trial design

## Abstract

Partially clustered trials are defined as trials where some observations belong to a cluster and others are independent. For example, neonatal trials may include infants from a single, twin, or triplet birth. The clustering of observations in partially clustered trials should be accounted for when determining the target sample size to avoid being over or underpowered. However, sample size methods have only been developed for limited partially clustered trial designs (e.g., designs with maximum cluster sizes of 2). In this article, we present new design effects that can be used to determine the sample size for two‐arm, parallel, partially clustered trials where clusters exist pre‐randomization. Design effects are derived algebraically for continuous and binary outcomes, assuming a generalized estimating equations‐based approach to estimation with either an independence or exchangeable working correlation structure. Both cluster and individual randomization are considered for the clustered observations. The design effects are shown to depend on the intracluster correlation coefficient, proportion of observations that belong to clusters of each size, method of randomization, type of outcome, and working correlation structure. The design effects are validated through a simulation study. Example sample size calculations are presented to illustrate how the design effects can be used to determine the target sample size for different partially clustered trial designs. The design effects depend on parameters that can be feasibly estimated when planning a trial and can be used to ensure that partially clustered trials are appropriately powered in the future.

## 1 Introduction

Partially clustered trials are trials that, by design, include a mixture of independent and clustered observations [1]. Partially clustered trials may involve clusters that exist pre‐randomization (e.g., body parts from the same person) or clusters formed post‐randomization (e.g., clusters induced by assigning otherwise independent participants to group education classes). They are common in fields including neonatology, where participants may include infants from single or multiple births, and orthopedics, where individuals may have one or more joints affected by disease. Partial clustering also arises in trials utilizing the re‐randomization design, where participants may complete the trial one or more times [2]. The presence of clustering in these trial designs can have important implications for sample size requirements, such that failing to account for clustering may lead to a trial being over‐ or under‐powered [3]. A common approach to account for clustering is to calculate the required sample size assuming all observations will be independent and multiplying this by an appropriate design effect (DEFF), however, this generally requires a formula for the DEFF.

Existing methods for calculating the required sample size for partially clustered trials based on DEFFs are somewhat limited. Partially clustered trials are commonly analyzed using mixed effects models fit via (restricted) maximum likelihood or marginal models fit via generalized estimating equations (GEEs) [4, 5, 6]. Sample size formulas based on these analysis methods have been developed for partially clustered trials with post‐randomization clustering. These are typically based on an asymmetrical mixed effects model by including a random cluster effect in the intervention group only [4, 7, 8, 9, 10]. More general treatments of such designs have allowed for clustering in both arms, but have only considered treatment effects at the cluster level such that cluster effects are nested within treatment group [11, 12]. Sample size methods for designs with pre‐existing clusters, where clustering occurs in both treatment arms and treatment assignment may be at the cluster or individual level (i.e., there may be cross‐classification of treatment and cluster), have received less attention and are the focus of this article.

We previously developed DEFF formulas for continuous and binary outcomes in trials involving independent and paired data (i.e., pre‐existing clusters of size two) based on analysis via GEEs with either an independence or exchangeable working correlation structure, and clustered observations randomized at the individual or cluster level (including balanced allocation to opposite groups) [13]. These DEFFs have also been shown to provide adequate power when analysis is via mixed effects models [14]. Clusters of size two are common in partially clustered trials, including in studies of paired body parts (e.g., eyes, knees), surgical procedures that are conducted either unilaterally or bilaterally, and infants from singleton or twin births. These DEFFs, allowing for clusters of size 2, are implemented in an online sample size calculator that can be used to plan new partially clustered trials with a maximum cluster size of two [15].

To our knowledge, no sample size methods have been developed for partially clustered trials with pre‐existing clusters larger than size two. Examples of partially clustered trials with larger cluster sizes include trials in orthopedics (e.g., diseased joints), dermatology (e.g., skin lesions), and neonatology (e.g., triplets). Re‐randomization trials also frequently include more than two randomizations for some participants and have been designed to allow up to seven [16, 17]. Previous research into methods for the design and analysis of partially clustered trials with a maximum cluster size of two has shown that the proportion of paired observations and the intracluster correlation coefficient (ICC) for outcomes of the paired observations are important for determining the power and required sample size [13, 18, 19]. However, it is not known how this extends to settings where there are larger clusters or a variety of cluster sizes.

The purpose of this article is to extend our previous sample size methods based on GEEs to partially clustered trial settings involving pre‐existing clusters with more than two observations. We focus on GEEs as they are commonly used for the analysis of partially clustered trials [5, 6, 20, 21]. The specific aims are to: (1) derive DEFFs for partially clustered trials with pre‐existing clusters of varying sizes; (2) validate the DEFFs via a simulation study; and (3) illustrate their use by determining the target sample sizes for hypothetical clinical trials. We restrict our attention to two‐arm, parallel, designs that use cluster or individual randomization for the clustered observations. While clusters of size two can also be randomized using balanced randomization to opposite treatment groups, the approach is more complex to implement and rarely used for larger clusters. We consider DEFFs for continuous and binary outcomes based on GEEs with an independence or exchangeable working correlation structure, in settings where cluster size is uninformative (i.e., neither the outcome nor the treatment effect depends on cluster size) [22].

The remainder of the paper is structured as follows. In Section 2, we algebraically derive the DEFFs and confirm their validity in a simulation study presented in Section 3. Applications of our sample size methods to hypothetical partially clustered trials are given in Section 4. Finally, a discussion is provided in Section 5.

## 2 Design Effects

### 2.1 Motivating Example

To motivate the use of DEFFs for calculating sample size, we consider a proposed trial of a new infant formula for infants born preterm. Multiple births are common among preterm births, so the researchers wish to include twins and triplets in the trial to enhance generalizability. To ensure the trial is appropriately powered, the presence of clusters of infants from multiple births should be accounted for in the sample size calculations. This section develops methods that can be used for designing such a trial, and sample size calculations are demonstrated for this example in Section 4.

### 2.2 Notation and Setting

Following the notation by Yelland et al. [13], let $Y_{\mathrm{ij}}$ be a continuous or binary outcome measured on the jth member of the ith cluster (i.e., the jth infant of the ith mother) ($i=1,\ldots M;j=1,\ldots n_{i};\sum_{i=1}^{M}n_{i}=N$). The effect of treatment group $X_{\mathrm{ij}}$ (1 = intervention, 0 = control) on the outcome can be estimated using GEEs based on the unadjusted mean model,

(1) $$g\left(\mu_{\mathrm{ij}}\right)=\beta_{0}+\beta_{1}X_{\mathrm{ij}}={X_{\mathrm{ij}}}^{T}\beta$$

where g is an appropriate link function, $\mu_{\mathrm{ij}}=E\left[Y_{\mathrm{ij}}\right],X_{\mathrm{ij}}={\left(1,X_{\mathrm{ij}}\right)}^{T}$ and $\beta ={\left(\beta_{0},\beta_{1}\right)}^{T}$ is a vector of model parameters. The GEE estimates of the model parameters ($\hat{\beta }$) are obtained by solving the estimating equations $\sum_{i=1}^{M}{D_{i}}^{T}{V_{i}}^{-1}\left(Y_{i}-\mu_{i}\right)=0$, where $Y_{i}={\left(Y_{i1},\ldots Y_{in_{i}}\right)}^{T}$, $\mu_{i}=E\left[Y_{i}\right]$, $V_{i}={A_{i}}^{1/2}R_{i}{A_{i}}^{1/2}$, $A_{i}=\mathrm{diag}\left(\mathrm{var}\left(Y_{i1}\right),\ldots \mathrm{var}\left(Y_{in_{i}}\right)\right)$, $R_{i}$ is a working correlation matrix for $Y_{i}$, and $D_{i}=\partial \mu_{i}/\partial \beta$. Let $C_{i}$ denote the true correlation matrix for $Y_{i}$. Then, $\mathrm{cov}\left(Y_{i}\right)={A_{i}}^{1/2}C_{i}{A_{i}}^{1/2}$ and the covariance matrix of the GEE estimates of the model parameters is given by

(2) $$\mathrm{cov}\left(\hat{\beta }\right)={\left[\sum_{i=1}^{M}{D_{i}}^{T}{V_{i}}^{-1}D_{i}\right]}^{-1}\left[\sum_{i=1}^{M}{D_{i}}^{T}{V_{i}}^{-1}\mathrm{cov}\left(Y_{i}\right){V_{i}}^{-1}D_{i}\right]{\left[\sum_{i=1}^{M}{D_{i}}^{T}{V_{i}}^{-1}D_{i}\right]}^{-1}$$

When the working correlation matrix is correct, that is, $R_{i}=C_{i}$, the covariance matrix in Equation (2) simplifies to $\mathrm{cov}\left(\hat{\beta }\right)={\left[\sum_{i=1}^{M}{D_{i}}^{T}{V_{i}}^{-1}D_{i}\right]}^{-1}$ [23, 24]. When g is the identity link, $D_{i}=X_{i}={\left(X_{i1},\ldots X_{in_{i}}\right)}^{T}$. As in other common clustered settings [25, 26], we make the simplifying assumption that the true correlation matrix is exchangeable, that is, $C_{i}=1$ when $n_{i}=1$ and $C_{i}=\left[\begin{array}{llll} 1 & \rho \; & \cdots & \rho \\ \rho & 1 & \ldots & \rho \\ \vdots & \vdots & \ddots & \vdots \\ \rho & \rho & \cdots & 1 \end{array}\right]$ when $n_{i}>1$, where ρ is the ICC (i.e., a measure of the correlation between outcomes of infants from the same birth). This is a reasonable assumption for many partially clustered settings such as multiple births [27] and clusters of multiple body sites per participant, though is less likely to hold in re‐randomization designs and other settings where outcomes are measured over time, since the correlation may diminish over time.

We treat independent observations (i.e., singleton infants) as clusters of size 1 and consider clusters of sizes k=1,…,K. Let $M_{k}$ be the number of clusters of size k (i.e., the number of mothers who give birth to k infants). We assume $M_{1}>0$ and $M_{K}>0$ for some K>1, while allowing for the possibility that $M_{k}=0$ for some k such that 1<k<K. The number of cluster members (observational units) in clusters of size k is denoted $N_{k}=kM_{k}$, and the total number of cluster members is $N=\sum_{k=1}^{K}N_{k}$. The proportion of cluster members that belong to a cluster of size k is denoted $\gamma_{k}=kM_{k}/N$, such that $\sum_{k=1}^{K}\gamma_{k}=1$, and we define $\gamma =\left(\gamma_{1},\ldots ,\gamma_{K}\right)$. Each cluster member may belong to the intervention group (I) or the control group (C). Within cluster i, we denote the number of cluster members that are allocated to the intervention and control groups as $t_{i}$ and $c_{i}$ respectively, such that the total number of cluster members in cluster i is $n_{i}=t_{i}+c_{i}$. The total numbers of cluster members in the intervention and control groups are denoted $N_{I}$ and $N_{C}$, respectively, such that $N_{I}+N_{C}=N$. Finally, we denote the numbers of cluster members in the intervention and control groups within each cluster size by $N_{\mathrm{Ik}}$ and $N_{\mathrm{Ck}}$, respectively, such that $N_{\mathrm{Ik}}+N_{\mathrm{Ck}}=N_{k}$ for k=1,…,K.

**FIGURE 1:**
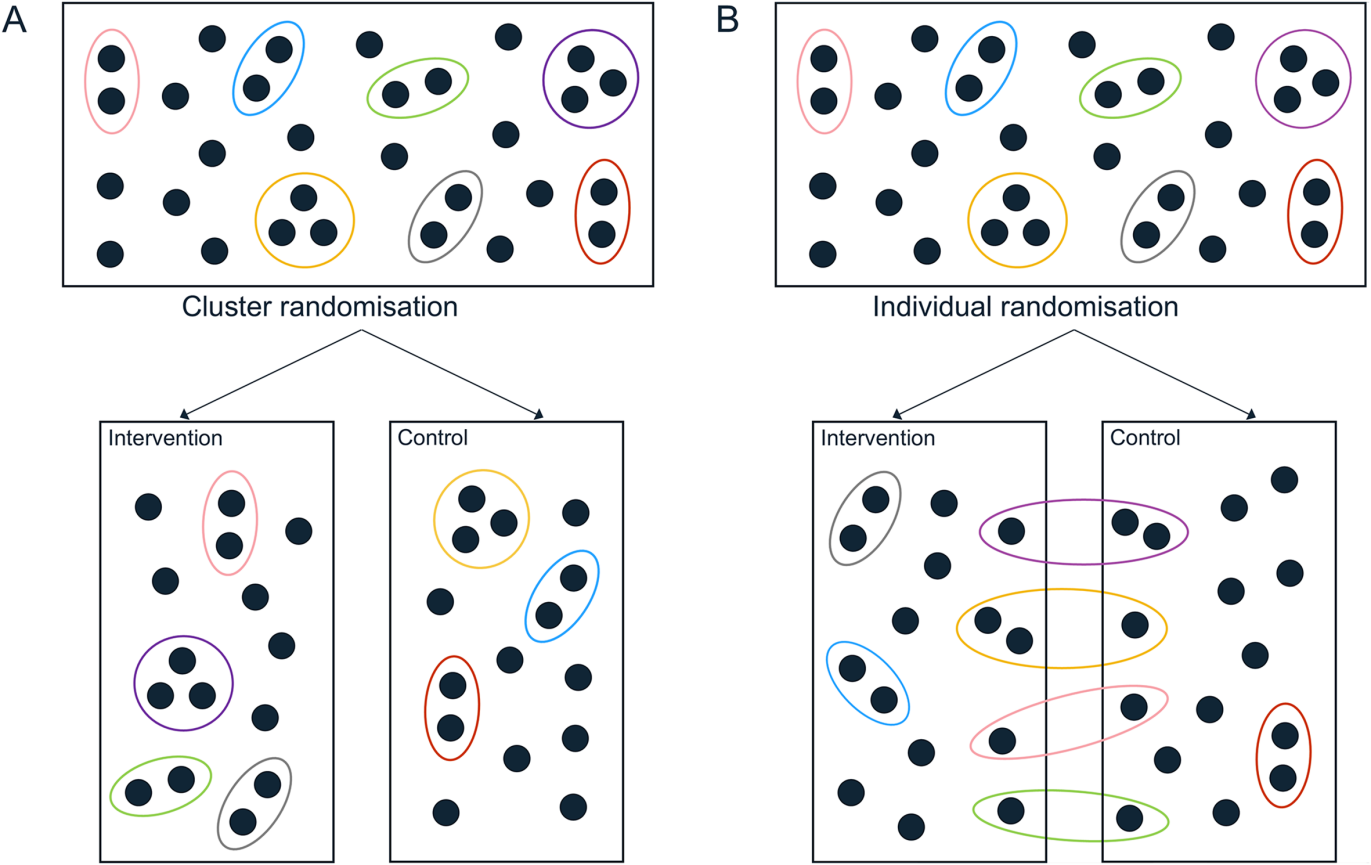
Two‐arm partially clustered designs with pre‐existing clusters. Filled circles represent observational units (cluster members). Ellipses group a cluster of observational units. Observational units may be assigned to treatment arms via (A) cluster randomization, where all observational units within a cluster are assigned to the same treatment arm, or (B) individual randomization, where each observational unit is randomized independently and clusters may therefore include observational units from one, or both, treatment groups.

We assume cluster members (i.e., infants) are randomly assigned to the intervention or control group using cluster or individual randomization. When cluster randomization is used, all members of a cluster are assigned to the same treatment group, and clusters are therefore nested within treatment group. When individual randomization is used, each cluster member is randomized independently, meaning that clusters may contain cluster members assigned to different treatment groups and are therefore cross‐classified with treatment group. Figure 1 illustrates partially clustered trial designs that use (a) cluster randomization, and (b) individual randomization.

### 2.3 Methods for Deriving Design Effects

DEFFs were derived algebraically based on linear, logistic, and log binomial models. For continuous outcomes, a linear model with g equal to the identity link (i.e., $g\left(\mu_{\mathrm{ij}}\right)=\mu_{\mathrm{ij}}$ in Equation (1)) was used to estimate the treatment effect (i.e., $\beta_{1}$) in the form of a difference in means. For binary outcomes, logistic (g = logit link, such that $g\left(\mu_{\mathrm{ij}}\right)=\ln\left(\mu_{\mathrm{ij}}/\left(1-\mu_{\mathrm{ij}}\right)\right)$) and log binomial (g = log link, such that $g\left(\mu_{\mathrm{ij}}\right)=\ln\left(\mu_{\mathrm{ij}}\right)$) models were used to express the treatment effect as an odds ratio or relative risk, respectively. The DEFF for the treatment effect is defined as

(3) $$\text{DEFF}=\frac{\mathrm{var}{\left({\hat{\beta }}_{1}\right)}_{\mathrm{GEE}}}{\mathrm{var}{\left({\hat{\beta }}_{1}\right)}_{\mathrm{IND}}}$$

where the numerator is the variance of the treatment effect from a GEE that accounts for clustering (using an independence or exchangeable working correlation structure), obtained using Equation (2), and the denominator is the variance of the treatment effect assuming all observations are independent. The denominators in Equation (3) were estimated using standard linear regression for continuous outcomes, and logistic or log binomial regression for binary outcomes.

To ensure that the DEFFs only involved terms that could reasonably be estimated when planning a trial, the following simplifying assumptions were made. (1) *Overall treatment balance*: The number of cluster members will be the same in each treatment group, that is, $N_{I}=N_{C}=N/2$, as is typically assumed in sample size calculations for trials when 1:1 randomization is employed. (2) *Treatment balance within cluster size*: The sample size within each cluster size $N_{k}$ will be balanced between treatment groups such that $N_{\mathrm{Ik}}=N_{\mathrm{Ck}}=N_{k}/2$ for k=1,…,K. While this can only be guaranteed when randomization is stratified by cluster size (with even numbers of clusters of each size), it is expected on average when overall treatment balance is achieved. For cluster randomization, assumption (2) implies an equal number of clusters of each size assigned to the intervention and control groups. To meet assumption (2) for 1:1 individual randomization, we additionally assumed that for each cluster size, the clusters of that size consisted of the expected proportion of clusters with each pattern of individual treatment allocation within a cluster, which we refer to as the *expected proportions* assumption. More specifically, the proportion of clusters of size k consisting of t=0 to k cluster members in the intervention group (and therefore k−t cluster members in the control group) is assumed to be kt/2k, as expected when cluster members are exchangeable within clusters and 1:1 randomization is used. For example, with clusters of size 2 it is assumed that 25% of clusters have both members assigned to the control group, 25% have both members assigned to the intervention group, and 50% contain one cluster member assigned to the control group and one member assigned to the intervention group.

### 2.4 Design Effects for Continuous Outcomes

Suppose $Y_{\mathrm{ij}}$ is a continuous outcome with constant variance $\sigma^{2}$. The general formula for the DEFF for an independence working correlation structure is

(4) $$\text{DEFF}=1+\rho \left(\frac{\left(\sum_{i=1}^{M}n_{i}^{2}\right)}{N}-4\frac{\left(\sum_{i=1}^{M}t_{i}c_{i}\right)}{N}-1\right)$$

and for an exchangeable working correlation structure is

(5) $$\text{DEFF}=\frac{\mathrm{NA}}{4\left(\mathrm{AB}-C^{2}\right)}$$

where $A=\sum_{i=1}^{M}\frac{n_{i}}{\left(1+\left(n_{i}-1\right)\rho \right)}$, $B=\sum_{i=1}^{M}\frac{t_{i}\left(1+\left(c_{i}-1\right)\rho \right)}{\left(1-\rho \right)\left(1+\left(n_{i}-1\right)\rho \right)}$, and $C=\sum_{i=1}^{M}\frac{t_{i}}{\left(1+\left(n_{i}-1\right)\rho \right)}$ (see Supporting Information File S1 for details of the derivations). Both DEFFs depend on the ICC and include terms that depend on the number of control, intervention, and total members within each cluster. The latter terms will vary depending on the randomization method used for treatment allocation. The general DEFFs in Equations (4) and (5) can therefore be simplified based on the method of randomization to be used, resulting in the randomization‐specific DEFFs given in Table 1. All of the DEFFs for continuous outcomes depend only on the ICC (ρ), the possible cluster sizes (i.e., the k's), and the proportion of observations that belong to clusters of each cluster size (i.e., the $\gamma_{k}$'s), all of which can be reasonably estimated when planning a trial.

**TABLE 1:** Design effects by type of outcome, method of randomization, and GEE working correlation structure.

Randomization method	Independence working correlation	Exchangeable working correlation
**Continuous outcome—identity link**		
Cluster	$1+\rho \sum_{k=1}^{K}\left(k-1\right)\gamma_{k}$	${\left[\sum_{k=1}^{K}\frac{1}{\;\left(1+\left(k-1\right)\rho \right)}\gamma_{k}\right]}^{-1}$
Individual	1	${\left[\sum_{k=1}^{K}\frac{1+\left(k-2\right)\rho }{\left(1-\rho \right)\left(1+\left(k-1\right)\rho \right)}\gamma_{k}\right]}^{-1}$
**Binary outcome—logit link** ^a^		
Cluster	$1+\rho \sum_{k=1}^{K}\left(k-1\right)\gamma_{k}$	${\left[\sum_{k=1}^{K}\frac{1}{\;\left(1+\left(k-1\right)\rho \right)}\gamma_{k}\right]}^{-1}$
Individual	$1+\rho \left[\left(\frac{1}{2}-\frac{\sqrt{\pi_{I}\pi_{C}\left(1-\pi_{I}\right)\left(1-\pi_{C}\right)}}{\pi_{I}\left(1-\pi_{I}\right)+\pi_{C}\left(1-\pi_{C}\right)}\right)\sum_{k=1}^{K}\left(k-1\right)\gamma_{k}\right]$	$\frac{\sum_{k=1}^{K}\frac{1}{\;\left(1+\left(k-1\right)\rho \right)}\gamma_{k}+\left(\frac{1}{2}-\frac{\sqrt{\pi_{I}\pi_{C}\left(1-\pi_{I}\right)\left(1-\pi_{C}\right)}}{\pi_{I}\left(1-\pi_{I}\right)+\pi_{C}\left(1-\pi_{C}\right)}\right)\left(\frac{\rho }{1-\rho }\right)\left(\sum_{k=1}^{K}\frac{k-1}{\;\left(1+\left(k-1\right)\rho \right)}\gamma_{k}\right)}{{\left(\sum_{k=1}^{K}\frac{1}{\;\left(1+\left(k-1\right)\rho \right)}\gamma_{k}\right)}^{2}+\left(\frac{\rho }{1-\rho }\right)\left(\sum_{k=1}^{K}\frac{1}{\;\left(1+\left(k-1\right)\rho \right)}\gamma_{k}\right)\left(\sum_{k=1}^{K}\frac{k-1}{\;\left(1+\left(k-1\right)\rho \right)}\gamma_{k}\right)}$

^a^ DEFFs for a binary outcome with a log link are presented in Table S1.

**FIGURE 2:**
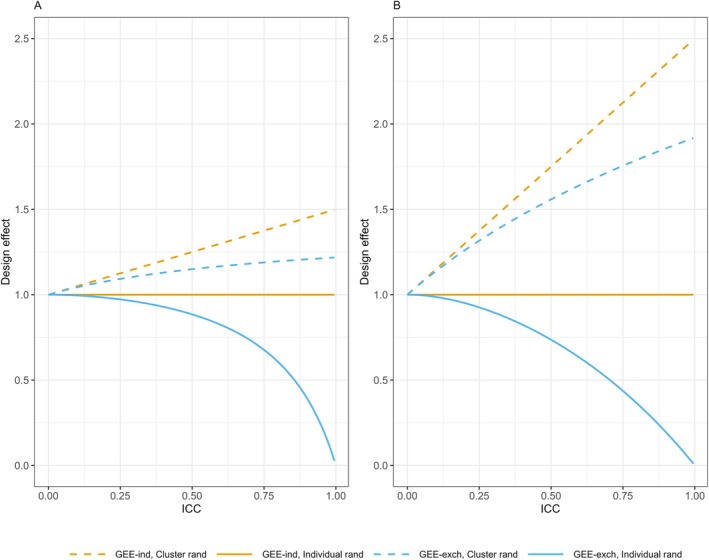
Design effects for a continuous outcome by randomization method and GEE working correlation structure. Figure shows the design effects for varying ICC with a maximum cluster size of 4 and proportions of clusters of sizes 1–4 of (A) (0.70, 0.15, 0.10. 0.05) (corresponding to γ = (0.47, 0.20, 0.20, 0.13)) and (B) (0.25, 0.25, 0.25, 0.25) (corresponding to γ = (0.1, 0.2, 0.3, 0.4)). Design effects are shown for generalized estimating equations (GEEs) with an independence (GEE‐ind) or exchangeable (GEE‐exch) working correlation structure under cluster or individual randomization of the clustered observations.

Assuming outcomes of observations within a cluster are positively correlated, as is typically the case, the DEFFs for cluster randomization are greater than 1, and as expected, increase with increasing ICC. The DEFF for the independence GEE is greater than for the exchangeable GEE and the difference between them increases with increasing ICC (Figure 2). The DEFF for individual randomization is equal to 1 when the independence working correlation structure is used for the GEE, and less than 1 for the exchangeable GEE, becoming smaller as the ICC increases. For both cluster and individual randomization, the absolute magnitudes of the DEFFs are greater when there are more larger clusters and fewer independent observations.

### 2.5 Design Effects for Binary Outcomes

Suppose $Y_{\mathrm{ij}}$ is a binary outcome with prevalence $\pi_{I}$ in the intervention group and $\pi_{C}$ in the control group, and an analysis will be performed with a logit link function to estimate an odds ratio. The general formulas for the DEFFs based on the independence and exchangeable working correlation structures are both complex equations involving $\pi_{I}$, $\pi_{C}$, the ICC and terms that depend on the number of control and intervention members within each cluster (see Supporting Information File S1). By considering specific methods of randomization, the DEFFs can be simplified to the randomization‐specific forms presented in Table 1.

For cluster randomization, the DEFFs for binary outcomes are identical to those for continuous outcomes, and depend only on the ICC, the possible cluster sizes, and the proportion of observations that belong to clusters of each size. For individual randomization, the DEFFs are more complex than the continuous case due to their dependence on the outcome prevalence in the intervention and control groups via the term

(6) $$\frac{\sqrt{\pi_{I}\pi_{C}\left(1-\pi_{I}\right)\left(1-\pi_{C}\right)}}{\pi_{I}\left(1-\pi_{I}\right)+\pi_{C}\left(1-\pi_{C}\right)}$$

however, the required parameters can still be estimated before a trial commences and the DEFFs are computationally straightforward, despite their complicated appearance. For individual randomization, the DEFFs for both the independence and exchangeable correlation structures will be identical to those for a continuous outcome when $\pi_{I}=\pi_{C}$ or $\pi_{I}=1-\pi_{C}$, that is, when Equation (6) equals 0.5, and similar otherwise. The values of the DEFFs for varying ICCs at fixed outcome prevalence values ($\pi_{C}$ = 0.4, $\pi_{I}$ = 0.3) are shown in Figure S1, with the DEFFs for individual randomization being very similar to the continuous DEFFs in this case (see Figure S1 vs. Figure 2).

**FIGURE 3:**
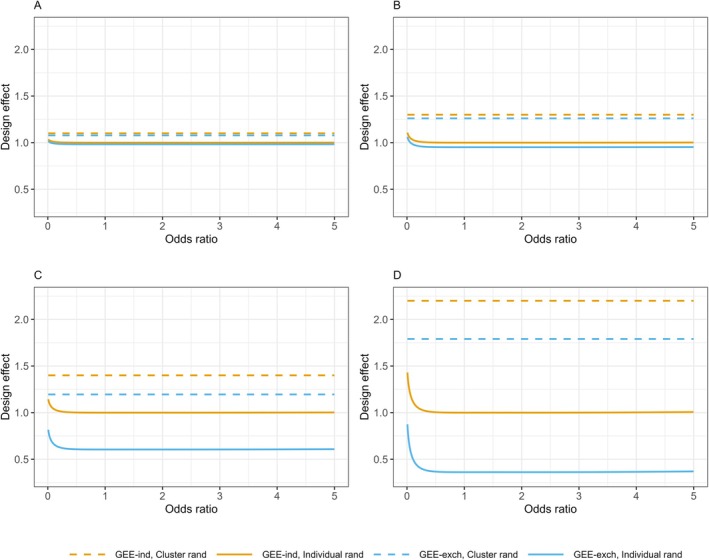
Design effects for a binary outcome with a logit link by randomization method and GEE working correlation structure. Figure shows the design effects for varying odds ratio with a maximum cluster size of 4, outcome prevalence in the control group of $\pi_{C}$ = 0.4, outcome prevalence in the intervention group ranging from $\pi_{I}$ = 0.005 to 0.77 to produce the varying odds ratio, and (A) proportions of clusters of sizes 1–4 of (0.70, 0.15, 0.10. 0.05) (corresponding to γ = (0.47, 0.20, 0.20, 0.13)) and ICC = 0.2, (B) proportions of clusters of sizes 1–4 of (0.25, 0.25, 0.25, 0.25) (corresponding to γ = (0.1, 0.2, 0.3, 0.4)) and ICC = 0.2, (C) proportions of clusters of sizes 1–4 of (0.70, 0.15, 0.10. 0.05) (corresponding to γ = (0.47, 0.20, 0.20, 0.13)) and ICC = 0.8, (D) proportions of clusters of sizes 1–4 of (0.25, 0.25, 0.25, 0.25) (corresponding to γ = (0.1, 0.2, 0.3, 0.4)) and ICC = 0.8. Design effects are shown for generalized estimating equations (GEEs) with an independence (GEE‐ind) or exchangeable (GEE‐exch) working correlation structure under cluster or individual randomization of the clustered observations.

For individual randomization, the ICC and distribution of cluster sizes can have marked effects on the resulting DEFF, but the prevalence values are less influential. For both correlation structures, the DEFFs are relatively constant for varying odds ratios, except when the odds ratio is very small (Figure 3), and are largely unaffected by the value of the outcome prevalence in the control group (see Figure 3 vs. Figure S2). This indicates that in most practical cases the DEFFs will be robust to the choice of prevalence estimates and similar to the DEFFs for continuous outcomes.

If a binary outcome will be analyzed using the log link function to estimate a relative risk, the DEFFs are identical to those for the logit link, except that the term $\pi_{I}\left(1-\pi_{I}\right)+\pi_{C}\left(1-\pi_{C}\right)$ is replaced by $\pi_{I}\left(1-\pi_{C}\right)+\pi_{C}\left(1-\pi_{I}\right)$ for individual randomization (Table S1, Figure S3). The DEFFs for individual randomization are slightly more sensitive to the choice of prevalence estimates for the log link compared to the logit link (Figures S4 and S5), particularly when the ICC is high and there are more larger clusters and fewer independent observations. However, as in the logit link case, DEFFs are relatively constant for the range of effect sizes most commonly seen in practice.

### 2.6 Applying Design Effects in Sample Size Calculations

To determine the required sample size for a partially clustered trial, the first step involves calculating the sample size assuming all observations are independent. Methods for a two‐sample *t*‐test or chi‐square test (with or without a continuity correction) can be used for a continuous or binary outcome, respectively. An appropriate DEFF formula is then chosen, depending on the randomization method to be used for the clustered observations and the chosen working correlation structure. To calculate the DEFF, estimates of $\gamma =\left(\gamma_{1},\ldots ,\gamma_{K}\right)$, the proportion of observations that belong to clusters of each size, are required (e.g., the proportion of infants expected from a single, twin, and triplet birth). In some cases, trialists may instead have estimates of the proportion of clusters of each cluster size (e.g., the proportion of women that are expected to be pregnant with each order of multiples). The following formula can then be used to calculate the $\gamma_{k}$ terms:

(7) $$\gamma_{k}=\frac{k\delta_{k}}{\sum_{k=1}^{K}k\delta_{k}}$$

where $\delta_{k}$ is the proportion of clusters that are of size k. Conversely, the proportion of clusters of each size, $\delta =\left(\delta_{1},\ldots ,\delta_{K}\right)$, can be derived from the $\gamma_{k}$ as:

$$\delta_{k}=\frac{\gamma_{k}/k}{\sum_{k=1}^{K}\gamma_{k}/k}$$

The sample size assuming independence is multiplied by the DEFF to produce the target sample size accounting for clustering in terms of the number of observations that are required. A recruitment target of cluster members (observational units) may be useful for some trials; for example, in neonatal trials in which newborn infants are recruited. However, in many other settings the recruitment target will be expressed as the number of clusters (e.g., in perinatal trials in which pregnant women [clusters] are recruited, with outcomes to be measured on their infants [cluster members]). When that is the case, the target number of observations can be converted to the target number of clusters using the proportions $\gamma_{k}$ in the DEFF. Specifically, if N is the target number of observations, then the required number of clusters is:

(8) $$M=N\sum_{k=1}^{K}\gamma_{k}/k$$

This process for calculating the sample size is illustrated through examples in Section 4.

## 3 Simulation Study

### 3.1 Simulation Methods

A simulation study was conducted to validate the DEFFs derived in the previous section. The primary aim was to assess the accuracy of the derived DEFFs. The secondary aim was to compare theoretical power (obtained through application of the derived DEFFs) to empirical power. Partially clustered datasets were generated with a specified number of clusters of sizes 1–4 (i.e., K=4), to reflect cluster sizes that may occur in trials that include multiple births or use a re‐randomization design. Clustered observations were allocated to one of two treatment groups using individual or cluster randomization. Independent (non‐clustered) observations were randomized independently. To match the assumptions made in deriving the theoretical DEFFs, randomization was performed such that treatment groups were balanced overall and within cluster size (assumptions (1) and (2) in Section 2.3). To avoid the need for overly large sample sizes, the expected proportions assumption of the pattern of individual treatment allocation within a cluster for individual randomization was not implemented for each dataset, but is assumed to hold on average across all replicated datasets. For each scenario, 10 000 datasets were generated, resulting in a Monte Carlo standard error for an observed power of 85% of 0.4%.

Continuous outcomes were randomly generated from the linear mixed model $Y_{\mathrm{ij}}=\beta_{0}+\beta_{1}X_{\mathrm{ij}}+\alpha_{i}+\varepsilon_{\mathrm{ij}}$, where $\alpha_{i}∼N\left(0,\sigma_{\alpha }^{2}\right)$ represents a random cluster effect, and $\varepsilon_{\mathrm{ij}}∼N\left(0,\sigma_{\varepsilon }^{2}\right)$ is a random error term. The model coefficients were set to $\beta_{0}=0$ and $\beta_{1}=0.25$. Variances were chosen such that the total variance was fixed at 1 (i.e., $\sigma_{\alpha }^{2}+\sigma_{\epsilon }^{2}=1$) and the desired ICC was produced ($\mathrm{ICC}=\sigma_{\alpha }^{2}/\left(\sigma_{\alpha }^{2}+\sigma_{\epsilon }^{2}\right)$). Binary outcomes were generated using the method of Jiang et al. [28] to create datasets with marginal prevalence of the outcome of 0.4 in the control group and 0.3 in the intervention group (odds ratio = 0.64, relative risk = 0.75), and with the desired ICC. Total sample sizes of *N* = 240 and 600 were assessed for continuous outcomes, and *N* = 360 and 840 were used for binary outcomes. Sample sizes were chosen to be divisible among the four cluster sizes in the ratios of interest (described below). For each type of outcome, the larger sample size was chosen to have 85% power for detecting the effect size specified in the data generation process when all observations are independent (i.e., a mean difference of 0.25 for continuous outcomes and an odds ratio of 0.64 for binary outcomes). A smaller total sample size was also included so that the applicability of the theoretical DEFFs could be assessed in settings with fewer clusters and lower power (˜50% when all observations are independent).

The ICC was set to 0.2 or 0.8, representing the range of values observed in neonatal trials [29]. Two distributions of cluster sizes were considered: (a) unequal cluster proportions, where clusters consisted of 70% independent observations (clusters of size 1), 15% clusters of size 2, 10% clusters of size 3, and 5% clusters of size 4 (i.e., δ = (0.70, 0.15, 0.10, 0.05) and hence γ = (0.47, 0.20, 0.20, 0.13) from Equation (7)); and (b) equal cluster proportions, where 25% of clusters were of each cluster size (i.e., δ = (0.25, 0.25, 0.25, 0.25) and γ = (0.1, 0.2, 0.3, 0.4)). The first distribution was chosen to reflect realistic values for trials in preterm infants, where multiple births are more common than in the general population, and re‐randomization trials that include multiple episodes per participant [30, 31, 32, 33]. Equal cluster proportions were included to assess performance in more extreme settings where most observations are members of larger clusters and hence DEFFs are further from 1. Table 2 shows the number of clusters and observations by cluster size for each sample size and distribution of cluster sizes. A full factorial design was implemented, resulting in 16 simulation scenarios (2 methods of randomization × 2 sample sizes × 2 ICCs × 2 distributions of cluster sizes) for each combination of outcome type and link function (continuous with identity link, binary with logit link, binary with log link). The expected DEFF was calculated for each scenario, outcome type, and link function using the formulas in Table 1 and Table S1.

**TABLE 2:** Sample sizes used for scenarios in the simulation study. Each row represents a combination of distribution of cluster sizes and total sample size that was simulated with ICC = 0.2 and 0.8, and with cluster and individual randomization.

Distribution of cluster sizes	Total number of observations (N)	Number (proportion) of clusters				Number (proportion) of observational units			
		$M_{1}$ $\left(\delta_{1}\right)$	$M_{2}\;\left(\delta_{2}\right)$	$M_{3}\;\left(\delta_{3}\right)$	$M_{4}\;\left(\delta_{4}\right)$	$N_{1}\;\left(\gamma_{1}\right)$	$N_{2}\;\left(\gamma_{2}\right)$	$N_{3}\;\left(\gamma_{3}\right)$	$N_{4}\;\left(\gamma_{4}\right)$
**Continuous outcome**									
Unequal cluster proportions	240	112 (0.70)	24 (0.15)	16 (0.10)	8 (0.05)	112 (0.47)	48 (0.20)	48 (0.20)	32 (0.13)
Unequal cluster proportions	600	280 (0.70)	60 (0.15)	40 (0.10)	20 (0.05)	280 (0.47)	120 (0.20)	120 (0.20)	80 (0.13)
Equal cluster proportions	240	24 (0.25)	24 (0.25)	24 (0.25)	24 (0.25)	24 (0.10)	48 (0.20)	72 (0.30)	96 (0.40)
Equal cluster proportions	600	60 (0.25)	60 (0.25)	60 (0.25)	60 (0.25)	60 (0.10)	120 (0.20)	180 (0.30)	240 (0.40)
**Binary outcome**									
Unequal cluster proportions	360	168 (0.70)	36 (0.15)	24 (0.10)	12 (0.05)	168 (0.47)	72 (0.20)	72 (0.20)	48 (0.13)
Unequal cluster proportions	840	392 (0.70)	84 (0.15)	56 (0.10)	28 (0.05)	392 (0.47)	168 (0.20)	168 (0.20)	112 (0.13)
Equal cluster proportions	360	36 (0.25)	36 (0.25)	36 (0.25)	36 (0.25)	36 (0.10)	72 (0.20)	108 (0.30)	144 (0.40)
Equal cluster proportions	840	84 (0.25)	84 (0.25)	84 (0.25)	84 (0.25)	84 (0.10)	168 (0.20)	252 (0.30)	336 (0.40)

*Note:* $M_{k}$ = the number of clusters of size k. $N_{k}$ = the number of observational units that belong to clusters of size k. The total number of observational units (the total sample size) is denoted by $N=\sum_{k=1}^{4}N_{k}$. $\delta_{k}$ = the proportion of clusters that are of size k. $\gamma_{k}$ = the proportion of observational units that belong to clusters of size k.

Each dataset was analyzed using GEEs with independence and exchangeable working correlation structures and the sandwich variance estimator to estimate the treatment effect, $\beta_{1}$. In the continuous outcome setting, $\beta_{1}$ is the difference in means, with marginal and conditional treatment effects equivalent. In the binary outcome setting, $\;\beta_{1}$ was estimated using both a logit link (leading to the marginal odds ratio) and a log link (producing a marginal relative risk). For each simulated dataset, the observed DEFF was calculated as the estimated variance of the treatment effect obtained using GEEs, divided by the estimated variance obtained using standard regression (i.e., not accounting for clustering of observations). To address our primary aim, the percentage relative difference in the observed and expected DEFFs was calculated for each dataset as 100 × (DEFF_obs_−DEFF_exp_)/DEFF_exp_, and the median across the replicated datasets for each scenario is reported. To address our secondary aim, the absolute difference between the observed and expected power, was calculated as Power_obs_−Power_exp_. The observed power was defined as the proportion of simulated datasets where *p* ≤ 0.05, where *p* = *p*‐value from the hypothesis test for $\beta_{1}=0$. The expected power was calculated based on a Wald test for independent data using the effective sample size, given by the actual sample size divided by the expected DEFF. The rates of non‐convergence for both GEE methods, and the rate of non‐positive definite models for the exchangeable GEE (identified by an estimated correlation coefficient < −1 or > 1) were also calculated for each scenario. Datasets where models did not converge were excluded from the calculation of other results, while results of non‐positive definite model fits were retained.

In sensitivity analyses, the simulation study was repeated under relaxed assumptions that are more realistic in practice. First, for cluster randomization, an equal number of clusters were assigned to each treatment group without stratification by cluster size. This resulted in the treatment groups being balanced overall on average, but the assumptions of overall treatment group balance and treatment group balance within cluster size (see Section 2.3) were not guaranteed in individual datasets. For individual randomization, treatment groups were balanced overall for each dataset, but not within cluster size. Second, instead of the number of clusters of each size being constant across replicated datasets for each scenario, the $M_{k}$ were generated randomly from a multinomial distribution with the total number of clusters (M) fixed at the same values used in the main simulation study, and probabilities equal to $\delta =\left(\delta_{1},\ldots ,\delta_{K}\right)$. The total number of observations (N) therefore varied across datasets but approximately matched the sample size used in the main simulation study on average.

Analyses were conducted in R 4.3.3 with GEEs fit using the geepack::geeglm() function [34] and models assuming independence fit using lm() for continuous outcomes and glm() for binary outcomes. The CorBin package was used to simulate the binary outcomes [28]. Simulation code is available at https://github.com/klange01/partial‐clustering‐deffs.

### 3.2 Results for Continuous Outcomes

All models converged for all scenarios and therefore no datasets were excluded from the calculation of the observed DEFFs and power. GEEs with an exchangeable working correlation structure occasionally resulted in non‐positive definite model fits in scenarios when the ICC was 0.8 (maximum 9.5% of datasets; data not shown) but these results were retained. The highest rates of non‐positive definite fits occurred when unequal cluster proportions were used at the smaller sample size, resulting in the fewest number of clusters of sizes 3 and 4. Observed DEFFs (empirical results from the simulated datasets) ranged from 0.95 to 2.58, while expected DEFFs (calculated using the theoretical formulas from Section 2) ranged from 1.00 to 2.60 (maximum absolute difference between observed and expected DEFFs = 0.05; Table 3). The percent relative difference between observed and expected DEFFs was small for all scenarios when cluster randomization was used, particularly for the larger sample size (within 2.9% when *N* = 240 and 1% when *N* = 600). In all scenarios, the observed DEFF was slightly lower than expected. Correspondingly, the differences in power were also small for cluster randomization (Table 4), with expected power based on the DEFF underestimating the observed power by up to 2.6 percentage points.

**TABLE 3:** Observed and expected design effects for a continuous outcome.

				GEE independence			GEE exchangeable		
Randomization method	Sample size	Distribution of cluster sizes	ICC	Observed DEFF^a^	Expected DEFF	Relative difference (%)^a^	Observed DEFF^a^	Expected DEFF	Relative difference (%)^a^
Cluster	240	Unequal cluster proportions	0.2	1.17	1.20	−2.54	1.14	1.16	−2.02
Cluster	240	Unequal cluster proportions	0.8	1.75	1.80	−2.85	1.44	1.44	−0.42
Cluster	240	Equal cluster proportions	0.2	1.36	1.40	−2.86	1.33	1.37	−2.59
Cluster	240	Equal cluster proportions	0.8	2.55	2.60	−2.00	2.22	2.25	−1.22
Cluster	600	Unequal cluster proportions	0.2	1.19	1.20	−0.96	1.15	1.16	−0.76
Cluster	600	Unequal cluster proportions	0.8	1.78	1.80	−0.98	1.44	1.44	−0.22
Cluster	600	Equal cluster proportions	0.2	1.39	1.40	−1.03	1.35	1.37	−1.00
Cluster	600	Equal cluster proportions	0.8	2.58	2.60	−0.91	2.24	2.25	−0.43
Individual	240	Unequal cluster proportions	0.2	0.98	1.00	−2.09	0.95	0.97	−1.87
Individual	240	Unequal cluster proportions	0.8	0.96	1.00	−4.29	0.48	0.45	6.04
Individual	240	Equal cluster proportions	0.2	0.97	1.00	−2.53	0.91	0.94	−2.80
Individual	240	Equal cluster proportions	0.8	0.95	1.00	−4.99	0.31	0.31	−1.15
Individual	600	Unequal cluster proportions	0.2	0.99	1.00	−0.93	0.96	0.97	−0.91
Individual	600	Unequal cluster proportions	0.8	0.98	1.00	−1.87	0.46	0.45	3.38
Individual	600	Equal cluster proportions	0.2	0.99	1.00	−1.05	0.93	0.94	−1.12
Individual	600	Equal cluster proportions	0.8	0.98	1.00	−1.97	0.31	0.31	−0.60

*Note:* Clusters of size 1–4 were distributed in proportions (0.70, 0.15, 0.10, 0.05) in scenarios with unequal cluster proportions (corresponding to γ = (0.47, 0.20, 0.20, 0.13)), and (0.25, 0.25, 0.25, 0.25) in scenarios with equal cluster proportions (corresponding to γ = (0.1, 0.2, 0.3, 0.4)).

Abbreviations: DEFF = design effect, GEE = generalized estimating equation, ICC = intracluster correlation coefficient.

^a^ Median value across 10000 simulated datasets.

**TABLE 4:** Observed and expected power for a continuous outcome.

				GEE independence			GEE exchangeable		
Randomization method	Sample size	Distribution of cluster sizes	ICC	Observed power	Expected power	Absolute difference	Observed power	Expected power	Absolute difference
Cluster	240	Unequal cluster proportions	0.2	42.72	42.05	0.67	44.33	43.14	1.19
Cluster	240	Unequal cluster proportions	0.8	32.45	29.94	2.51	37.70	36.10	1.60
Cluster	240	Equal cluster proportions	0.2	39.13	36.99	2.14	40.34	37.71	2.63
Cluster	240	Equal cluster proportions	0.8	23.59	22.10	1.49	26.01	24.85	1.16
Cluster	600	Unequal cluster proportions	0.2	80.34	79.67	0.67	81.72	80.88	0.84
Cluster	600	Unequal cluster proportions	0.8	62.50	62.39	0.11	71.74	72.06	−0.32
Cluster	600	Equal cluster proportions	0.2	74.56	73.30	1.26	75.64	74.28	1.36
Cluster	600	Equal cluster proportions	0.8	48.24	47.25	0.99	53.12	52.92	0.20
Individual	240	Unequal cluster proportions	0.2	49.55	48.76	0.79	51.15	50.11	1.04
Individual	240	Unequal cluster proportions	0.8	51.28	48.76	2.52	78.58	82.20	−3.62
Individual	240	Equal cluster proportions	0.2	49.91	48.76	1.15	52.44	51.32	1.12
Individual	240	Equal cluster proportions	0.8	52.69	48.76	3.93	93.38	93.48	−0.10
Individual	600	Unequal cluster proportions	0.2	86.30	86.37	−0.07	87.50	87.50	−0.00
Individual	600	Unequal cluster proportions	0.8	86.20	86.37	−0.17	99.33	99.54	−0.21
Individual	600	Equal cluster proportions	0.2	86.62	86.37	0.25	88.94	88.46	0.48
Individual	600	Equal cluster proportions	0.8	87.17	86.37	0.80	99.97	99.98	−0.01

*Note:* Clusters of size 1–4 were distributed in proportions (0.70, 0.15, 0.10, 0.05) in scenarios with unequal cluster proportions (corresponding to γ = (0.47, 0.20, 0.20, 0.13)), and (0.25, 0.25, 0.25, 0.25) in scenarios with equal cluster proportions (corresponding to γ = (0.1, 0.2, 0.3, 0.4)).

Abbreviations: GEE = generalized estimating equation, ICC = intracluster correlation coefficient.

When individual randomization was used, the observed DEFF for the independence GEE was slightly less than expected, and this difference decreased with the total sample size (minimum relative difference −5.0% when *N* = 240 and − 2.0% when *N* = 600). The differences in power were also small for the independence GEE in this setting, with the expected power underestimating the observed power by up to 3.9 percentage points. When an exchangeable working correlation structure was used with individual randomization, the observed DEFFs were slightly less than expected for all scenarios (minimum relative difference −2.8%), except when unequal cluster proportions was combined with ICC = 0.8. In this case, the observed DEFF was 6.0% larger than expected at the smaller sample size, and 3.4% larger than expected at the larger sample size. The differences in power when the exchangeable GEE was used were small and within ±1.1 percentage points for all scenarios, except for the setting with unequal cluster proportions, ICC = 0.8 and *N* = 240, where the expected DEFF overestimated the observed power by 3.6 percentage points.

### 3.3 Results for Binary Outcomes

Simulation results for binary outcomes analyzed with a logit link are shown in Tables 5 and 6. All models converged for all scenarios and results are therefore based on all 10 000 replicated datasets. Non‐positive definite model fits for the GEE with an exchangeable working correlation structure occurred in similar scenarios as in the continuous setting (i.e., ICC = 0.8 and the smaller sample size), though less than 1.1% of replicated datasets were affected (data not shown). Observed DEFFs ranged from 0.30 to 2.59, and expected DEFFs ranged from 0.31 to 2.60. When cluster randomization was used, all scenarios resulted in small relative differences in the DEFFs and the differences decreased with increasing sample size (within 1.3% when *N* = 360 and 0.5% when *N* = 840). As for continuous outcomes, the observed DEFF was slightly lower than expected for all scenarios. The differences between the observed and expected power were also small under cluster randomization, with the expected power based on the DEFF generally underestimating the observed power by up to 1.4 percentage points.

**TABLE 5:** Observed and expected design effects for a binary outcome with a logit link.

				GEE independence			GEE exchangeable		
Randomization method	Sample size	Distribution of cluster sizes	ICC	Observed DEFF^a^	Expected DEFF	Relative difference (%)^a^	Observed DEFF^a^	Expected DEFF	Relative difference (%)^a^
Cluster	360	Unequal cluster proportions	0.2	1.19	1.20	−0.93	1.16	1.16	−0.56
Cluster	360	Unequal cluster proportions	0.8	1.79	1.80	−0.77	1.44	1.44	−0.33
Cluster	360	Equal cluster proportions	0.2	1.38	1.40	−1.26	1.36	1.37	−0.95
Cluster	360	Equal cluster proportions	0.8	2.58	2.60	−0.59	2.24	2.25	−0.40
Cluster	840	Unequal cluster proportions	0.2	1.19	1.20	−0.52	1.16	1.16	−0.31
Cluster	840	Unequal cluster proportions	0.8	1.79	1.80	−0.30	1.44	1.44	−0.11
Cluster	840	Equal cluster proportions	0.2	1.39	1.40	−0.43	1.36	1.37	−0.34
Cluster	840	Equal cluster proportions	0.8	2.59	2.60	−0.22	2.25	2.25	−0.11
Individual	360	Unequal cluster proportions	0.2	0.99	1.00	−0.93	0.96	0.97	−1.15
Individual	360	Unequal cluster proportions	0.8	0.98	1.00	−2.12	0.44	0.45	−1.36
Individual	360	Equal cluster proportions	0.2	0.99	1.00	−1.07	0.93	0.94	−1.25
Individual	360	Equal cluster proportions	0.8	0.98	1.00	−2.57	0.30	0.31	−2.71
Individual	840	Unequal cluster proportions	0.2	1.00	1.00	−0.41	0.96	0.97	−0.43
Individual	840	Unequal cluster proportions	0.8	0.99	1.00	−0.93	0.45	0.45	−1.19
Individual	840	Equal cluster proportions	0.2	1.00	1.00	−0.40	0.93	0.94	−0.51
Individual	840	Equal cluster proportions	0.8	0.99	1.00	−1.23	0.31	0.31	−1.26

*Note:* Clusters of size 1–4 were distributed in proportions (0.70, 0.15, 0.10, 0.05) in scenarios with unequal cluster proportions (corresponding to γ = (0.47, 0.20, 0.20, 0.13)), and (0.25, 0.25, 0.25, 0.25) in scenarios with equal cluster proportions (corresponding to γ = (0.1, 0.2, 0.3, 0.4)).

Abbreviations: DEFF = design effect, GEE = generalized estimating equation, ICC = intracluster correlation coefficient.

^a^ Median value across 10 000 simulated datasets.

**TABLE 6:** Observed and expected power for a binary outcome with a logit link.

				GEE independence			GEE exchangeable		
Randomization method	Sample size	Distribution of cluster sizes	ICC	Observed power	Expected power	Absolute difference	Observed power	Expected power	Absolute difference
Cluster	360	Unequal cluster proportions	0.2	44.87	44.05	0.82	45.85	45.17	0.68
Cluster	360	Unequal cluster proportions	0.8	32.41	31.43	0.98	38.42	37.86	0.56
Cluster	360	Equal cluster proportions	0.2	39.96	38.79	1.17	40.92	39.54	1.38
Cluster	360	Equal cluster proportions	0.8	22.97	23.20	−0.23	25.95	26.09	−0.14
Cluster	840	Unequal cluster proportions	0.2	78.86	79.15	−0.29	80.35	80.38	−0.03
Cluster	840	Unequal cluster proportions	0.8	62.34	61.82	0.52	71.70	71.50	0.20
Cluster	840	Equal cluster proportions	0.2	72.75	72.75	0.00	73.84	73.73	0.11
Cluster	840	Equal cluster proportions	0.8	47.36	46.77	0.59	52.89	52.39	0.50
Individual	360	Unequal cluster proportions	0.2	51.33	50.95	0.38	52.81	52.34	0.47
Individual	360	Unequal cluster proportions	0.8	52.61	50.93	1.68	90.72	84.18	6.54
Individual	360	Equal cluster proportions	0.2	51.75	50.94	0.81	54.62	53.57	1.05
Individual	360	Equal cluster proportions	0.8	51.74	50.89	0.85	99.56	94.52	5.04
Individual	840	Unequal cluster proportions	0.2	85.94	85.93	0.01	87.12	87.08	0.04
Individual	840	Unequal cluster proportions	0.8	86.06	85.91	0.15	99.98	99.49	0.49
Individual	840	Equal cluster proportions	0.2	85.07	85.92	−0.85	87.15	88.05	−0.90
Individual	840	Equal cluster proportions	0.8	86.40	85.88	0.52	100.00	99.98	0.02

*Note:* Clusters of size 1–4 were distributed in proportions (0.70, 0.15, 0.10, 0.05) in scenarios with unequal cluster proportions (corresponding to γ = (0.47, 0.20, 0.20, 0.13)), and (0.25, 0.25, 0.25, 0.25) in scenarios with equal cluster proportions (corresponding to γ = (0.1, 0.2, 0.3, 0.4)).

Abbreviations: GEE = generalized estimating equation, ICC = intracluster correlation coefficient.

When individual randomization was used for the clustered observations, the expected DEFFs for both GEE methods slightly underestimated the observed DEFFs, but the differences were small (minimum percent relative difference −2.7% when *N* = 360 and −1.3% when *N* = 840). The expected power for the independence GEE generally underestimated the observed power but by no more than 1.7 percentage points. Differences in power for the exchangeable GEE were generally also small, with the expected power underestimating the observed power by up to 1.1 percentage points. The exception to this was when the smaller sample size was combined with ICC = 0.8, when the expected power underestimated the observed power by up to 6.5 percentage points.

Results for binary outcomes analyzed with a log link were very similar (Tables S2 and S3). In both sensitivity analyses, where firstly the assumption of treatment balance within cluster size was relaxed, and secondly the number of clusters of each size varied across replicates, the observed DEFFs and power were very similar to the main results for all scenarios (Tables S4–S15).

## 4 Application to Example Trials

We illustrate how the presented DEFF formulas and process described in Section 2.6 can be used to calculate the target sample size when planning a partially clustered trial through two examples.

First, we revisit the motivating example introduced in Section 2.1. We extend the hypothetical neonatal trial described in Yelland et al. [13] involving singletons and twins by allowing higher‐order multiple births to be included. Suppose a trial is to be conducted to assess the effect of a new infant formula on neonatal morbidity among infants born preterm. Infants born before 37 weeks' gestation will be eligible, with mothers approached for consent to randomize their infants to either a standard formula or the new formula. It is anticipated that 70% of eligible infants will be singletons, 25% twins, and 5% triplets (i.e., γ = (0.70, 0.25, 0.05)). The sample size calculations will assume an ICC for morbidity of 0.5, and a prevalence of 20% with the standard infant formula. The minimum clinically important effect is a reduction in morbidity to 14% with the new formula (odds ratio = 0.65). The sample size for a trial in singletons only can be calculated based on a continuity‐corrected chi‐square test with a two‐sided alpha of 0.05, and results in a target sample size of 647 infants/mothers per group (1294 overall) for 80% power. This sample size can be multiplied by a DEFF from Table 1 to determine the sample size (number of infants) required to account for the partial clustering induced by the inclusion of infants from multiple births. Parents of multiples may prefer that their infants receive the same treatment [35], and therefore cluster randomization will be used for the twins and triplets. The appropriate DEFF for the GEE with an independence correlation structure is 1.18 and for the exchangeable GEE is 1.12, resulting in sample sizes of 764 and 725 infants per group (corresponding to 644 and 611 mothers per group, calculated using Equation (8)) respectively. These designs require substantially more infants per group than a trial consisting only of independent observations (117 and 78 more, respectively), though fewer mothers are needed to consent to the trial (3 and 36 fewer per group, respectively), and results will be more generalizable.

Some trials in preterm populations exclude triplets and higher order pregnancies. If we applied this exclusion criteria to our example, we would expect 73.7% of eligible infants to be singletons and 26.3% twins (i.e., γ = (0.737, 0.263)). This cluster size distribution results in a DEFF of 1.13 for the independence GEE (compared to 1.18 when triplets were included), and 1.10 for the exchangeable GEE (compared to 1.12). The target sample sizes therefore reduce to 732 and 712 infants per group, respectively (compared to 764 and 725). In this example, broadening the trial's inclusion criteria to include triplets requires a larger sample size than when only singletons and twins are included, but may aid in recruitment and result in more generalizable conclusions. Ignoring the inclusion of triplets during the planning stage risks the trial being underpowered.

As a second example, consider a randomized trial designed to assess an intervention for treating severe asthma exacerbations in children that require treatment in hospital. Eligible participants include patients aged 2–16 presenting to the emergency department with acute severe asthma, and those whose caregiver consents will be randomized to nebulized salbutamol or placebo. The primary outcome is symptom severity, measured by the Yung Asthma Severity Score, after 1 h of treatment. If the outcomes of all participants in the trial were independent, then a total sample size of 100 participants per group would be needed to detect a difference in the outcome of 0.92 points with 90% power, based on an independent samples *t*‐test with an assumed standard deviation of 2 points and a two‐sided alpha of 0.05. If the trial uses a re‐randomization design, patients who present to hospital with multiple episodes of asthma exacerbation during the study period are eligible to participate in the trial at each presentation, and will be randomized independently at each enrolment. It is assumed that 50% of patients (clusters) will participate in the study once, 20% twice, 20% three times, 5% four times, and 5% five times, meaning δ = (0.50, 0.20, 0.20, 0.05, 0.05). Using Equation (7), the proportion of observations at each cluster size is therefore γ = (0.26, 0.20, 0.31, 0.10, 0.13). An ICC of 0.5 is assumed for the clustered outcomes. For a continuous outcome and individual randomization, the DEFF for the independence GEE is 1, meaning that 100 enrolments per group are also needed for a re‐randomization design when the analysis will use this working correlation structure, however this could be achieved by recruiting 104 unique patients in total (via Equation (8)) rather than 200. If the exchangeable working correlation structure is used then the DEFF is 0.73, and the target number of enrolments is 73 per group (146 in total), which could be achieved across just 75 unique patients in total.

## 5 Discussion

In this article we have derived DEFFs for calculating the target sample size of two‐arm partially clustered trials with pre‐existing clusters when marginal treatment effects will be estimated via the GEE approach. The DEFFs can be applied to a wide range of partially clustered designs by accommodating the different ways of randomizing the clustered observations and the varying distributions of cluster sizes that occur in practice. The information required to calculate the DEFFs, and hence the target sample size, can all reasonably be estimated when planning a trial, and the DEFF formulas are computationally straightforward. By expressing our DEFFs in terms of the proportions of observations at each cluster size, we have presented exact methods that can be practically used in many partially clustered settings, where the range of cluster sizes is usually relatively small. We have also validated the DEFFs through simulation for a range of partially clustered trial settings. In most cases, observed DEFFs and power were close to expected values, particularly at larger sample sizes, though the DEFFs were typically slightly lower, and the observed power slightly higher, than expected. At smaller sample sizes, the individual randomization DEFFs could be less accurate when the ICC was high. While differences between estimated and observed DEFFs were not extreme, they may be meaningful in trials where an accurate estimate of the required sample size is critical, and simulation‐based methods could be used to determine the sample size instead of our GEE‐based DEFFs in those cases. Importantly, the observed power was typically similar to, or higher than, the expected power, suggesting that the DEFFs can be used to provide a conservative estimate of the required sample size and should not result in an underpowered trial.

The DEFFs that we have derived simplify to those presented in Yelland et al. for the case of independent and paired data (i.e., a maximum cluster size of 2) [13]. They demonstrated through simulation that the DEFFs were valid for a range of settings applicable to neonatal trials that include twins, including a lower proportion of clustered observations than we examined here. Consistent with our findings, the DEFFs were accurate when cluster randomization was used. When individual randomization was used, they found that the exchangeable GEE underestimated the DEFF, and hence overestimated the observed power by up to 30%, when the proportion of pairs was very low (0.015) and the ICC was high (0.8). This coincides with settings where a GEE with an exchangeable working correlation structure has been shown to exhibit poor performance in the analysis of partially clustered trials with paired clusters, with coverage, type I error, and power not achieving target levels when the ICC is high and individual or balanced randomization of pairs is used [14]. The performance of the exchangeable GEE in the analysis of partially clustered trials with clusters larger than size 2 is an area that warrants further research and would help inform the choice of working correlation structure when performing sample size calculations using our DEFFs.

The re‐randomization design [2] is an example of a partially clustered trial design that uses individual randomization of pre‐existing clusters (individuals who participate in the trial multiple times), and our DEFFs can be used to calculate the required sample size for such trials under certain assumptions. In particular, we have assumed that observations within clusters are exchangeable. In a re‐randomization design, this means assuming that there is no impact of treatment history on future outcomes, that is, treatments received during one enrolment do not affect the outcome or treatment effect in future enrolments. We have also assumed that cluster size is not informative, such that the outcome or treatment effect does not vary with the number of enrolments per participant. Taking these assumptions together, this means that our DEFFs apply to re‐randomization trials where the so‐called policy‐benefit and added‐benefit estimands, and the per‐episode and per‐patient estimands, are equivalent [36]. In the terminology of Kahan et al., we have considered both an “unadjusted” (GEE with an independence working correlation structure) and “adjusted” (GEE with an exchangeable working correlation structure) analysis approach [2]. Consistent with their work, we have shown that the unadjusted analysis has a DEFF of 1 for continuous outcomes. Unlike Kahan et al., our independence DEFF for binary outcomes is very close to, but not necessarily exactly equal to 1, due to our use of the robust sandwich variance estimator for the GEE, rather than the naive variance estimator they used. We have extended the theoretical results that were shown in Kahan et al. for an adjusted analysis with clusters of size 2, and our DEFFs can be used to quantify the potential increase in power that would result from using an exchangeable working correlation structure compared to an independence correlation structure, as we have illustrated in Example 2.

Using our DEFFs requires choosing an appropriate working correlation structure. DEFFs based on an exchangeable correlation structure are generally smaller than those that assume an independence correlation structure, and will therefore result in a smaller target sample size. This reduction in the required sample size can be substantial and result in both marked cost savings and reduced time required to conduct the trial. However, there are several reasons to prefer the use of the independence working correlation structure in the analysis in many settings, suggesting the corresponding independence‐based DEFF should be used for calculating the target sample size to ensure that the desired power is achieved. First, a GEE with an exchangeable working correlation structure can perform poorly in the analysis of partially clustered trials when individual level randomization is used, particularly when the sample size is small or the ICC is high [14]. To avoid under‐coverage and inflated type I error rates in these settings, such trials may be analyzed with a GEE with an independence working correlation structure. Second, GEEs with an exchangeable working correlation structure can produce biased estimates of the treatment effect where there is informative cluster size [22]. When the cluster size may be related to the outcome or the treatment effect, then the GEE with an independence working correlation structure is recommended for analysis to ensure an unbiased estimate of the marginal treatment effect [37, 38].

Sample size methods for trials with varying cluster sizes have been developed for cluster randomized trials [39, 40, 41], and our DEFFs can be shown to be algebraically consistent with various DEFFs that are available for that setting. For example, previously published DEFFs for continuous and binary cluster‐level summaries with weighting by cluster size are equivalent to our DEFFs for the independence GEE [42]. Our DEFFs are also equivalent to the GEE‐based DEFFs developed by Pan et al. for binary outcomes with a logit link [43], and DEFFs for continuous outcomes based on an exchangeable GEE or mixed effects model [39]. The exact DEFF for cluster randomized trials requires knowledge of the size of each cluster, and simplifications that use the average cluster size or the coefficient of variation of the cluster sizes are often used in practice [40]. However, in cases where the proportions of clusters with each cluster size are known, our formulas can be used to calculate the exact DEFF for cluster randomized trials when clusters vary in size. Our work complements existing sample size methods for partially clustered trials with post‐randomization clustering (e.g., individually randomized group treatment designs, and designs that include facilitator effects), where clustering is typically present in one arm only [8, 10, 12, 44, 45].

While GEEs are commonly used for analysis of partially clustered trials, mixed effects models are also sometimes used [46]. In the fully clustered setting, estimates obtained via restricted maximum likelihood estimation of parameters in mixed effects models and GEE‐based estimation of marginal model parameters are asymptotically equivalent for continuous outcomes and an identity link, assuming that the working correlation structure is correct [47, 48]. We therefore hypothesize that the DEFF for the exchangeable GEE for a continuous outcome will also give valid estimates of the DEFF if the analysis is to be conducted with a mixed effects model. However, small differences in power have been reported between the mixed model and exchangeable GEE for partially clustered trials with a maximum cluster of size 2 in some settings [14], and the exchangeable correlation structure may not be correct for larger cluster sizes. Further investigation is needed to determine how applicable our DEFFs are for trials that will be analyzed using mixed effects models.

An estimate of the ICC for the outcome of interest is needed in order to use our DEFFs to calculate the required sample size for a trial. While repositories of published ICCs are becoming more common for cluster randomized trials and longitudinal clustered designs [49, 50, 51, 52], fewer estimates are available for settings where partially clustered trials are used. One study summarized ICCs for a range of outcomes measured on twins in neonatal trials [29], and in the absence of more specific data, we suggest using the estimates for twins for trials involving higher order multiples. We are unaware of repositories of ICCs in other partially clustered settings, though values for specific outcomes have been sporadically reported [53, 54]. Reporting the estimated ICC is encouraged in CONSORT reporting guidelines for cluster randomized trials [55], and while recommendations have been developed for describing partially clustered designs [1], no general guidelines for reporting the results of such trials currently exist. Guidelines have recently been developed specifically for studies in plastic surgery that include unilateral and bilateral procedures [56]. While these guidelines include important issues for the reporting of partially clustered trials more generally (e.g., specifying both the number of observations and clusters, and choosing an appropriate statistical model to account for the clustering), the guidelines do not include reporting the ICC. The development of reporting guidelines for partially clustered trials that can be used across the range of fields where these trials are used is warranted, and should encourage the reporting of ICCs with confidence intervals.

The key strength of our work is that we provide, for the first time, sample size methods that can be used to design partially clustered trials with pre‐existing clusters of varying size. Our study also has several limitations. First, we have only considered settings where the cluster size does not affect the treatment effect or the outcome (i.e., non‐informative cluster sizes). While this is often a reasonable assumption, informative cluster size can occur in many settings including neonatal trials with multiple births [38] and re‐randomization designs [36]. Second, we have restricted attention to trials that use simple 1:1 randomization. Although the DEFFs have been derived under the simplifying assumption of treatment balance within cluster size, we have not considered cluster size as a stratification variable in the analysis model. Stratified randomization is often employed to ensure balance in key prognostic factors, and analyses must adjust for the stratification variables to ensure that the variance of the treatment effect is unbiased [57]. DEFFs for partially clustered trials that use stratified randomization by cluster size where cluster size is informative would be a useful extension of the current work. Third, we have assumed that the true correlation structure is exchangeable. More complex correlation structures, such as time‐decaying structures for re‐randomization designs, and allowing for different ICCs by cluster size and/or treatment group, are also relevant in some settings and warrant further investigation. Finally, we have validated the DEFFs for two cluster size distributions with a maximum cluster size of 4, and performance in other settings may vary.

## 6 Conclusion

The effect of clustering should be taken into account during the design of partially clustered trials. The DEFFs provided in this article can be used to determine the target sample size for partially clustered trials with pre‐existing clusters of varying sizes. R functions to calculate the DEFFs are available at https://github.com/klange01/partial‐clustering‐deffs.

## Conflicts of Interest

The authors declare no conflicts of interest.

## Supporting information


**File S1.** Derivation of design effects.

**File S2.** Extra results.

**File S3.** Sensitivity analyses.

**Data S1.** R code.

## Data Availability

R code to reproduce the figures and simulation study in this paper is available at https://github.com/klange01/partial‐clustering‐deffs.
