# Supplementary material for: Sample Size Calculations for Partially Clustered Trials
Source: Stat Med. 2025 Jul 15;44(15-17):e70172. doi: 10.1002/sim.70172 (PMC12261973; doi:10.1002/sim.70172)
Supplement: Supplementary file 1 — File S1. Derivation of design effects. [file SIM-44-0-s002.docx]

**Supplementary File 1 – Derivation of design effects**

**Title:** Sample size calculations for partially clustered trials

**Authors:** Kylie M. Lange, Jessica Kasza, Thomas R. Sullivan, Lisa N. Yelland

Table of Contents:

[1 NOTATION AND ASSUMPTIONS 1](#_Toc174034653)

[2 DESIGN EFFECTS FOR A CONTINUOUS OUTCOME 4](#_Toc174034654)

[3 DESIGN EFFECTS FOR A BINARY OUTCOME WITH A LOGIT LINK 14](#_Toc174034655)

[4 DESIGN EFFECTS FOR A BINARY OUTCOME WITH A LOG LINK 39](#_Toc174034656)

[5 PROOFS OF COMBINATORIAL PROPERTIES 63](#_Toc174034657)

[6 REFERENCES 65](#_Toc174034658)

1. NOTATION AND ASSUMPTIONS

Let $Y_{ij}$ be a continuous or binary outcome measured on the $j$th member of the $i$th cluster ($i=1,\ldots M; j=1,\ldots n_{i}; \sum_{i=1}^{M} n_{i}=N$) and $X_{ij}$ be a binary indicator for the randomised treatment group (1=intervention, 0=control). Clusters are of sizes $k=1, \ldots, K$. Let $M_{k}$ be the number of clusters of size $k$. We assume $M_{1}>0$ and $M_{K}>0$ for some $K>1$, while allowing for the possibility that $M_{k}=0$ for some $k$ such that $1<k<K$. The number of cluster members (observations) in clusters of size $k$ is denoted $N_{k}=kM_{k}$, and the total number of cluster members is $N=\sum_{k=1}^{K} N_{k}$. The proportion of cluster members that belong to a cluster of size $k$ is denoted $\gamma_{k}=kM_{k}/N$ , such that $\sum_{k=1}^{K} \gamma_{k}=1$ and we define $\boldsymbol{\gamma}=(\gamma_{1}, \ldots, \gamma_{K})$. Each cluster member may belong to the intervention group (I) or the control group (C). Within cluster $i$, we denote the number of cluster members that are allocated to the intervention and control groups as $t_{i}$ and $c_{i}$respectively, such that the total number of cluster members in cluster $i$ is $n_{i}=t_{i}+c_{i}$. The total numbers of cluster members in the intervention and control groups are denoted $N_{I}$ and $N_{C}$ respectively, such that $N_{I}=\sum_{i=1}^{M} t_{i}=\sum_{i=1}^{M} \sum_{j=1}^{n_{i}} X_{ij}$ and $N_{C}=\sum_{i=1}^{M} c_{i}=\sum_{i=1}^{M} \sum_{j=1}^{n_{i}} {(1-X}_{ij})$ respectively, and $N_{I}+N_{C}=N$. The numbers of cluster members in the intervention and control groups within each cluster size are denoted $N_{Ik}=\sum_{i=1|n_{i}=k}^{M_{k}} \sum_{j=1}^{k} X_{ij}$ and $N_{Ck}=\sum_{i=1|n_{i}=k}^{M_{k}} \sum_{j=1}^{k} {(1-X}_{ij})$ respectively, such that $N_{Ik}+N_{Ck}=N_{k}$ for$k=1, \ldots, K$. Note that we use the notation $\sum_{i=1|n_{i}=k}^{M_{k}}$to indicate the sum over $i=1,\ldots M$ conditional on $n_{i}=k$, and hence $\sum_{i=1|n_{i}=k}^{M_{k}} \sum_{j=1}^{k}$indicates the sum over all cluster members within clusters of size $k$.

**Assumptions**

To simplify the equations below, the following assumptions are made.

**Overall treatment balance:** The total number of cluster members is the same in each treatment group, i.e. $N_{I}=N_{C}=N/2$.

**Treatment balance within cluster size:** For each cluster size, the number of cluster members is the same in each treatment group, i.e. $N_{Ik}=N_{Ck}={N_{k}}/2$, for $k=1, \ldots, K.$ Note this also produces balanced treatment groups overall.

- **Cluster randomisation**: For cluster randomisation, the assumption of treatment balance within cluster size implies that for each cluster size, the number of clusters in the intervention group, denoted $M_{Ik}$, equals the number of clusters in the control group, denoted $M_{Ck}$. Therefore $N_{Ik}=N_{Ck}=\sum_{i=1|n_{i}=k}^{M} \sum_{j=1}^{k} X_{ij}=\sum_{i=1|n_{i}=k}^{M} \sum_{j=1}^{k} {(1-X}_{ij})$.
- **Individual randomisation**: For individual randomisation, the assumption of treatment balance within cluster size is met by assuming that clusters of each size consist of the **expected proportion of clusters with each pattern of individual treatment allocation within a cluster**. For clusters of size $k$ there are $k+1$ unique patterns of individual treatment allocation corresponding to clusters with $d=0$ to $k$ cluster members in the intervention group (and therefore $k-d$ cluster members in the control group). We assume that observations on the cluster members are exchangeable within clusters, such that there is no ordering of cluster members, and all clusters of the same size with the same number of cluster members in the intervention group are considered to have the same pattern of individual treatment allocation (i.e. a cluster of size 2 where the first cluster member is assigned to the intervention group and the second member to control, has the same pattern of individual treatment allocation as a cluster where the first cluster member is assigned to control and the second member to the intervention group). The number of clusters of size $k$ with $d$ cluster members assigned to the intervention group is denoted $M_{kd}$ and each pattern of individual treatment allocation for a given cluster size $k$ is assumed to occur with proportion ${\binom{k}{d}}/{2^{k}}$. I.e., $N_{Ik}=\sum_{d=0}^{k} dM_{kd}{\binom{k}{d}}/{2^{k}}$, $N_{kC}=\sum_{d=0}^{k} (k-d)M_{kd}{\binom{k}{d}}/{2^{k}}$, and $N_{Ik}=N_{Ck}$.

In the following sections, the derivations are presented as follows: (1) Establish the required terms involved in the covariance formula for a cluster of size $k$; (2) Sum over all clusters so can derive the variance of $\hat{\beta}_{1}$ and hence the DEFF for the general case; (3) Apply the above assumptions to derive the DEFF specifically for cluster and individual randomisation.

# DESIGN EFFECTS FOR A CONTINUOUS OUTCOME

The variance of the treatment effect estimate from a linear regression model for independent data is ${\mathrm{var}\left( \hat{\beta}_{1} \right)}_{IND}=\frac{\sigma^{2}}{N_{I}}+\frac{\sigma^{2}}{N_{C}}$ , which reduces to $\frac{4\sigma^{2}}{N}$ under the assumption of overall treatment balance. This will be used as the denominator in the DEFF (Equation 3 in the main paper).

- 1. **Independence working correlation structure**

$$\mathrm{cov} \left( \hat{\boldsymbol{\beta}} \right)=\left[ \sum_{i=1}^{M} {\mathbf{X}_{i}}^{T}{\mathbf{V}_{i}}^{-1}\mathbf{X}_{i} \right]^{-1}\left[ \sum_{i=1}^{M} {\mathbf{X}_{i}}^{T}{\mathbf{V}_{i}}^{-1}\mathrm{cov} \left( \mathbf{Y}_{i} \right){\mathbf{V}_{i}}^{-1}\mathbf{X}_{i} \right]\left[ \sum_{i=1}^{M} {\mathbf{X}_{i}}^{T}{\mathbf{V}_{i}}^{-1}\mathbf{X}_{i} \right]^{-1}$$

For the independent observations (i.e. $n_{i}=1$ ($j$ subscript dropped for convenience), $X_{i}=0 \left( \mathrm{control} \right) \mathrm{or} 1\left( \mathrm{intervention} \right))$:

$\mathbf{R}_{i}=1$, $\mathbf{A}_{i}^{1/2}=\sigma\Rightarrow$ $\mathbf{V}_{i}= \mathbf{A}_{i}^{1/2}{\mathbf{R}_{i}\mathbf{A}}_{i}^{1/2}=\sigma^{2}\Rightarrow\mathbf{V}_{i}^{-1}=1/\sigma^{2}$

$$\mathbf{X}_{i}^{T}\mathbf{V}_{i}^{-1}\mathbf{X}_{i}= \left[ \begin{matrix} 1 \\ X_{i} \end{matrix} \right]\frac{1}{\sigma^{2}}\left[ \begin{matrix} 1 & X_{i} \end{matrix} \right] = \frac{1}{\sigma^{2}}\left[ \begin{matrix} 1 & X_{i} \\ X_{i} & X_{i}^{2} \end{matrix} \right] = \frac{1}{\sigma^{2}}\left[ \begin{matrix} n_{i} & t_{i} \\ t_{i} & t_{i} \end{matrix} \right]$$

$$\mathbf{X}_{i}^{T}\mathbf{V}_{i}^{-1}{\mathrm{cov}\left( \mathbf{Y}_{i} \right)\mathbf{V}_{i}^{-1}\mathbf{X}}_{i}= \left[ \begin{matrix} 1 \\ X_{i} \end{matrix} \right]\left( \frac{1}{\sigma^{2}} \right)\sigma^{2}\left( \frac{1}{\sigma^{2}} \right)\left[ \begin{matrix} 1 & X_{i} \end{matrix} \right] = \frac{1}{\sigma^{2}}\left[ \begin{matrix} 1 & X_{i} \\ X_{i} & X_{i}^{2} \end{matrix} \right] = \frac{1}{\sigma^{2}}\left[ \begin{matrix} n_{i} & t_{i} \\ t_{i} & t_{i} \end{matrix} \right]$$

For a cluster of size $n_{i}>1$ with $t_{i}$ members assigned to the treatment group and $c_{i}$ members assigned to the control group:

$$\mathbf{R}_{i}=\mathbf{I}_{n_{i}}$$

$$\mathbf{A}_{i}^{1/2}=\sigma\mathbf{I}_{n_{i}}$$

$$\mathbf{V}_{i}= \mathbf{A}_{i}^{1/2}{\mathbf{R}_{i}\mathbf{A}}_{i}^{1/2}=\sigma^{2}\mathbf{I}_{n_{i}}$$

$$\mathbf{V}_{i}^{-1}=(1/\sigma^{2})\mathbf{I}_{n_{i}}$$

$\mathbf{C}_{i}= \left[ \begin{matrix} 1 & \rho& \cdots& \rho\\ \rho& 1 & \ldots& \rho\\ \vdots& \vdots& \ddots& \vdots\\ \rho& \rho& \cdots& 1 \end{matrix} \right]$ , a $n_{i}\times n_{i}$ matrix

$$\mathrm{cov}\left( \mathbf{Y}_{i} \right)=\mathbf{A}_{i}^{1/2}\mathbf{C}_{i}\mathbf{A}_{i}^{1/2}=\sigma^{2} \left[ \begin{matrix} 1 & \rho& \cdots& \rho\\ \rho& 1 & \ldots& \rho\\ \vdots& \vdots& \ddots& \vdots\\ \rho& \rho& \cdots& 1 \end{matrix} \right]$$

$$\mathbf{X}_{i}^{T}\mathbf{V}_{i}^{-1}\mathbf{X}_{i}= \left[ \begin{matrix} 1 & \ldots& 1 \\ X_{i1} & \ldots& X_{{in}_{i}} \end{matrix} \right]\frac{1}{\sigma^{2}}\mathbf{I}_{n_{i}}\left[ \begin{matrix} 1 & X_{i1} \\ \vdots& \vdots\\ 1 & X_{{in}_{i}} \end{matrix} \right] = \frac{1}{\sigma^{2}}\left[ \begin{matrix} n_{i} & \sum_{j=1}^{n_{i}} X_{ij} \\ \sum_{j=1}^{n_{i}} X_{ij} & \sum_{j=1}^{n_{i}} X_{ij}^{2} \end{matrix} \right] = \frac{1}{\sigma^{2}}\left[ \begin{matrix} n_{i} & t_{i} \\ t_{i} & t_{i} \end{matrix} \right]$$

$$\mathbf{X}_{i}^{T}\mathbf{V}_{i}^{-1}{\mathrm{cov}\left( \mathbf{Y}_{i} \right)\mathbf{V}_{i}^{-1}\mathbf{X}}_{i}= \left[ \begin{matrix} 1 & \ldots& 1 \\ X_{i1} & \ldots& X_{in_{i}} \end{matrix} \right]\left( \frac{1}{\sigma^{2}} \right)\mathbf{I}_{n_{i}}\sigma^{2}\left[ \begin{matrix} 1 & \rho& \cdots& \rho\\ \rho& 1 & \ldots& \rho\\ \vdots& \vdots& \ddots& \vdots\\ \rho& \rho& \cdots& 1 \end{matrix} \right]\left( \frac{1}{\sigma^{2}} \right)\mathbf{I}_{n_{i}}\left[ \begin{matrix} 1 & X_{i1} \\ \vdots& \vdots\\ 1 & X_{in_{i}} \end{matrix} \right]$$

$$= \frac{1}{\sigma^{2}}\left[ \begin{matrix} 1+\left( n_{i}-1 \right)\rho& \ldots& 1+\left( n_{i}-1 \right)\rho\\ X_{i1}+\rho\sum_{j\neq1} X_{ij} & \ldots& X_{in_{i}}+\rho\sum_{j\neq n_{i}} X_{ij} \end{matrix} \right]\left[ \begin{matrix} 1 & X_{i1} \\ \vdots& \vdots\\ 1 & X_{in_{i}} \end{matrix} \right]$$

$$= \frac{1}{\sigma^{2}}\left[ \begin{matrix} n_{i}\left( 1+\left( n_{i}-1 \right)\rho\right) & t_{i}\left( 1+\left( n_{i}-1 \right)\rho\right) \\ t_{i}\left( 1+\left( n_{i}-1 \right)\rho\right) & t_{i}+2\rho\left( {t_{i}\left( t_{i}-1 \right)}/2 \right) \end{matrix} \right]$$

$$= \frac{1}{\sigma^{2}}\left[ \begin{matrix} n_{i}(1+\left( n_{i}-1 \right)\rho) & t_{i}(1+\left( n_{i}-1 \right)\rho) \\ t_{i}(1+\left( n_{i}-1 \right)\rho) & t_{i}(1+\left( t_{i}-1 \right)\rho) \end{matrix} \right]$$

Note that when $n_{i}=1$ (and so $t_{i}$ equals either 0 or 1), this reduces to $\frac{1}{\sigma^{2}}\left[ \begin{matrix} n_{i} & t_{i} \\ t_{i} & t_{i} \end{matrix} \right]$ and therefore this expression for $\mathbf{X}_{i}^{T}\mathbf{V}_{i}^{-1}{\mathrm{cov}\left( \mathbf{Y}_{i} \right)\mathbf{V}_{i}^{-1}\mathbf{X}}_{i}$ holds for all $n_{i}\geq1$.

Sum over all clusters $i=1,\ldots, M$. Cluster $i$ is of size $n_{i}$and contains $t_{i}$ members assigned to treatment and $c_{i}$ members assigned to control.

$$\sum_{i=1}^{M} \mathbf{X}_{i}^{T}\mathbf{V}_{i}^{-1}\mathbf{X}_{i}= \sum_{i=1}^{M} \frac{1}{\sigma^{2}}\left[ \begin{matrix} n_{i} & t_{i} \\ t_{i} & t_{i} \end{matrix} \right] = \frac{1}{\sigma^{2}}\left[ \begin{matrix} \sum_{i=1}^{M} n_{i} & \sum_{i=1}^{M} t_{i} \\ \sum_{i=1}^{M} t_{i} & \sum_{i=1}^{M} t_{i} \end{matrix} \right] = \frac{1}{\sigma^{2}}\left[ \begin{matrix} N & N_{I} \\ N_{I} & N_{I} \end{matrix} \right] = \frac{N}{2\sigma^{2}}\left[ \begin{matrix} 2 & 1 \\ 1 & 1 \end{matrix} \right]$$

$= \frac{N}{2\sigma^{2}}\left[ \begin{matrix} 2 & 1 \\ 1 & 1 \end{matrix} \right]$, under the assumption of overall treatment balance

$$\left[ \sum_{i=1}^{M} \mathbf{X}_{i}^{T}\mathbf{V}_{i}^{-1}\mathbf{X}_{i} \right]^{-1}= \frac{2\sigma^{2}}{N}\left[ \begin{matrix} 1 & -1 \\ -1 & 2 \end{matrix} \right]$$

$$\sum_{i=1}^{M} \mathbf{X}_{i}^{T}\mathbf{V}_{i}^{-1}{\mathrm{cov}\left( \mathbf{Y}_{i} \right)\mathbf{V}_{i}^{-1}\mathbf{X}}_{i}= \sum_{i=1}^{M} \frac{1}{\sigma^{2}}\left[ \begin{matrix} n_{i}\left( 1+\left( n_{i}-1 \right)\rho\right) & t_{i}\left( 1+\left( n_{i}-1 \right)\rho\right) \\ t_{i}\left( 1+\left( n_{i}-1 \right)\rho\right) & t_{i}\left( 1+\left( t_{i}-1 \right)\rho\right) \end{matrix} \right] = \frac{1}{\sigma^{2}}\left[ \begin{matrix} \sum_{i=1}^{M} {(n}_{i}+\rho n_{i}^{2}-\rho n_{i}) & \sum_{i=1}^{M} {(t}_{i}+{\rho n}_{i}t_{i}-\rho t_{i}) \\ \sum_{i=1}^{M} {(t}_{i}+\rho n_{i}t_{i}-\rho t_{i}) & \sum_{i=1}^{M} {(t}_{i}+\rho t_{i}^{2}-\rho t_{i}) \end{matrix} \right]$$

$$= \frac{1}{{2\sigma}^{2}}\left[ \begin{matrix} 2N+2\rho\sum_{i=1}^{M} n_{i}^{2}-2\rho N & N+2\rho\sum_{i=1}^{M} n_{i}t_{i}-\rho N \\ N+2\rho\sum_{i=1}^{M} n_{i}t_{i}-\rho N & N+2\rho\sum_{i=1}^{M} t_{i}^{2}-\rho N \end{matrix} \right]$$

$$\mathrm{cov}\left( \hat{\boldsymbol{\beta}} \right)= \left[ \sum_{i=1}^{M} \mathbf{X}_{i}^{T}\mathbf{V}_{i}^{-1}\mathbf{X}_{i} \right]^{-1}\sum_{i=1}^{M} \mathbf{X}_{i}^{T}\mathbf{V}_{i}^{-1}{\mathrm{cov}\left( \mathbf{Y}_{i} \right)\mathbf{V}_{i}^{-1}\mathbf{X}}_{i}\left[ \sum_{i=1}^{M} \mathbf{X}_{i}^{T}\mathbf{V}_{i}^{-1}\mathbf{X}_{i} \right]^{-1}$$

$$= \left( \frac{2\sigma^{2}}{N} \right)\left[ \begin{matrix} 1 & -1 \\ -1 & 2 \end{matrix} \right]\left( \frac{1}{{2\sigma}^{2}} \right)\left[ \begin{matrix} 2N+2\rho\sum_{i=1}^{M} n_{i}^{2}-2\rho N & N+2\rho\sum_{i=1}^{M} n_{i}t_{i}-\rho N \\ N+2\rho\sum_{i=1}^{M} n_{i}t_{i}-\rho N & N+2\rho\sum_{i=1}^{M} t_{i}^{2}-\rho N \end{matrix} \right]\left( \frac{2\sigma^{2}}{N} \right)\left[ \begin{matrix} 1 & -1 \\ -1 & 2 \end{matrix} \right]$$

$$= \frac{2\sigma^{2}}{N^{2}}\left[ \begin{matrix} N-\rho N+2\rho\sum_{i=1}^{M} n_{i}c_{i} & 2\rho\sum_{i=1}^{M} t_{i}c_{i} \\ 4\rho\sum_{i=1}^{M} n_{i}t_{i}-2\rho\sum_{i=1}^{M} n_{i}^{2} & N+4\rho\sum_{i=1}^{M} t_{i}^{2}-2\rho\sum_{i=1}^{M} n_{i}t_{i}-\rho N \end{matrix} \right]\left[ \begin{matrix} 1 & -1 \\ -1 & 2 \end{matrix} \right]$$

$$= \frac{2\sigma^{2}}{N^{2}}\left[ \begin{matrix} N-\rho N+2\rho\sum_{i=1}^{M} \left( n_{i}c_{i}-t_{i}c_{i} \right) & -N+\rho N-2\rho\sum_{i=1}^{M} n_{i}c_{i}+4\rho\sum_{i=1}^{M} t_{i}c_{i} \\ -N+\rho N+\rho\sum_{i=1}^{M} \left( 6n_{i}t_{i}-{2n}_{i}^{2}-4t_{i}(n_{i}-c_{i}) \right) & 2N-2\rho N-\rho\sum_{i=1}^{M} \left( 8n_{i}t_{i}-2n_{i}^{2}-8t_{i}\left( n_{i}-c_{i} \right) \right) \end{matrix} \right]$$

$$= \frac{2\sigma^{2}}{N^{2}}\left[ \begin{matrix} N-\rho N+2\rho\sum_{i=1}^{M} c_{i}^{2} & -N+\rho N-2\rho\sum_{i=1}^{M} n_{i}c_{i}+4\rho\sum_{i=1}^{M} t_{i}c_{i} \\ -N+\rho N-2\rho\sum_{i=1}^{M} n_{i}c_{i}+4\rho\sum_{i=1}^{M} t_{i}c_{i} & 2N-2\rho N-8\rho\sum_{i=1}^{M} t_{i}c_{i}+2\rho\sum_{i=1}^{M} n_{i}^{2} \end{matrix} \right]$$

$$\mathrm{var}\left( \hat{\beta}_{1} \right)= \frac{2\sigma^{2}}{N^{2}}\left( 2N-2\rho N-8\rho\sum_{i=1}^{M} t_{i}c_{i}+2\rho\sum_{i=1}^{M} n_{i}^{2} \right) = \frac{4\sigma^{2}}{N^{2}}\left( N-\rho N-4\rho\sum_{i=1}^{M} t_{i}c_{i}+\rho\sum_{i=1}^{M} n_{i}^{2} \right)$$

$$\Rightarrow DEFF=\left( \frac{4\sigma^{2}}{N^{2}}\left( N-\rho N-4\rho\sum_{i=1}^{M} t_{i}c_{i}+\rho\sum_{i=1}^{M} n_{i}^{2} \right) \right)/\left( {4\sigma^{2}}/N \right)=\frac{1}{N}\left( N-\rho N-4\rho\sum_{i=1}^{M} t_{i}c_{i}+\rho\sum_{i=1}^{M} n_{i}^{2} \right)$$

$$=1+\rho\left( \frac{\sum_{i=1}^{M} n_{i}^{2}}{N}-4\frac{\sum_{i=1}^{M} t_{i}c_{i}}{N}-1 \right)$$

- - 1. Cluster randomisation

$$DEFF= 1+\rho\left( \frac{\sum_{i=1}^{M} n_{i}^{2}}{N}-4\frac{\sum_{i=1}^{M} t_{i}c_{i}}{N}-1 \right) = 1+\rho\left( \frac{\left. \sum_{k=1}^{K} M_{k}k^{2} \right.}{N}-1 \right) = 1+\rho\left( \sum_{k=1}^{K} k\gamma_{k}-1 \right) = 1+\rho\sum_{k=1}^{K} (k-1)\gamma_{k}$$

- - 1. Individual randomisation

$$DEFF= 1+\rho\left( \frac{\sum_{i=1}^{M} n_{i}^{2}}{N}-4\frac{\sum_{i=1}^{M} t_{i}c_{i}}{N}-1 \right)$$

$\frac{\sum_{i=1}^{M} n_{i}^{2}}{N}= \sum_{k=1}^{K} k\gamma_{k}$ as for cluster randomisation above.

Applying property P8 (see Section 5):

$$4\frac{\sum_{i=1}^{M} t_{i}c_{i}}{N} = 4\sum_{k=1}^{K} \sum_{d=0}^{k} \frac{d(k-d)}{N}\left( \frac{\binom{k}{d}}{2^{k}} \right)M_{k} = 4\sum_{k=1}^{K} \frac{M_{k}}{2^{k}N} \sum_{d=0}^{k} d\left( k-d \right)\binom{k}{d} = 4\sum_{k=1}^{K} \frac{M_{k}}{2^{k}N} 2^{k-2}\left( k-1 \right)k = \sum_{k=1}^{K} \frac{{kM}_{k}}{N} \left( k-1 \right) = \sum_{k=1}^{K} \left( k-1 \right) \gamma_{k}$$

$$DEFF= 1+\rho\left( \frac{\sum_{i=1}^{M} n_{i}^{2}}{N}-4\frac{\sum_{i=1}^{M} t_{i}c_{i}}{N}-1 \right) = 1+\rho\left( \sum_{k=1}^{K} k\gamma_{k}+\sum_{k=1}^{K} \left( k-1 \right)\gamma_{k}-1 \right) = 1+\rho\left( \sum_{k=1}^{K} \gamma_{k}-1 \right)=1$$

- 1. **Exchangeable working correlation structure**

$$\mathrm{cov} \left( \hat{\boldsymbol{\beta}} \right)=\left[ \sum_{i=1}^{M} {\mathbf{X}_{i}}^{T}{\mathbf{V}_{i}}^{-1}\mathbf{X}_{i} \right]^{-1}$$

For the independent observations (i.e. $n_{i}=1$ ($j$ subscript dropped for convenience), $X_{i}=0 \left( \mathrm{control} \right) \mathrm{or} 1\left( \mathrm{intervention} \right))$:

$\mathbf{R}_{i}=1$, $\mathbf{A}_{i}^{1/2}=\sigma\Rightarrow$ $\mathbf{V}_{i}= \mathbf{A}_{i}^{1/2}{\mathbf{R}_{i}\mathbf{A}}_{i}^{1/2}=\sigma^{2}\Rightarrow\mathbf{V}_{i}^{-1}=1/\sigma^{2}$

$$\mathbf{X}_{i}^{T}\mathbf{V}_{i}^{-1}\mathbf{X}_{i} = \left[ \begin{matrix} 1 \\ X_{i} \end{matrix} \right]\frac{1}{\sigma^{2}}\left[ \begin{matrix} 1 & X_{i} \end{matrix} \right] = \frac{1}{\sigma^{2}}\left[ \begin{matrix} 1 & X_{i} \\ X_{i} & X_{i}^{2} \end{matrix} \right] = \frac{1}{\sigma^{2}}\left[ \begin{matrix} n_{i} & t_{i} \\ t_{i} & t_{i} \end{matrix} \right]$$

For a cluster of size $n_{i}>1$with $t_{i}$ members assigned to the treatment group and $c_{i}$ members assigned to the control group:

$$\mathbf{R}_{i}=\mathbf{C}_{i}= \left[ \begin{matrix} 1 & \rho& \cdots& \rho\\ \rho& 1 & \ldots& \rho\\ \vdots& \vdots& \ddots& \vdots\\ \rho& \rho& \cdots& 1 \end{matrix} \right], a n_{i}\times n_{i}\mathrm{matrix}$$

$$\mathbf{A}_{i}^{1/2}=\sigma\mathbf{I}_{n_{i}}$$

$$\mathbf{V}_{i}= \mathbf{A}_{i}^{1/2}{\mathbf{R}_{i}\mathbf{A}}_{i}^{1/2} = \sigma\mathbf{I}_{n_{i}}\left[ \begin{matrix} 1 & \rho& \cdots& \rho\\ \rho& 1 & \ldots& \rho\\ \vdots& \vdots& \ddots& \vdots\\ \rho& \rho& \cdots& 1 \end{matrix} \right]\sigma\mathbf{I}_{n_{i}} = \sigma^{2}\left[ \begin{matrix} 1 & \rho& \cdots& \rho\\ \rho& 1 & \ldots& \rho\\ \vdots& \vdots& \ddots& \vdots\\ \rho& \rho& \cdots& 1 \end{matrix} \right]$$

Using the McDaniel et al (2013) expression for the inverse of an exchangeable matrix, $\mathbf{V}_{i}^{-1}$ can be written as:

$\mathbf{V}_{i}^{-1}=(1/\sigma^{2})(\left( a-b \right)\mathbf{I}_{n_{i}}+b\mathbf{J}_{n_{i}}),$ where $a=\frac{1+\left( n_{i}-2 \right)\rho}{(1-\rho)(1+\left( n_{i}-1 \right)\rho)}$, $b=\frac{-\rho}{(1-\rho)(1+\left( n_{i}-1 \right)\rho)}$, $\mathbf{I}_{n_{i}}$ is the $n_{i}\times n_{i}$ identity matrix, and $\mathbf{J}_{n_{i}}$is the $n_{i}\times n_{i}$ matrix of 1s

$$\mathbf{V}_{i}^{-1}=\frac{1}{\sigma^{2}}\left( \left( \frac{1+\left( n_{i}-2 \right)\rho}{\left( 1-\rho\right)\left( 1+\left( n_{i}-1 \right)\rho\right)}+\frac{\rho}{\left( 1-\rho\right)\left( 1+\left( n_{i}-1 \right)\rho\right)} \right)\mathbf{I}_{n_{i}}- \frac{\rho}{(1-\rho)(1+\left( n_{i}-1 \right)\rho}\mathbf{J}_{n_{i}} \right)$$

$$= \frac{1}{\sigma^{2}\left( 1-\rho\right)\left( 1+\left( n_{i}-1 \right)\rho\right)}\left( (1+\left( n_{i}-1 \right)\rho)\left[ \begin{matrix} 1 & 0 & \cdots& 0 \\ 0 & 1 & \ldots& 0 \\ \vdots& \vdots& \ddots& \vdots\\ 0 & 0 & \cdots& 1 \end{matrix} \right]-\left[ \begin{matrix} \rho& \rho& \cdots& \rho\\ \rho& \rho& \ldots& \rho\\ \vdots& \vdots& \ddots& \vdots\\ \rho& \rho& \cdots& \rho\end{matrix} \right] \right)$$

$$= \frac{1}{\sigma^{2}\left( 1-\rho\right)\left( 1+\left( n_{i}-1 \right)\rho\right)}\left[ \begin{matrix} 1+\left( n_{i}-2 \right)\rho& -\rho& \cdots& -\rho\\ -\rho& 1+\left( n_{i}-2 \right)\rho& \ldots& -\rho\\ \vdots& \vdots& \ddots& \vdots\\ -\rho& -\rho& \cdots& 1+\left( n_{i}-2 \right)\rho\end{matrix} \right]$$

$$\mathbf{X}_{i}^{T}\mathbf{V}_{i}^{-1}\mathbf{X}_{i}= \left[ \begin{matrix} 1 & \ldots& 1 \\ X_{1} & \ldots& X_{n_{i}} \end{matrix} \right]\left( \frac{1}{\sigma^{2}\left( 1-\rho\right)\left( 1+\left( n_{i}-1 \right)\rho\right)} \right)\left[ \begin{matrix} 1+\left( n_{i}-2 \right)\rho& -\rho& \cdots& -\rho\\ -\rho& 1+\left( n_{i}-2 \right)\rho& \ldots& -\rho\\ \vdots& \vdots& \ddots& \vdots\\ -\rho& -\rho& \cdots& 1+\left( n_{i}-2 \right)\rho\end{matrix} \right]\left[ \begin{matrix} 1 & X_{1} \\ \vdots& \vdots\\ 1 & X_{n_{i}} \end{matrix} \right]$$

$$= \frac{1}{\sigma^{2}\left( 1-\rho\right)\left( 1+\left( n_{i}-1 \right)\rho\right)}\left[ \begin{matrix} 1-\rho& \ldots& 1-\rho\\ \left( 1+\left( n_{i}-1 \right)\rho\right)X_{1}-\rho\sum_{j=1}^{n_{i}} X_{j} & \ldots& \left( 1+\left( n_{i}-1 \right)\rho\right)X_{n_{i}}-\rho\sum_{j=1}^{n_{i}} X_{j} \end{matrix} \right]\left[ \begin{matrix} 1 & X_{1} \\ \vdots& \vdots\\ 1 & X_{n_{i}} \end{matrix} \right]$$

$$=\frac{1}{\sigma^{2}\left( 1-\rho\right)\left( 1+\left( n_{i}-1 \right)\rho\right)}\left[ \begin{matrix} n_{i}\left( 1-\rho\right) & \left( 1-\rho\right)\sum_{j=1}^{n_{i}} X_{j} \\ \left( 1+\left( n_{i}-1 \right)\rho\right)\sum_{j=1}^{n_{i}} X_{j}-n_{i}\rho\sum_{j=1}^{n_{i}} X_{j} & \left( 1+\left( n_{i}-1 \right)\rho\right)\sum_{j=1}^{n_{i}} X_{j}^{2}-\rho\sum_{j=1}^{n_{i}} X_{j}\sum_{j=1}^{n_{i}} X_{j} \end{matrix} \right]$$

$$=\frac{1}{\sigma^{2}\left( 1-\rho\right)\left( 1+\left( n_{i}-1 \right)\rho\right)}\left[ \begin{matrix} n_{i}\left( 1-\rho\right) & t_{i}\left( 1-\rho\right) \\ t_{i}\left( 1-\rho\right) & \left( 1+\left( n_{i}-2 \right)\rho\right)t_{i}-2\rho\frac{t_{i}\left( t_{i}-1 \right)}{2} \end{matrix} \right]$$

$$=\frac{1}{\sigma^{2}\left( 1-\rho\right)\left( 1+\left( n_{i}-1 \right)\rho\right)}\left[ \begin{matrix} n_{i}\left( 1-\rho\right) & t_{i}\left( 1-\rho\right) \\ t_{i}\left( 1-\rho\right) & t_{i}\left( 1-\rho)+\rho t_{i}c_{i} \right) \end{matrix} \right] = \frac{1-\rho}{\sigma^{2}\left( 1-\rho\right)\left( 1+\left( n_{i}-1 \right)\rho\right)}\left[ \begin{matrix} n_{i} & t_{i} \\ t_{i} & t_{i}+\frac{\rho}{1-\rho}t_{i}c_{i} \end{matrix} \right]$$

Note that when $n_{i}=1$ (and so $t_{i}c_{i}$ equals 0), this reduces to $\frac{1}{\sigma^{2}}\left[ \begin{matrix} n_{i} & t_{i} \\ t_{i} & t_{i} \end{matrix} \right]$ and therefore this expression for $\mathbf{X}_{i}^{T}\mathbf{V}_{i}^{-1}\mathbf{X}_{i}$ holds for all $n_{i}\geq1$.

Sum over all clusters $i=1,\ldots, M$. Cluster $i$ is of size $n_{i}$and contains $t_{i}$ members assigned to treatment and $c_{i}$ members assigned to control.

$$\sum_{i=1}^{M} \mathbf{X}_{i}^{T}\mathbf{V}_{i}^{-1}\mathbf{X}_{i} = \frac{1}{\sigma^{2}}\sum_{i=1}^{M} \left( \frac{1}{\left( 1-\rho\right)\left( 1+\left( n_{i}-1 \right)\rho\right)} \right)\left[ \begin{matrix} n_{i}\left( 1-\rho\right) & t_{i}\left( 1-\rho\right) \\ t_{i}\left( 1-\rho\right) & t_{i}(1+\left( c_{i}-1 \right)\rho) \end{matrix} \right] = \frac{1}{\sigma^{2}}\left[ \begin{matrix} \sum_{i=1}^{M} \frac{n_{i}}{\left( 1+\left( n_{i}-1 \right)\rho\right)} & \sum_{i=1}^{M} \frac{t_{i}}{\left( 1+\left( n_{i}-1 \right)\rho\right)} \\ \sum_{i=1}^{M} \frac{t_{i}}{\left( 1+\left( n_{i}-1 \right)\rho\right)} & \sum_{i=1}^{M} \frac{t_{i}(1+\left( c_{i}-1 \right)\rho)}{\left( 1-\rho\right)\left( 1+\left( n_{i}-1 \right)\rho\right)} \end{matrix} \right]$$

$$\mathrm{cov} \left( \hat{\boldsymbol{\beta}} \right)=\left[ \sum_{i=1}^{M} {\mathbf{X}_{i}}^{T}{\mathbf{V}_{i}}^{-1}\mathbf{X}_{i} \right]^{-1}= \sigma^{2}\left[ \begin{matrix} \sum_{i=1}^{M} \frac{n_{i}}{\left( 1+\left( n_{i}-1 \right)\rho\right)} & \sum_{i=1}^{M} \frac{t_{i}}{\left( 1+\left( n_{i}-1 \right)\rho\right)} \\ \sum_{i=1}^{M} \frac{t_{i}}{\left( 1+\left( n_{i}-1 \right)\rho\right)} & \sum_{i=1}^{M} \frac{t_{i}(1+\left( c_{i}-1 \right)\rho)}{\left( 1-\rho\right)\left( 1+\left( n_{i}-1 \right)\rho\right)} \end{matrix} \right]^{-1}$$

$$= \frac{\sigma^{2}}{\left( \sum_{i=1}^{M} \frac{n_{i}}{\left( 1+\left( n_{i}-1 \right)\rho\right)} \right)\left( \sum_{i=1}^{M} \frac{t_{i}(1+\left( c_{i}-1 \right)\rho)}{\left( 1-\rho\right)\left( 1+\left( n_{i}-1 \right)\rho\right)} \right)-\left( \sum_{i=1}^{M} \frac{t_{i}}{\left( 1+\left( n_{i}-1 \right)\rho\right)} \right)^{2}}\left[ \begin{matrix} \sum_{i=1}^{M} \frac{t_{i}(1+\left( c_{i}-1 \right)\rho)}{\left( 1-\rho\right)\left( 1+\left( n_{i}-1 \right)\rho\right)} & -\sum_{i=1}^{M} \frac{t_{i}}{\left( 1+\left( n_{i}-1 \right)\rho\right)} \\ -\sum_{i=1}^{M} \frac{t_{i}}{\left( 1+\left( n_{i}-1 \right)\rho\right)} & \sum_{i=1}^{M} \frac{n_{i}}{\left( 1+\left( n_{i}-1 \right)\rho\right)} \end{matrix} \right]$$

$$\mathrm{var}\left( \hat{\beta}_{1} \right)= \frac{\sigma^{2}\sum_{i=1}^{M} \frac{n_{i}}{\left( 1+\left( n_{i}-1 \right)\rho\right)}}{\left( \sum_{i=1}^{M} \frac{n_{i}}{\left( 1+\left( n_{i}-1 \right)\rho\right)} \right)\left( \sum_{i=1}^{M} \frac{t_{i}(1+\left( c_{i}-1 \right)\rho)}{\left( 1-\rho\right)\left( 1+\left( n_{i}-1 \right)\rho\right)} \right)-\left( \sum_{i=1}^{M} \frac{t_{i}}{\left( 1+\left( n_{i}-1 \right)\rho\right)} \right)^{2}}$$

$$\Rightarrow DEFF= \left( \frac{N}{4} \right)\times\frac{\sum_{i=1}^{M} \frac{n_{i}}{\left( 1+\left( n_{i}-1 \right)\rho\right)}}{\left( \sum_{i=1}^{M} \frac{n_{i}}{\left( 1+\left( n_{i}-1 \right)\rho\right)} \right)\left( \sum_{i=1}^{M} \frac{t_{i}(1+\left( c_{i}-1 \right)\rho)}{\left( 1-\rho\right)\left( 1+\left( n_{i}-1 \right)\rho\right)} \right)-\left( \sum_{i=1}^{M} \frac{t_{i}}{\left( 1+\left( n_{i}-1 \right)\rho\right)} \right)^{2}}$$

which can be written as:

$$DEFF= \frac{NA}{4\left( AB-C^{2} \right)}$$

where $A=\sum_{i=1}^{M} \frac{n_{i}}{\left( 1+\left( n_{i}-1 \right)\rho\right)}$ , $B=\sum_{i=1}^{M} \frac{t_{i}(1+\left( c_{i}-1 \right)\rho)}{\left( 1-\rho\right)\left( 1+\left( n_{i}-1 \right)\rho\right)}$ , and $C=\sum_{i=1}^{M} \frac{t_{i}}{\left( 1+\left( n_{i}-1 \right)\rho\right)}$

- - 1. Cluster randomisation

$$A=\sum_{i=1}^{M} \frac{n_{i}}{\left( 1+\left( n_{i}-1 \right)\rho\right)} = \sum_{k=1}^{K} M_{k}\left( \frac{k}{1+\left( k-1 \right)\rho} \right)$$

$$B=\sum_{i=1}^{M} \frac{t_{i}(1+\left( c_{i}-1 \right)\rho)}{\left( 1-\rho\right)\left( 1+\left( n_{i}-1 \right)\rho\right)} = \sum_{k=1}^{K} \left[ \frac{M_{k}}{2}\left( \frac{k(1+\left( 0-1 \right)\rho)}{\left( 1-\rho\right)\left( 1+\left( k-1 \right)\rho\right)} \right)+\frac{M_{k}}{2}\left( \frac{0(1+\left( k-1 \right)\rho)}{\left( 1-\rho\right)\left( 1+\left( k-1 \right)\rho\right)} \right) \right] = \frac{1}{2}\sum_{k=1}^{K} M_{k}\left( \frac{k}{1+\left( k-1 \right)\rho} \right) = \frac{1}{2}A$$

$$C=\sum_{i=1}^{M} \frac{t_{i}}{\left( 1+\left( n_{i}-1 \right)\rho\right)} = \sum_{k=1}^{K} \left[ \frac{M_{k}}{2}\left( \frac{k}{1+\left( k-1 \right)\rho} \right)+\frac{M_{k}}{2}\left( \frac{0}{1+\left( k-1 \right)\rho} \right) \right] = \frac{1}{2}\sum_{k=1}^{K} M_{j}\left( \frac{k}{1+\left( k-1 \right)\rho} \right) = B = \frac{1}{2}A$$

$$DEFF= \frac{NA}{4\left( AB-C^{2} \right)} = \frac{NA}{4\left( A\left( \frac{A}{2} \right)-\frac{1}{4}A^{2} \right)} = \frac{NA}{2A^{2}-A^{2}} = \frac{N}{A}$$

$$= \frac{N}{\sum_{k=1}^{K} M_{k}\left( \frac{k}{1+\left( k-1 \right)\rho} \right)} = \frac{1}{\sum_{k=1}^{K} \left( \frac{1}{1+\left( k-1 \right)\rho} \right)\gamma_{k}} = \left[ \sum_{k=1}^{K} \left( \frac{1}{1+\left( k-1 \right)\rho} \right)\gamma_{k} \right]^{-1}$$

- - 1. Individual randomisation

$$A=\sum_{i=1}^{M} \frac{n_{i}}{\left( 1+\left( n_{i}-1 \right)\rho\right)} = \sum_{k=1}^{K} M_{k}\left( \frac{k}{1+\left( k-1 \right)\rho} \right)$$

Applying property P10 (See Section 5):

$$B=\sum_{i=1}^{M} \frac{t_{i}\left( 1+\left( c_{i}-1 \right)\rho\right)}{\left( 1-\rho\right)\left( 1+\left( n_{i}-1 \right)\rho\right)} = \sum_{k=1}^{K} \sum_{d=0}^{k} \frac{d\left( 1+\left( k-d-1 \right)\rho\right)}{\left( 1-\rho\right)\left( 1+\left( k-1 \right)\rho\right)}\left( \frac{\binom{k}{d}}{2^{k}}M_{k} \right) = \sum_{k=1}^{K} \frac{1}{\left( 1-\rho\right)\left( 1+\left( k-1 \right)\rho\right)}\left( \frac{M_{k}}{2^{k}} \right) \sum_{d=0}^{k} \left( d(1+\left( k-d-1 \right)\rho\right)\binom{k}{d}$$

$$=\sum_{k=1}^{K} \frac{1}{\left( 1-\rho\right)\left( 1+\left( k-1 \right)\rho\right)}\left( \frac{M_{k}}{2^{k}} \right)2^{k-2}k\left( \left( k+3 \right)\rho+2 \right) = \frac{1}{4}\sum_{k=1}^{K} \frac{\left( k+3 \right)\rho+2}{\left( 1-\rho\right)\left( 1+\left( k-1 \right)\rho\right)}M_{k}k$$

Applying property P4 (See Section 5):

$$C=\sum_{i=1}^{M} \frac{t_{i}}{\left( 1+\left( n_{i}-1 \right)\rho\right)} = \sum_{k=1}^{K} \sum_{d=0}^{k} \frac{d}{\left( 1+\left( k-1 \right)\rho\right)} = \sum_{k=1}^{K} \frac{1}{\left( 1+\left( k-1 \right)\rho\right)} \sum_{d=0}^{k} d\frac{\binom{k}{d}}{2^{k}}M_{k} = \sum_{k=1}^{K} \frac{1}{\left( 1+\left( k-1 \right)\rho\right)}\left( \frac{M_{k}}{2^{k}} \right) \sum_{d=0}^{k} d\binom{k}{d}$$

$$=\sum_{k=1}^{K} \frac{1}{\left( 1+\left( k-1 \right)\rho\right)}\left( \frac{M_{k}}{2^{k}} \right)2^{k-1}k = \sum_{k=1}^{K} \frac{k}{\left( 1+\left( k-1 \right)\rho\right)}\left( \frac{M_{k}}{2} \right) = \frac{1}{2}A$$

$$DEFF= \frac{NA}{4\left( AB-C^{2} \right)}=\frac{NA}{4\left( AB-\frac{1}{4}A^{2} \right)}= \frac{NA}{4AB-A^{2}}= \frac{N}{4B-A}$$

$$= \frac{N}{4\left( \frac{1}{4}\sum_{k=1}^{K} \frac{\left( k+3 \right)\rho+2}{\left( 1-\rho\right)\left( 1+\left( k-1 \right)\rho\right)}M_{k}k \right)-\sum_{k=1}^{K} M_{k}\left( \frac{k}{1+\left( k-1 \right)\rho} \right)}$$

$$= \frac{1}{\sum_{k=1}^{K} \frac{\left( k+3 \right)\rho+2}{\left( 1-\rho\right)\left( 1+\left( k-1 \right)\rho\right)}\left( \frac{M_{k}k}{N} \right)-\sum_{k=1}^{K} \frac{1}{1+\left( k-1 \right)\rho}\left( \frac{M_{k}k}{N} \right)}$$

$$= \frac{1}{\sum_{k=1}^{K} \frac{\left( k+3 \right)\rho+2-\left( 1-\rho\right)}{\left( 1-\rho\right)\left( 1+\left( k-1 \right)\rho\right)}\gamma_{k}} = \frac{1}{\sum_{k=1}^{K} \frac{1+\left( k-2 \right)\rho}{\left( 1-\rho\right)\left( 1+\left( k-1 \right)\rho\right)}\gamma_{k}} = \left[ \sum_{k=1}^{K} \frac{1+\left( k-2 \right)\rho}{\left( 1-\rho\right)\left( 1+\left( k-1 \right)\rho\right)}\gamma_{k} \right]^{-1}$$

# DESIGN EFFECTS FOR A BINARY OUTCOME WITH A LOGIT LINK

The variance of the treatment effect estimate from a generalised linear model for independent data, using the multivariate delta method, is ${\mathrm{var}\left( \hat{\beta}_{1} \right)}_{IND}=\frac{1}{N_{I}\pi_{I}(1-\pi_{I})}+\frac{1}{N_{C}\pi_{C}(1-\pi_{C})}$ , which equals $\frac{2(\pi_{I}\left( 1-\pi_{I} \right)+\pi_{C}\left( 1-\pi_{C} \right))}{N\pi_{I}\pi_{C}(1-\pi_{I})(1-\pi_{C})}$ under the assumption of overall treatment balance. This will be used as the denominator in the DEFF (Equation 3 in the main paper).

- 1. **Independence working correlation structure**

$$\mathrm{cov} \left( \hat{\boldsymbol{\beta}} \right)=\left[ \sum_{i=1}^{M} {\mathbf{D}_{i}}^{T}{\mathbf{V}_{i}}^{-1}\mathbf{D}_{i} \right]^{-1}\left[ \sum_{i=1}^{M} {\mathbf{D}_{i}}^{T}{\mathbf{V}_{i}}^{-1}\mathrm{cov} \left( \mathbf{Y}_{i} \right){\mathbf{V}_{i}}^{-1}\mathbf{D}_{i} \right]\left[ \sum_{i=1}^{M} {\mathbf{D}_{i}}^{T}{\mathbf{V}_{i}}^{-1}\mathbf{D}_{i} \right]^{-1}$$

For the independent observations (i.e. $n_{i}=1 \left( j subscript dropped for convenience \right), X_{i}=0 \left( \mathrm{control} \right) \mathrm{or} 1\left( \mathrm{intervention} \right))$:

$\mathbf{R}_{i}=1$, $\mathbf{A}_{i}^{1/2}=\sqrt{\mu_{i}(1-\mu_{i})}\Rightarrow$ $\mathbf{V}_{i}= \mathbf{A}_{i}^{1/2}{\mathbf{R}_{i}\mathbf{A}}_{i}^{1/2}=\mu_{i}(1-\mu_{i})\Rightarrow\mathbf{V}_{i}^{-1}=1/\mu_{i}(1-\mu_{i})$

$\mathbf{C}_{i}=1$, $\mathbf{A}_{i}^{1/2}=\sqrt{\mu_{i}(1-\mu_{i})}\Rightarrow\mathrm{cov}\left( \mathbf{Y}_{i} \right)=$ $\mathbf{A}_{i}^{1/2}{\mathbf{C}_{i}\mathbf{A}}_{i}^{1/2}=\mu_{i}(1-\mu_{i})$

$$\frac{\partial\mu_{i}}{\partial\beta_{0}} = \frac{\exp\left( \beta_{0}+\beta_{1}X_{i} \right)\left[ 1+{\exp(\beta}_{0}+\beta_{1}X_{i}) \right]-\left[ \exp\left( \beta_{0}+\beta_{1}X_{i} \right) \right]^{2}}{\left[ 1+{\exp(\beta}_{0}+\beta_{1}X_{i}) \right]^{2}} = \frac{\exp\left( \beta_{0}+\beta_{1}X_{i} \right)}{1+\exp\left( \beta_{0}+\beta_{1}X_{i} \right)}x\frac{1}{1+\exp\left( \beta_{0}+\beta_{1}X_{i} \right)} = \mu_{i}(1-\mu_{i})$$

$$\frac{\partial\mu_{i}}{\partial\beta_{1}} = \frac{X_{i}\exp\left( \beta_{0}+\beta_{1}X_{i} \right)\left[ 1+{\exp(\beta}_{0}+\beta_{1}X_{i}) \right]-X_{i}\left[ \exp\left( \beta_{0}+\beta_{1}X_{i} \right) \right]^{2}}{\left[ 1+{\exp(\beta}_{0}+\beta_{1}X_{i}) \right]^{2}} = {X_{i}\mu}_{i}(1-\mu_{i})$$

$$\Rightarrow\mathbf{D}_{i}=\left[ \begin{matrix} \frac{\partial\mu_{i}}{\partial\beta_{0}} & \frac{\partial\mu_{i}}{\partial\beta_{1}} \end{matrix} \right] = \left[ \begin{matrix} \mu_{i}(1-\mu_{i}) & {X_{i}\mu}_{i}(1-\mu_{i}) \end{matrix} \right]$$

$$\mathbf{D}_{i}^{T}\mathbf{V}_{i}^{-1}\mathbf{D}_{i}= \left[ \begin{matrix} \mu_{i}\left( 1-\mu_{i} \right) \\ {X_{i}\mu}_{i}\left( 1-\mu_{i} \right) \end{matrix} \right]\frac{1}{\mu_{i}\left( 1-\mu_{i} \right)}\left[ \begin{matrix} \mu_{i}\left( 1-\mu_{i} \right) & {X_{i}\mu}_{i}\left( 1-\mu_{i} \right) \end{matrix} \right] = \frac{1}{\mu_{i}\left( 1-\mu_{i} \right)}\left[ \begin{matrix} \left[ \mu_{i}\left( 1-\mu_{i} \right) \right]^{2} & X_{i}\left[ \mu_{i}\left( 1-\mu_{i} \right) \right]^{2} \\ X_{i}\left[ \mu_{i}\left( 1-\mu_{i} \right) \right]^{2} & X_{i}^{2}\left[ \mu_{i}\left( 1-\mu_{i} \right) \right]^{2} \end{matrix} \right]$$

$$=\left[ \begin{matrix} \mu_{i}\left( 1-\mu_{i} \right) & X_{i}\mu_{i}\left( 1-\mu_{i} \right) \\ X_{i}\mu_{i}\left( 1-\mu_{i} \right) & X_{i}^{2}\mu_{i}\left( 1-\mu_{i} \right) \end{matrix} \right]$$

$$\mathbf{D}_{i}^{T}\mathbf{V}_{i}^{-1}{\mathrm{cov}\left( \mathbf{Y}_{i} \right)\mathbf{V}_{i}^{-1}\mathbf{D}}_{i}= \left[ \begin{matrix} \mu_{i}\left( 1-\mu_{i} \right) \\ {X_{i}\mu}_{i}\left( 1-\mu_{i} \right) \end{matrix} \right]\frac{1}{\mu_{i}\left( 1-\mu_{i} \right)}\mu_{i}\left( 1-\mu_{i} \right)\frac{1}{\mu_{i}\left( 1-\mu_{i} \right)}\left[ \begin{matrix} \mu_{i}\left( 1-\mu_{i} \right) & {X_{i}\mu}_{i}\left( 1-\mu_{i} \right) \end{matrix} \right]$$

$$=\frac{1}{\mu_{i}\left( 1-\mu_{i} \right)}\left[ \begin{matrix} \left[ \mu_{i}\left( 1-\mu_{i} \right) \right]^{2} & X_{i}\left[ \mu_{i}\left( 1-\mu_{i} \right) \right]^{2} \\ X_{i}\left[ \mu_{i}\left( 1-\mu_{i} \right) \right]^{2} & X_{i}^{2}\left[ \mu_{i}\left( 1-\mu_{i} \right) \right]^{2} \end{matrix} \right] = \left[ \begin{matrix} \mu_{i}(1-\mu_{i}) & X_{i}\mu_{i}(1-\mu_{i}) \\ X_{i}\mu_{i}(1-\mu_{i}) & X_{i}^{2}\mu_{i}(1-\mu_{i}) \end{matrix} \right]$$

For a cluster of size $n_{i}>1$ with $t_{i}$ members assigned to the treatment group and $c_{i}$ members assigned to the control group:

$$\mathbf{R}_{i}=\mathbf{I}_{n_{i}}$$

$\mathbf{A}_{i}^{1/2}=\left[ \begin{matrix} \sqrt{\mu_{i1}(1-\mu_{i1})} & 0 & \cdots& 0 \\ 0 & \sqrt{\mu_{i2}(1-\mu_{i2})} & \ldots& 0 \\ \vdots& \vdots& \ddots& \vdots\\ 0 & 0 & \cdots& \sqrt{\mu_{{in}_{i}}(1-\mu_{in_{i}})} \end{matrix} \right]$ , a $n_{i}\times n_{i}$ matrix

$$\mathbf{V}_{i}= \mathbf{A}_{i}^{1/2}{\mathbf{R}_{i}\mathbf{A}}_{i}^{1/2}=\left[ \begin{matrix} \sqrt{\mu_{i1}(1-\mu_{i1})} & 0 & \cdots& 0 \\ 0 & \sqrt{\mu_{i2}(1-\mu_{i2})} & \ldots& 0 \\ \vdots& \vdots& \ddots& \vdots\\ 0 & 0 & \cdots& \sqrt{\mu_{in_{i}}(1-\mu_{in_{i}})} \end{matrix} \right]\mathbf{I}_{n_{i}}\left[ \begin{matrix} \sqrt{\mu_{i1}(1-\mu_{i1})} & 0 & \cdots& 0 \\ 0 & \sqrt{\mu_{i2}(1-\mu_{i2})} & \ldots& 0 \\ \vdots& \vdots& \ddots& \vdots\\ 0 & 0 & \cdots& \sqrt{\mu_{{in}_{i}}(1-\mu_{{in}_{i}})} \end{matrix} \right]$$

$$=\left[ \begin{matrix} \mu_{i1}(1-\mu_{i1}) & 0 & \cdots& 0 \\ 0 & \mu_{i2}(1-\mu_{i2}) & \ldots& 0 \\ \vdots& \vdots& \ddots& \vdots\\ 0 & 0 & \cdots& \mu_{in_{i}}(1-\mu_{{in}_{i}}) \end{matrix} \right]$$

$$\mathbf{V}_{i}^{-1}=\left[ \begin{matrix} \left[ \mu_{i1}\left( 1-\mu_{i1} \right) \right]^{-1} & 0 & \cdots& 0 \\ 0 & \left[ \mu_{i2}\left( 1-\mu_{i2} \right) \right]^{-1} & \ldots& 0 \\ \vdots& \vdots& \ddots& \vdots\\ 0 & 0 & \cdots& \left[ \mu_{in_{i}}\left( 1-\mu_{in_{i}} \right) \right]^{-1} \end{matrix} \right]$$

$\mathbf{C}_{i}= \left[ \begin{matrix} 1 & \rho& \cdots& \rho\\ \rho& 1 & \ldots& \rho\\ \vdots& \vdots& \ddots& \vdots\\ \rho& \rho& \cdots& 1 \end{matrix} \right]$ , a $n_{i}\times n_{i}$ matrix

$$\mathrm{cov}\left( \mathbf{Y}_{i} \right)=\mathbf{A}_{i}^{1/2}\mathbf{C}_{i}\mathbf{A}_{i}^{1/2}=$$

$$\left[ \begin{matrix} \sqrt{\mu_{i1}\left( 1-\mu_{i1} \right)} & 0 & \cdots& 0 \\ 0 & \sqrt{\mu_{i2}\left( 1-\mu_{i2} \right)} & \ldots& 0 \\ \vdots& \vdots& \ddots& \vdots\\ 0 & 0 & \cdots& \sqrt{\mu_{{in}_{i}}\left( 1-\mu_{{in}_{i}} \right)} \end{matrix} \right] \left[ \begin{matrix} 1 & \rho& \cdots& \rho\\ \rho& 1 & \ldots& \rho\\ \vdots& \vdots& \ddots& \vdots\\ \rho& \rho& \cdots& 1 \end{matrix} \right]\left[ \begin{matrix} \sqrt{\mu_{i1}\left( 1-\mu_{i1} \right)} & 0 & \cdots& 0 \\ 0 & \sqrt{\mu_{i2}\left( 1-\mu_{i2} \right)} & \ldots& 0 \\ \vdots& \vdots& \ddots& \vdots\\ 0 & 0 & \cdots& \sqrt{\mu_{{in}_{i}}\left( 1-\mu_{{in}_{i}} \right)} \end{matrix} \right]$$

$$=\left[ \begin{matrix} \sqrt{\mu_{i1}\left( 1-\mu_{i1} \right)} & \rho\sqrt{\mu_{i1}\left( 1-\mu_{i1} \right)} & \cdots& \rho\sqrt{\mu_{i1}\left( 1-\mu_{i1} \right)} \\ \rho\sqrt{\mu_{i2}\left( 1-\mu_{i2} \right)} & \sqrt{\mu_{i2}\left( 1-\mu_{i2} \right)} & \ldots& \rho\sqrt{\mu_{i2}\left( 1-\mu_{i2} \right)} \\ \vdots& \vdots& \ddots& \vdots\\ \rho\sqrt{\mu_{in_{i}}\left( 1-\mu_{{in}_{i}} \right)} & \rho\sqrt{\mu_{in_{i}}\left( 1-\mu_{{in}_{i}} \right)} & \cdots& \sqrt{\mu_{{in}_{i}}\left( 1-\mu_{{in}_{i}} \right)} \end{matrix} \right] \left[ \begin{matrix} \sqrt{\mu_{i1}\left( 1-\mu_{i1} \right)} & 0 & \cdots& 0 \\ 0 & \sqrt{\mu_{i2}\left( 1-\mu_{i2} \right)} & \ldots& 0 \\ \vdots& \vdots& \ddots& \vdots\\ 0 & 0 & \cdots& \sqrt{\mu_{{in}_{i}}\left( 1-\mu_{in_{i}} \right)} \end{matrix} \right]$$

$$=\left[ \begin{matrix} \mu_{i1}\left( 1-\mu_{i1} \right) & \rho\sqrt{\mu_{i1}\mu_{i2}\left( 1-\mu_{i1} \right)\left( 1-\mu_{i2} \right)} & \cdots& \rho\sqrt{\mu_{i1}\mu_{{in}_{i}}\left( 1-\mu_{i1} \right)\left( 1-\mu_{in_{i}} \right)} \\ \rho\sqrt{\mu_{i1i}\mu_{i2}\left( 1-\mu_{i1} \right)\left( 1-\mu_{i2} \right)} & \mu_{i2}\left( 1-\mu_{i2} \right) & \ldots& \rho\sqrt{\mu_{i2}\mu_{{in}_{i}}\left( 1-\mu_{i2} \right)\left( 1-\mu_{{in}_{i}} \right)} \\ \vdots& \vdots& \ddots& \vdots\\ \rho\sqrt{\mu_{i1}\mu_{in_{i}}\left( 1-\mu_{i1} \right)\left( 1-\mu_{in_{i}} \right)} & \rho\sqrt{\mu_{i2}\mu_{{in}_{i}}\left( 1-\mu_{i2} \right)\left( 1-\mu_{{in}_{i}} \right)} & \cdots& \mu_{{in}_{i}}\left( 1-\mu_{in_{i}} \right) \end{matrix} \right]$$

$$\mathbf{D}_{i}=\left[ \begin{matrix} \begin{matrix} \frac{\partial\mu_{i1}}{\partial\beta_{0}} \end{matrix} & \frac{\partial\mu_{i1}}{\partial\beta_{1}} \\ \vdots& \vdots\\ \frac{\partial\mu_{in_{i}}}{\partial\beta_{0}} & \frac{\partial\mu_{in_{i}}}{\partial\beta_{1}} \end{matrix} \right]=\left[ \begin{matrix} \begin{matrix} \mu_{i1}\left( 1-\mu_{i1} \right) \end{matrix} & X_{i1}\mu_{i1}\left( 1-\mu_{i1} \right) \\ \vdots& \vdots\\ \mu_{in_{i}}\left( 1-\mu_{in_{i}} \right) & X_{in_{i}}\mu_{{in}_{i}}\left( 1-\mu_{{in}_{i}} \right) \end{matrix} \right]$$

$$\mathbf{D}_{i}^{T}\mathbf{V}_{i}^{-1}\mathbf{D}_{i}=$$

$$\left[ \begin{matrix} \begin{matrix} \mu_{i1}\left( 1-\mu_{i1} \right) \end{matrix} & \cdots& \mu_{{in}_{i}}\left( 1-\mu_{in_{i}} \right) \\ X_{i1}\mu_{i1}\left( 1-\mu_{i1} \right) & \cdots& X_{in_{i}}\mu_{in_{i}}\left( 1-\mu_{{in}_{i}} \right) \end{matrix} \right]\left[ \begin{matrix} \left[ \mu_{i1}\left( 1-\mu_{i1} \right) \right]^{-1} & 0 & \cdots& 0 \\ 0 & \left[ \mu_{i2}\left( 1-\mu_{i2} \right) \right]^{-1} & \ldots& 0 \\ \vdots& \vdots& \ddots& \vdots\\ 0 & 0 & \cdots& \left[ \mu_{{in}_{i}}\left( 1-\mu_{{in}_{i}} \right) \right]^{-1} \end{matrix} \right]\left[ \begin{matrix} \begin{matrix} \mu_{i1}\left( 1-\mu_{i1} \right) \end{matrix} & X_{i1}\mu_{i1}\left( 1-\mu_{i1} \right) \\ \vdots& \vdots\\ \mu_{{in}_{i}}\left( 1-\mu_{{in}_{i}} \right) & X_{{in}_{i}}\mu_{in_{i}}\left( 1-\mu_{in_{i}} \right) \end{matrix} \right]$$

$$= \left[ \begin{matrix} 1 & \cdots& 1 \\ X_{i1} & \cdots& X_{in_{i}} \end{matrix} \right]\left[ \begin{matrix} \begin{matrix} \mu_{i1}\left( 1-\mu_{i1} \right) \end{matrix} & X_{i1}\mu_{i1}\left( 1-\mu_{i1} \right) \\ \vdots& \vdots\\ \mu_{{in}_{i}}\left( 1-\mu_{{in}_{i}} \right) & X_{in_{i}}\mu_{{in}_{i}}\left( 1-\mu_{{in}_{i}} \right) \end{matrix} \right]= \left[ \begin{matrix} \begin{matrix} \sum_{j=1}^{n_{i}} \mu_{ij}\left( 1-\mu_{ij} \right) \end{matrix} & \sum_{j=1}^{n_{i}} X_{ij}\mu_{ij}\left( 1-\mu_{ij} \right) \\ \sum_{j=1}^{n_{i}} X_{ij}\mu_{ij}\left( 1-\mu_{ij} \right) & \sum_{j=1}^{n_{i}} X_{ij}^{2}\mu_{ij}\left( 1-\mu_{ij} \right) \end{matrix} \right]$$

Note that when $n_{i}=1$, this equals $\left[ \begin{matrix} \mu_{i1}(1-\mu_{i1}) & X_{i1}\mu_{i1}(1-\mu_{i1}) \\ X_{i1}\mu_{i1}(1-\mu_{i1}) & X_{i1}^{2}\mu_{i1}(1-\mu_{i1}) \end{matrix} \right]$ and therefore this expression for $\mathbf{D}_{i}^{T}\mathbf{V}_{i}^{-1}\mathbf{D}_{i}$ holds for all $n_{i}\geq1$.

$$\mathbf{D}_{i}^{T}\mathbf{V}_{i}^{-1}{\mathrm{cov}\left( \mathbf{Y}_{i} \right)\mathbf{V}_{i}^{-1}\mathbf{D}}_{i}=$$

$$\left[ \begin{matrix} \begin{matrix} \mu_{i1}\left( 1-\mu_{i1} \right) \end{matrix} & \cdots& \mu_{{in}_{i}}\left( 1-\mu_{{in}_{i}} \right) \\ X_{i1}\mu_{i1}\left( 1-\mu_{i1} \right) & \cdots& X_{{in}_{i}}\mu_{{in}_{i}}\left( 1-\mu_{in_{i}} \right) \end{matrix} \right]\left[ \begin{matrix} \left[ \mu_{i1}\left( 1-\mu_{i1} \right) \right]^{-1} & 0 & \cdots& 0 \\ 0 & \left[ \mu_{i2}\left( 1-\mu_{i2} \right) \right]^{-1} & \ldots& 0 \\ \vdots& \vdots& \ddots& \vdots\\ 0 & 0 & \cdots& \left[ \mu_{{in}_{i}}\left( 1-\mu_{{in}_{i}} \right) \right]^{-1} \end{matrix} \right]\times\left[ \begin{matrix} \mu_{i1}\left( 1-\mu_{i1} \right) & \rho\sqrt{\mu_{i1}\mu_{i2}\left( 1-\mu_{i1} \right)\left( 1-\mu_{i2} \right)} & \cdots& \rho\sqrt{\mu_{i1}\mu_{{in}_{i}}\left( 1-\mu_{i1} \right)\left( 1-\mu_{in_{i}} \right)} \\ \rho\sqrt{\mu_{i1}\mu_{i2}\left( 1-\mu_{i1} \right)\left( 1-\mu_{i2} \right)} & \mu_{i2}\left( 1-\mu_{i2} \right) & \ldots& \rho\sqrt{\mu_{i2}\mu_{in_{i}}\left( 1-\mu_{i2} \right)\left( 1-\mu_{in_{i}} \right)} \\ \vdots& \vdots& \ddots& \vdots\\ \rho\sqrt{\mu_{i1}\mu_{in_{i}}\left( 1-\mu_{i1} \right)\left( 1-\mu_{in_{i}} \right)} & \rho\sqrt{\mu_{i2}\mu_{in_{i}}\left( 1-\mu_{i2} \right)\left( 1-\mu_{in_{i}} \right)} & \cdots& \mu_{in_{i}}\left( 1-\mu_{in_{i}} \right) \end{matrix} \right]\times\left[ \begin{matrix} \left[ \mu_{i1}\left( 1-\mu_{i1} \right) \right]^{-1} & 0 & \cdots& 0 \\ 0 & \left[ \mu_{i2}\left( 1-\mu_{i2} \right) \right]^{-1} & \ldots& 0 \\ \vdots& \vdots& \ddots& \vdots\\ 0 & 0 & \cdots& \left[ \mu_{{in}_{i}}\left( 1-\mu_{{in}_{i}} \right) \right]^{-1} \end{matrix} \right]\left[ \begin{matrix} \begin{matrix} \mu_{i1}\left( 1-\mu_{i1} \right) \end{matrix} & X_{i1}\mu_{i1}\left( 1-\mu_{i1} \right) \\ \vdots& \vdots\\ \mu_{in_{i}}\left( 1-\mu_{in_{i}} \right) & X_{in_{i}}\mu_{in_{i}}\left( 1-\mu_{in_{i}} \right) \end{matrix} \right]$$

$$=\left[ \begin{matrix} 1 & \cdots& 1 \\ X_{i1} & \cdots& X_{in_{i}} \end{matrix} \right] \left[ \begin{matrix} \mu_{i1}\left( 1-\mu_{i1} \right) & \rho\sqrt{\mu_{i1}\mu_{i2}\left( 1-\mu_{i1} \right)\left( 1-\mu_{i2} \right)} & \cdots& \rho\sqrt{\mu_{i1}\mu_{in_{i}}\left( 1-\mu_{i1} \right)\left( 1-\mu_{in_{i}} \right)} \\ \rho\sqrt{\mu_{i1}\mu_{i2}\left( 1-\mu_{i1} \right)\left( 1-\mu_{i2} \right)} & \mu_{i2}\left( 1-\mu_{i2} \right) & \ldots& \rho\sqrt{\mu_{i2}\mu_{in_{i}}\left( 1-\mu_{i2} \right)\left( 1-\mu_{{in}_{i}} \right)} \\ \vdots& \vdots& \ddots& \vdots\\ \rho\sqrt{\mu_{i1}\mu_{in_{i}}\left( 1-\mu_{i1} \right)\left( 1-\mu_{in_{i}} \right)} & \rho\sqrt{\mu_{i2}\mu_{{in}_{i}}\left( 1-\mu_{i2} \right)\left( 1-\mu_{in_{i}} \right)} & \cdots& \mu_{in_{i}}\left( 1-\mu_{{in}_{i}} \right) \end{matrix} \right]\left[ \begin{matrix} 1 & X_{i1} \\ \vdots& \vdots\\ 1 & X_{{in}_{i}} \end{matrix} \right]$$

$$=\left[ \begin{matrix} (1-\rho)\mu_{i1}\left( 1-\mu_{i1} \right)+\rho\sum_{j=1}^{n_{i}} \sqrt{\mu_{i1}\mu_{ij}\left( 1-\mu_{i1} \right)\left( 1-\mu_{ij} \right)} & \cdots& (1-\rho)\mu_{{in}_{i}}\left( 1-\mu_{{in}_{i}} \right)+\rho\sum_{j=1}^{n_{i}} \sqrt{\mu_{{in}_{i}}\mu_{ij}\left( 1-\mu_{in_{i}} \right)\left( 1-\mu_{ij} \right)} \\ {(1-\rho)X_{i1}\mu}_{i1}\left( 1-\mu_{i1} \right)+\rho\sum_{j=1}^{n_{i}} X_{ij}\sqrt{\mu_{i1}\mu_{ij}\left( 1-\mu_{i1} \right)\left( 1-\mu_{ij} \right)} & \cdots& (1-\rho){X_{{in}_{i}}\mu}_{in_{i}}\left( 1-\mu_{{in}_{i}} \right)+\rho\sum_{j=1}^{n_{i}} X_{ij}\sqrt{\mu_{{in}_{i}}\mu_{ij}\left( 1-\mu_{in_{i}} \right)\left( 1-\mu_{ij} \right)} \end{matrix} \right]\left[ \begin{matrix} 1 & X_{i1} \\ \vdots& \vdots\\ 1 & X_{in_{i}} \end{matrix} \right]$$

$$=\left[ \begin{matrix} \sum_{j=1}^{n_{i}} \mu_{ij}\left( 1-\mu_{ij} \right)+2\rho\sum_{j=1}^{n_{i}} \sum_{a\neq j} \sqrt{\mu_{ij}\mu_{ia}\left( 1-\mu_{ij} \right)\left( 1-\mu_{ia} \right)} & \sum_{j=1}^{n_{i}} X_{ij}\mu_{ij}\left( 1-\mu_{ij} \right)+\rho\sum_{j=1}^{n_{i}} \sum_{a\neq j} (X_{ij}+X_{ia})\sqrt{\mu_{ij}\mu_{ia}\left( 1-\mu_{ij} \right)\left( 1-\mu_{ia} \right)} \\ \sum_{j=1}^{n_{i}} X_{ij}\mu_{ij}\left( 1-\mu_{ij} \right)+\rho\sum_{j=1}^{n_{i}} \sum_{a\neq j} (X_{ij}+X_{ia})\sqrt{\mu_{ij}\mu_{ia}\left( 1-\mu_{ij} \right)\left( 1-\mu_{ia} \right)} & \sum_{j=1}^{n_{i}} X_{ij}^{2}\mu_{ij}\left( 1-\mu_{ij} \right)+2\rho\sum_{j=1}^{n_{i}} \sum_{a\neq j} X_{ij}X_{ia}\sqrt{\mu_{ij}\mu_{ia}\left( 1-\mu_{ij} \right)\left( 1-\mu_{ia} \right)} \end{matrix} \right]$$

Note that when $n_{i}=1$, this reduces to $\left[ \begin{matrix} \mu_{i1}(1-\mu_{i1}) & X_{i1}\mu_{i1}(1-\mu_{i1}) \\ X_{i1}\mu_{i1}(1-\mu_{i1}) & X_{i1}^{2}\mu_{i1}(1-\mu_{i1}) \end{matrix} \right]$ and therefore this expression for $\mathbf{D}_{i}^{T}\mathbf{V}_{i}^{-1}{\mathrm{cov}\left( \mathbf{Y}_{i} \right)\mathbf{V}_{i}^{-1}\mathbf{D}}_{i}$ holds for all $n_{i}\geq1$.

The first sum in each element of this matrix can be rewritten as the sum over cluster members assigned to intervention plus the sum over the cluster members assigned to control (e.g. $\sum_{j=1}^{n_{i}} \mu_{j}\left( 1-\mu_{j} \right)$ can be rewritten as $\sum_{j=1|X_{ij}=1}^{n_{i}} \mu_{j}\left( 1-\mu_{j} \right)+\sum_{j=1|X_{ij}=0}^{n_{i}} \mu_{j}\left( 1-\mu_{j} \right)$). A similar approach can be used for the double sums in each element of the matrix, by rewriting these as the sums of paired terms where the two cluster members are both assigned to intervention, both assigned to control, and one to intervention and one to control. For example, $\sum_{j=1}^{n_{i}} \sum_{a\neq j} \sqrt{\mu_{ij}\mu_{ia}\left( 1-\mu_{ij} \right)\left( 1-\mu_{ia} \right)}=\sum_{j=1|X_{ij}=1}^{n_{i}} \sum_{a\neq j|X_{ia}=1} \sqrt{\mu_{ij}\mu_{ia}\left( 1-\mu_{ij} \right)\left( 1-\mu_{ia} \right)}+\sum_{j=1|X_{ij}=0}^{n_{i}} \sum_{a\neq j|X_{ia}=0} \sqrt{\mu_{ij}\mu_{ia}\left( 1-\mu_{ij} \right)\left( 1-\mu_{ia} \right)}+\sum_{j=1|X_{ij}=1}^{n_{i}} \sum_{a\neq j|X_{ia}=0} \sqrt{\mu_{ij}\mu_{ia}\left( 1-\mu_{ij} \right)\left( 1-\mu_{ia} \right)}$. The sum over pairs where both are assigned to intervention is a sum of $\binom{t_{i}}{2}=t_{i}(t_{i}-1)/2$ terms, the sum over pairs where both are assigned to control is a sum of $\binom{c_{i}}{2}=c_{i}(c_{i}-1)/2$ terms, and the sum over pairs with one member assigned to intervention and one to control is a sum of $t_{i}c_{i}$ terms.

$\Rightarrow\mathbf{D}_{i}^{T}\mathbf{V}_{i}^{-1}{\mathrm{cov}\left( \mathbf{Y}_{i} \right)\mathbf{V}_{i}^{-1}\mathbf{D}}_{i}=\left[ \begin{matrix} a & b \\ b & c \end{matrix} \right]$, where

$$a=\sum_{j=1}^{n_{i}} \mu_{ij}\left( 1-\mu_{ij} \right)+2\rho\sum_{j=1}^{n_{i}} \sum_{a\neq j} \sqrt{\mu_{ij}\mu_{ia}\left( 1-\mu_{ij} \right)\left( 1-\mu_{ia} \right)}$$

$$=\pi_{I}\left( 1-\pi_{I} \right)t_{i}+\pi_{C}\left( 1-\pi_{C} \right)c_{i}+2\rho\left[ \sqrt{\pi_{I}^{2}\left( 1-\pi_{I} \right)^{2}}\frac{t_{i}\left( t_{i}-1 \right)}{2}+\sqrt{\pi_{C}^{2}\left( 1-\pi_{C} \right)^{2}}\frac{c_{i}\left( c_{i}-1 \right)}{2}+\sqrt{\pi_{I}\pi_{C}\left( 1-\pi_{I} \right)\left( 1-\pi_{C} \right)}t_{i}c_{i} \right]$$

$$=\pi_{I}\left( 1-\pi_{I} \right)t_{i}+\pi_{C}\left( 1-\pi_{C} \right)c_{i}+\pi_{I}\left( 1-\pi_{I} \right)\rho t_{i}\left( t_{i}-1 \right)+\pi_{C}\left( 1-\pi_{C} \right)\rho c_{i}\left( c_{i}-1 \right)+2\sqrt{\pi_{I}\pi_{C}\left( 1-\pi_{I} \right)\left( 1-\pi_{C} \right)}\rho t_{i}c_{i}$$

$$b=\sum_{j=1}^{n_{i}} X_{ij}\mu_{ij}\left( 1-\mu_{j} \right)+\rho\sum_{j=1}^{n_{i}} \sum_{a\neq j} \left( X_{ij}+X_{ia} \right)\sqrt{\mu_{ij}\mu_{ia}\left( 1-\mu_{ij} \right)\left( 1-\mu_{ia} \right)}$$

$$=\left( 1 \right)\pi_{I}\left( 1-\pi_{I} \right)t_{i}+\rho\left[ \left( 1+1 \right)\sqrt{\pi_{I}^{2}\left( 1-\pi_{I} \right)^{2}}\frac{t_{i}\left( t_{i}-1 \right)}{2}+\left( 1+0 \right)\sqrt{\pi_{I}\pi_{C}\left( 1-\pi_{I} \right)\left( 1-\pi_{C} \right)}t_{i}c_{i} \right]$$

$$=\pi_{I}\left( 1-\pi_{I} \right)t_{i}+\pi_{I}\left( 1-\pi_{I} \right)\rho t_{i}\left( t_{i}-1 \right)+\sqrt{\pi_{I}\pi_{C}\left( 1-\pi_{I} \right)\left( 1-\pi_{C} \right)}\rho t_{i}c_{i}$$

$$c=\sum_{j=1}^{n_{i}} X_{ij}^{2}\mu_{ij}\left( 1-\mu_{ij} \right)+2\rho\sum_{j=1}^{n_{i}} \sum_{a\neq j} X_{ij}X_{ia}\sqrt{\mu_{ij}\mu_{ia}\left( 1-\mu_{ij} \right)\left( 1-\mu_{ia} \right)}=\left( 1 \right)^{2}\pi_{I}\left( 1-\pi_{I} \right)t_{i}+2\rho\left[ \left( 1 \right)\left( 1 \right)\sqrt{\pi_{I}^{2}\left( 1-\pi_{I} \right)^{2}}\frac{t_{i}\left( t_{i}-1 \right)}{2} \right]$$

$$=\pi_{I}\left( 1-\pi_{I} \right)t_{i}+\pi_{I}\left( 1-\pi_{I} \right)\rho t_{i}\left( t_{i}-1 \right)$$

Sum over all clusters $i=1,\ldots, M$. Cluster $i$ is of size $n_{i}$and contains $t_{i}$ members assigned to intervention and $c_{i}$ members assigned to control.

$$\sum_{i=1}^{M} \mathbf{D}_{i}^{T}\mathbf{V}_{i}^{-1}\mathbf{D}_{i}= \sum_{i=1}^{M} \left[ \begin{matrix} \begin{matrix} \sum_{j=1}^{n_{i}} \mu_{ij}\left( 1-\mu_{ij} \right) \end{matrix} & \sum_{j=1}^{n_{i}} X_{ij}\mu_{ij}\left( 1-\mu_{ij} \right) \\ \sum_{j=1}^{n_{i}} X_{ij}\mu_{ij}\left( 1-\mu_{ij} \right) & \sum_{j=1}^{n_{i}} X_{ij}^{2}\mu_{ij}\left( 1-\mu_{ij} \right) \end{matrix} \right]$$

$$=\sum_{i=1}^{M} \left[ \begin{matrix} {t_{i}\pi}_{I}\left( 1-\pi_{I} \right)+{c_{i}\pi}_{C}\left( 1-\pi_{C} \right) & {t_{i}\pi}_{I}\left( 1-\pi_{I} \right) \\ {t_{i}\pi}_{I}\left( 1-\pi_{I} \right) & {t_{i}\pi}_{I}\left( 1-\pi_{I} \right) \end{matrix} \right] = \left[ \begin{matrix} {N_{I}\pi}_{I}\left( 1-\pi_{I} \right)+{N_{C}\pi}_{C}\left( 1-\pi_{C} \right) & {N_{I}\pi}_{I}\left( 1-\pi_{I} \right) \\ {N_{I}\pi}_{I}\left( 1-\pi_{I} \right) & {N_{I}\pi}_{I}\left( 1-\pi_{I} \right) \end{matrix} \right]$$

$=\frac{N}{2}\left[ \begin{matrix} \pi_{I}\left( 1-\pi_{I} \right)+\pi_{C}\left( 1-\pi_{C} \right) & \pi_{I}\left( 1-\pi_{I} \right) \\ \pi_{I}\left( 1-\pi_{I} \right) & \pi_{I}\left( 1-\pi_{I} \right) \end{matrix} \right]$ under the assumption of overall treatment balance

$$\left[ \sum_{i=1}^{M} \mathbf{D}_{i}^{T}\mathbf{V}_{i}^{-1}\mathbf{D}_{i} \right]^{-1}= \frac{2}{N(\pi_{I}\left( 1-\pi_{I} \right)(\pi_{I}\left( 1-\pi_{I} \right)+\pi_{C}\left( 1-\pi_{C} \right)-\pi_{I}\left( 1-\pi_{I} \right)\pi_{I}\left( 1-\pi_{I} \right))}\left[ \begin{matrix} \pi_{I}\left( 1-\pi_{I} \right) & -\pi_{I}\left( 1-\pi_{I} \right) \\ {-\pi}_{I}\left( 1-\pi_{I} \right) & \pi_{I}\left( 1-\pi_{I} \right)+\pi_{C}\left( 1-\pi_{C} \right) \end{matrix} \right]$$

$$=\frac{2}{N\pi_{I}\pi_{C}\left( 1-\pi_{I} \right)\left( 1-\pi_{C} \right)}\left[ \begin{matrix} \pi_{I}\left( 1-\pi_{I} \right) & -\pi_{I}\left( 1-\pi_{I} \right) \\ {-\pi}_{I}\left( 1-\pi_{I} \right) & \pi_{I}\left( 1-\pi_{I} \right)+\pi_{C}\left( 1-\pi_{C} \right) \end{matrix} \right]$$

$\sum_{i=1}^{M} \mathbf{D}_{i}^{T}\mathbf{V}_{i}^{-1}{\mathrm{cov}\left( \mathbf{Y}_{i} \right)\mathbf{V}_{i}^{-1}\mathbf{D}}_{i}=\left[ \begin{matrix} \sum_{i=1}^{M} a & \sum_{i=1}^{M} b \\ \sum_{i=1}^{M} b & \sum_{i=1}^{M} c \end{matrix} \right]= \left[ \begin{matrix} A & B \\ B & C \end{matrix} \right]$ , where under the assumption of overall treatment balance:

$$A=\sum_{i=1}^{M} \left( \pi_{I}\left( 1-\pi_{I} \right)t_{i}+\pi_{C}\left( 1-\pi_{C} \right)c_{i}+\pi_{I}\left( 1-\pi_{I} \right)\rho t_{i}\left( t_{i}-1 \right)+\pi_{C}\left( 1-\pi_{C} \right)\rho c_{i}\left( c_{i}-1 \right)+2\sqrt{\pi_{I}\pi_{C}\left( 1-\pi_{I} \right)\left( 1-\pi_{C} \right)}\rho t_{i}c_{i} \right)$$

$$={N_{I}\pi}_{I}\left( 1-\pi_{I} \right)+N_{C}\pi_{C}\left( 1-\pi_{C} \right)+\pi_{I}\left( 1-\pi_{I} \right)\rho\sum_{i=1}^{M} t_{i}\left( t_{i}-1 \right)+\pi_{C}\left( 1-\pi_{C} \right)\rho\sum_{i=1}^{M} c_{i}\left( c_{i}-1 \right)+2\sqrt{\pi_{I}\pi_{C}\left( 1-\pi_{I} \right)\left( 1-\pi_{C} \right)}\rho\sum_{i=1}^{M} t_{i}c_{i}$$

$$=\frac{N}{2}\left[ \pi_{I}\left( 1-\pi_{I} \right)+\pi_{C}\left( 1-\pi_{C} \right) \right]+\pi_{I}\left( 1-\pi_{I} \right)\rho\sum_{i=1}^{M} t_{i}\left( t_{i}-1 \right)+\pi_{C}\left( 1-\pi_{C} \right)\rho\sum_{i=1}^{M} c_{i}\left( c_{i}-1 \right)+2\sqrt{\pi_{I}\pi_{C}\left( 1-\pi_{I} \right)\left( 1-\pi_{C} \right)}\rho\sum_{i=1}^{M} t_{i}c_{i}$$

$$B=\sum_{i=1}^{M} \left( \pi_{I}\left( 1-\pi_{I} \right)t_{i}+\pi_{I}\left( 1-\pi_{I} \right)\rho t_{i}\left( t_{i}-1 \right)+\sqrt{\pi_{I}\pi_{C}\left( 1-\pi_{I} \right)\left( 1-\pi_{C} \right)}\rho t_{i}c_{i} \right)$$

$$={N_{I}\pi}_{I}\left( 1-\pi_{I} \right)+\pi_{I}\left( 1-\pi_{I} \right)\rho\sum_{i=1}^{M} t_{i}\left( t_{i}-1 \right)+\sqrt{\pi_{I}\pi_{C}\left( 1-\pi_{I} \right)\left( 1-\pi_{C} \right)}\rho\sum_{i=1}^{M} t_{i}c_{i}$$

$$={\frac{N}{2}\pi}_{I}\left( 1-\pi_{I} \right)+\pi_{I}\left( 1-\pi_{I} \right)\rho\sum_{i=1}^{M} t_{i}\left( t_{i}-1 \right)+\sqrt{\pi_{I}\pi_{C}\left( 1-\pi_{I} \right)\left( 1-\pi_{C} \right)}\rho\sum_{i=1}^{M} t_{i}c_{i}$$

$$C=\sum_{i=1}^{M} \left( \pi_{I}\left( 1-\pi_{I} \right)t_{i}+\pi_{I}\left( 1-\pi_{I} \right)\rho t_{i}\left( t_{i}-1 \right) \right) = {N_{I}\pi}_{I}\left( 1-\pi_{I} \right)+\pi_{I}\left( 1-\pi_{I} \right)\rho\sum_{i=1}^{M} t_{i}\left( t_{i}-1 \right) = {\frac{N}{2}\pi}_{I}\left( 1-\pi_{I} \right)+\pi_{I}\left( 1-\pi_{I} \right)\rho\sum_{i=1}^{M} t_{i}\left( t_{i}-1 \right)$$

$$\mathrm{cov}\left( \hat{\boldsymbol{\beta}} \right)= \left[ \sum_{i=1}^{M} \mathbf{D}_{i}^{T}\mathbf{V}_{i}^{-1}\mathbf{D}_{i} \right]^{-1}\sum_{i=1}^{M} \mathbf{D}_{i}^{T}\mathbf{V}_{i}^{-1}{\mathrm{cov}\left( \mathbf{Y}_{i} \right)\mathbf{V}_{i}^{-1}\mathbf{D}}_{i}\left[ \sum_{i=1}^{M} \mathbf{D}_{i}^{T}\mathbf{V}_{i}^{-1}\mathbf{D}_{i} \right]^{-1}$$

$$= \frac{2}{N\pi_{I}\pi_{C}\left( 1-\pi_{I} \right)\left( 1-\pi_{C} \right)}\left[ \begin{matrix} \pi_{I}\left( 1-\pi_{I} \right) & -\pi_{I}\left( 1-\pi_{I} \right) \\ {-\pi}_{I}\left( 1-\pi_{I} \right) & \pi_{I}\left( 1-\pi_{I} \right)+\pi_{C}\left( 1-\pi_{C} \right) \end{matrix} \right] \left[ \begin{matrix} A & B \\ B & C \end{matrix} \right]\frac{2}{N\pi_{I}\pi_{C}\left( 1-\pi_{I} \right)\left( 1-\pi_{C} \right)}\left[ \begin{matrix} \pi_{I}\left( 1-\pi_{I} \right) & -\pi_{I}\left( 1-\pi_{I} \right) \\ {-\pi}_{I}\left( 1-\pi_{I} \right) & \pi_{I}\left( 1-\pi_{I} \right)+\pi_{C}\left( 1-\pi_{C} \right) \end{matrix} \right]$$

$$=\frac{4}{\left[ N\pi_{I}\pi_{C}\left( 1-\pi_{I} \right)\left( 1-\pi_{C} \right) \right]^{2}}\left[ \begin{matrix} D & E \\ F & G \end{matrix} \right]\left[ \begin{matrix} \pi_{I}\left( 1-\pi_{I} \right) & -\pi_{I}\left( 1-\pi_{I} \right) \\ {-\pi}_{I}\left( 1-\pi_{I} \right) & \pi_{I}\left( 1-\pi_{I} \right)+\pi_{C}\left( 1-\pi_{C} \right) \end{matrix} \right]$$

where:

$$D=\pi_{I}\left( 1-\pi_{I} \right)A-\pi_{I}\left( 1-\pi_{I} \right)B$$

$$=\pi_{I}\left( 1-\pi_{I} \right)\left[ \frac{N}{2}\left[ \pi_{I}\left( 1-\pi_{I} \right)+\pi_{C}\left( 1-\pi_{C} \right) \right]+\pi_{I}\left( 1-\pi_{I} \right)\rho\sum_{i=1}^{M} t_{i}\left( t_{i}-1 \right)+\pi_{C}\left( 1-\pi_{C} \right)\rho\sum_{i=1}^{M} c_{i}\left( c_{i}-1 \right)+2\sqrt{\pi_{I}\pi_{C}\left( 1-\pi_{I} \right)\left( 1-\pi_{C} \right)}\rho\sum_{i=1}^{M} t_{i}c_{i} \right]-\pi_{I}\left( 1-\pi_{I} \right)\left[ {\frac{N}{2}\pi}_{I}\left( 1-\pi_{I} \right)+\pi_{I}\left( 1-\pi_{I} \right)\rho\sum_{i=1}^{M} t_{i}\left( t_{i}-1 \right)+\sqrt{\pi_{I}\pi_{C}\left( 1-\pi_{I} \right)\left( 1-\pi_{C} \right)}\rho\sum_{i=1}^{M} t_{i}c_{i} \right]$$

$$=\frac{N}{2}\pi_{I}\pi_{C}\left( 1-\pi_{I} \right)\left( 1-\pi_{C} \right)+\pi_{I}\pi_{C}\left( 1-\pi_{I} \right)\left( 1-\pi_{C} \right)\rho\sum_{i=1}^{M} c_{i}\left( c_{i}-1 \right)+\pi_{I}\left( 1-\pi_{I} \right)\sqrt{\pi_{I}\pi_{C}\left( 1-\pi_{I} \right)\left( 1-\pi_{C} \right)}\rho\sum_{i=1}^{M} t_{i}c_{i}$$

$${E=\pi}_{I}\left( 1-\pi_{I} \right)B-\pi_{I}\left( 1-\pi_{I} \right)C$$

$$=\pi_{I}\left( 1-\pi_{I} \right)\left[ {\frac{N}{2}\pi}_{I}\left( 1-\pi_{I} \right)+\pi_{I}\left( 1-\pi_{I} \right)\rho\sum_{i=1}^{M} t_{i}\left( t_{i}-1 \right)+\sqrt{\pi_{I}\pi_{C}\left( 1-\pi_{I} \right)\left( 1-\pi_{C} \right)}\rho\sum_{i=1}^{M} t_{i}c_{i} \right]-\pi_{I}\left( 1-\pi_{I} \right)\left[ {\frac{N}{2}\pi}_{I}\left( 1-\pi_{I} \right)+\pi_{I}\left( 1-\pi_{I} \right)\rho\sum_{i=1}^{M} t_{i}\left( t_{i}-1 \right) \right]$$

$$=\pi_{I}\left( 1-\pi_{I} \right)\sqrt{\pi_{I}\pi_{C}\left( 1-\pi_{I} \right)\left( 1-\pi_{C} \right)}\rho\sum_{i=1}^{M} t_{i}c_{i}$$

$$F={-\pi}_{I}\left( 1-\pi_{I} \right)A+\left[ \pi_{I}\left( 1-\pi_{I} \right)+\pi_{C}\left( 1-\pi_{C} \right) \right]B$$

$$={-\pi}_{I}\left( 1-\pi_{I} \right)\left[ \frac{N}{2}\left[ \pi_{I}\left( 1-\pi_{I} \right)+\pi_{C}\left( 1-\pi_{C} \right) \right]+\pi_{I}\left( 1-\pi_{I} \right)\rho\sum_{i=1}^{M} t_{i}\left( t_{i}-1 \right)+\pi_{C}\left( 1-\pi_{C} \right)\rho\sum_{i=1}^{M} c_{i}\left( c_{i}-1 \right)+2\sqrt{\pi_{I}\pi_{C}\left( 1-\pi_{I} \right)\left( 1-\pi_{C} \right)}\rho\sum_{i=1}^{M} t_{i}c_{i} \right]+\left[ \pi_{I}\left( 1-\pi_{I} \right)+\pi_{C}\left( 1-\pi_{C} \right) \right]\left[ {\frac{N}{2}\pi}_{I}\left( 1-\pi_{I} \right)+\pi_{I}\left( 1-\pi_{I} \right)\rho\sum_{i=1}^{M} t_{i}\left( t_{i}-1 \right)+\sqrt{\pi_{I}\pi_{C}\left( 1-\pi_{I} \right)\left( 1-\pi_{C} \right)}\rho\sum_{i=1}^{M} t_{i}c_{i} \right]$$

$$=\pi_{I}\pi_{C}\left( 1-\pi_{I} \right)\left( 1-\pi_{C} \right)\rho\sum_{i=1}^{M} t_{i}\left( t_{i}-1 \right)-{\pi_{I}\left( 1-\pi_{I} \right)\pi}_{C}\left( 1-\pi_{C} \right)\rho\sum_{i=1}^{M} c_{i}\left( c_{i}-1 \right)-\left[ \pi_{I}\left( 1-\pi_{I} \right)-\pi_{C}\left( 1-\pi_{C} \right) \right]\sqrt{\pi_{I}\pi_{C}\left( 1-\pi_{I} \right)\left( 1-\pi_{C} \right)}\rho\sum_{i=1}^{M} t_{i}c_{i}$$

$$G={-\pi}_{I}\left( 1-\pi_{I} \right)B+\left[ \pi_{I}\left( 1-\pi_{I} \right)+\pi_{C}\left( 1-\pi_{C} \right) \right]C$$

$$={-\pi}_{I}\left( 1-\pi_{I} \right)\left[ {\frac{N}{2}\pi}_{I}\left( 1-\pi_{I} \right)+\pi_{I}\left( 1-\pi_{I} \right)\rho\sum_{i=1}^{M} t_{i}\left( t_{i}-1 \right)+\sqrt{\pi_{I}\pi_{C}\left( 1-\pi_{I} \right)\left( 1-\pi_{C} \right)}\rho\sum_{i=1}^{M} t_{i}c_{i} \right]+\left[ \pi_{I}\left( 1-\pi_{I} \right)+\pi_{C}\left( 1-\pi_{C} \right) \right]\left[ {\frac{N}{2}\pi}_{I}\left( 1-\pi_{I} \right)+\pi_{I}\left( 1-\pi_{I} \right)\rho\sum_{i=1}^{M} t_{i}\left( t_{i}-1 \right) \right]$$

$$={\frac{N}{2}\pi}_{I}\pi_{C}\left( 1-\pi_{I} \right)\left( 1-\pi_{C} \right)+\pi_{I}\pi_{C}\left( 1-\pi_{I} \right)\left( 1-\pi_{C} \right)\rho\sum_{i=1}^{M} t_{i}\left( t_{i}-1 \right)-\pi_{I}\left( 1-\pi_{I} \right)\sqrt{\pi_{I}\pi_{C}\left( 1-\pi_{I} \right)\left( 1-\pi_{C} \right)}\rho\sum_{i=1}^{M} t_{i}c_{i}$$

$$\Rightarrow\mathrm{cov}\left( \hat{\boldsymbol{\beta}} \right)=\frac{4}{\left[ N\pi_{I}\pi_{C}\left( 1-\pi_{I} \right)\left( 1-\pi_{C} \right) \right]^{2}}\left[ \begin{matrix} D & E \\ F & G \end{matrix} \right]\left[ \begin{matrix} \pi_{I}\left( 1-\pi_{I} \right) & -\pi_{I}\left( 1-\pi_{I} \right) \\ {-\pi}_{I}\left( 1-\pi_{I} \right) & \pi_{I}\left( 1-\pi_{I} \right)+\pi_{C}\left( 1-\pi_{C} \right) \end{matrix} \right]$$

$$=\frac{4}{\left[ N\pi_{I}\pi_{C}\left( 1-\pi_{I} \right)\left( 1-\pi_{C} \right) \right]^{2}} \left[ \begin{matrix} \pi_{I}\left( 1-\pi_{I} \right)D{-\pi}_{I}\left( 1-\pi_{I} \right)E & -\pi_{I}\left( 1-\pi_{I} \right)D+\left[ \pi_{I}\left( 1-\pi_{I} \right)+\pi_{C}\left( 1-\pi_{C} \right) \right]E \\ \pi_{I}\left( 1-\pi_{I} \right)F{-\pi}_{I}\left( 1-\pi_{I} \right)G & -\pi_{I}\left( 1-\pi_{I} \right)F+\left[ \pi_{I}\left( 1-\pi_{I} \right)+\pi_{C}\left( 1-\pi_{C} \right) \right]G \end{matrix} \right]$$

$$\mathrm{var}\left( \hat{\beta}_{1} \right)= \frac{4}{\left[ N\pi_{I}\pi_{C}\left( 1-\pi_{I} \right)\left( 1-\pi_{C} \right) \right]^{2}}H$$

where $H=-\pi_{I}\left( 1-\pi_{I} \right)F+\left[ \pi_{I}\left( 1-\pi_{I} \right)+\pi_{C}\left( 1-\pi_{C} \right) \right]G$

$$H=-\pi_{I}\left( 1-\pi_{I} \right)\left[ \pi_{I}\pi_{C}\left( 1-\pi_{I} \right)\left( 1-\pi_{C} \right)\rho\sum_{i=1}^{M} t_{i}\left( t_{i}-1 \right)-\pi_{I}\pi_{C}\left( 1-\pi_{I} \right)\left( 1-\pi_{C} \right)\rho\sum_{i=1}^{M} c_{i}\left( c_{i}-1 \right)-\left[ \pi_{I}\left( 1-\pi_{I} \right)-\pi_{C}\left( 1-\pi_{C} \right) \right]\sqrt{\pi_{I}\pi_{C}\left( 1-\pi_{I} \right)\left( 1-\pi_{C} \right)}\rho\sum_{i=1}^{M} t_{i}c_{i} \right]+\left[ \pi_{I}\left( 1-\pi_{I} \right)+\pi_{C}\left( 1-\pi_{C} \right) \right]\left[ {\frac{N}{2}\pi}_{I}\pi_{C}\left( 1-\pi_{I} \right)\left( 1-\pi_{C} \right)+\pi_{I}\pi_{C}\left( 1-\pi_{I} \right)\left( 1-\pi_{C} \right)\rho\sum_{i=1}^{M} t_{i}\left( t_{i}-1 \right)-\pi_{I}\left( 1-\pi_{I} \right)\sqrt{\pi_{I}\pi_{C}\left( 1-\pi_{I} \right)\left( 1-\pi_{C} \right)}\rho\sum_{i=1}^{M} t_{i}c_{i} \right]$$

$$=\frac{N}{2}\pi_{I}\pi_{C}\left( 1-\pi_{I} \right)\left( 1-\pi_{C} \right)\left( \pi_{I}\left( 1-\pi_{I} \right)+\pi_{C}\left( 1-\pi_{C} \right) \right)+\pi_{I}\left( 1-\pi_{I} \right)\pi_{C}^{2}\left( 1-\pi_{C} \right)^{2}\rho\sum_{i=1}^{M} t_{i}\left( t_{i}-1 \right)+\pi_{I}^{2}\left( 1-\pi_{I} \right)^{2}\pi_{C}\left( 1-\pi_{C} \right)\rho\sum_{i=1}^{M} c_{i}\left( c_{i}-1 \right)-2\pi_{I}\pi_{C}\left( 1-\pi_{I} \right)\left( 1-\pi_{C} \right)\sqrt{\pi_{I}\pi_{C}\left( 1-\pi_{I} \right)\left( 1-\pi_{C} \right)}\rho\sum_{i=1}^{M} t_{i}c_{i}$$

$$\Rightarrow\mathrm{var}\left( \hat{\beta}_{1} \right)= \frac{4}{\left[ N\pi_{I}\pi_{C}\left( 1-\pi_{I} \right)\left( 1-\pi_{C} \right) \right]^{2}}\left( \frac{N}{2}\pi_{I}\pi_{C}\left( 1-\pi_{I} \right)\left( 1-\pi_{C} \right)\left( \pi_{I}\left( 1-\pi_{I} \right)+\pi_{C}\left( 1-\pi_{C} \right) \right)+\pi_{I}\left( 1-\pi_{I} \right)\pi_{C}^{2}\left( 1-\pi_{C} \right)^{2}\rho\sum_{i=1}^{M} t_{i}\left( t_{i}-1 \right)+\pi_{I}^{2}\left( 1-\pi_{I} \right)^{2}\pi_{C}\left( 1-\pi_{C} \right)\rho\sum_{i=1}^{M} c_{i}\left( c_{i}-1 \right)-2\pi_{I}\pi_{C}\left( 1-\pi_{I} \right)\left( 1-\pi_{C} \right)\sqrt{\pi_{I}\pi_{C}\left( 1-\pi_{I} \right)\left( 1-\pi_{C} \right)}\rho\sum_{i=1}^{M} t_{i}c_{i} \right)$$

$$=\frac{4}{N^{2}\pi_{I}\pi_{C}\left( 1-\pi_{I} \right)\left( 1-\pi_{C} \right)}\left( \frac{N}{2}\left( \pi_{I}\left( 1-\pi_{I} \right)+\pi_{C}\left( 1-\pi_{C} \right) \right)+\pi_{C}\left( 1-\pi_{C} \right)\rho\sum_{i=1}^{M} t_{i}\left( t_{i}-1 \right)+\pi_{I}\left( 1-\pi_{I} \right)\rho\sum_{i=1}^{M} c_{i}\left( c_{i}-1 \right)-2\sqrt{\pi_{I}\pi_{C}\left( 1-\pi_{I} \right)\left( 1-\pi_{C} \right)}\rho\sum_{i=1}^{M} t_{i}c_{i} \right)$$

$$\Rightarrow DEFF=\frac{4}{N^{2}\pi_{I}\pi_{C}\left( 1-\pi_{I} \right)\left( 1-\pi_{C} \right)}\left( \frac{N}{2}\left( \pi_{I}\left( 1-\pi_{I} \right)+\pi_{C}\left( 1-\pi_{C} \right) \right)+\pi_{C}\left( 1-\pi_{C} \right)\rho\sum_{i=1}^{M} t_{i}\left( t_{i}-1 \right)+\pi_{I}\left( 1-\pi_{I} \right)\rho\sum_{i=1}^{M} c_{i}\left( c_{i}-1 \right)-2\sqrt{\pi_{I}\pi_{C}\left( 1-\pi_{I} \right)\left( 1-\pi_{C} \right)}\rho\sum_{i=1}^{M} t_{i}c_{i} \right)\times\frac{N\pi_{I}\pi_{C}\left( 1-\pi_{I} \right)\left( 1-\pi_{C} \right)}{2\left( \pi_{I}\left( 1-\pi_{I} \right)+\pi_{C}\left( 1-\pi_{C} \right) \right)}$$

$$=\frac{2}{N\left( \pi_{I}\left( 1-\pi_{I} \right)+\pi_{C}\left( 1-\pi_{C} \right) \right)}\left( \frac{N}{2}\left( \pi_{I}\left( 1-\pi_{I} \right)+\pi_{C}\left( 1-\pi_{C} \right) \right)+\pi_{C}\left( 1-\pi_{C} \right)\rho\sum_{i=1}^{M} t_{i}\left( t_{i}-1 \right)+\pi_{I}\left( 1-\pi_{I} \right)\rho\sum_{i=1}^{M} c_{i}\left( c_{i}-1 \right)-2\sqrt{\pi_{I}\pi_{C}\left( 1-\pi_{I} \right)\left( 1-\pi_{C} \right)}\rho\sum_{i=1}^{M} t_{i}c_{i} \right)$$

$$=1+\rho\left[ \frac{2}{N\left( \pi_{I}\left( 1-\pi_{I} \right)+\pi_{C}\left( 1-\pi_{C} \right) \right)}\left( \pi_{C}\left( 1-\pi_{C} \right)\sum_{i=1}^{M} t_{i}\left( t_{i}-1 \right)+\pi_{I}\left( 1-\pi_{I} \right)\sum_{i=1}^{M} c_{i}\left( c_{i}-1 \right)-2\sqrt{\pi_{I}\pi_{C}\left( 1-\pi_{I} \right)\left( 1-\pi_{C} \right)}\sum_{i=1}^{M} t_{i}c_{i} \right) \right]$$

- - 1. Cluster randomisation

$$\frac{1}{N}\sum_{i=1}^{M} t_{i}\left( t_{i}-1 \right) = \frac{1}{N}\sum_{k=1}^{K} \left[ \frac{M_{k}}{2}k\left( k-1 \right)+\frac{M_{k}}{2}0(0-1) \right] = \frac{1}{2}\sum_{k=1}^{K} \frac{M_{k}}{N}k\left( k-1 \right) = \frac{1}{2}\sum_{k=1}^{K} {\left( k-1 \right)\gamma}_{k}$$

$$\frac{1}{N}\sum_{i=1}^{M} c_{i}\left( c_{i}-1 \right) = \frac{1}{N}\sum_{k=1}^{K} \left[ \frac{M_{k}}{2}0(0-1)+\frac{M_{k}}{2}k\left( k-1 \right) \right] = \frac{1}{2}\sum_{k=1}^{K} \frac{M_{k}}{N}k\left( k-1 \right) = \frac{1}{2}\sum_{k=1}^{K} {\left( k-1 \right)\gamma}_{k}$$

$$\frac{1}{N}\sum_{i=1}^{M} {t_{i}c}_{i} = \frac{1}{N}\sum_{k=1}^{K} \left[ \frac{M_{k}}{2}k(0)+\frac{M_{k}}{2}(0)k \right] = 0$$

$$DEFF= 1+\rho\left[ \frac{2}{N\left( \pi_{I}\left( 1-\pi_{I} \right)+\pi_{C}\left( 1-\pi_{C} \right) \right)}\left( \pi_{C}\left( 1-\pi_{C} \right)\sum_{i=1}^{M} t_{i}\left( t_{i}-1 \right)+\pi_{I}\left( 1-\pi_{I} \right)\sum_{i=1}^{M} c_{i}\left( c_{i}-1 \right)-2\sqrt{\pi_{I}\pi_{C}\left( 1-\pi_{I} \right)\left( 1-\pi_{C} \right)}\sum_{i=1}^{M} t_{i}c_{i} \right) \right]$$

$$=1+\rho\left[ \frac{2}{\pi_{I}\left( 1-\pi_{I} \right)+\pi_{C}\left( 1-\pi_{C} \right)}\left( \pi_{C}\left( 1-\pi_{C} \right)\left( \frac{1}{2} \right)\sum_{k=1}^{K} {\left( k-1 \right)\gamma}_{k}+\pi_{I}\left( 1-\pi_{I} \right)\left( \frac{1}{2} \right)\sum_{k=1}^{K} {\left( k-1 \right)\gamma}_{k} \right) \right]$$

$$=1+\rho\left[ \frac{1}{\pi_{I}\left( 1-\pi_{I} \right)+\pi_{C}\left( 1-\pi_{C} \right)}\left( \pi_{I}\left( 1-\pi_{I} \right)+\pi_{C}\left( 1-\pi_{C} \right) \right)\sum_{k=1}^{K} {\left( k-1 \right)\gamma}_{k} \right]=1+\rho\sum_{k=1}^{K} {\left( k-1 \right)\gamma}_{k}$$

- - 1. Individual randomisation

# Applying property (P5) (Section 5):

$$\frac{1}{N}\sum_{i=1}^{M} t_{i}\left( t_{i}-1 \right) = \frac{1}{N}\sum_{k=1}^{K} \sum_{d=0}^{k} d\left( d-1 \right)\left( \frac{\binom{k}{d}}{2^{k}} \right)M_{k} = \sum_{k=1}^{K} \frac{M_{k}}{N2^{k}}\sum_{d=0}^{k} d\left( d-1 \right)\binom{k}{d} = \sum_{k=1}^{K} \frac{M_{k}}{N2^{k}}2^{k-2}\left( k-1 \right)k = \frac{1}{4}\sum_{k=1}^{K} \frac{{kM}_{k}}{N}(k-1) = \frac{1}{4}\sum_{k=1}^{K} \left( k-1 \right)\gamma_{k}$$

Applying property (P9) (Section 5):

$$\frac{1}{N}\sum_{i=1}^{M} c_{i}\left( c_{i}-1 \right) = \frac{1}{N}\sum_{k=1}^{K} \sum_{d=0}^{k} \left( k-d \right)\left( k-d-1 \right)\left( \frac{\binom{k}{d}}{2^{k}} \right)M_{k} = \sum_{k=1}^{K} \frac{M_{k}}{N2^{k}}\sum_{d=0}^{k} \left( k-d \right)\left( k-d-1 \right)\binom{k}{d} = \sum_{k=1}^{K} \frac{M_{k}}{N2^{k}}2^{k-2}\left( k-1 \right)k$$

$$= \frac{1}{4}\sum_{k=1}^{K} \frac{{kM}_{k}}{N}(k-1)=\frac{1}{4}\sum_{k=1}^{K} \left( k-1 \right)\gamma_{k}$$

Applying property (P8) (Section 5):

$$\frac{1}{N}\sum_{i=1}^{M} t_{i}c_{i}=\frac{1}{N}\sum_{k=1}^{K} \sum_{d=0}^{k} d\left( k-d \right)\left( \frac{\binom{k}{d}}{2^{k}} \right)M_{k} =\sum_{k=1}^{K} \frac{M_{k}}{N2^{k}}\sum_{d=0}^{k} d\left( k-d \right)\binom{k}{d} = \sum_{k=1}^{K} \frac{M_{k}}{N2^{k}}2^{k-2}\left( k-1 \right)k = \frac{1}{4}\sum_{k=1}^{K} \frac{{kM}_{k}}{N}(k-1) = \frac{1}{4}\sum_{k=1}^{K} \left( k-1 \right)\gamma_{k}$$

$$DEFF= 1+\rho\left[ \frac{2}{N\left( \pi_{I}\left( 1-\pi_{I} \right)+\pi_{C}\left( 1-\pi_{C} \right) \right)}\left( \pi_{C}\left( 1-\pi_{C} \right)\sum_{i=1}^{M} t_{i}\left( t_{i}-1 \right)+\pi_{I}\left( 1-\pi_{I} \right)\sum_{i=1}^{M} c_{i}\left( c_{i}-1 \right)-2\sqrt{\pi_{I}\pi_{C}\left( 1-\pi_{I} \right)\left( 1-\pi_{C} \right)}\sum_{i=1}^{M} t_{i}c_{i} \right) \right]$$

$$=1+\rho\left[ \frac{2}{\pi_{I}\left( 1-\pi_{I} \right)+\pi_{C}\left( 1-\pi_{C} \right)}\left( \pi_{C}\left( 1-\pi_{C} \right)\left( \frac{1}{4} \right)\sum_{k=1}^{K} \left( k-1 \right)\gamma_{k}+\pi_{I}\left( 1-\pi_{I} \right)\left( \frac{1}{4} \right)\sum_{k=1}^{K} \left( k-1 \right)\gamma_{k}-2\sqrt{\pi_{I}\pi_{C}\left( 1-\pi_{I} \right)\left( 1-\pi_{C} \right)}\left( \frac{1}{4} \right)\sum_{k=1}^{K} \left( k-1 \right)\gamma_{k} \right) \right]$$

$$=1+\rho\left[ \frac{1}{\pi_{I}\left( 1-\pi_{I} \right)+\pi_{C}\left( 1-\pi_{C} \right)}\left( \frac{1}{2}\left[ \pi_{C}\left( 1-\pi_{C} \right)+\pi_{I}\left( 1-\pi_{I} \right)-2\sqrt{\pi_{I}\pi_{C}\left( 1-\pi_{I} \right)\left( 1-\pi_{C} \right)} \right]\sum_{k=1}^{K} \left( k-1 \right)\gamma_{k} \right) \right]$$

$$=1+\rho\left[ \left( \frac{1}{2}-\frac{\sqrt{\pi_{I}\pi_{C}\left( 1-\pi_{I} \right)\left( 1-\pi_{C} \right)}}{\pi_{I}\left( 1-\pi_{I} \right)+\pi_{C}\left( 1-\pi_{C} \right)} \right)\sum_{k=1}^{K} \left( k-1 \right)\gamma_{k} \right]$$

- 1. **Exchangeable working correlation structure**

$$\mathrm{cov} \left( \hat{\boldsymbol{\beta}} \right)=\left[ \sum_{i=1}^{M} \mathbf{D}_{i}^{T}\mathbf{V}_{i}^{-1}\mathbf{D}_{i} \right]^{-1}$$

For the independent observations (i.e. $n_{i}=1 \left( j subscript dropped for convenience \right), X_{i}=0 \left( \mathrm{control} \right) \mathrm{or} 1\left( \mathrm{intervention} \right))$:

$\mathbf{R}_{i}=\mathbf{C}_{i}=1$, $\mathbf{A}_{i}^{1/2}=\sqrt{\mu_{i}(1-\mu_{i})}\Rightarrow$ $\mathbf{V}_{i}= \mathbf{A}_{i}^{1/2}{\mathbf{R}_{i}\mathbf{A}}_{i}^{1/2}=\mu_{i}(1-\mu_{i})\Rightarrow\mathbf{V}_{i}^{-1}=1/\mu_{i}(1-\mu_{i})$

$$\frac{\partial\mu_{i}}{\partial\beta_{0}}=\frac{\exp\left( \beta_{0}+\beta_{1}X_{i} \right)\left[ 1+{\exp(\beta}_{0}+\beta_{1}X_{i}) \right]-\left[ \exp\left( \beta_{0}+\beta_{1}X_{i} \right) \right]^{2}}{\left[ 1+{\exp(\beta}_{0}+\beta_{1}X_{i}) \right]^{2}} = \frac{\exp\left( \beta_{0}+\beta_{1}X_{i} \right)}{1+\exp\left( \beta_{0}+\beta_{1}X_{i} \right)}x\frac{1}{1+\exp\left( \beta_{0}+\beta_{1}X_{i} \right)} = \mu_{i}(1-\mu_{i})$$

$$\frac{\partial\mu_{i}}{\partial\beta_{1}}=\frac{X_{i}\exp\left( \beta_{0}+\beta_{1}X_{i} \right)\left[ 1+{\exp(\beta}_{0}+\beta_{1}X_{i}) \right]-X_{i}\left[ \exp\left( \beta_{0}+\beta_{1}X_{i} \right) \right]^{2}}{\left[ 1+{\exp(\beta}_{0}+\beta_{1}X_{i}) \right]^{2}} = {X_{i}\mu}_{i}(1-\mu_{i})$$

$$\Rightarrow\mathbf{D}_{i}=\left[ \begin{matrix} \frac{\partial\mu_{i}}{\partial\beta_{0}} & \frac{\partial\mu_{i}}{\partial\beta_{1}} \end{matrix} \right] = \left[ \begin{matrix} \mu_{i}(1-\mu_{i}) & {X_{i}\mu}_{i}(1-\mu_{i}) \end{matrix} \right]$$

$$\mathbf{D}_{i}^{T}\mathbf{V}_{i}^{-1}\mathbf{D}_{i}= \left[ \begin{aligned} \mu_{i}\left( 1-\mu_{i} \right) \\ {X_{i}\mu}_{i}\left( 1-\mu_{i} \right) \end{aligned} \right]\frac{1}{\mu_{i}\left( 1-\mu_{i} \right)}\left[ \begin{matrix} \mu_{i}\left( 1-\mu_{i} \right) & {X_{i}\mu}_{i}\left( 1-\mu_{i} \right) \end{matrix} \right] = \frac{1}{\mu_{i}\left( 1-\mu_{i} \right)}\left[ \begin{matrix} \left[ \mu_{i}\left( 1-\mu_{i} \right) \right]^{2} & X_{i}\left[ \mu_{i}\left( 1-\mu_{i} \right) \right]^{2} \\ X_{i}\left[ \mu_{i}\left( 1-\mu_{i} \right) \right]^{2} & X_{i}^{2}\left[ \mu_{i}\left( 1-\mu_{i} \right) \right]^{2} \end{matrix} \right]$$

$$=\left[ \begin{matrix} \mu_{i}\left( 1-\mu_{i} \right) & X_{i}\mu_{i}\left( 1-\mu_{i} \right) \\ X_{i}\mu_{i}\left( 1-\mu_{i} \right) & X_{i}^{2}\mu_{i}\left( 1-\mu_{i} \right) \end{matrix} \right]$$

For a cluster of size $n_{i}>1$with $t_{i}$ members assigned to the treatment group and $c_{i}$ members assigned to the control group:

$$\mathbf{R}_{i}=\mathbf{C}_{i}= \left[ \begin{matrix} 1 & \rho& \cdots& \rho\\ \rho& 1 & \ldots& \rho\\ \vdots& \vdots& \ddots& \vdots\\ \rho& \rho& \cdots& 1 \end{matrix} \right], a n_{i}\times n_{i}\mathrm{matrix}$$

$\mathbf{A}_{i}^{1/2}=\left[ \begin{matrix} \sqrt{\mu_{i1}(1-\mu_{i1})} & 0 & \cdots& 0 \\ 0 & \sqrt{\mu_{i2}(1-\mu_{i2})} & \ldots& 0 \\ \vdots& \vdots& \ddots& \vdots\\ 0 & 0 & \cdots& \sqrt{\mu_{in_{i}}(1-\mu_{{in}_{i}})} \end{matrix} \right]$ , a $n_{i}\times n_{i}$ matrix

$$\mathbf{V}_{i}= \mathbf{A}_{i}^{1/2}{\mathbf{R}_{i}\mathbf{A}}_{i}^{1/2}=\left[ \begin{matrix} \sqrt{\mu_{i1}\left( 1-\mu_{i1} \right)} & 0 & \cdots& 0 \\ 0 & \sqrt{\mu_{i2}\left( 1-\mu_{i2} \right)} & \ldots& 0 \\ \vdots& \vdots& \ddots& \vdots\\ 0 & 0 & \cdots& \sqrt{\mu_{{in}_{i}}\left( 1-\mu_{{in}_{i}} \right)} \end{matrix} \right]\left[ \begin{matrix} 1 & \rho& \cdots& \rho\\ \rho& 1 & \ldots& \rho\\ \vdots& \vdots& \ddots& \vdots\\ \rho& \rho& \cdots& 1 \end{matrix} \right]\left[ \begin{matrix} \sqrt{\mu_{i1}\left( 1-\mu_{i1} \right)} & 0 & \cdots& 0 \\ 0 & \sqrt{\mu_{i2}\left( 1-\mu_{i2} \right)} & \ldots& 0 \\ \vdots& \vdots& \ddots& \vdots\\ 0 & 0 & \cdots& \sqrt{\mu_{{in}_{i}}\left( 1-\mu_{{in}_{i}} \right)} \end{matrix} \right]$$

$$=\left[ \begin{matrix} \mu_{i1}\left( 1-\mu_{i1} \right) & \rho\sqrt{\mu_{i1}\mu_{i2}\left( 1-\mu_{i1} \right)\left( 1-\mu_{i2} \right)} & \cdots& \rho\sqrt{\mu_{i1}\mu_{{in}_{i}}\left( 1-\mu_{i1} \right)\left( 1-\mu_{{in}_{i}} \right)} \\ \rho\sqrt{\mu_{i1}\mu_{i2}\left( 1-\mu_{i1} \right)\left( 1-\mu_{i2} \right)} & \mu_{i2}\left( 1-\mu_{i2} \right) & \ldots& \rho\sqrt{\mu_{i2}\mu_{{in}_{i}}\left( 1-\mu_{i2} \right)\left( 1-\mu_{{in}_{i}} \right)} \\ \vdots& \vdots& \ddots& \vdots\\ \rho\sqrt{\mu_{i1}\mu_{in_{i}}\left( 1-\mu_{i1} \right)\left( 1-\mu_{{in}_{i}} \right)} & \rho\sqrt{\mu_{i2}\mu_{{in}_{i}}\left( 1-\mu_{i2} \right)\left( 1-\mu_{{in}_{i}} \right)} & \cdots& \mu_{in_{i}}\left( 1-\mu_{in_{i}} \right) \end{matrix} \right]$$

Using the properties of non-singular matrices, $\mathbf{V}_{i}^{\mathbf{-1}}= \left[ \mathbf{A}_{i}^{1/2}{\mathbf{R}_{i}\mathbf{A}}_{i}^{1/2} \right]^{-1}=\left[ \mathbf{A}_{i}^{1/2} \right]^{-1}\mathbf{R}_{i}^{-1}\left[ \mathbf{A}_{i}^{1/2} \right]^{-1}$

$$\left[ \mathbf{A}_{i}^{1/2} \right]^{-1}=\left[ \begin{matrix} \left[ \sqrt{\mu_{i1}\left( 1-\mu_{i1} \right)} \right]^{-1} & 0 & \cdots& 0 \\ 0 & \left[ \sqrt{\mu_{i2}\left( 1-\mu_{i2} \right)} \right]^{-1} & \ldots& 0 \\ \vdots& \vdots& \ddots& \vdots\\ 0 & 0 & \cdots& \left[ \sqrt{\mu_{{in}_{i}}\left( 1-\mu_{{in}_{i}} \right)} \right]^{-1} \end{matrix} \right]$$

Using the McDaniel et al (2013) expression for the inverse of an exchangeable matrix, $\mathbf{R}_{i}^{-1}$ can be written as:

$\mathbf{R}_{i}^{-1}=\left( a-b \right)\mathbf{I}_{n_{i}}+b\mathbf{J}_{n_{i}},$ where $a=\frac{1+\left( n_{i}-2 \right)\rho}{(1-\rho)(1+\left( n_{i}-1 \right)\rho)}$, $b=\frac{-\rho}{(1-\rho)(1+\left( n_{i}-1 \right)\rho)}$, $\mathbf{I}_{n_{i}}$ is the $n_{i}\times n_{i}$ identity matrix, and $\mathbf{J}_{n_{i}}$is the $n_{i}\times n_{i}$ matrix of 1s

$$\mathbf{R}_{i}^{-1}=\left( \frac{1+\left( n_{i}-2 \right)\rho}{\left( 1-\rho\right)\left( 1+\left( n_{i}-1 \right)\rho\right)}+\frac{\rho}{\left( 1-\rho\right)\left( 1+\left( n_{i}-1 \right)\rho\right)} \right)\mathbf{I}_{n_{i}}- \frac{\rho}{(1-\rho)(1+\left( n_{i}-1 \right)\rho}\mathbf{J}_{n_{i}}$$

$$= \frac{1}{\left( 1-\rho\right)\left( 1+\left( n_{i}-1 \right)\rho\right)}\left( (1+\left( n_{i}-1 \right)\rho)\left[ \begin{matrix} 1 & 0 & \cdots& 0 \\ 0 & 1 & \ldots& 0 \\ \vdots& \vdots& \ddots& \vdots\\ 0 & 0 & \cdots& 1 \end{matrix} \right]-\left[ \begin{matrix} \rho& \rho& \cdots& \rho\\ \rho& \rho& \ldots& \rho\\ \vdots& \vdots& \ddots& \vdots\\ \rho& \rho& \cdots& \rho\end{matrix} \right] \right)$$

$$= \frac{1}{\left( 1-\rho\right)\left( 1+\left( n_{i}-1 \right)\rho\right)}\left[ \begin{matrix} 1+\left( n_{i}-2 \right)\rho& -\rho& \cdots& -\rho\\ -\rho& 1+\left( n_{i}-2 \right)\rho& \ldots& -\rho\\ \vdots& \vdots& \ddots& \vdots\\ -\rho& -\rho& \cdots& 1+\left( n_{i}-2 \right)\rho\end{matrix} \right]$$

$$\Rightarrow\mathbf{V}_{i}^{-1}=\left[ \begin{matrix} \left[ \sqrt{\mu_{i1}\left( 1-\mu_{i1} \right)} \right]^{-1} & 0 & \cdots& 0 \\ 0 & \left[ \sqrt{\mu_{i2}\left( 1-\mu_{i2} \right)} \right]^{-1} & \ldots& 0 \\ \vdots& \vdots& \ddots& \vdots\\ 0 & 0 & \cdots& \left[ \sqrt{\mu_{{in}_{i}}\left( 1-\mu_{in_{i}} \right)} \right]^{-1} \end{matrix} \right]\left( \frac{1}{\left( 1-\rho\right)\left( 1+\left( n_{i}-1 \right)\rho\right)} \right)\times\left[ \begin{matrix} 1+\left( n_{i}-2 \right)\rho& -\rho& \cdots& -\rho\\ -\rho& 1+\left( n_{i}-2 \right)\rho& \ldots& -\rho\\ \vdots& \vdots& \ddots& \vdots\\ -\rho& -\rho& \cdots& 1+\left( n_{i}-2 \right)\rho\end{matrix} \right]\left[ \begin{matrix} \left[ \sqrt{\mu_{i1}\left( 1-\mu_{i1} \right)} \right]^{-1} & 0 & \cdots& 0 \\ 0 & \left[ \sqrt{\mu_{i2}\left( 1-\mu_{i2} \right)} \right]^{-1} & \ldots& 0 \\ \vdots& \vdots& \ddots& \vdots\\ 0 & 0 & \cdots& \left[ \sqrt{\mu_{in_{i}}\left( 1-\mu_{{in}_{i}} \right)} \right]^{-1} \end{matrix} \right]$$

$$=\frac{1}{\left( 1-\rho\right)\left( 1+\left( n_{i}-1 \right)\rho\right)}\left[ \begin{matrix} \frac{1+\left( n_{i}-2 \right)\rho}{\sqrt{\mu_{i1}\left( 1-\mu_{i1} \right)}} & \frac{-\rho}{\sqrt{\mu_{i1}\left( 1-\mu_{i1} \right)}} & \cdots& \frac{-\rho}{\sqrt{\mu_{i1}\left( 1-\mu_{i1} \right)}} \\ \frac{-\rho}{\sqrt{\mu_{i2}\left( 1-\mu_{i2} \right)}} & \frac{1+\left( n_{i}-2 \right)\rho}{\sqrt{\mu_{i2}\left( 1-\mu_{i2} \right)}} & \ldots& \frac{-\rho}{\sqrt{\mu_{i2}\left( 1-\mu_{i2} \right)}} \\ \vdots& \vdots& \ddots& \vdots\\ \frac{-\rho}{\sqrt{\mu_{{in}_{i}}\left( 1-\mu_{in_{i}} \right)}} & \frac{-\rho}{\sqrt{\mu_{{in}_{i}}\left( 1-\mu_{{in}_{i}} \right)}} & \cdots& \frac{1+\left( n_{i}-2 \right)\rho}{\sqrt{\mu_{{in}_{i}}\left( 1-\mu_{{in}_{i}} \right)}} \end{matrix} \right]\left[ \begin{matrix} \left[ \sqrt{\mu_{i1}\left( 1-\mu_{i1} \right)} \right]^{-1} & 0 & \cdots& 0 \\ 0 & \left[ \sqrt{\mu_{i2}\left( 1-\mu_{i2} \right)} \right]^{-1} & \ldots& 0 \\ \vdots& \vdots& \ddots& \vdots\\ 0 & 0 & \cdots& \left[ \sqrt{\mu_{{in}_{i}}\left( 1-\mu_{{in}_{i}} \right)} \right]^{-1} \end{matrix} \right]$$

$$= \frac{1}{\left( 1-\rho\right)\left( 1+\left( n_{i}-1 \right)\rho\right)}\left[ \begin{matrix} \frac{1+\left( n_{i}-2 \right)\rho}{\mu_{i1}\left( 1-\mu_{i1} \right)} & \frac{-\rho}{\sqrt{\mu_{i1}\mu_{i2}\left( 1-\mu_{i1} \right)\left( 1-\mu_{i2} \right)}} & \cdots& \frac{-\rho}{\sqrt{\mu_{i1}\mu_{in_{i}}\left( 1-\mu_{i1} \right)\left( 1-\mu_{in_{i}} \right)}} \\ \frac{-\rho}{\sqrt{\mu_{i1}\mu_{i2}\left( 1-\mu_{i1} \right)\left( 1-\mu_{i2} \right)}} & \frac{1+\left( n_{i}-2 \right)\rho}{\mu_{i2}\left( 1-\mu_{i2} \right)} & \ldots& \frac{-\rho}{\sqrt{\mu_{i2}\mu_{in_{i}}\left( 1-\mu_{i2} \right)\left( 1-\mu_{in_{i}} \right)}} \\ \vdots& \vdots& \ddots& \vdots\\ \frac{-\rho}{\sqrt{\mu_{i1}\mu_{in_{i}}\left( 1-\mu_{i1} \right)\left( 1-\mu_{{in}_{i}} \right)}} & \frac{-\rho}{\sqrt{\mu_{i2}\mu_{in_{i}}\left( 1-\mu_{i2} \right)\left( 1-\mu_{{in}_{i}} \right)}} & \cdots& \frac{1+\left( n_{i}-2 \right)\rho}{\mu_{{in}_{i}}\left( 1-\mu_{in_{i}} \right)} \end{matrix} \right]$$

$$\mathbf{D}_{i}=\left[ \begin{matrix} \begin{matrix} \frac{\partial\mu_{i1}}{\partial\beta_{0}} \end{matrix} & \frac{\partial\mu_{i1}}{\partial\beta_{1}} \\ \vdots& \vdots\\ \frac{\partial\mu_{in_{i}}}{\partial\beta_{0}} & \frac{\partial\mu_{{in}_{i}}}{\partial\beta_{1}} \end{matrix} \right]=\left[ \begin{matrix} \begin{matrix} \mu_{i1}\left( 1-\mu_{i1} \right) \end{matrix} & X_{i1}\mu_{i1}\left( 1-\mu_{i1} \right) \\ \vdots& \vdots\\ \mu_{{in}_{i}}\left( 1-\mu_{{in}_{i}} \right) & X_{{in}_{i}}\mu_{{in}_{i}}\left( 1-\mu_{{in}_{i}} \right) \end{matrix} \right]$$

$$\mathbf{D}_{i}^{T}\mathbf{V}_{i}^{-1}\mathbf{D}_{i}= \left[ \begin{matrix} \begin{matrix} \mu_{i1}\left( 1-\mu_{i1} \right) \end{matrix} & \ldots& \mu_{in_{i}}\left( 1-\mu_{in_{i}} \right) \\ X_{i1}\mu_{i1}\left( 1-\mu_{i1} \right) & \ldots& X_{in_{i}}\mu_{in_{i}}\left( 1-\mu_{in_{i}} \right) \end{matrix} \right]\left( \frac{1}{\left( 1-\rho\right)\left( 1+\left( n_{i}-1 \right)\rho\right)} \right)$$

$$\times\left[ \begin{matrix} \frac{1+\left( n_{i}-2 \right)\rho}{\mu_{i1}\left( 1-\mu_{i1} \right)} & \frac{-\rho}{\sqrt{\mu_{i1}\mu_{i2}\left( 1-\mu_{i1} \right)\left( 1-\mu_{i2} \right)}} & \cdots& \frac{-\rho}{\sqrt{\mu_{i1}\mu_{{in}_{i}}\left( 1-\mu_{1} \right)\left( 1-\mu_{n_{i}} \right)}} \\ \frac{-\rho}{\sqrt{\mu_{i1}\mu_{i2}\left( 1-\mu_{i1} \right)\left( 1-\mu_{i2} \right)}} & \frac{1+\left( n_{i}-2 \right)\rho}{\mu_{i2}\left( 1-\mu_{i2} \right)} & \ldots& \frac{-\rho}{\sqrt{\mu_{i2}\mu_{{in}_{i}}\left( 1-\mu_{i2} \right)\left( 1-\mu_{in_{i}} \right)}} \\ \vdots& \vdots& \ddots& \vdots\\ \frac{-\rho}{\sqrt{\mu_{i1}\mu_{{in}_{i}}\left( 1-\mu_{i1} \right)\left( 1-\mu_{{in}_{i}} \right)}} & \frac{-\rho}{\sqrt{\mu_{i2}\mu_{in_{i}}\left( 1-\mu_{i2} \right)\left( 1-\mu_{{in}_{i}} \right)}} & \cdots& \frac{1+\left( n_{i}-2 \right)\rho}{\mu_{{in}_{i}}\left( 1-\mu_{{in}_{i}} \right)} \end{matrix} \right]\left[ \begin{matrix} \begin{matrix} \mu_{i1}\left( 1-\mu_{i1} \right) \end{matrix} & X_{i1}\mu_{i1}\left( 1-\mu_{i1} \right) \\ \vdots& \vdots\\ \mu_{{in}_{i}}\left( 1-\mu_{{in}_{i}} \right) & X_{in_{i}}\mu_{in_{i}}\left( 1-\mu_{{in}_{i}} \right) \end{matrix} \right]$$

$$= \frac{1}{\left( 1-\rho\right)\left( 1+\left( n_{i}-1 \right)\rho\right)}\left[ \begin{matrix} 1+\left( n_{i}-1 \right)\rho-\rho\sum_{j=1}^{n_{i}} \frac{\mu_{ij}\left( 1-\mu_{ij} \right)}{\sqrt{\mu_{i1}\mu_{ij}\left( 1-\mu_{i1} \right)\left( 1-\mu_{ij} \right)}} & \cdots& 1+\left( n_{i}-1 \right)\rho-\rho\sum_{j=1}^{n_{i}} \frac{\mu_{ij}\left( 1-\mu_{ij} \right)}{\sqrt{\mu_{{in}_{i}}\mu_{ij}\left( 1-\mu_{{in}_{i}} \right)\left( 1-\mu_{ij} \right)}} \\ X_{1}\left( 1+\left( n_{i}-1 \right)\rho\right)-\rho\sum_{j=1}^{n_{i}} \frac{X_{j}\mu_{j}\left( 1-\mu_{j} \right)}{\sqrt{\mu_{1}\mu_{j}\left( 1-\mu_{1} \right)\left( 1-\mu_{j} \right)}} & \cdots& X_{n_{i}}\left( 1+\left( n_{i}-1 \right)\rho\right)-\rho\sum_{j=1}^{n_{i}} \frac{X_{ij}\mu_{ij}\left( 1-\mu_{ij} \right)}{\sqrt{\mu_{{in}_{i}}\mu_{ij}\left( 1-\mu_{{in}_{i}} \right)\left( 1-\mu_{ij} \right)}} \end{matrix} \right]$$

$$\times\left[ \begin{matrix} \begin{matrix} \mu_{i1}\left( 1-\mu_{i1} \right) \end{matrix} & X_{i1}\mu_{i1}\left( 1-\mu_{i1} \right) \\ \vdots& \vdots\\ \mu_{in_{i}}\left( 1-\mu_{in_{i}} \right) & X_{in_{i}}\mu_{in_{i}}\left( 1-\mu_{{in}_{i}} \right) \end{matrix} \right]$$

$$= \frac{1}{\left( 1-\rho\right)\left( 1+\left( n_{i}-1 \right)\rho\right)}\times\left[ \begin{matrix} \left( 1+\left( n_{i}-2 \right)\rho\right)\sum_{j=1}^{n_{i}} \begin{matrix} \mu_{ij}\left( 1-\mu_{ij} \right) \end{matrix}-2\rho\sum_{j=1}^{n_{i}} \sum_{a\neq j} \sqrt{\mu_{ij}\mu_{ia}\left( 1-\mu_{ij} \right)\left( 1-\mu_{ia} \right)} & \left( 1+\left( n_{i}-2 \right)\rho\right)\sum_{j=1}^{n_{i}} \begin{matrix} X_{ij}\mu_{ij}\left( 1-\mu_{ij} \right) \end{matrix}-\rho\sum_{j=1}^{n_{i}} \sum_{a\neq j} \left( X_{ij}+X_{ia} \right)\sqrt{\mu_{ij}\mu_{ia}\left( 1-\mu_{ij} \right)\left( 1-\mu_{ia} \right)} \\ \left( 1+\left( n_{i}-2 \right)\rho\right)\sum_{j=1}^{n_{i}} \begin{matrix} X_{ij}\mu_{ij}\left( 1-\mu_{ij} \right) \end{matrix}-\rho\sum_{j=1}^{n_{i}} \sum_{a\neq j} \left( X_{ij}+X_{ia} \right)\sqrt{\mu_{ij}\mu_{ia}\left( 1-\mu_{ij} \right)\left( 1-\mu_{ia} \right)} & \left( 1+\left( n_{i}-2 \right)\rho\right)\sum_{j=1}^{n_{i}} \begin{matrix} X_{ij}^{2}\mu_{ij}\left( 1-\mu_{ij} \right) \end{matrix}-2\rho\sum_{j=1}^{n_{i}} \sum_{a\neq j} X_{ij}X_{ia}\sqrt{\mu_{ij}\mu_{ia}\left( 1-\mu_{ij} \right)\left( 1-\mu_{ia} \right)} \end{matrix} \right]$$

Note that when $n_{i}=1$, this equals $\left[ \begin{matrix} \mu_{i1}(1-\mu_{i1}) & X_{i1}\mu_{i1}(1-\mu_{i1}) \\ X_{i1}\mu_{i1}(1-\mu_{i1}) & X_{i1}^{2}\mu_{i1}(1-\mu_{i1}) \end{matrix} \right]$ and therefore this expression for $\mathbf{D}_{i}^{T}\mathbf{V}_{i}^{-1}\mathbf{D}_{i}$ holds for all $n_{i}\geq1$.

The first sum in each element of this matrix can be written as the sum over cluster members assigned to intervention plus the sum over the cluster members assigned to control (e.g. $\sum_{j=1}^{n_{i}} \mu_{j}\left( 1-\mu_{j} \right)$ can be rewritten as $\sum_{j=1|X_{ij}=1}^{n_{i}} \mu_{j}\left( 1-\mu_{j} \right)+\sum_{j=1|X_{ij}=0}^{n_{i}} \mu_{j}\left( 1-\mu_{j} \right)$). A similar approach can be used for the double sums in each element of the matrix, by rewriting these as the sums of paired terms where the two cluster members are both assigned to intervention, both assigned to control, and one to intervention and one to control. For example, $\sum_{j=1}^{n_{i}} \sum_{a\neq j} \sqrt{\mu_{ij}\mu_{ia}\left( 1-\mu_{ij} \right)\left( 1-\mu_{ia} \right)}=\sum_{j=1|X_{ij}=1}^{n_{i}} \sum_{a\neq j|X_{ia}=1} \sqrt{\mu_{ij}\mu_{ia}\left( 1-\mu_{ij} \right)\left( 1-\mu_{ia} \right)}+\sum_{j=1|X_{ij}=0}^{n_{i}} \sum_{a\neq j|X_{ia}=0} \sqrt{\mu_{ij}\mu_{ia}\left( 1-\mu_{ij} \right)\left( 1-\mu_{ia} \right)}+\sum_{j=1|X_{ij}=1}^{n_{i}} \sum_{a\neq j|X_{ia}=0} \sqrt{\mu_{ij}\mu_{ia}\left( 1-\mu_{ij} \right)\left( 1-\mu_{ia} \right)}$. The sum over pairs where both are assigned to intervention is a sum of $\binom{t_{i}}{2}=t_{i}(t_{i}-1)/2$ terms, the sum over pairs where both are assigned to control is a sum of $\binom{c_{i}}{2}=c_{i}(c_{i}-1)/2$ terms, and the sum over pairs with one member assigned to intervention and one to control is a sum of $t_{i}c_{i}$ terms.

$\Rightarrow\mathbf{D}_{i}^{T}\mathbf{V}_{i}^{-1}\mathbf{D}_{i}=\frac{1}{\left( 1-\rho\right)\left( 1+\left( n_{i}-1 \right)\rho\right)} \left[ \begin{matrix} a & b \\ b & c \end{matrix} \right]$ , where

$$a=\left( 1+\left( n_{i}-2 \right)\rho\right)\sum_{j=1}^{n_{i}} \begin{matrix} \mu_{ij}\left( 1-\mu_{ij} \right) \end{matrix}-2\rho\sum_{j=1}^{n_{i}} \sum_{a\neq j} \sqrt{\mu_{ij}\mu_{ia}\left( 1-\mu_{ij} \right)\left( 1-\mu_{ia} \right)}$$

$$=\left( 1+\left( n_{i}-2 \right)\rho\right)\left( \pi_{I}\left( 1-\pi_{I} \right)t_{i}+\pi_{C}\left( 1-\pi_{C} \right)c_{i} \right)-2\rho\left[ \sqrt{\pi_{I}^{2}\left( 1-\pi_{I} \right)^{2}}\frac{t_{i}\left( t_{i}-1 \right)}{2}+\sqrt{\pi_{C}^{2}\left( 1-\pi_{C} \right)^{2}}\frac{c_{i}\left( c_{i}-1 \right)}{2}+\sqrt{\pi_{I}\pi_{C}\left( 1-\pi_{I} \right)\left( 1-\pi_{C} \right)}t_{i}c_{i} \right]$$

$$=\pi_{I}\left( 1-\pi_{I} \right)\left( 1+\left( n_{i}-t_{i}-1 \right)\rho\right)t_{i}+\pi_{C}\left( 1-\pi_{C} \right)\left( 1+\left( n_{i}-c_{i}-1 \right)\rho\right)c_{i}-2\sqrt{\pi_{I}\pi_{C}\left( 1-\pi_{I} \right)\left( 1-\pi_{C} \right)}\rho t_{i}c_{i}$$

$$=\pi_{I}\left( 1-\pi_{I} \right)\left( 1+\left( c_{i}-1 \right)\rho\right)t_{i}+\pi_{C}\left( 1-\pi_{C} \right)\left( 1+\left( t_{i}-1 \right)\rho\right)c_{i}-2\sqrt{\pi_{I}\pi_{C}\left( 1-\pi_{I} \right)\left( 1-\pi_{C} \right)}\rho t_{i}c_{i}$$

$$=\pi_{I}\left( 1-\pi_{I} \right)\left( 1-\rho\right)t_{i}+\pi_{C}\left( 1-\pi_{C} \right)\left( 1-\rho\right)c_{i}+\left[ \pi_{I}\left( 1-\pi_{I} \right)+\pi_{C}\left( 1-\pi_{C} \right)-2\sqrt{\pi_{I}\pi_{C}\left( 1-\pi_{I} \right)\left( 1-\pi_{C} \right)} \right] \rho t_{i}c_{i}$$

$$b=\left( 1+\left( n_{i}-2 \right)\rho\right)\sum_{j=1}^{n_{i}} \begin{matrix} X_{ij}\mu_{ij}\left( 1-\mu_{ij} \right) \end{matrix}-\rho\sum_{j=1}^{n_{i}} \sum_{a\neq j} \left( X_{ij}+X_{ia} \right)\sqrt{\mu_{ij}\mu_{ia}\left( 1-\mu_{ij} \right)\left( 1-\mu_{ia} \right)}$$

$$=\left( 1+\left( n_{i}-2 \right)\rho\right)\left( 1 \right)\pi_{I}\left( 1-\pi_{I} \right)t_{i}-\rho\left[ \left( 1+1 \right)\sqrt{\pi_{I}^{2}\left( 1-\pi_{I} \right)^{2}}\frac{t_{i}\left( t_{i}-1 \right)}{2}+\left( 1+0 \right)\sqrt{\pi_{I}\pi_{C}\left( 1-\pi_{I} \right)\left( 1-\pi_{C} \right)}t_{i}c_{i} \right]$$

$$=\left( 1+\left( n_{i}-2 \right)\rho\right)\pi_{I}\left( 1-\pi_{I} \right)t_{i}-\pi_{I}\left( 1-\pi_{I} \right)\rho t_{i}\left( t_{i}-1 \right)-\sqrt{\pi_{I}\pi_{C}\left( 1-\pi_{I} \right)\left( 1-\pi_{C} \right)}\rho t_{i}c_{i}$$

$$= \pi_{I}\left( 1-\pi_{I} \right)\left( 1+\left( c_{i}-1 \right)\rho\right)t_{i}-\sqrt{\pi_{I}\pi_{C}\left( 1-\pi_{I} \right)\left( 1-\pi_{C} \right)}\rho t_{i}c_{i} = \pi_{I}\left( 1-\pi_{I} \right)\left( 1-\rho\right)t_{i}+\left[ \pi_{I}\left( 1-\pi_{I} \right)-\sqrt{\pi_{I}\pi_{C}\left( 1-\pi_{I} \right)\left( 1-\pi_{C} \right)} \right]\rho t_{i}c_{i}$$

$$c=\left( 1+\left( n_{i}-2 \right)\rho\right)\sum_{j=1}^{n_{i}} \begin{matrix} X_{ij}^{2}\mu_{ij}\left( 1-\mu_{ij} \right) \end{matrix}-2\rho\sum_{j=1}^{n_{i}} \sum_{a\neq j} X_{ij}X_{ia}\sqrt{\mu_{ij}\mu_{ia}\left( 1-\mu_{ij} \right)\left( 1-\mu_{ia} \right)}$$

$$=\left( 1+\left( n_{i}-2 \right)\rho\right)\left( 1 \right)^{2}\pi_{I}\left( 1-\pi_{I} \right)t_{i}-2\rho\left[ \left( 1 \right)\left( 1 \right)\sqrt{\pi_{I}^{2}\left( 1-\pi_{I} \right)^{2}}\frac{t_{i}\left( t_{i}-1 \right)}{2} \right]$$

$$=\left( 1+\left( n_{i}-2 \right)\rho\right)\pi_{I}\left( 1-\pi_{I} \right)t_{i}-\pi_{I}\left( 1-\pi_{I} \right)\rho t_{i}\left( t_{i}-1 \right) = \pi_{I}\left( 1-\pi_{I} \right)\left( 1+\left( c_{i}-1 \right)\rho\right)t_{i} = \pi_{I}\left( 1-\pi_{I} \right)\left( 1-\rho\right)t_{i}+\pi_{I}\left( 1-\pi_{I} \right)\rho t_{i}c_{i}$$

Sum over all clusters $i=1,\ldots, M$. Cluster $i$ is of size $n_{i}$and contains $t_{i}$ members assigned to treatment and $c_{i}$ members assigned to control.

$\sum_{i=1}^{M} \mathbf{D}_{i}^{T}\mathbf{V}_{i}^{-1}\mathbf{D}_{i}= \sum_{i=1}^{M} \left[ \frac{1}{\left( 1-\rho\right)\left( 1+\left( n_{i}-1 \right)\rho\right)}\left[ \begin{matrix} a & b \\ b & c \end{matrix} \right] \right]= \left[ \begin{matrix} A & B \\ B & C \end{matrix} \right]$ , where

$$A=\sum_{i=1}^{M} \frac{1}{\left( 1-\rho\right)\left( 1+\left( n_{i}-1 \right)\rho\right)}\left[ \pi_{I}\left( 1-\pi_{I} \right)\left( 1-\rho\right)t_{i}+\pi_{C}\left( 1-\pi_{C} \right)\left( 1-\rho\right)c_{i}+\left[ \pi_{I}\left( 1-\pi_{I} \right)+\pi_{C}\left( 1-\pi_{C} \right)-2\sqrt{\pi_{I}\pi_{C}\left( 1-\pi_{I} \right)\left( 1-\pi_{C} \right)} \right] \rho t_{i}c_{i} \right]$$

$$=\pi_{I}\left( 1-\pi_{I} \right)\sum_{i=1}^{M} \frac{t_{i}}{\left( 1+\left( n_{i}-1 \right)\rho\right)}+\pi_{C}\left( 1-\pi_{C} \right)\sum_{i=1}^{M} \frac{c_{i}}{\left( 1+\left( n_{i}-1 \right)\rho\right)}+\left[ \pi_{I}\left( 1-\pi_{I} \right)+\pi_{C}\left( 1-\pi_{C} \right)-2\sqrt{\pi_{I}\pi_{C}\left( 1-\pi_{I} \right)\left( 1-\pi_{C} \right)} \right]\frac{\rho}{1-\rho}\sum_{i=1}^{M} \frac{{t_{i}c}_{i}}{\left( 1+\left( n_{i}-1 \right)\rho\right)}$$

$$B=\sum_{i=1}^{M} \frac{1}{\left( 1-\rho\right)\left( 1+\left( n_{i}-1 \right)\rho\right)}\left[ \pi_{I}\left( 1-\pi_{I} \right)\left( 1-\rho\right)t_{i}+\left[ \pi_{I}\left( 1-\pi_{I} \right)-\sqrt{\pi_{I}\pi_{C}\left( 1-\pi_{I} \right)\left( 1-\pi_{C} \right)} \right]\rho t_{i}c_{i} \right]$$

$$=\pi_{I}\left( 1-\pi_{I} \right)\sum_{i=1}^{M} \frac{t_{i}}{\left( 1+\left( n_{i}-1 \right)\rho\right)}+\left[ \pi_{I}\left( 1-\pi_{I} \right)-\sqrt{\pi_{I}\pi_{C}\left( 1-\pi_{I} \right)\left( 1-\pi_{C} \right)} \right]\frac{\rho}{1-\rho}\sum_{i=1}^{M} \frac{{t_{i}c}_{i}}{\left( 1+\left( n_{i}-1 \right)\rho\right)}$$

$$C=\sum_{i=1}^{M} \frac{1}{\left( 1-\rho\right)\left( 1+\left( n_{i}-1 \right)\rho\right)}\left[ \pi_{I}\left( 1-\pi_{I} \right)\left( 1-\rho\right)t_{i}+\pi_{I}\left( 1-\pi_{I} \right)\rho t_{i}c_{i} \right]$$

$$=\pi_{I}\left( 1-\pi_{I} \right)\sum_{i=1}^{M} \frac{t_{i}}{\left( 1+\left( n_{i}-1 \right)\rho\right)}+\pi_{I}\left( 1-\pi_{I} \right)\frac{\rho}{1-\rho}\sum_{i=1}^{M} \frac{{t_{i}c}_{i}}{\left( 1+\left( n_{i}-1 \right)\rho\right)}$$

$$\mathrm{cov} \left( \hat{\boldsymbol{\beta}} \right)=\left[ \sum_{i=1}^{M} \mathbf{D}_{i}^{T}\mathbf{V}_{i}^{-1}\mathbf{D}_{i} \right]^{-1} = \left[ \begin{matrix} A & B \\ B & C \end{matrix} \right]^{-1} = \frac{1}{AC-B^{2}}\left[ \begin{matrix} C & -B \\ -B & A \end{matrix} \right]$$

$$AC-B^{2}=\left[ \pi_{I}\left( 1-\pi_{I} \right)\sum_{i=1}^{M} \frac{t_{i}}{\left( 1+\left( n_{i}-1 \right)\rho\right)}+\pi_{C}\left( 1-\pi_{C} \right)\sum_{i=1}^{M} \frac{c_{i}}{\left( 1+\left( n_{i}-1 \right)\rho\right)}+\left[ \pi_{I}\left( 1-\pi_{I} \right)+\pi_{C}\left( 1-\pi_{C} \right)-2\sqrt{\pi_{I}\pi_{C}\left( 1-\pi_{I} \right)\left( 1-\pi_{C} \right)} \right]\frac{\rho}{1-\rho}\sum_{i=1}^{M} \frac{{t_{i}c}_{i}}{\left( 1+\left( n_{i}-1 \right)\rho\right)} \right]\times\left[ \pi_{I}\left( 1-\pi_{I} \right)\sum_{i=1}^{M} \frac{t_{i}}{\left( 1+\left( n_{i}-1 \right)\rho\right)}+\pi_{I}\left( 1-\pi_{I} \right)\frac{\rho}{1-\rho}\sum_{i=1}^{M} \frac{{t_{i}c}_{i}}{\left( 1+\left( n_{i}-1 \right)\rho\right)} \right]-\left[ \pi_{I}\left( 1-\pi_{I} \right)\sum_{i=1}^{M} \frac{t_{i}}{\left( 1+\left( n_{i}-1 \right)\rho\right)}+\left[ \pi_{I}\left( 1-\pi_{I} \right)-\sqrt{\pi_{I}\pi_{C}\left( 1-\pi_{I} \right)\left( 1-\pi_{C} \right)} \right]\frac{\rho}{1-\rho}\sum_{i=1}^{M} \frac{{t_{i}c}_{i}}{\left( 1+\left( n_{i}-1 \right)\rho\right)} \right]^{2}$$

$$=\pi_{I}\pi_{C}\left( 1-\pi_{I} \right)\left( 1-\pi_{C} \right)\sum_{i=1}^{M} \frac{t_{i}}{\left( 1+\left( n_{i}-1 \right)\rho\right)}\sum_{i=1}^{M} \frac{c_{i}}{\left( 1+\left( n_{i}-1 \right)\rho\right)}+\pi_{I}\pi_{C}\left( 1-\pi_{I} \right)\left( 1-\pi_{C} \right)\frac{\rho}{1-\rho}\sum_{i=1}^{M} \frac{c_{i}}{\left( 1+\left( n_{i}-1 \right)\rho\right)}\sum_{i=1}^{M} \frac{{t_{i}c}_{i}}{\left( 1+\left( n_{i}-1 \right)\rho\right)}+\left[ \pi_{I}^{2}\left( 1-\pi_{I} \right)^{2}+\pi_{I}\pi_{C}\left( 1-\pi_{I} \right)\left( 1-\pi_{C} \right)+\pi_{I}^{2}\left( 1-\pi_{I} \right)^{2}-2\pi_{I}\left( 1-\pi_{I} \right)\sqrt{\pi_{I}\pi_{C}\left( 1-\pi_{I} \right)\left( 1-\pi_{C} \right)}-2\pi_{I}^{2}\left( 1-\pi_{I} \right)^{2}+2\pi_{I}\left( 1-\pi_{I} \right)\sqrt{\pi_{I}\pi_{C}\left( 1-\pi_{I} \right)\left( 1-\pi_{C} \right)} \right]\frac{\rho}{1-\rho}\sum_{i=1}^{M} \frac{t_{i}}{\left( 1+\left( n_{i}-1 \right)\rho\right)}\sum_{i=1}^{M} \frac{{t_{i}c}_{i}}{\left( 1+\left( n_{i}-1 \right)\rho\right)}+\left[ \pi_{I}^{2}\left( 1-\pi_{I} \right)^{2}+\pi_{I}\pi_{C}\left( 1-\pi_{I} \right)\left( 1-\pi_{C} \right)-2\pi_{I}\left( 1-\pi_{I} \right)\sqrt{\pi_{I}\pi_{C}\left( 1-\pi_{I} \right)\left( 1-\pi_{C} \right)}-\pi_{I}^{2}\left( 1-\pi_{I} \right)^{2}+2\pi_{I}\left( 1-\pi_{I} \right)\sqrt{\pi_{I}\pi_{C}\left( 1-\pi_{I} \right)\left( 1-\pi_{C} \right)}-\pi_{I}\pi_{C}\left( 1-\pi_{I} \right)\left( 1-\pi_{C} \right) \right]\frac{\rho^{2}}{\left( 1-\rho\right)^{2}}\left[ \sum_{i=1}^{M} \frac{{t_{i}c}_{i}}{\left( 1+\left( n_{i}-1 \right)\rho\right)} \right]^{2}$$

$$=\pi_{I}\pi_{C}\left( 1-\pi_{I} \right)\left( 1-\pi_{C} \right)\left[ \sum_{i=1}^{M} \frac{t_{i}}{\left( 1+\left( n_{i}-1 \right)\rho\right)}\sum_{i=1}^{M} \frac{c_{i}}{\left( 1+\left( n_{i}-1 \right)\rho\right)}+\frac{\rho}{1-\rho}\sum_{i=1}^{M} \frac{t_{i}}{\left( 1+\left( n_{i}-1 \right)\rho\right)}\sum_{i=1}^{M} \frac{{t_{i}c}_{i}}{\left( 1+\left( n_{i}-1 \right)\rho\right)}+\frac{\rho}{1-\rho}\sum_{i=1}^{M} \frac{c_{i}}{\left( 1+\left( n_{i}-1 \right)\rho\right)}\sum_{i=1}^{M} \frac{{t_{i}c}_{i}}{\left( 1+\left( n_{i}-1 \right)\rho\right)} \right]$$

$$\Rightarrow\mathrm{cov} \left( \hat{\boldsymbol{\beta}} \right)=$$

$$\frac{1}{\pi_{I}\pi_{C}\left( 1-\pi_{I} \right)\left( 1-\pi_{C} \right)\left[ \sum_{i=1}^{M} \frac{t_{i}}{\left( 1+\left( n_{i}-1 \right)\rho\right)}\sum_{i=1}^{M} \frac{c_{i}}{\left( 1+\left( n_{i}-1 \right)\rho\right)}+\frac{\rho}{1-\rho}\sum_{i=1}^{M} \frac{t_{i}}{\left( 1+\left( n_{i}-1 \right)\rho\right)}\sum_{i=1}^{M} \frac{{t_{i}c}_{i}}{\left( 1+\left( n_{i}-1 \right)\rho\right)}+\frac{\rho}{1-\rho}\sum_{i=1}^{M} \frac{c_{i}}{\left( 1+\left( n_{i}-1 \right)\rho\right)}\sum_{i=1}^{M} \frac{{t_{i}c}_{i}}{\left( 1+\left( n_{i}-1 \right)\rho\right)} \right]}\times\left[ \begin{matrix} C & -B \\ -B & A \end{matrix} \right]$$

$$\mathrm{var}\left( \hat{\beta}_{1} \right)=$$

$$\frac{\pi_{I}\left( 1-\pi_{I} \right)\sum_{i=1}^{M} \frac{t_{i}}{\left( 1+\left( n_{i}-1 \right)\rho\right)}+\pi_{C}\left( 1-\pi_{C} \right)\sum_{i=1}^{M} \frac{c_{i}}{\left( 1+\left( n_{i}-1 \right)\rho\right)}+\left[ \pi_{I}\left( 1-\pi_{I} \right)+\pi_{C}\left( 1-\pi_{C} \right)-2\sqrt{\pi_{I}\pi_{C}\left( 1-\pi_{I} \right)\left( 1-\pi_{C} \right)} \right]\frac{\rho}{1-\rho}\sum_{i=1}^{M} \frac{{t_{i}c}_{i}}{\left( 1+\left( n_{i}-1 \right)\rho\right)}}{\pi_{I}\pi_{C}\left( 1-\pi_{I} \right)\left( 1-\pi_{C} \right)\left[ \sum_{i=1}^{M} \frac{t_{i}}{\left( 1+\left( n_{i}-1 \right)\rho\right)}\sum_{i=1}^{M} \frac{c_{i}}{\left( 1+\left( n_{i}-1 \right)\rho\right)}+\frac{\rho}{1-\rho}\sum_{i=1}^{M} \frac{t_{i}}{\left( 1+\left( n_{i}-1 \right)\rho\right)}\sum_{i=1}^{M} \frac{{t_{i}c}_{i}}{\left( 1+\left( n_{i}-1 \right)\rho\right)}+\frac{\rho}{1-\rho}\sum_{i=1}^{M} \frac{c_{i}}{\left( 1+\left( n_{i}-1 \right)\rho\right)}\sum_{i=1}^{M} \frac{{t_{i}c}_{i}}{\left( 1+\left( n_{i}-1 \right)\rho\right)} \right]}$$

$$\Rightarrow DEFF=$$

$$\frac{\pi_{I}\left( 1-\pi_{I} \right)\sum_{i=1}^{M} \frac{t_{i}}{\left( 1+\left( n_{i}-1 \right)\rho\right)}+\pi_{C}\left( 1-\pi_{C} \right)\sum_{i=1}^{M} \frac{c_{i}}{\left( 1+\left( n_{i}-1 \right)\rho\right)}+\left[ \pi_{I}\left( 1-\pi_{I} \right)+\pi_{C}\left( 1-\pi_{C} \right)-2\sqrt{\pi_{I}\pi_{C}\left( 1-\pi_{I} \right)\left( 1-\pi_{C} \right)} \right]\frac{\rho}{1-\rho}\sum_{i=1}^{M} \frac{{t_{i}c}_{i}}{\left( 1+\left( n_{i}-1 \right)\rho\right)}}{\pi_{I}\pi_{C}\left( 1-\pi_{I} \right)\left( 1-\pi_{C} \right)\left[ \sum_{i=1}^{M} \frac{t_{i}}{\left( 1+\left( n_{i}-1 \right)\rho\right)}\sum_{i=1}^{M} \frac{c_{i}}{\left( 1+\left( n_{i}-1 \right)\rho\right)}+\frac{\rho}{1-\rho}\sum_{i=1}^{M} \frac{t_{i}}{\left( 1+\left( n_{i}-1 \right)\rho\right)}\sum_{i=1}^{M} \frac{{t_{i}c}_{i}}{\left( 1+\left( n_{i}-1 \right)\rho\right)}+\frac{\rho}{1-\rho}\sum_{i=1}^{M} \frac{c_{i}}{\left( 1+\left( n_{i}-1 \right)\rho\right)}\sum_{i=1}^{M} \frac{{t_{i}c}_{i}}{\left( 1+\left( n_{i}-1 \right)\rho\right)} \right]} \times\frac{N\pi_{I}\pi_{C}\left( 1-\pi_{I} \right)\left( 1-\pi_{C} \right)}{2\left( \pi_{I}\left( 1-\pi_{I} \right)+\pi_{C}\left( 1-\pi_{C} \right) \right)}$$

$$=\frac{\pi_{I}\left( 1-\pi_{I} \right)\sum_{i=1}^{M} \frac{t_{i}}{\left( 1+\left( n_{i}-1 \right)\rho\right)}+\pi_{C}\left( 1-\pi_{C} \right)\sum_{i=1}^{M} \frac{c_{i}}{\left( 1+\left( n_{i}-1 \right)\rho\right)}+\left[ \pi_{I}\left( 1-\pi_{I} \right)+\pi_{C}\left( 1-\pi_{C} \right)-2\sqrt{\pi_{I}\pi_{C}\left( 1-\pi_{I} \right)\left( 1-\pi_{C} \right)} \right]\frac{\rho}{1-\rho}\sum_{i=1}^{M} \frac{{t_{i}c}_{i}}{\left( 1+\left( n_{i}-1 \right)\rho\right)}}{\sum_{i=1}^{M} \frac{t_{i}}{\left( 1+\left( n_{i}-1 \right)\rho\right)}\sum_{i=1}^{M} \frac{c_{i}}{\left( 1+\left( n_{i}-1 \right)\rho\right)}+\frac{\rho}{1-\rho}\sum_{i=1}^{M} \frac{t_{i}}{\left( 1+\left( n_{i}-1 \right)\rho\right)}\sum_{i=1}^{M} \frac{{t_{i}c}_{i}}{\left( 1+\left( n_{i}-1 \right)\rho\right)}+\frac{\rho}{1-\rho}\sum_{i=1}^{M} \frac{c_{i}}{\left( 1+\left( n_{i}-1 \right)\rho\right)}\sum_{i=1}^{M} \frac{{t_{i}c}_{i}}{\left( 1+\left( n_{i}-1 \right)\rho\right)}} \times\frac{N}{2\left( \pi_{I}\left( 1-\pi_{I} \right)+\pi_{C}\left( 1-\pi_{C} \right) \right)}$$

- - 1. Cluster randomisation

$$\sum_{i=1}^{M} \frac{t_{i}}{\left( 1+\left( n_{i}-1 \right)\rho\right)} = \sum_{k=1}^{K} \left[ \left( \frac{M_{k}}{2} \right)\frac{k}{\left( 1+\left( k-1 \right)\rho\right)}+\left( \frac{M_{k}}{2} \right)\frac{0}{\left( 1+\left( k-1 \right)\rho\right)} \right] = \frac{N}{2}\sum_{k=1}^{K} \frac{kM_{k}}{N\left( 1+\left( k-1 \right)\rho\right)} = \frac{N}{2}\sum_{k=1}^{K} {\frac{1}{\left( 1+\left( k-1 \right)\rho\right)}\gamma}_{k}$$

$$\sum_{i=1}^{M} \frac{c_{i}}{\left( 1+\left( n_{i}-1 \right)\rho\right)} = \sum_{k=1}^{K} \left[ \left( \frac{M_{k}}{2} \right)\frac{0}{\left( 1+\left( k-1 \right)\rho\right)}+\left( \frac{M_{k}}{2} \right)\frac{k}{\left( 1+\left( k-1 \right)\rho\right)} \right] = \frac{N}{2}\sum_{k=1}^{K} \frac{kM_{k}}{N\left( 1+\left( k-1 \right)\rho\right)} = \frac{N}{2}\sum_{k=1}^{K} {\frac{1}{\left( 1+\left( k-1 \right)\rho\right)}\gamma}_{k}$$

$$\sum_{i=1}^{M} \frac{{t_{i}c}_{i}}{\left( 1+\left( n_{i}-1 \right)\rho\right)} = \sum_{k=1}^{K} \left[ \left( \frac{M_{k}}{2} \right)\frac{k(0)}{\left( 1+\left( k-1 \right)\rho\right)}+\left( \frac{M_{k}}{2} \right)\frac{\left( 0 \right)k}{\left( 1+\left( k-1 \right)\rho\right)} \right] = 0$$

$$DEFF= \frac{\pi_{I}\left( 1-\pi_{I} \right)\frac{N}{2}\left( \sum_{k=1}^{K} {\frac{1}{\left( 1+\left( k-1 \right)\rho\right)}\gamma}_{k} \right)+\pi_{C}\left( 1-\pi_{C} \right)\left( \frac{N}{2}\sum_{k=1}^{K} {\frac{1}{\left( 1+\left( k-1 \right)\rho\right)}\gamma}_{k} \right)}{\left( \frac{N}{2}\sum_{k=1}^{K} {\frac{1}{\left( 1+\left( k-1 \right)\rho\right)}\gamma}_{k} \right)^{2}} \times\frac{N}{2\left( \pi_{I}\left( 1-\pi_{I} \right)+\pi_{C}\left( 1-\pi_{C} \right) \right)}$$

$$=\frac{\left( \pi_{I}\left( 1-\pi_{I} \right)+\pi_{C}\left( 1-\pi_{C} \right) \right)\left( \sum_{k=1}^{K} {\frac{1}{\left( 1+\left( k-1 \right)\rho\right)}\gamma}_{k} \right)}{\left( \sum_{k=1}^{K} {\frac{1}{\left( 1+\left( k-1 \right)\rho\right)}\gamma}_{k} \right)^{2}} \times\frac{1}{\left( \pi_{I}\left( 1-\pi_{I} \right)+\pi_{C}\left( 1-\pi_{C} \right) \right)}=\frac{1}{\sum_{k=1}^{K} {\frac{1}{\left( 1+\left( k-1 \right)\rho\right)}\gamma}_{k}} = \left[ \sum_{k=1}^{K} {\frac{1}{\left( 1+\left( k-1 \right)\rho\right)}\gamma}_{k} \right]^{-1}$$

- - 1. Individual randomisation

# Applying property (P4) (Section 5):

$$\sum_{i=1}^{M} \frac{t_{i}}{\left( 1+\left( n_{i}-1 \right)\rho\right)} = \sum_{k=1}^{K} \sum_{d=0}^{k} \frac{d}{\left( 1+\left( k-1 \right)\rho\right)}\left( \frac{\binom{k}{d}}{2^{k}} \right)M_{k} = \sum_{k=1}^{K} \frac{1}{\left( 1+\left( k-1 \right)\rho\right)}\left( \frac{M_{k}}{2^{k}} \right)\sum_{d=0}^{k} d\binom{k}{d} = \sum_{k=1}^{K} \frac{1}{\left( 1+\left( k-1 \right)\rho\right)}\left( \frac{M_{k}}{2^{k}} \right)2^{k-1}k$$

$$=\frac{N}{2}\sum_{k=1}^{K} \frac{1}{\left( 1+\left( k-1 \right)\rho\right)}\left( \frac{{kM}_{k}}{N} \right)=\frac{N}{2}\sum_{k=1}^{K} \frac{1}{\left( 1+\left( k-1 \right)\rho\right)}\gamma_{k}$$

Applying property (P7) (Section 5):

$$\sum_{i=1}^{M} \frac{c_{i}}{\left( 1+\left( n_{i}-1 \right)\rho\right)} = \sum_{k=1}^{K} \sum_{d=0}^{k} \frac{k-d}{\left( 1+\left( k-1 \right)\rho\right)}\left( \frac{\binom{k}{d}}{2^{k}} \right)M_{k} = \sum_{k=1}^{K} \frac{1}{\left( 1+\left( k-1 \right)\rho\right)}\left( \frac{M_{k}}{2^{k}} \right)\sum_{d=0}^{k} \left( k-d \right)\binom{k}{d} = \sum_{k=1}^{K} \frac{1}{\left( 1+\left( k-1 \right)\rho\right)}\left( \frac{M_{k}}{2^{k}} \right)2^{k-1}k$$

$$=\frac{N}{2}\sum_{k=1}^{K} \frac{1}{\left( 1+\left( k-1 \right)\rho\right)}\left( \frac{{kM}_{k}}{N} \right) = \frac{N}{2}\sum_{k=1}^{K} \frac{1}{\left( 1+\left( k-1 \right)\rho\right)}\gamma_{k}$$

Applying property (P8) (Section 5):

$$\sum_{i=1}^{M} \frac{t_{i}c_{i}}{\left( 1+\left( n_{i}-1 \right)\rho\right)} = \sum_{k=1}^{K} \sum_{d=0}^{k} \frac{d\left( k-d \right)}{\left( 1+\left( k-1 \right)\rho\right)}\left( \frac{\binom{k}{d}}{2^{k}} \right)M_{k} = \sum_{k=1}^{K} \frac{1}{\left( 1+\left( k-1 \right)\rho\right)}\left( \frac{M_{k}}{2^{k}} \right)\sum_{d=0}^{k} d\left( k-d \right)\binom{k}{d} = \sum_{k=1}^{K} \frac{1}{\left( 1+\left( k-1 \right)\rho\right)}\left( \frac{M_{k}}{2^{k}} \right)2^{k-2}k\left( k-1 \right)$$

$$= \frac{N}{4}\sum_{k=1}^{K} \frac{k-1}{\left( 1+\left( k-1 \right)\rho\right)}\left( \frac{{kM}_{k}}{N} \right)=\frac{N}{4}\sum_{k=1}^{K} \frac{k-1}{\left( 1+\left( k-1 \right)\rho\right)}\gamma_{k}$$

$$DEFF=$$

$$\frac{\pi_{I}\left( 1-\pi_{I} \right)\left( \frac{N}{2} \right)\sum_{k=1}^{K} \frac{1}{\left( 1+\left( k-1 \right)\rho\right)}\gamma_{k}+\pi_{C}\left( 1-\pi_{C} \right)\left( \frac{N}{2} \right)\sum_{k=1}^{K} \frac{1}{\left( 1+\left( k-1 \right)\rho\right)}\gamma_{k}+\left[ \pi_{I}\left( 1-\pi_{I} \right)+\pi_{C}\left( 1-\pi_{C} \right)-2\sqrt{\pi_{I}\pi_{C}\left( 1-\pi_{I} \right)\left( 1-\pi_{C} \right)} \right]\frac{\rho}{1-\rho}\left( \frac{N}{4} \right)\sum_{k=1}^{K} \frac{k-1}{\left( 1+\left( k-1 \right)\rho\right)}\gamma_{k}}{\left( \frac{N}{2}\sum_{k=1}^{K} \frac{1}{\left( 1+\left( k-1 \right)\rho\right)}\gamma_{k} \right)^{2}+2\left( \frac{\rho}{1-\rho}\left( \frac{N}{2} \right)\sum_{k=1}^{K} \frac{1}{\left( 1+\left( k-1 \right)\rho\right)}\gamma_{k}\left( \frac{N}{4} \right)\sum_{k=1}^{K} \frac{k-1}{\left( 1+\left( k-1 \right)\rho\right)}\gamma_{k} \right)} \times\frac{N}{2\left( \pi_{I}\left( 1-\pi_{I} \right)+\pi_{C}\left( 1-\pi_{C} \right) \right)}$$

$$=\frac{\left( \pi_{I}\left( 1-\pi_{I} \right)+\pi_{C}\left( 1-\pi_{C} \right) \right)\sum_{k=1}^{K} \frac{1}{\left( 1+\left( k-1 \right)\rho\right)}\gamma_{k}+\frac{1}{2}\left[ \pi_{I}\left( 1-\pi_{I} \right)+\pi_{C}\left( 1-\pi_{C} \right)-2\sqrt{\pi_{I}\pi_{C}\left( 1-\pi_{I} \right)\left( 1-\pi_{C} \right)} \right]\left( \frac{\rho}{1-\rho} \right)\left( \sum_{k=1}^{K} \frac{k-1}{\left( 1+\left( k-1 \right)\rho\right)}\gamma_{k} \right)}{\left( \pi_{I}\left( 1-\pi_{I} \right)+\pi_{C}\left( 1-\pi_{C} \right) \right)\left( \sum_{k=1}^{K} \frac{1}{\left( 1+\left( k-1 \right)\rho\right)}\gamma_{k} \right)^{2}+\left( \frac{\rho}{1-\rho} \right)\left( \sum_{k=1}^{K} \frac{1}{\left( 1+\left( k-1 \right)\rho\right)}\gamma_{k} \right)\left( \sum_{k=1}^{K} \frac{k-1}{\left( 1+\left( k-1 \right)\rho\right)}\gamma_{k} \right)}$$

$$=\frac{\sum_{k=1}^{K} \frac{1}{\left( 1+\left( k-1 \right)\rho\right)}\gamma_{k}+\left( \frac{1}{2}-\frac{\sqrt{\pi_{I}\pi_{C}\left( 1-\pi_{I} \right)\left( 1-\pi_{C} \right)}}{\pi_{I}\left( 1-\pi_{I} \right)+\pi_{C}\left( 1-\pi_{C} \right)} \right)\left( \frac{\rho}{1-\rho} \right)\left( \sum_{k=1}^{K} \frac{k-1}{\left( 1+\left( k-1 \right)\rho\right)}\gamma_{k} \right)}{\left( \sum_{k=1}^{K} \frac{1}{\left( 1+\left( k-1 \right)\rho\right)}\gamma_{k} \right)^{2}+\left( \frac{\rho}{1-\rho} \right)\left( \sum_{k=1}^{K} \frac{1}{\left( 1+\left( k-1 \right)\rho\right)}\gamma_{k} \right)\left( \sum_{k=1}^{K} \frac{k-1}{\left( 1+\left( k-1 \right)\rho\right)}\gamma_{k} \right)}$$

# DESIGN EFFECTS FOR A BINARY OUTCOME WITH A LOG LINK

The variance of the treatment effect estimate from a generalised linear model for independent data, using the multivariate delta method, is ${\mathrm{var}\left( \hat{\beta}_{1} \right)}_{IND}=\frac{1-\pi_{I}}{N_{I}\pi_{I}}+\frac{1-\pi_{C}}{N_{C}\pi_{C}}$ , which equals $\frac{2(\pi_{C}\left( 1-\pi_{I} \right)+\pi_{I}\left( 1-\pi_{C} \right))}{N\pi_{I}\pi_{C}}$ under the assumption of overall treatment balance. This will be used as the denominator in the DEFF (Equation 3 in the main paper).

- 1. **Independence working correlation structure**

$$\mathrm{cov} \left( \hat{\boldsymbol{\beta}} \right)=\left[ \sum_{i=1}^{M} {\mathbf{D}_{i}}^{T}{\mathbf{V}_{i}}^{-1}\mathbf{D}_{i} \right]^{-1}\left[ \sum_{i=1}^{M} {\mathbf{D}_{i}}^{T}{\mathbf{V}_{i}}^{-1}\mathrm{cov} \left( \mathbf{Y}_{i} \right){\mathbf{V}_{i}}^{-1}\mathbf{D}_{i} \right]\left[ \sum_{i=1}^{M} {\mathbf{D}_{i}}^{T}{\mathbf{V}_{i}}^{-1}\mathbf{D}_{i} \right]^{-1}$$

For the independent observations (i.e. $n_{i}=1$ ($j$ subscript dropped for convenience), $X_{i}=0 \left( \mathrm{control} \right)\mathrm{or} 1\left( \mathrm{intervention} \right))$:

$\mathbf{R}_{i}=1$, $\mathbf{A}_{i}^{1/2}=\sqrt{\mu_{i}(1-\mu_{i})}\Rightarrow$ $\mathbf{V}_{i}= \mathbf{A}_{i}^{1/2}{\mathbf{R}_{i}\mathbf{A}}_{i}^{1/2}=\mu_{i}(1-\mu_{i})\Rightarrow\mathbf{V}_{i}^{-1}=1/\mu_{i}(1-\mu_{i})$

$\mathbf{C}_{i}=1$, $\mathbf{A}_{i}^{1/2}=\sqrt{\mu_{i}(1-\mu_{i})}\Rightarrow\mathrm{cov}\left( \mathbf{Y}_{i} \right)=$ $\mathbf{A}_{i}^{1/2}{\mathbf{C}_{i}\mathbf{A}}_{i}^{1/2}=\mu_{i}(1-\mu_{i})$

$$\frac{\partial\mu_{i}}{\partial\beta_{0}}= {\exp\left( \beta_{0}+\beta_{1}X_{i} \right)=\mu}_{i}$$

$$\frac{\partial\mu_{i}}{\partial\beta_{1}}= X_{i}{{\exp\left( \beta_{0}+\beta_{1}X_{i} \right)=X}_{i}\mu}_{i}$$

$$\Rightarrow\mathbf{D}_{i}=\left[ \begin{matrix} \frac{\partial\mu_{i}}{\partial\beta_{0}} & \frac{\partial\mu_{i}}{\partial\beta_{1}} \end{matrix} \right]=\left[ \begin{matrix} \mu_{i} & {X_{i}\mu}_{i} \end{matrix} \right]$$

$$\mathbf{D}_{i}^{T}\mathbf{V}_{i}^{-1}\mathbf{D}_{i}= \left[ \begin{matrix} \mu_{i} \\ {X_{i}\mu}_{i} \end{matrix} \right]\frac{1}{\mu_{i}\left( 1-\mu_{i} \right)}\left[ \begin{matrix} \mu_{i} & {X_{i}\mu}_{i} \end{matrix} \right] = \frac{1}{\mu_{i}\left( 1-\mu_{i} \right)}\left[ \begin{matrix} {\mu_{i}}^{2} & X_{i}{\mu_{i}}^{2} \\ X_{i}{\mu_{i}}^{2} & X_{i}^{2}{\mu_{i}}^{2} \end{matrix} \right] = \left[ \begin{matrix} \frac{\mu_{i}}{1-\mu_{i}} & \frac{X_{i}\mu_{i}}{1-\mu_{i}} \\ \frac{X_{i}\mu_{i}}{1-\mu_{i}} & \frac{X_{i}^{2}\mu_{i}}{1-\mu_{i}} \end{matrix} \right]$$

$$\mathbf{D}_{i}^{T}\mathbf{V}_{i}^{-1}{\mathrm{cov}\left( \mathbf{Y}_{i} \right)\mathbf{V}_{i}^{-1}\mathbf{D}}_{i}= \left[ \begin{matrix} \mu_{i} \\ {X_{i}\mu}_{i} \end{matrix} \right]\frac{1}{\mu_{i}\left( 1-\mu_{i} \right)}\mu_{i}\left( 1-\mu_{i} \right)\frac{1}{\mu_{i}\left( 1-\mu_{i} \right)}\left[ \begin{matrix} \mu_{i} & {X_{i}\mu}_{i} \end{matrix} \right] = \frac{1}{\mu_{i}\left( 1-\mu_{i} \right)}\left[ \begin{matrix} {\mu_{i}}^{2} & X_{i}{\mu_{i}}^{2} \\ X_{i}{\mu_{i}}^{2} & X_{i}^{2}{\mu_{i}}^{2} \end{matrix} \right] = \left[ \begin{matrix} \frac{\mu_{i}}{1-\mu_{i}} & \frac{X_{i}\mu_{i}}{1-\mu_{i}} \\ \frac{X_{i}\mu_{i}}{1-\mu_{i}} & \frac{X_{i}^{2}\mu_{i}}{1-\mu_{i}} \end{matrix} \right]$$

For a cluster of size $n_{i}>1$ with $t_{i}$ members assigned to the treatment group and $c_{i}$ members assigned to the control group:

$$\mathbf{R}_{i}=\mathbf{I}_{n_{i}}$$

$\mathbf{A}_{i}^{1/2}=\left[ \begin{matrix} \sqrt{\mu_{i1}(1-\mu_{i1})} & 0 & \cdots& 0 \\ 0 & \sqrt{\mu_{i2}(1-\mu_{i2})} & \ldots& 0 \\ \vdots& \vdots& \ddots& \vdots\\ 0 & 0 & \cdots& \sqrt{\mu_{in_{i}}(1-\mu_{{in}_{i}})} \end{matrix} \right]$ , a $n_{i}\times n_{i}$ matrix

$$\mathbf{V}_{i}= \mathbf{A}_{i}^{1/2}{\mathbf{R}_{i}\mathbf{A}}_{i}^{1/2}=\left[ \begin{matrix} \sqrt{\mu_{i1}(1-\mu_{i1})} & 0 & \cdots& 0 \\ 0 & \sqrt{\mu_{i2}(1-\mu_{i2})} & \ldots& 0 \\ \vdots& \vdots& \ddots& \vdots\\ 0 & 0 & \cdots& \sqrt{\mu_{{in}_{i}}(1-\mu_{{in}_{i}})} \end{matrix} \right]\mathbf{I}_{n_{i}}\left[ \begin{matrix} \sqrt{\mu_{i1}(1-\mu_{i1})} & 0 & \cdots& 0 \\ 0 & \sqrt{\mu_{i2}(1-\mu_{i2})} & \ldots& 0 \\ \vdots& \vdots& \ddots& \vdots\\ 0 & 0 & \cdots& \sqrt{\mu_{in_{i}}(1-\mu_{{in}_{i}})} \end{matrix} \right]$$

$$=\left[ \begin{matrix} \mu_{i1}(1-\mu_{i1}) & 0 & \cdots& 0 \\ 0 & \mu_{i2}(1-\mu_{i2}) & \ldots& 0 \\ \vdots& \vdots& \ddots& \vdots\\ 0 & 0 & \cdots& \mu_{{in}_{i}}(1-\mu_{in_{i}}) \end{matrix} \right]$$

$$\mathbf{V}_{i}^{-1}=\left[ \begin{matrix} \left[ \mu_{i1}\left( 1-\mu_{i1} \right) \right]^{-1} & 0 & \cdots& 0 \\ 0 & \left[ \mu_{i2}\left( 1-\mu_{i2} \right) \right]^{-1} & \ldots& 0 \\ \vdots& \vdots& \ddots& \vdots\\ 0 & 0 & \cdots& \left[ \mu_{in_{i}}\left( 1-\mu_{{in}_{i}} \right) \right]^{-1} \end{matrix} \right]$$

$\mathbf{C}_{i}= \left[ \begin{matrix} 1 & \rho& \cdots& \rho\\ \rho& 1 & \ldots& \rho\\ \vdots& \vdots& \ddots& \vdots\\ \rho& \rho& \cdots& 1 \end{matrix} \right]$ , a $n_{i}\times n_{i}$ matrix

$$\mathrm{cov}\left( \mathbf{Y}_{i} \right)=\mathbf{A}_{i}^{1/2}\mathbf{C}_{i}\mathbf{A}_{i}^{1/2}=$$

$$\left[ \begin{matrix} \sqrt{\mu_{i1}\left( 1-\mu_{i1} \right)} & 0 & \cdots& 0 \\ 0 & \sqrt{\mu_{i2}\left( 1-\mu_{i2} \right)} & \ldots& 0 \\ \vdots& \vdots& \ddots& \vdots\\ 0 & 0 & \cdots& \sqrt{\mu_{in_{i}}\left( 1-\mu_{in_{i}} \right)} \end{matrix} \right] \left[ \begin{matrix} 1 & \rho& \cdots& \rho\\ \rho& 1 & \ldots& \rho\\ \vdots& \vdots& \ddots& \vdots\\ \rho& \rho& \cdots& 1 \end{matrix} \right]\left[ \begin{matrix} \sqrt{\mu_{i1}\left( 1-\mu_{i1} \right)} & 0 & \cdots& 0 \\ 0 & \sqrt{\mu_{i2}\left( 1-\mu_{i2} \right)} & \ldots& 0 \\ \vdots& \vdots& \ddots& \vdots\\ 0 & 0 & \cdots& \sqrt{\mu_{in_{i}}\left( 1-\mu_{{in}_{i}} \right)} \end{matrix} \right]$$

$$=\left[ \begin{matrix} \sqrt{\mu_{i1}\left( 1-\mu_{i1} \right)} & \rho\sqrt{\mu_{i1}\left( 1-\mu_{i1} \right)} & \cdots& \rho\sqrt{\mu_{i1}\left( 1-\mu_{i1} \right)} \\ \rho\sqrt{\mu_{i2}\left( 1-\mu_{i2} \right)} & \sqrt{\mu_{i2}\left( 1-\mu_{i2} \right)} & \ldots& \rho\sqrt{\mu_{i2}\left( 1-\mu_{i2} \right)} \\ \vdots& \vdots& \ddots& \vdots\\ \rho\sqrt{\mu_{{in}_{i}}\left( 1-\mu_{in_{i}} \right)} & \rho\sqrt{\mu_{in_{i}}\left( 1-\mu_{in_{i}} \right)} & \cdots& \sqrt{\mu_{in_{i}}\left( 1-\mu_{in_{i}} \right)} \end{matrix} \right] \left[ \begin{matrix} \sqrt{\mu_{i1}\left( 1-\mu_{i1} \right)} & 0 & \cdots& 0 \\ 0 & \sqrt{\mu_{i2}\left( 1-\mu_{i2} \right)} & \ldots& 0 \\ \vdots& \vdots& \ddots& \vdots\\ 0 & 0 & \cdots& \sqrt{\mu_{in_{i}}\left( 1-\mu_{in_{i}} \right)} \end{matrix} \right]$$

$$=\left[ \begin{matrix} \mu_{i1}\left( 1-\mu_{i1} \right) & \rho\sqrt{\mu_{i1}\mu_{i2}\left( 1-\mu_{i1} \right)\left( 1-\mu_{i2} \right)} & \cdots& \rho\sqrt{\mu_{i1}\mu_{in_{i}}\left( 1-\mu_{i1} \right)\left( 1-\mu_{{in}_{i}} \right)} \\ \rho\sqrt{\mu_{i1}\mu_{i2}\left( 1-\mu_{i1} \right)\left( 1-\mu_{i2} \right)} & \mu_{i2}\left( 1-\mu_{i2} \right) & \ldots& \rho\sqrt{\mu_{i2}\mu_{{in}_{i}}\left( 1-\mu_{i2} \right)\left( 1-\mu_{in_{i}} \right)} \\ \vdots& \vdots& \ddots& \vdots\\ \rho\sqrt{\mu_{i1}\mu_{in_{i}}\left( 1-\mu_{i1} \right)\left( 1-\mu_{{in}_{i}} \right)} & \rho\sqrt{\mu_{i2}\mu_{in_{i}}\left( 1-\mu_{i2} \right)\left( 1-\mu_{in_{i}} \right)} & \cdots& \mu_{{in}_{i}}\left( 1-\mu_{{in}_{i}} \right) \end{matrix} \right]$$

$$\mathbf{D}_{i}=\left[ \begin{matrix} \begin{matrix} \frac{\partial\mu_{i1}}{\partial\beta_{0}} \end{matrix} & \frac{\partial\mu_{i1}}{\partial\beta_{1}} \\ \vdots& \vdots\\ \frac{\partial\mu_{in_{i}}}{\partial\beta_{0}} & \frac{\partial\mu_{in_{i}}}{\partial\beta_{1}} \end{matrix} \right]=\left[ \begin{matrix} \begin{matrix} \mu_{i1} \end{matrix} & X_{i1}\mu_{i1} \\ \vdots& \vdots\\ \mu_{in_{i}} & X_{in_{i}}\mu_{in_{i}} \end{matrix} \right]$$

$$\mathbf{D}_{i}^{T}\mathbf{V}_{i}^{-1}\mathbf{D}_{i}= \left[ \begin{matrix} \begin{matrix} \mu_{i1} \end{matrix} & \cdots& \mu_{{in}_{i}} \\ X_{i1}\mu_{i1} & \cdots& X_{in_{i}}\mu_{{in}_{i}} \end{matrix} \right]\left[ \begin{matrix} \left[ \mu_{i1}\left( 1-\mu_{i1} \right) \right]^{-1} & 0 & \cdots& 0 \\ 0 & \left[ \mu_{i2}\left( 1-\mu_{i2} \right) \right]^{-1} & \ldots& 0 \\ \vdots& \vdots& \ddots& \vdots\\ 0 & 0 & \cdots& \left[ \mu_{{in}_{i}}\left( 1-\mu_{{in}_{i}} \right) \right]^{-1} \end{matrix} \right]\left[ \begin{matrix} \begin{matrix} \mu_{i1} \end{matrix} & X_{i1}\mu_{i1} \\ \vdots& \vdots\\ \mu_{{in}_{i}} & X_{in_{i}}\mu_{{in}_{i}} \end{matrix} \right]$$

$$= \left[ \begin{matrix} \left( 1-\mu_{i1} \right)^{-1} & \cdots& \left( 1-\mu_{{in}_{i}} \right)^{-1} \\ X_{i1}\left( 1-\mu_{i1} \right)^{-1} & \cdots& X_{in_{i}}\left( 1-\mu_{i1} \right)^{-1} \end{matrix} \right]\left[ \begin{matrix} \begin{matrix} \mu_{i1} \end{matrix} & X_{i1}\mu_{i1} \\ \vdots& \vdots\\ \mu_{in_{i}} & X_{in_{i}}\mu_{{in}_{i}} \end{matrix} \right] = \left[ \begin{matrix} \begin{matrix} \sum_{j=1}^{n_{i}} \frac{\mu_{ij}}{1-\mu_{ij}} \end{matrix} & \sum_{j=1}^{n_{i}} \frac{X_{ij}\mu_{ij}}{1-\mu_{ij}} \\ \sum_{j=1}^{n_{i}} \frac{X_{ij}\mu_{ij}}{1-\mu_{ij}} & \sum_{j=1}^{n_{i}} \frac{X_{ij}^{2}\mu_{ij}}{1-\mu_{ij}} \end{matrix} \right]$$

Note that when $n_{i}=1$, this equals $\left[ \begin{matrix} \frac{\mu_{i1}}{1-\mu_{i1}} & \frac{X_{ij}\mu_{i1}}{1-\mu_{i1}} \\ \frac{X_{i1}\mu_{i1}}{1-\mu_{i1}} & \frac{X_{i1}^{2}\mu_{i1}}{1-\mu_{i1}} \end{matrix} \right]$ and therefore this expression for $\mathbf{D}_{i}^{T}\mathbf{V}_{i}^{-1}\mathbf{D}_{i}$ holds for all $n_{i}\geq1$.

$$\mathbf{D}_{i}^{T}\mathbf{V}_{i}^{-1}{\mathrm{cov}\left( \mathbf{Y}_{i} \right)\mathbf{V}_{i}^{-1}\mathbf{D}}_{i}=$$

$$\left[ \begin{matrix} \begin{matrix} \mu_{i1} \end{matrix} & \cdots& \mu_{in_{i}} \\ X_{i1}\mu_{i1} & \cdots& X_{in_{i}}\mu_{{in}_{i}} \end{matrix} \right]\left[ \begin{matrix} \left[ \mu_{i1}\left( 1-\mu_{i1} \right) \right]^{-1} & 0 & \cdots& 0 \\ 0 & \left[ \mu_{i2}\left( 1-\mu_{i2} \right) \right]^{-1} & \ldots& 0 \\ \vdots& \vdots& \ddots& \vdots\\ 0 & 0 & \cdots& \left[ \mu_{{in}_{i}}\left( 1-\mu_{{in}_{i}} \right) \right]^{-1} \end{matrix} \right]\times\left[ \begin{matrix} \mu_{i1}\left( 1-\mu_{i1} \right) & \rho\sqrt{\mu_{i1}\mu_{i2}\left( 1-\mu_{i1} \right)\left( 1-\mu_{i2} \right)} & \cdots& \rho\sqrt{\mu_{i1}\mu_{in_{i}}\left( 1-\mu_{i1} \right)\left( 1-\mu_{{in}_{i}} \right)} \\ \rho\sqrt{\mu_{i1}\mu_{i2}\left( 1-\mu_{i1} \right)\left( 1-\mu_{i2} \right)} & \mu_{i2}\left( 1-\mu_{i2} \right) & \ldots& \rho\sqrt{\mu_{i2}\mu_{in_{i}}\left( 1-\mu_{i2} \right)\left( 1-\mu_{in_{i}} \right)} \\ \vdots& \vdots& \ddots& \vdots\\ \rho\sqrt{\mu_{i1}\mu_{in_{i}}\left( 1-\mu_{i1} \right)\left( 1-\mu_{{in}_{i}} \right)} & \rho\sqrt{\mu_{i2}\mu_{{in}_{i}}\left( 1-\mu_{i2} \right)\left( 1-\mu_{{in}_{i}} \right)} & \cdots& \mu_{in_{i}}\left( 1-\mu_{in_{i}} \right) \end{matrix} \right]\times\left[ \begin{matrix} \left[ \mu_{i1}\left( 1-\mu_{i1} \right) \right]^{-1} & 0 & \cdots& 0 \\ 0 & \left[ \mu_{i2}\left( 1-\mu_{i2} \right) \right]^{-1} & \ldots& 0 \\ \vdots& \vdots& \ddots& \vdots\\ 0 & 0 & \cdots& \left[ \mu_{in_{i}}\left( 1-\mu_{in_{i}} \right) \right]^{-1} \end{matrix} \right]\left[ \begin{matrix} \begin{matrix} \mu_{i1} \end{matrix} & X_{i1}\mu_{i1} \\ \vdots& \vdots\\ \mu_{in_{i}} & X_{{in}_{i}}\mu_{{in}_{i}} \end{matrix} \right]$$

$$=\left[ \begin{matrix} \left( 1-\mu_{i1} \right)^{-1} & \cdots& \left( 1-\mu_{{in}_{i}} \right)^{-1} \\ X_{i1}\left( 1-\mu_{i1} \right)^{-1} & \cdots& X_{{in}_{i}}\left( 1-\mu_{i1} \right)^{-1} \end{matrix} \right] \left[ \begin{matrix} \mu_{i1}\left( 1-\mu_{i1} \right) & \rho\sqrt{\mu_{i1}\mu_{i2}\left( 1-\mu_{i1} \right)\left( 1-\mu_{i2} \right)} & \cdots& \rho\sqrt{\mu_{i1}\mu_{in_{i}}\left( 1-\mu_{i1} \right)\left( 1-\mu_{{in}_{i}} \right)} \\ \rho\sqrt{\mu_{i1}\mu_{i2}\left( 1-\mu_{i1} \right)\left( 1-\mu_{i2} \right)} & \mu_{i2}\left( 1-\mu_{i2} \right) & \ldots& \rho\sqrt{\mu_{i2}\mu_{{in}_{i}}\left( 1-\mu_{i2} \right)\left( 1-\mu_{in_{i}} \right)} \\ \vdots& \vdots& \ddots& \vdots\\ \rho\sqrt{\mu_{i1}\mu_{{in}_{i}}\left( 1-\mu_{i1} \right)\left( 1-\mu_{{in}_{i}} \right)} & \rho\sqrt{\mu_{i2}\mu_{{in}_{i}}\left( 1-\mu_{i2} \right)\left( 1-\mu_{{in}_{i}} \right)} & \cdots& \mu_{{in}_{i}}\left( 1-\mu_{{in}_{i}} \right) \end{matrix} \right]\left[ \begin{matrix} \left( 1-\mu_{i1} \right)^{-1} & X_{i1}\left( 1-\mu_{i1} \right)^{-1} \\ \vdots& \vdots\\ \left( 1-\mu_{{in}_{i}} \right)^{-1} & X_{in_{i}}\left( 1-\mu_{i1} \right)^{-1} \end{matrix} \right]$$

$$=\left[ \begin{matrix} (1-\rho)\mu_{i1}+\rho\sum_{j=1}^{n_{i}} \frac{\sqrt{\mu_{i1}\mu_{ij}\left( 1-\mu_{i1} \right)\left( 1-\mu_{ij} \right)}}{1-\mu_{ij}} & \cdots& (1-\rho)\mu_{{in}_{i}}+\rho\sum_{j=1}^{n_{i}} \frac{\sqrt{\mu_{in_{i}}\mu_{ij}\left( 1-\mu_{{in}_{i}} \right)\left( 1-\mu_{ij} \right)}}{1-\mu_{ij}} \\ (1-\rho){X_{i1}\mu}_{i1}+\rho\sum_{j=1}^{n_{i}} \frac{X_{j}\sqrt{\mu_{i1}\mu_{ij}\left( 1-\mu_{i1} \right)\left( 1-\mu_{ij} \right)}}{1-\mu_{ij}} & \cdots& {(1-\rho)X_{in_{i}}\mu}_{in_{i}}+\rho\sum_{j=1}^{n_{i}} \frac{X_{ij}\sqrt{\mu_{in_{i}}\mu_{ij}\left( 1-\mu_{in_{i}} \right)\left( 1-\mu_{ij} \right)}}{1-\mu_{ij}} \end{matrix} \right]\left[ \begin{matrix} \left( 1-\mu_{i1} \right)^{-1} & X_{i1}\left( 1-\mu_{i1} \right)^{-1} \\ \vdots& \vdots\\ \left( 1-\mu_{{in}_{i}} \right)^{-1} & X_{in_{i}}\left( 1-\mu_{i1} \right)^{-1} \end{matrix} \right]$$

$$=\left[ \begin{matrix} \sum_{j=1}^{n_{i}} \frac{\mu_{ij}}{1-\mu_{ij}}+2\rho\sum_{j=1}^{n_{i}} \sum_{a\neq j} \frac{\sqrt{\mu_{ij}\mu_{ia}\left( 1-\mu_{ij} \right)\left( 1-\mu_{ia} \right)}}{(1-\mu_{ij})(1-\mu_{ia})} & \sum_{j=1}^{n_{i}} \frac{X_{ij}\mu_{ij}}{1-\mu_{ij}}+\rho\sum_{j=1}^{n_{i}} \sum_{a\neq j} \frac{(X_{ij}+X_{ia})\sqrt{\mu_{ij}\mu_{ia}\left( 1-\mu_{ij} \right)\left( 1-\mu_{ia} \right)}}{(1-\mu_{ij})(1-\mu_{ia})} \\ \sum_{j=1}^{n_{i}} \frac{X_{ij}\mu_{ij}}{1-\mu_{ij}}+\rho\sum_{j=1}^{n_{i}} \sum_{a\neq j} \frac{(X_{ij}+X_{ia})\sqrt{\mu_{ij}\mu_{ia}\left( 1-\mu_{ij} \right)\left( 1-\mu_{ia} \right)}}{(1-\mu_{ij})(1-\mu_{ia})} & \sum_{j=1}^{n_{i}} \frac{X_{ij}^{2}\mu_{ij}}{1-\mu_{ij}}+2\rho\sum_{j=1}^{n_{i}} \sum_{a\neq j} \frac{X_{ij}X_{ia}\sqrt{\mu_{ij}\mu_{ia}\left( 1-\mu_{ij} \right)\left( 1-\mu_{ia} \right)}}{(1-\mu_{ij})(1-\mu_{ia})} \end{matrix} \right]$$

Note that when $n_{i}=1$, this equals $\left[ \begin{matrix} \frac{\mu_{i1}}{1-\mu_{i1}} & \frac{X_{i}\mu_{i1}}{1-\mu_{i1}} \\ \frac{X_{i1}\mu_{i1}}{1-\mu_{i1}} & \frac{X_{i1}^{2}\mu_{i1}}{1-\mu_{i1}} \end{matrix} \right]$ and therefore this expression for $\mathbf{D}_{i}^{T}\mathbf{V}_{i}^{-1}{\mathrm{cov}\left( \mathbf{Y}_{i} \right)\mathbf{V}_{i}^{-1}\mathbf{D}}_{i}$ holds for all $n_{i}\geq1$.

The first sum in each element of this matrix can be rewritten as the sum over cluster members assigned to intervention plus the sum over the cluster members assigned to control (e.g. $\sum_{j=1}^{n_{i}} \frac{\mu_{ij}}{1-\mu_{ij}}$ can be rewritten as $\sum_{j=1|X_{ij}=1}^{n_{i}} \frac{\mu_{ij}}{1-\mu_{ij}}+\sum_{j=1|X_{ij}=0}^{n_{i}} \frac{\mu_{ij}}{1-\mu_{ij}}$). A similar approach can be used for the double sums in each element of the matrix, by rewriting these as the sums of paired terms where the two cluster members are both assigned to intervention, both assigned to control, and one to intervention and one to control. For example, $\sum_{j=1}^{n_{i}} \sum_{a\neq j} \frac{\sqrt{\mu_{ij}\mu_{ia}\left( 1-\mu_{ij} \right)\left( 1-\mu_{ia} \right)}}{(1-\mu_{ij})(1-\mu_{ia})}=\sum_{j=1|X_{ij}=1}^{n_{i}} \sum_{a\neq j|X_{ia}=1} \frac{\sqrt{\mu_{ij}\mu_{ia}\left( 1-\mu_{ij} \right)\left( 1-\mu_{ia} \right)}}{(1-\mu_{ij})(1-\mu_{ia})}+\sum_{j=1|X_{ij}=0}^{n_{i}} \sum_{a\neq j|X_{ia}=0} \frac{\sqrt{\mu_{ij}\mu_{ia}\left( 1-\mu_{ij} \right)\left( 1-\mu_{ia} \right)}}{(1-\mu_{ij})(1-\mu_{ia})}+\sum_{j=1|X_{ij}=1}^{n_{i}} \sum_{a\neq j|X_{ia}=0} \frac{\sqrt{\mu_{ij}\mu_{ia}\left( 1-\mu_{ij} \right)\left( 1-\mu_{ia} \right)}}{(1-\mu_{ij})(1-\mu_{ia})}$. The sum over pairs where both are assigned to intervention is a sum of $\binom{t_{i}}{2}=t_{i}(t_{i}-1)/2$ terms, the sum over pairs where both are assigned to control is a sum of $\binom{c_{i}}{2}=c_{i}(c_{i}-1)/2$ terms, and the sum over pairs with one member assigned to intervention and one to control is a sum of $t_{i}c_{i}$ terms.

$\Rightarrow\mathbf{D}_{i}^{T}\mathbf{V}_{i}^{-1}{\mathrm{cov}\left( \mathbf{Y}_{i} \right)\mathbf{V}_{i}^{-1}\mathbf{D}}_{i}= \left[ \begin{matrix} a & b \\ b & c \end{matrix} \right]$ , where

$$a=\sum_{j=1}^{n_{i}} \frac{\mu_{ij}}{1-\mu_{ij}}+2\rho\sum_{j=1}^{n_{i}} \sum_{a\neq j} \frac{\sqrt{\mu_{ij}\mu_{ia}\left( 1-\mu_{ij} \right)\left( 1-\mu_{ia} \right)}}{(1-\mu_{ij})(1-\mu_{ia})}$$

$$=\frac{\pi_{I}}{1-\pi_{I}}t_{i}+\frac{\pi_{C}}{1-\pi_{C}}c_{i}+2\rho\left[ \frac{\sqrt{\pi_{I}^{2}\left( 1-\pi_{I} \right)^{2}}}{\left( 1-\pi_{I} \right)^{2}}\left( \frac{t_{i}\left( t_{i}-1 \right)}{2} \right)+\frac{\sqrt{\pi_{C}^{2}\left( 1-\pi_{C} \right)^{2}}}{\left( 1-\pi_{C} \right)^{2}}\left( \frac{c_{i}\left( c_{i}-1 \right)}{2} \right)+\frac{\sqrt{\pi_{I}\pi_{C}\left( 1-\pi_{I} \right)\left( 1-\pi_{C} \right)}}{\left( 1-\pi_{I} \right)\left( 1-\pi_{C} \right)}t_{i}c_{i} \right]$$

$$=\frac{\pi_{I}}{1-\pi_{I}}t_{i}+\frac{\pi_{C}}{1-\pi_{C}}c_{i}+\frac{\pi_{I}}{1-\pi_{I}}\rho t_{i}\left( t_{i}-1 \right)+\frac{\pi_{C}}{1-\pi_{C}}\rho c_{i}\left( c_{i}-1 \right)+\frac{2\sqrt{\pi_{I}\pi_{C}\left( 1-\pi_{I} \right)\left( 1-\pi_{C} \right)}}{\left( 1-\pi_{I} \right)\left( 1-\pi_{C} \right)}\rho t_{i}c_{i}$$

$$b=\sum_{j=1}^{n_{i}} \frac{X_{ij}\mu_{ij}}{1-\mu_{ij}}+\rho\sum_{j=1}^{n_{i}} \sum_{a\neq j} \frac{(X_{ij}+X_{ia})\sqrt{\mu_{ij}\mu_{ia}\left( 1-\mu_{ij} \right)\left( 1-\mu_{ia} \right)}}{(1-\mu_{ij})(1-\mu_{ia})}$$

$$=\frac{\left( 1 \right)\pi_{I}}{1-\pi_{I}}t_{i}+\rho\left[ \frac{(1+1)\sqrt{\pi_{I}^{2}\left( 1-\pi_{I} \right)^{2}}}{\left( 1-\pi_{I} \right)^{2}}\left( \frac{t_{i}\left( t_{i}-1 \right)}{2} \right)+\frac{(1+0)\sqrt{\pi_{I}\pi_{C}\left( 1-\pi_{I} \right)\left( 1-\pi_{C} \right)}}{\left( 1-\pi_{I} \right)\left( 1-\pi_{C} \right)}t_{i}c_{i} \right]$$

$$=\frac{\pi_{I}}{1-\pi_{I}}t_{i}+\frac{\pi_{I}}{1-\pi_{I}}\rho t_{i}\left( t_{i}-1 \right)+\frac{\sqrt{\pi_{I}\pi_{C}\left( 1-\pi_{I} \right)\left( 1-\pi_{C} \right)}}{\left( 1-\pi_{I} \right)\left( 1-\pi_{C} \right)}\rho t_{i}c_{i}$$

$$c=\sum_{j=1}^{n_{i}} \frac{X_{ij}^{2}\mu_{ij}}{1-\mu_{ij}}+2\rho\sum_{j=1}^{n_{i}} \sum_{a\neq j} \frac{X_{ij}X_{ia}\sqrt{\mu_{ij}\mu_{ia}\left( 1-\mu_{ij} \right)\left( 1-\mu_{ia} \right)}}{(1-\mu_{ij})(1-\mu_{ia})} = \frac{\left( 1 \right)\pi_{I}}{1-\pi_{I}}t_{i}+2\rho\left[ \frac{\left( 1 \right)^{2}\sqrt{\pi_{I}^{2}\left( 1-\pi_{I} \right)^{2}}}{\left( 1-\pi_{I} \right)^{2}}\left( \frac{t_{i}\left( t_{i}-1 \right)}{2} \right) \right] = \frac{\pi_{I}}{1-\pi_{I}}t_{i}+\frac{\pi_{I}}{1-\pi_{I}}\rho t_{i}\left( t_{i}-1 \right)$$

Sum over all clusters $i=1,\ldots, M$. Cluster $i$ is of size $n_{i}$and contains $t_{i}$ members assigned to intervention and $c_{i}$ members assigned to control.

$$\sum_{i=1}^{M} \mathbf{D}_{i}^{T}\mathbf{V}_{i}^{-1}\mathbf{D}_{i}= \sum_{i=1}^{M} \left[ \begin{matrix} \begin{matrix} \sum_{j=1}^{n_{i}} \frac{\mu_{ij}}{1-\mu_{i1}} \end{matrix} & \sum_{j=1}^{n_{i}} \frac{X_{ij}\mu_{ij}}{1-\mu_{i1}} \\ \sum_{j=1}^{n_{i}} \frac{X_{ij}\mu_{ij}}{1-\mu_{i1}} & \sum_{j=1}^{n_{i}} \frac{X_{ij}^{2}\mu_{ij}}{1-\mu_{i1}} \end{matrix} \right] = \sum_{i=1}^{M} \left[ \begin{matrix} t_{i}\frac{\pi_{I}}{1-\pi_{I}}+c_{i}\frac{\pi_{C}}{1-\pi_{C}} & t_{i}\frac{\pi_{I}}{1-\pi_{I}} \\ t_{i}\frac{\pi_{I}}{1-\pi_{I}} & t_{i}\frac{\pi_{I}}{1-\pi_{I}} \end{matrix} \right] = \left[ \begin{matrix} N_{I}\frac{\pi_{I}}{1-\pi_{I}}+N_{C}\frac{\pi_{C}}{1-\pi_{C}} & N_{I}\frac{\pi_{I}}{1-\pi_{I}} \\ N_{I}\frac{\pi_{I}}{1-\pi_{I}} & N_{I}\frac{\pi_{I}}{1-\pi_{I}} \end{matrix} \right]$$

$=\frac{N}{2}\left[ \begin{matrix} \frac{\pi_{I}}{1-\pi_{I}}+\frac{\pi_{C}}{1-\pi_{C}} & \frac{\pi_{I}}{1-\pi_{I}} \\ \frac{\pi_{I}}{1-\pi_{I}} & \frac{\pi_{I}}{1-\pi_{I}} \end{matrix} \right]$ under the assumption of overall treatment balance

$$\left[ \sum_{i=1}^{M} \mathbf{D}_{i}^{T}\mathbf{V}_{i}^{-1}\mathbf{D}_{i} \right]^{-1}= \frac{2}{N\left[ \left( \frac{\pi_{I}}{1-\pi_{I}}+\frac{\pi_{C}}{1-\pi_{C}} \right)\left( \frac{\pi_{I}}{1-\pi_{I}} \right)-\frac{\pi_{I}^{2}}{\left( 1-\pi_{I} \right)^{2}} \right]}\left[ \begin{matrix} \frac{\pi_{I}}{1-\pi_{I}} & \frac{-\pi_{I}}{1-\pi_{I}} \\ \frac{-\pi_{I}}{1-\pi_{I}} & \frac{\pi_{I}}{1-\pi_{I}}+\frac{\pi_{C}}{1-\pi_{C}} \end{matrix} \right] = \frac{2\left( 1-\pi_{I} \right)\left( 1-\pi_{C} \right)}{N\pi_{I}\pi_{C}}\left[ \begin{matrix} \frac{\pi_{I}}{1-\pi_{I}} & \frac{-\pi_{I}}{1-\pi_{I}} \\ \frac{-\pi_{I}}{1-\pi_{I}} & \frac{\pi_{I}}{1-\pi_{I}}+\frac{\pi_{C}}{1-\pi_{C}} \end{matrix} \right]$$

$\sum_{i=1}^{M} \mathbf{D}_{i}^{T}\mathbf{V}_{i}^{-1}{\mathrm{cov}\left( \mathbf{Y}_{i} \right)\mathbf{V}_{i}^{-1}\mathbf{D}}_{i}=\left[ \begin{matrix} \sum_{i=1}^{M} a & \sum_{i=1}^{M} b \\ \sum_{i=1}^{M} b & \sum_{i=1}^{M} c \end{matrix} \right]= \left[ \begin{matrix} A & B \\ B & C \end{matrix} \right]$ , where under the assumption of overall treatment balance:

$$A=\sum_{i=1}^{M} \left( \frac{\pi_{I}}{1-\pi_{I}}t_{i}+\frac{\pi_{C}}{1-\pi_{C}}c_{i}+\frac{\pi_{I}}{1-\pi_{I}}\rho t_{i}\left( t_{i}-1 \right)+\frac{\pi_{C}}{1-\pi_{C}}\rho c_{i}\left( c_{i}-1 \right)+\frac{2\sqrt{\pi_{I}\pi_{C}\left( 1-\pi_{I} \right)\left( 1-\pi_{C} \right)}}{\left( 1-\pi_{I} \right)\left( 1-\pi_{C} \right)}\rho t_{i}c_{i} \right)$$

$$=N_{I}\frac{\pi_{I}}{1-\pi_{I}}+N_{C}\frac{\pi_{C}}{1-\pi_{C}}+\frac{\pi_{I}}{1-\pi_{I}}\rho\sum_{i=1}^{M} t_{i}\left( t_{i}-1 \right)+\frac{\pi_{C}}{1-\pi_{C}}\rho\sum_{i=1}^{M} c_{i}\left( c_{i}-1 \right)+\frac{2\sqrt{\pi_{I}\pi_{C}\left( 1-\pi_{I} \right)\left( 1-\pi_{C} \right)}}{\left( 1-\pi_{I} \right)\left( 1-\pi_{C} \right)}\rho\sum_{i=1}^{M} t_{i}c_{i}$$

$$=\frac{N}{2}\left( \frac{\pi_{I}}{1-\pi_{I}}+\frac{\pi_{C}}{1-\pi_{C}} \right)+\frac{\pi_{I}}{1-\pi_{I}}\rho\sum_{i=1}^{M} t_{i}\left( t_{i}-1 \right)+\frac{\pi_{C}}{1-\pi_{C}}\rho\sum_{i=1}^{M} c_{i}\left( c_{i}-1 \right)+\frac{2\sqrt{\pi_{I}\pi_{C}\left( 1-\pi_{I} \right)\left( 1-\pi_{C} \right)}}{\left( 1-\pi_{I} \right)\left( 1-\pi_{C} \right)}\rho\sum_{i=1}^{M} t_{i}c_{i}$$

$$B=\sum_{i=1}^{M} \left( \frac{\pi_{I}}{1-\pi_{I}}t_{i}+\frac{\pi_{I}}{1-\pi_{I}}\rho t_{i}\left( t_{i}-1 \right)+\frac{\sqrt{\pi_{I}\pi_{C}\left( 1-\pi_{I} \right)\left( 1-\pi_{C} \right)}}{\left( 1-\pi_{I} \right)\left( 1-\pi_{C} \right)}\rho t_{i}c_{i} \right)$$

$$=N_{I}\frac{\pi_{I}}{1-\pi_{I}}+\frac{\pi_{I}}{1-\pi_{I}}\rho\sum_{i=1}^{M} t_{i}\left( t_{i}-1 \right)+\frac{\sqrt{\pi_{I}\pi_{C}\left( 1-\pi_{I} \right)\left( 1-\pi_{C} \right)}}{\left( 1-\pi_{I} \right)\left( 1-\pi_{C} \right)}\rho\sum_{i=1}^{M} t_{i}c_{i}$$

$$=\frac{N}{2}\left( \frac{\pi_{I}}{1-\pi_{I}} \right)+\frac{\pi_{I}}{1-\pi_{I}}\rho\sum_{i=1}^{M} t_{i}\left( t_{i}-1 \right)+\frac{\sqrt{\pi_{I}\pi_{C}\left( 1-\pi_{I} \right)\left( 1-\pi_{C} \right)}}{\left( 1-\pi_{I} \right)\left( 1-\pi_{C} \right)}\rho\sum_{i=1}^{M} t_{i}c_{i}$$

$$C=\sum_{i=1}^{M} \left( \frac{\pi_{I}}{1-\pi_{I}}t_{i}+\frac{\pi_{I}}{1-\pi_{I}}\rho t_{i}\left( t_{i}-1 \right) \right) = N_{I}\frac{\pi_{I}}{1-\pi_{I}}+\frac{\pi_{I}}{1-\pi_{I}}\rho\sum_{i=1}^{M} t_{i}\left( t_{i}-1 \right) = \frac{N}{2}\left( \frac{\pi_{I}}{1-\pi_{I}} \right)+\frac{\pi_{I}}{1-\pi_{I}}\rho\sum_{i=1}^{M} t_{i}\left( t_{i}-1 \right)$$

$$\mathrm{cov}\left( \hat{\boldsymbol{\beta}} \right)= \left[ \sum_{i=1}^{M} \mathbf{D}_{i}^{T}\mathbf{V}_{i}^{-1}\mathbf{D}_{i} \right]^{-1}\sum_{i=1}^{M} \mathbf{D}_{i}^{T}\mathbf{V}_{i}^{-1}{\mathrm{cov}\left( \mathbf{Y}_{i} \right)\mathbf{V}_{i}^{-1}\mathbf{D}}_{i}\left[ \sum_{i=1}^{M} \mathbf{D}_{i}^{T}\mathbf{V}_{i}^{-1}\mathbf{D}_{i} \right]^{-1}$$

$$= \frac{2\left( 1-\pi_{I} \right)\left( 1-\pi_{C} \right)}{N\pi_{I}\pi_{C}}\left[ \begin{matrix} \frac{\pi_{I}}{1-\pi_{I}} & \frac{-\pi_{I}}{1-\pi_{I}} \\ \frac{-\pi_{I}}{1-\pi_{I}} & \frac{\pi_{I}}{1-\pi_{I}}+\frac{\pi_{C}}{1-\pi_{C}} \end{matrix} \right] \left[ \begin{matrix} A & B \\ B & C \end{matrix} \right]\frac{2\left( 1-\pi_{I} \right)\left( 1-\pi_{C} \right)}{N\pi_{I}\pi_{C}}\left[ \begin{matrix} \frac{\pi_{I}}{1-\pi_{I}} & \frac{-\pi_{I}}{1-\pi_{I}} \\ \frac{-\pi_{I}}{1-\pi_{I}} & \frac{\pi_{I}}{1-\pi_{I}}+\frac{\pi_{C}}{1-\pi_{C}} \end{matrix} \right]$$

$$=\frac{4\left( 1-\pi_{I} \right)^{2}\left( 1-\pi_{C} \right)^{2}}{\left( N\pi_{I}\pi_{C} \right)^{2}}\left[ \begin{matrix} D & E \\ F & G \end{matrix} \right]\left[ \begin{matrix} \frac{\pi_{I}}{1-\pi_{I}} & \frac{-\pi_{I}}{1-\pi_{I}} \\ \frac{-\pi_{I}}{1-\pi_{I}} & \frac{\pi_{I}}{1-\pi_{I}}+\frac{\pi_{C}}{1-\pi_{C}} \end{matrix} \right]$$

where:

$$D=\frac{\pi_{I}}{1-\pi_{I}}A-\frac{\pi_{I}}{1-\pi_{I}}B$$

$$=\frac{\pi_{I}}{1-\pi_{I}}\left[ \frac{N}{2}\left( \frac{\pi_{I}}{1-\pi_{I}}+\frac{\pi_{C}}{1-\pi_{C}} \right)+\frac{\pi_{I}}{1-\pi_{I}}\rho\sum_{i=1}^{M} t_{i}\left( t_{i}-1 \right)+\frac{\pi_{C}}{1-\pi_{C}}\rho\sum_{i=1}^{M} c_{i}\left( c_{i}-1 \right)+\frac{2\sqrt{\pi_{I}\pi_{C}\left( 1-\pi_{I} \right)\left( 1-\pi_{C} \right)}}{\left( 1-\pi_{I} \right)\left( 1-\pi_{C} \right)}\rho\sum_{i=1}^{M} t_{i}c_{i} \right]-\frac{\pi_{I}}{1-\pi_{I}}\left[ \frac{N}{2}\left( \frac{\pi_{I}}{1-\pi_{I}} \right)+\frac{\pi_{I}}{1-\pi_{I}}\rho\sum_{i=1}^{M} t_{i}\left( t_{i}-1 \right)+\frac{\sqrt{\pi_{I}\pi_{C}\left( 1-\pi_{I} \right)\left( 1-\pi_{C} \right)}}{\left( 1-\pi_{I} \right)\left( 1-\pi_{C} \right)}\rho\sum_{i=1}^{M} t_{i}c_{i} \right]$$

$$=\frac{{N\pi}_{I}\pi_{C}}{2\left( 1-\pi_{I} \right)\left( 1-\pi_{C} \right)}+\frac{\pi_{I}\pi_{C}}{\left( 1-\pi_{I} \right)\left( 1-\pi_{C} \right)}\rho\sum_{i=1}^{M} c_{i}\left( c_{i}-1 \right)+\left( \frac{\pi_{I}}{1-\pi_{I}} \right)\frac{\sqrt{\pi_{I}\pi_{C}\left( 1-\pi_{I} \right)\left( 1-\pi_{C} \right)}}{\left( 1-\pi_{I} \right)\left( 1-\pi_{C} \right)}\rho\sum_{i=1}^{M} t_{i}c_{i}$$

$$E=\frac{\pi_{I}}{1-\pi_{I}}B-\frac{\pi_{I}}{1-\pi_{I}}C$$

$$=\frac{\pi_{I}}{1-\pi_{I}}\left[ \frac{N}{2}\left( \frac{\pi_{I}}{1-\pi_{I}} \right)+\frac{\pi_{I}}{1-\pi_{I}}\rho\sum_{i=1}^{M} t_{i}\left( t_{i}-1 \right)+\frac{\sqrt{\pi_{I}\pi_{C}\left( 1-\pi_{I} \right)\left( 1-\pi_{C} \right)}}{\left( 1-\pi_{I} \right)\left( 1-\pi_{C} \right)}\rho\sum_{i=1}^{M} t_{i}c_{i} \right]-\frac{\pi_{I}}{1-\pi_{I}}\left[ \frac{N}{2}\left( \frac{\pi_{I}}{1-\pi_{I}} \right)+\frac{\pi_{I}}{1-\pi_{I}}\rho\sum_{i=1}^{M} t_{i}\left( t_{i}-1 \right) \right]$$

$$=\left( \frac{\pi_{I}}{1-\pi_{I}} \right)\frac{\sqrt{\pi_{I}\pi_{C}\left( 1-\pi_{I} \right)\left( 1-\pi_{C} \right)}}{\left( 1-\pi_{I} \right)\left( 1-\pi_{C} \right)}\rho\sum_{i=1}^{M} t_{i}c_{i}$$

$$F=\frac{-\pi_{I}}{1-\pi_{I}}A+\left[ \frac{\pi_{I}}{1-\pi_{I}}+\frac{\pi_{C}}{1-\pi_{C}} \right]B$$

$$=\frac{-\pi_{I}}{1-\pi_{I}}\left[ \frac{N}{2}\left( \frac{\pi_{I}}{1-\pi_{I}}+\frac{\pi_{C}}{1-\pi_{C}} \right)+\frac{\pi_{I}}{1-\pi_{I}}\rho\sum_{i=1}^{M} t_{i}\left( t_{i}-1 \right)+\frac{\pi_{C}}{1-\pi_{C}}\rho\sum_{i=1}^{M} c_{i}\left( c_{i}-1 \right)+\frac{2\sqrt{\pi_{I}\pi_{C}\left( 1-\pi_{I} \right)\left( 1-\pi_{C} \right)}}{\left( 1-\pi_{I} \right)\left( 1-\pi_{C} \right)}\rho\sum_{i=1}^{M} t_{i}c_{i} \right]+\left[ \frac{\pi_{I}}{1-\pi_{I}}+\frac{\pi_{C}}{1-\pi_{C}} \right]\left[ \frac{N}{2}\left( \frac{\pi_{I}}{1-\pi_{I}} \right)+\frac{\pi_{I}}{1-\pi_{I}}\rho\sum_{i=1}^{M} t_{i}\left( t_{i}-1 \right)+\frac{\sqrt{\pi_{I}\pi_{C}\left( 1-\pi_{I} \right)\left( 1-\pi_{C} \right)}}{\left( 1-\pi_{I} \right)\left( 1-\pi_{C} \right)}\rho\sum_{i=1}^{M} t_{i}c_{i} \right]$$

$$=\frac{\pi_{I}\pi_{C}}{\left( 1-\pi_{I} \right)\left( 1-\pi_{C} \right)}\rho\sum_{i=1}^{M} t_{i}\left( t_{i}-1 \right)-\frac{\pi_{I}\pi_{C}}{\left( 1-\pi_{I} \right)\left( 1-\pi_{C} \right)}\rho\sum_{i=1}^{M} c_{i}\left( c_{i}-1 \right)-\left( \frac{\pi_{I}}{1-\pi_{I}}-\frac{\pi_{C}}{1-\pi_{C}} \right)\frac{\sqrt{\pi_{I}\pi_{C}\left( 1-\pi_{I} \right)\left( 1-\pi_{C} \right)}}{\left( 1-\pi_{I} \right)\left( 1-\pi_{C} \right)}\rho\sum_{i=1}^{M} t_{i}c_{i}$$

$$G=\frac{-\pi_{I}}{1-\pi_{I}}B+\left[ \frac{\pi_{I}}{1-\pi_{I}}+\frac{\pi_{C}}{1-\pi_{C}} \right]C$$

$$=\frac{-\pi_{I}}{1-\pi_{I}}\left[ \frac{N}{2}\left( \frac{\pi_{I}}{1-\pi_{I}} \right)+\frac{\pi_{I}}{1-\pi_{I}}\rho\sum_{i=1}^{M} t_{i}\left( t_{i}-1 \right)+\frac{\sqrt{\pi_{I}\pi_{C}\left( 1-\pi_{I} \right)\left( 1-\pi_{C} \right)}}{\left( 1-\pi_{I} \right)\left( 1-\pi_{C} \right)}\rho\sum_{i=1}^{M} t_{i}c_{i} \right]+\left[ \frac{\pi_{I}}{1-\pi_{I}}+\frac{\pi_{C}}{1-\pi_{C}} \right]\left[ \frac{N}{2}\left( \frac{\pi_{I}}{1-\pi_{I}} \right)+\frac{\pi_{I}}{1-\pi_{I}}\rho\sum_{i=1}^{M} t_{i}\left( t_{i}-1 \right) \right]$$

$$=\frac{N\pi_{I}\pi_{C}}{2\left( 1-\pi_{I} \right)\left( 1-\pi_{C} \right)}+\frac{\pi_{I}\pi_{C}}{\left( 1-\pi_{I} \right)\left( 1-\pi_{C} \right)}\rho\sum_{i=1}^{M} t_{i}\left( t_{i}-1 \right)-\left( \frac{\pi_{I}}{1-\pi_{I}} \right)\frac{\sqrt{\pi_{I}\pi_{C}\left( 1-\pi_{I} \right)\left( 1-\pi_{C} \right)}}{\left( 1-\pi_{I} \right)\left( 1-\pi_{C} \right)}\rho\sum_{i=1}^{M} t_{i}c_{i}$$

$$\Rightarrow\mathrm{cov}\left( \hat{\boldsymbol{\beta}} \right)=\frac{4\left( 1-\pi_{I} \right)^{2}\left( 1-\pi_{C} \right)^{2}}{\left( N\pi_{I}\pi_{C} \right)^{2}}\left[ \begin{matrix} D & E \\ F & G \end{matrix} \right]\left[ \begin{matrix} \frac{\pi_{I}}{1-\pi_{I}} & \frac{-\pi_{I}}{1-\pi_{I}} \\ \frac{-\pi_{I}}{1-\pi_{I}} & \frac{\pi_{I}}{1-\pi_{I}}+\frac{\pi_{C}}{1-\pi_{C}} \end{matrix} \right] = \frac{4\left( 1-\pi_{I} \right)^{2}\left( 1-\pi_{C} \right)^{2}}{\left( N\pi_{I}\pi_{C} \right)^{2}} \left[ \begin{matrix} \frac{\pi_{I}}{1-\pi_{I}}D-\frac{\pi_{I}}{1-\pi_{I}}E & \frac{-\pi_{I}}{1-\pi_{I}}D+\left[ \frac{\pi_{I}}{1-\pi_{I}}+\frac{\pi_{C}}{1-\pi_{C}} \right]E \\ \frac{\pi_{I}}{1-\pi_{I}}F-\frac{\pi_{I}}{1-\pi_{I}}G & \frac{-\pi_{I}}{1-\pi_{I}}F+\left[ \frac{\pi_{I}}{1-\pi_{I}}+\frac{\pi_{C}}{1-\pi_{C}} \right]G \end{matrix} \right]$$

$$\mathrm{var}\left( \hat{\beta}_{1} \right)= \frac{4\left( 1-\pi_{I} \right)^{2}\left( 1-\pi_{C} \right)^{2}}{\left( N\pi_{I}\pi_{C} \right)^{2}}H$$

where $H=\frac{-\pi_{I}}{1-\pi_{I}}F+\left[ \frac{\pi_{I}}{1-\pi_{I}}+\frac{\pi_{C}}{1-\pi_{C}} \right]G$

$$H=\frac{-\pi_{I}}{1-\pi_{I}}F+\left[ \frac{\pi_{I}}{1-\pi_{I}}+\frac{\pi_{C}}{1-\pi_{C}} \right]G$$

$$=\frac{-\pi_{I}}{1-\pi_{I}}\left[ \frac{\pi_{I}\pi_{C}}{\left( 1-\pi_{I} \right)\left( 1-\pi_{C} \right)}\rho\sum_{i=1}^{M} t_{i}\left( t_{i}-1 \right)-\frac{\pi_{I}\pi_{C}}{\left( 1-\pi_{I} \right)\left( 1-\pi_{C} \right)}\rho\sum_{i=1}^{M} c_{i}\left( c_{i}-1 \right)-\left( \frac{\pi_{I}}{1-\pi_{I}}-\frac{\pi_{C}}{1-\pi_{C}} \right)\frac{\sqrt{\pi_{I}\pi_{C}\left( 1-\pi_{I} \right)\left( 1-\pi_{C} \right)}}{\left( 1-\pi_{I} \right)\left( 1-\pi_{C} \right)}\rho\sum_{i=1}^{M} t_{i}c_{i} \right]+\left[ \frac{\pi_{I}}{1-\pi_{I}}+\frac{\pi_{C}}{1-\pi_{C}} \right]\left[ \frac{N\pi_{I}\pi_{C}}{2\left( 1-\pi_{I} \right)\left( 1-\pi_{C} \right)}+\frac{\pi_{I}\pi_{C}}{\left( 1-\pi_{I} \right)\left( 1-\pi_{C} \right)}\rho\sum_{i=1}^{M} t_{i}\left( t_{i}-1 \right)-\left( \frac{\pi_{I}}{1-\pi_{I}} \right)\frac{\sqrt{\pi_{I}\pi_{C}\left( 1-\pi_{I} \right)\left( 1-\pi_{C} \right)}}{\left( 1-\pi_{I} \right)\left( 1-\pi_{C} \right)}\rho\sum_{i=1}^{M} t_{i}c_{i} \right]$$

$$=\frac{N}{2}\left( \frac{\pi_{I}^{2}\pi_{C}\left( 1-\pi_{C} \right)+\pi_{I}\pi_{C}^{2}\left( 1-\pi_{I} \right)}{\left( 1-\pi_{I} \right)^{2}\left( 1-\pi_{C} \right)^{2}} \right)+\frac{\pi_{I}\pi_{C}^{2}}{\left( 1-\pi_{I} \right)\left( 1-\pi_{C} \right)^{2}}\rho\sum_{i=1}^{M} t_{i}\left( t_{i}-1 \right)+\frac{\pi_{I}^{2}\pi_{C}}{\left( 1-\pi_{I} \right)^{2}\left( 1-\pi_{C} \right)}\rho\sum_{i=1}^{M} c_{i}\left( c_{i}-1 \right)-\frac{2\pi_{I}\pi_{C}\sqrt{\pi_{I}\pi_{C}\left( 1-\pi_{I} \right)\left( 1-\pi_{C} \right)}}{\left( 1-\pi_{I} \right)^{2}\left( 1-\pi_{C} \right)^{2}}\rho\sum_{i=1}^{M} t_{i}c_{i}$$

$$\Rightarrow\mathrm{var}\left( \hat{\beta}_{1} \right)= \frac{4\left( 1-\pi_{I} \right)^{2}\left( 1-\pi_{C} \right)^{2}}{\left( N\pi_{I}\pi_{C} \right)^{2}}\left( \frac{N}{2}\left( \frac{\pi_{I}^{2}\pi_{C}\left( 1-\pi_{C} \right)+\pi_{I}\pi_{C}^{2}\left( 1-\pi_{I} \right)}{\left( 1-\pi_{I} \right)^{2}\left( 1-\pi_{C} \right)^{2}} \right)+\frac{\pi_{I}\pi_{C}^{2}}{\left( 1-\pi_{I} \right)\left( 1-\pi_{C} \right)^{2}}\rho\sum_{i=1}^{M} t_{i}\left( t_{i}-1 \right)+\frac{\pi_{I}^{2}\pi_{C}}{\left( 1-\pi_{I} \right)^{2}\left( 1-\pi_{C} \right)}\rho\sum_{i=1}^{M} c_{i}\left( c_{i}-1 \right)-\frac{2\pi_{I}\pi_{C}\sqrt{\pi_{I}\pi_{C}\left( 1-\pi_{I} \right)\left( 1-\pi_{C} \right)}}{\left( 1-\pi_{I} \right)^{2}\left( 1-\pi_{C} \right)^{2}}\rho\sum_{i=1}^{M} t_{i}c_{i} \right)$$

$$=\frac{4\left( 1-\pi_{I} \right)\left( 1-\pi_{C} \right)}{N^{2}\pi_{I}\pi_{C}}\left( \frac{{N(\pi}_{I}\left( 1-\pi_{C} \right)+\pi_{C}\left( 1-\pi_{I} \right)}{2\left( 1-\pi_{I} \right)\left( 1-\pi_{C} \right)}+\frac{\pi_{C}}{1-\pi_{C}}\rho\sum_{i=1}^{M} t_{i}\left( t_{i}-1 \right)+\frac{\pi_{I}}{1-\pi_{I}}\rho\sum_{i=1}^{M} c_{i}\left( c_{i}-1 \right)-\frac{2\sqrt{\pi_{I}\pi_{C}\left( 1-\pi_{I} \right)\left( 1-\pi_{C} \right)}}{\left( 1-\pi_{I} \right)\left( 1-\pi_{C} \right)}\rho\sum_{i=1}^{M} t_{i}c_{i} \right)$$

$$\Rightarrow DEFF=\frac{4\left( 1-\pi_{I} \right)\left( 1-\pi_{C} \right)}{N^{2}\pi_{I}\pi_{C}}\left( \frac{N\left( \pi_{I}\left( 1-\pi_{C} \right)+\pi_{C}\left( 1-\pi_{I} \right) \right)}{2\left( 1-\pi_{I} \right)\left( 1-\pi_{C} \right)}+\frac{\pi_{C}}{1-\pi_{C}}\rho\sum_{i=1}^{M} t_{i}\left( t_{i}-1 \right)+\frac{\pi_{I}}{1-\pi_{I}}\rho\sum_{i=1}^{M} c_{i}\left( c_{i}-1 \right)-\frac{2\sqrt{\pi_{I}\pi_{C}\left( 1-\pi_{I} \right)\left( 1-\pi_{C} \right)}}{\left( 1-\pi_{I} \right)\left( 1-\pi_{C} \right)}\rho\sum_{i=1}^{M} t_{i}c_{i} \right)\times\frac{N\pi_{I}\pi_{C}}{2\left( \pi_{I}\left( 1-\pi_{C} \right)+\pi_{C}\left( 1-\pi_{I} \right) \right)}$$

$$=\frac{2\left( 1-\pi_{I} \right)\left( 1-\pi_{C} \right)}{N\left( \pi_{I}\left( 1-\pi_{C} \right)+\pi_{C}\left( 1-\pi_{I} \right) \right)}\left( \frac{N\left( \pi_{I}\left( 1-\pi_{C} \right)+\pi_{C}\left( 1-\pi_{I} \right) \right)}{2\left( 1-\pi_{I} \right)\left( 1-\pi_{C} \right)}+\frac{\pi_{C}}{1-\pi_{C}}\rho\sum_{i=1}^{M} t_{i}\left( t_{i}-1 \right)+\frac{\pi_{I}}{1-\pi_{I}}\rho\sum_{i=1}^{M} c_{i}\left( c_{i}-1 \right)-\frac{2\sqrt{\pi_{I}\pi_{C}\left( 1-\pi_{I} \right)\left( 1-\pi_{C} \right)}}{\left( 1-\pi_{I} \right)\left( 1-\pi_{C} \right)}\rho\sum_{i=1}^{M} t_{i}c_{i} \right)$$

$$=1+\rho\left[ \frac{2}{N\left( \pi_{I}\left( 1-\pi_{C} \right)+\pi_{C}\left( 1-\pi_{I} \right) \right)}\left( \pi_{C}\left( 1-\pi_{I} \right)\sum_{i=1}^{M} t_{i}\left( t_{i}-1 \right)+\pi_{I}\left( 1-\pi_{C} \right)\sum_{i=1}^{M} c_{i}\left( c_{i}-1 \right)-2\sqrt{\pi_{I}\pi_{C}\left( 1-\pi_{I} \right)\left( 1-\pi_{C} \right)}\sum_{i=1}^{M} t_{i}c_{i} \right) \right]$$

- - 1. Cluster randomisation

As shown in section 3.1.1:

$$\frac{1}{N}\sum_{i=1}^{M} t_{i}\left( t_{i}-1 \right)=\frac{1}{2}\sum_{k=1}^{K} {\left( k-1 \right)\gamma}_{k}$$

$$\frac{1}{N}\sum_{i=1}^{M} c_{i}\left( c_{i}-1 \right)=\frac{1}{2}\sum_{k=1}^{K} {\left( k-1 \right)\gamma}_{k}$$

$$\frac{1}{N}\sum_{i=1}^{M} {t_{i}c}_{i}=0$$

$$DEFF= 1+\rho\left[ \frac{2}{N\left( \pi_{I}\left( 1-\pi_{C} \right)+\pi_{C}\left( 1-\pi_{I} \right) \right)}\left( \pi_{C}\left( 1-\pi_{I} \right)\sum_{i=1}^{M} t_{i}\left( t_{i}-1 \right)+\pi_{I}\left( 1-\pi_{C} \right)\sum_{i=1}^{M} c_{i}\left( c_{i}-1 \right)-2\sqrt{\pi_{I}\pi_{C}\left( 1-\pi_{I} \right)\left( 1-\pi_{C} \right)}\sum_{i=1}^{M} t_{i}c_{i} \right) \right]$$

$$=1+\rho\left[ \frac{2}{\pi_{I}\left( 1-\pi_{C} \right)+\pi_{C}\left( 1-\pi_{I} \right)}\left( \pi_{C}\left( 1-\pi_{I} \right)\left( \frac{1}{2} \right)\sum_{k=1}^{K} {\left( k-1 \right)\gamma}_{k}+\pi_{I}\left( 1-\pi_{C} \right)\left( \frac{1}{2} \right)\sum_{k=1}^{K} {\left( k-1 \right)\gamma}_{k} \right) \right]$$

$$=1+\rho\left[ \frac{1}{\pi_{I}\left( 1-\pi_{C} \right)+\pi_{C}\left( 1-\pi_{I} \right)}\left( \pi_{C}\left( 1-\pi_{I} \right)+\pi_{I}\left( 1-\pi_{C} \right) \right)\sum_{k=1}^{K} {\left( k-1 \right)\gamma}_{k} \right]=1+\rho\sum_{k=1}^{K} {\left( k-1 \right)\gamma}_{k}$$

- - 1. Individual randomisation

As shown in section 3.1.2:

$$\frac{1}{N}\sum_{i=1}^{M} t_{i}\left( t_{i}-1 \right)=\frac{1}{4}\sum_{k=1}^{K} \left( k-1 \right)\gamma_{k}$$

$$\frac{1}{N}\sum_{i=1}^{M} c_{i}\left( c_{i}-1 \right)=\frac{1}{4}\sum_{k=1}^{K} \left( k-1 \right)\gamma_{k}$$

$$\frac{1}{N}\sum_{i=1}^{M} t_{i}c_{i}=\frac{1}{4}\sum_{k=1}^{K} \left( k-1 \right)\gamma_{k}$$

$$DEFF= 1+\rho\left[ \frac{2}{N\left( \pi_{I}\left( 1-\pi_{C} \right)+\pi_{C}\left( 1-\pi_{I} \right) \right)}\left( \pi_{C}\left( 1-\pi_{I} \right)\sum_{i=1}^{M} t_{i}\left( t_{i}-1 \right)+\pi_{I}\left( 1-\pi_{C} \right)\sum_{i=1}^{M} c_{i}\left( c_{i}-1 \right)-2\sqrt{\pi_{I}\pi_{C}\left( 1-\pi_{I} \right)\left( 1-\pi_{C} \right)}\sum_{i=1}^{M} t_{i}c_{i} \right) \right]$$

$$=1+\rho\left[ \frac{2}{N\left( \pi_{I}\left( 1-\pi_{C} \right)+\pi_{C}\left( 1-\pi_{I} \right) \right)}\left( \pi_{C}\left( 1-\pi_{I} \right)\left( \frac{1}{4} \right)\sum_{k=1}^{K} \left( k-1 \right)\gamma_{k}+\pi_{I}\left( 1-\pi_{C} \right)\left( \frac{1}{4} \right)\sum_{k=1}^{K} \left( k-1 \right)\gamma_{k}-2\sqrt{\pi_{I}\pi_{C}\left( 1-\pi_{I} \right)\left( 1-\pi_{C} \right)}\left( \frac{1}{4} \right)\sum_{k=1}^{K} \left( k-1 \right)\gamma_{k} \right) \right]$$

$$=1+\rho\left[ \frac{1}{\pi_{I}\left( 1-\pi_{C} \right)+\pi_{C}\left( 1-\pi_{I} \right)}\left( \left( \frac{1}{2}\left[ \pi_{C}\left( 1-\pi_{I} \right)+\pi_{I}\left( 1-\pi_{C} \right) \right]-\sqrt{\pi_{I}\pi_{C}\left( 1-\pi_{I} \right)\left( 1-\pi_{C} \right)} \right)\sum_{k=1}^{K} \left( k-1 \right)\gamma_{k} \right) \right]$$

$$=1+\rho\left[ \left( \frac{1}{2}-\frac{\sqrt{\pi_{I}\pi_{C}\left( 1-\pi_{I} \right)\left( 1-\pi_{C} \right)}}{\pi_{I}\left( 1-\pi_{C} \right)+\pi_{C}\left( 1-\pi_{I} \right)} \right)\sum_{k=1}^{K} \left( k-1 \right)\gamma_{k} \right]$$

- 1. **Exchangeable working correlation structure**

$$\mathrm{cov} \left( \hat{\boldsymbol{\beta}} \right)=\left[ \sum_{i=1}^{M} \mathbf{D}_{i}^{T}\mathbf{V}_{i}^{-1}\mathbf{D}_{i} \right]^{-1}$$

For the independent observations (i.e. $n_{i}=1 \left( j subscript dropped for convenience \right), X_{i}=0 \left( \mathrm{control} \right) \mathrm{or} 1\left( \mathrm{intervention} \right))$:

$\mathbf{R}_{i}=\mathbf{C}_{i}=1$, $\mathbf{A}_{i}^{1/2}=\sqrt{\mu_{i}(1-\mu_{i})}\Rightarrow$ $\mathbf{V}_{i}= \mathbf{A}_{i}^{1/2}{\mathbf{R}_{i}\mathbf{A}}_{i}^{1/2}=\mu_{i}(1-\mu_{i})\Rightarrow\mathbf{V}_{i}^{-1}=1/\mu_{i}(1-\mu_{i})$

$$\frac{\partial\mu_{i}}{\partial\beta_{0}}= \exp\left( \beta_{0}+\beta_{1}X_{i} \right)=\mu_{i}$$

$$\frac{\partial\mu_{i}}{\partial\beta_{1}}=X_{i}\exp\left( \beta_{0}+\beta_{1}X_{i} \right) = {X_{i}\mu}_{i}$$

$$\Rightarrow\mathbf{D}_{i}=\left[ \begin{matrix} \frac{\partial\mu_{i}}{\partial\beta_{0}} & \frac{\partial\mu_{i}}{\partial\beta_{1}} \end{matrix} \right]=\left[ \begin{matrix} \mu_{i} & {X_{i}\mu}_{i} \end{matrix} \right]$$

$$\mathbf{D}_{i}^{T}\mathbf{V}_{i}^{-1}\mathbf{D}_{i}= \left[ \begin{aligned} \mu_{i} \\ {X_{i}\mu}_{i} \end{aligned} \right]\frac{1}{\mu_{i}\left( 1-\mu_{i} \right)}\left[ \begin{matrix} \mu_{i} & {X_{i}\mu}_{i} \end{matrix} \right] = \frac{1}{\mu_{i}\left( 1-\mu_{i} \right)}\left[ \begin{matrix} {\mu_{i}}^{2} & X_{i}{\mu_{i}}^{2} \\ X_{i}{\mu_{i}}^{2} & X_{i}^{2}{\mu_{i}}^{2} \end{matrix} \right] = \left[ \begin{matrix} \frac{\mu_{i}}{1-\mu_{i}} & \frac{X_{i}\mu_{i}}{1-\mu_{i}} \\ \frac{X_{i}\mu_{i}}{1-\mu_{i}} & \frac{X_{i}^{2}\mu_{i}}{1-\mu_{i}} \end{matrix} \right]$$

For a cluster of size $n_{i}>1$with $t_{i}$ members assigned to the treatment group and $c_{i}$ members assigned to the control group:

$$\mathbf{R}_{i}=\mathbf{C}_{i}= \left[ \begin{matrix} 1 & \rho& \cdots& \rho\\ \rho& 1 & \ldots& \rho\\ \vdots& \vdots& \ddots& \vdots\\ \rho& \rho& \cdots& 1 \end{matrix} \right], a n_{i}\times n_{i}\mathrm{matrix}$$

$\mathbf{A}_{i}^{1/2}=\left[ \begin{matrix} \sqrt{\mu_{i1}(1-\mu_{i1})} & 0 & \cdots& 0 \\ 0 & \sqrt{\mu_{i2}(1-\mu_{i2})} & \ldots& 0 \\ \vdots& \vdots& \ddots& \vdots\\ 0 & 0 & \cdots& \sqrt{\mu_{{in}_{i}}(1-\mu_{{in}_{i}})} \end{matrix} \right]$ , a $n_{i}\times n_{i}$ matrix

$$\mathbf{V}_{i}= \mathbf{A}_{i}^{1/2}{\mathbf{R}_{i}\mathbf{A}}_{i}^{1/2}=\left[ \begin{matrix} \sqrt{\mu_{i1}\left( 1-\mu_{i1} \right)} & 0 & \cdots& 0 \\ 0 & \sqrt{\mu_{i2}\left( 1-\mu_{i2} \right)} & \ldots& 0 \\ \vdots& \vdots& \ddots& \vdots\\ 0 & 0 & \cdots& \sqrt{\mu_{{in}_{i}}\left( 1-\mu_{in_{i}} \right)} \end{matrix} \right]\left[ \begin{matrix} 1 & \rho& \cdots& \rho\\ \rho& 1 & \ldots& \rho\\ \vdots& \vdots& \ddots& \vdots\\ \rho& \rho& \cdots& 1 \end{matrix} \right]\left[ \begin{matrix} \sqrt{\mu_{i1}\left( 1-\mu_{i1} \right)} & 0 & \cdots& 0 \\ 0 & \sqrt{\mu_{i2}\left( 1-\mu_{i2} \right)} & \ldots& 0 \\ \vdots& \vdots& \ddots& \vdots\\ 0 & 0 & \cdots& \sqrt{\mu_{in_{i}}\left( 1-\mu_{{in}_{i}} \right)} \end{matrix} \right]$$

$$=\left[ \begin{matrix} \mu_{i1}\left( 1-\mu_{i1} \right) & \rho\sqrt{\mu_{i1}\mu_{i2}\left( 1-\mu_{i1} \right)\left( 1-\mu_{i2} \right)} & \cdots& \rho\sqrt{\mu_{i1}\mu_{{in}_{i}}\left( 1-\mu_{i1} \right)\left( 1-\mu_{in_{i}} \right)} \\ \rho\sqrt{\mu_{i1}\mu_{i2}\left( 1-\mu_{i1} \right)\left( 1-\mu_{i2} \right)} & \mu_{i2}\left( 1-\mu_{i2} \right) & \ldots& \rho\sqrt{\mu_{i2}\mu_{in_{i}}\left( 1-\mu_{i2} \right)\left( 1-\mu_{{in}_{i}} \right)} \\ \vdots& \vdots& \ddots& \vdots\\ \rho\sqrt{\mu_{i1}\mu_{{in}_{i}}\left( 1-\mu_{i1} \right)\left( 1-\mu_{{in}_{i}} \right)} & \rho\sqrt{\mu_{i2}\mu_{{in}_{i}}\left( 1-\mu_{i2} \right)\left( 1-\mu_{{in}_{i}} \right)} & \cdots& \mu_{in_{i}}\left( 1-{\mu i}_{n_{i}} \right) \end{matrix} \right]$$

Using the properties of non-singular matrices, $\mathbf{V}_{i}^{\mathbf{-1}}= \left[ \mathbf{A}_{i}^{1/2}{\mathbf{R}_{i}\mathbf{A}}_{i}^{1/2} \right]^{-1}=\left[ \mathbf{A}_{i}^{1/2} \right]^{-1}\mathbf{R}_{i}^{-1}\left[ \mathbf{A}_{i}^{1/2} \right]^{-1}$

$$\left[ \mathbf{A}_{i}^{1/2} \right]^{-1}=\left[ \begin{matrix} \left[ \sqrt{\mu_{i1}\left( 1-\mu_{i1} \right)} \right]^{-1} & 0 & \cdots& 0 \\ 0 & \left[ \sqrt{\mu_{i2}\left( 1-\mu_{i2} \right)} \right]^{-1} & \ldots& 0 \\ \vdots& \vdots& \ddots& \vdots\\ 0 & 0 & \cdots& \left[ \sqrt{\mu_{{in}_{i}}\left( 1-\mu_{{in}_{i}} \right)} \right]^{-1} \end{matrix} \right]$$

Using the McDaniel et al (2013) expression for the inverse of an exchangeable matrix, $\mathbf{R}_{i}^{-1}$ can be written as:

$\mathbf{R}_{i}^{-1}=\left( a-b \right)\mathbf{I}_{n_{i}}+b\mathbf{J}_{n_{i}},$ where $a=\frac{1+\left( n_{i}-2 \right)\rho}{(1-\rho)(1+\left( n_{i}-1 \right)\rho)}$, $b=\frac{-\rho}{(1-\rho)(1+\left( n_{i}-1 \right)\rho)}$, $\mathbf{I}_{n_{i}}$ is the $n_{i}\times n_{i}$ identity matrix, and $\mathbf{J}_{n_{i}}$is the $n_{i}\times n_{i}$ matrix of 1s

$$\mathbf{R}_{i}^{-1}=\left( \frac{1+\left( n_{i}-2 \right)\rho}{\left( 1-\rho\right)\left( 1+\left( n_{i}-1 \right)\rho\right)}+\frac{\rho}{\left( 1-\rho\right)\left( 1+\left( n_{i}-1 \right)\rho\right)} \right)\mathbf{I}_{n_{i}}- \frac{\rho}{(1-\rho)(1+\left( n_{i}-1 \right)\rho}\mathbf{J}_{n_{i}}$$

$$= \frac{1}{\left( 1-\rho\right)\left( 1+\left( n_{i}-1 \right)\rho\right)}\left( (1+\left( n_{i}-1 \right)\rho)\left[ \begin{matrix} 1 & 0 & \cdots& 0 \\ 0 & 1 & \ldots& 0 \\ \vdots& \vdots& \ddots& \vdots\\ 0 & 0 & \cdots& 1 \end{matrix} \right]-\left[ \begin{matrix} \rho& \rho& \cdots& \rho\\ \rho& \rho& \ldots& \rho\\ \vdots& \vdots& \ddots& \vdots\\ \rho& \rho& \cdots& \rho\end{matrix} \right] \right)$$

$$= \frac{1}{\left( 1-\rho\right)\left( 1+\left( n_{i}-1 \right)\rho\right)}\left[ \begin{matrix} 1+\left( n_{i}-2 \right)\rho& -\rho& \cdots& -\rho\\ -\rho& 1+\left( n_{i}-2 \right)\rho& \ldots& -\rho\\ \vdots& \vdots& \ddots& \vdots\\ -\rho& -\rho& \cdots& 1+\left( n_{i}-2 \right)\rho\end{matrix} \right]$$

$$\Rightarrow\mathbf{V}_{i}^{-1}=\left[ \begin{matrix} \left[ \sqrt{\mu_{i1}\left( 1-\mu_{i1} \right)} \right]^{-1} & 0 & \cdots& 0 \\ 0 & \left[ \sqrt{\mu_{i2}\left( 1-\mu_{i2} \right)} \right]^{-1} & \ldots& 0 \\ \vdots& \vdots& \ddots& \vdots\\ 0 & 0 & \cdots& \left[ \sqrt{\mu_{{in}_{i}}\left( 1-\mu_{{in}_{i}} \right)} \right]^{-1} \end{matrix} \right]\frac{1}{\left( 1-\rho\right)\left( 1+\left( n_{i}-1 \right)\rho\right)}\times\left[ \begin{matrix} 1+\left( n_{i}-2 \right)\rho& -\rho& \cdots& -\rho\\ -\rho& 1+\left( n_{i}-2 \right)\rho& \ldots& -\rho\\ \vdots& \vdots& \ddots& \vdots\\ -\rho& -\rho& \cdots& 1+\left( n_{i}-2 \right)\rho\end{matrix} \right]\left[ \begin{matrix} \left[ \sqrt{\mu_{i1}\left( 1-\mu_{i1} \right)} \right]^{-1} & 0 & \cdots& 0 \\ 0 & \left[ \sqrt{\mu_{i2}\left( 1-\mu_{i2} \right)} \right]^{-1} & \ldots& 0 \\ \vdots& \vdots& \ddots& \vdots\\ 0 & 0 & \cdots& \left[ \sqrt{\mu_{{in}_{i}}\left( 1-\mu_{{in}_{i}} \right)} \right]^{-1} \end{matrix} \right]$$

$$=\frac{1}{\left( 1-\rho\right)\left( 1+\left( n_{i}-1 \right)\rho\right)}\left[ \begin{matrix} \frac{1+\left( n_{i}-2 \right)\rho}{\sqrt{\mu_{i1}\left( 1-\mu_{i1} \right)}} & \frac{-\rho}{\sqrt{\mu_{i1}\left( 1-\mu_{i1} \right)}} & \cdots& \frac{-\rho}{\sqrt{\mu_{i1}\left( 1-\mu_{i1} \right)}} \\ \frac{-\rho}{\sqrt{\mu_{i2}\left( 1-\mu_{i2} \right)}} & \frac{1+\left( n_{i}-2 \right)\rho}{\sqrt{\mu_{i2}\left( 1-\mu_{i2} \right)}} & \ldots& \frac{-\rho}{\sqrt{\mu_{i2}\left( 1-\mu_{i2} \right)}} \\ \vdots& \vdots& \ddots& \vdots\\ \frac{-\rho}{\sqrt{\mu_{in_{i}}\left( 1-\mu_{{in}_{i}} \right)}} & \frac{-\rho}{\sqrt{\mu_{{in}_{i}}\left( 1-\mu_{{in}_{i}} \right)}} & \cdots& \frac{1+\left( n_{i}-2 \right)\rho}{\sqrt{\mu_{{in}_{i}}\left( 1-\mu_{{in}_{i}} \right)}} \end{matrix} \right]\left[ \begin{matrix} \left[ \sqrt{\mu_{i1}\left( 1-\mu_{i1} \right)} \right]^{-1} & 0 & \cdots& 0 \\ 0 & \left[ \sqrt{\mu_{i2}\left( 1-\mu_{i2} \right)} \right]^{-1} & \ldots& 0 \\ \vdots& \vdots& \ddots& \vdots\\ 0 & 0 & \cdots& \left[ \sqrt{\mu_{{in}_{i}}\left( 1-\mu_{{in}_{i}} \right)} \right]^{-1} \end{matrix} \right]$$

$$= \frac{1}{\left( 1-\rho\right)\left( 1+\left( n_{i}-1 \right)\rho\right)}\left[ \begin{matrix} \frac{1+\left( n_{i}-2 \right)\rho}{\mu_{i1}\left( 1-\mu_{i1} \right)} & \frac{-\rho}{\sqrt{\mu_{i1}\mu_{i2}\left( 1-\mu_{i1} \right)\left( 1-\mu_{i2} \right)}} & \cdots& \frac{-\rho}{\sqrt{\mu_{i1}\mu_{in_{i}}\left( 1-\mu_{i1} \right)\left( 1-\mu_{{in}_{i}} \right)}} \\ \frac{-\rho}{\sqrt{\mu_{i1}\mu_{i2}\left( 1-\mu_{i1} \right)\left( 1-\mu_{i2} \right)}} & \frac{1+\left( n_{i}-2 \right)\rho}{\mu_{i2}\left( 1-\mu_{i2} \right)} & \ldots& \frac{-\rho}{\sqrt{\mu_{i2}\mu_{{in}_{i}}\left( 1-\mu_{i2} \right)\left( 1-\mu_{in_{i}} \right)}} \\ \vdots& \vdots& \ddots& \vdots\\ \frac{-\rho}{\sqrt{\mu_{i1}\mu_{in_{i}}\left( 1-\mu_{i1} \right)\left( 1-\mu_{{in}_{i}} \right)}} & \frac{-\rho}{\sqrt{\mu_{i2}\mu_{in_{i}}\left( 1-\mu_{i2} \right)\left( 1-\mu_{in_{i}} \right)}} & \cdots& \frac{1+\left( n_{i}-2 \right)\rho}{\mu_{in_{i}}\left( 1-\mu_{in_{i}} \right)} \end{matrix} \right]$$

$$\mathbf{D}_{i}=\left[ \begin{matrix} \begin{matrix} \frac{\partial\mu_{i1}}{\partial\beta_{0}} \end{matrix} & \frac{\partial\mu_{i1}}{\partial\beta_{1}} \\ \vdots& \vdots\\ \frac{\partial\mu_{{in}_{i}}}{\partial\beta_{0}} & \frac{\partial\mu_{{in}_{i}}}{\partial\beta_{1}} \end{matrix} \right]=\left[ \begin{matrix} \begin{matrix} \mu_{i1} \end{matrix} & X_{i1}\mu_{i1} \\ \vdots& \vdots\\ \mu_{{in}_{i}} & X_{in_{i}}\mu_{in_{i}} \end{matrix} \right]$$

$$\mathbf{D}_{i}^{T}\mathbf{V}_{i}^{-1}\mathbf{D}_{i}= \left[ \begin{matrix} \begin{matrix} \mu_{i1} \end{matrix} & \ldots& \mu_{{in}_{i}} \\ X_{i1}\mu_{i1} & \ldots& X_{in_{i}}\mu_{in_{i}} \end{matrix} \right]\left( \frac{1}{\left( 1-\rho\right)\left( 1+\left( n_{i}-1 \right)\rho\right)} \right)$$

$$\times\left[ \begin{matrix} \frac{1+\left( n_{i}-2 \right)\rho}{\mu_{i1}\left( 1-\mu_{i1} \right)} & \frac{-\rho}{\sqrt{\mu_{i1}\mu_{i2}\left( 1-\mu_{i1} \right)\left( 1-\mu_{i2} \right)}} & \cdots& \frac{-\rho}{\sqrt{\mu_{i1}\mu_{in_{i}}\left( 1-\mu_{i1} \right)\left( 1-\mu_{{in}_{i}} \right)}} \\ \frac{-\rho}{\sqrt{\mu_{i1}\mu_{i2}\left( 1-\mu_{i1} \right)\left( 1-\mu_{i2} \right)}} & \frac{1+\left( n_{i}-2 \right)\rho}{\mu_{i2}\left( 1-\mu_{i2} \right)} & \ldots& \frac{-\rho}{\sqrt{\mu_{i2}\mu_{in_{i}}\left( 1-\mu_{i2} \right)\left( 1-\mu_{{in}_{i}} \right)}} \\ \vdots& \vdots& \ddots& \vdots\\ \frac{-\rho}{\sqrt{\mu_{i1}\mu_{{in}_{i}}\left( 1-\mu_{i1} \right)\left( 1-\mu_{{in}_{i}} \right)}} & \frac{-\rho}{\sqrt{\mu_{i2}\mu_{in_{i}}\left( 1-\mu_{i2} \right)\left( 1-\mu_{{in}_{i}} \right)}} & \cdots& \frac{1+\left( n_{i}-2 \right)\rho}{\mu_{in_{i}}\left( 1-\mu_{{in}_{i}} \right)} \end{matrix} \right]\left[ \begin{matrix} \begin{matrix} \mu_{i1} \end{matrix} & X_{i1}\mu_{i1} \\ \vdots& \vdots\\ \mu_{in_{i}} & X_{{in}_{i}}\mu_{{in}_{i}} \end{matrix} \right]$$

$$= \frac{1}{\left( 1-\rho\right)\left( 1+\left( n_{i}-1 \right)\rho\right)}\left[ \begin{matrix} \frac{1+\left( n_{i}-2 \right)\rho}{1-\mu_{i1}}-\rho\sum_{j\neq1} \frac{\mu_{ij}}{\sqrt{\mu_{i1}\mu_{ij}\left( 1-\mu_{i1} \right)\left( 1-\mu_{ij} \right)}} & \cdots& \frac{1+\left( n_{i}-2 \right)\rho}{1-\mu_{in_{i}}}-\rho\sum_{j\neq n_{i}} \frac{\mu_{ij}}{\sqrt{\mu_{{in}_{i}}\mu_{ij}\left( 1-\mu_{{in}_{i}} \right)\left( 1-\mu_{ij} \right)}} \\ \frac{X_{i1}\left( 1+\left( n_{i}-2 \right)\rho\right)}{1-\mu_{i1}}-\rho\sum_{j\neq1} \frac{X_{ij}\mu_{ij}}{\sqrt{\mu_{i1}\mu_{ij}\left( 1-\mu_{i1} \right)\left( 1-\mu_{ij} \right)}} & \cdots& \frac{X_{{in}_{i}}\left( 1+\left( n_{i}-2 \right)\rho\right)}{1-\mu_{{in}_{i}}}-\rho\sum_{j\neq n_{i}} \frac{X_{ij}\mu_{ij}}{\sqrt{\mu_{{in}_{i}}\mu_{ij}\left( 1-\mu_{{in}_{i}} \right)\left( 1-\mu_{ij} \right)}} \end{matrix} \right]$$

$$\times\left[ \begin{matrix} \begin{matrix} \mu_{i1} \end{matrix} & X_{i1}\mu_{i1} \\ \vdots& \vdots\\ \mu_{{in}_{i}} & X_{{in}_{i}}\mu_{{in}_{i}} \end{matrix} \right]$$

$$= \frac{1}{\left( 1-\rho\right)\left( 1+\left( n_{i}-1 \right)\rho\right)}\times\left[ \begin{matrix} \left( 1+\left( n_{i}-2 \right)\rho\right)\sum_{j=1}^{n_{i}} \begin{matrix} \frac{\mu_{ij}}{1-\mu_{ij}} \end{matrix}-2\rho\sum_{j=1}^{n_{i}} \sum_{a\neq j} \frac{\mu_{ij}\mu_{ia}}{\sqrt{\mu_{ij}\mu_{ia}\left( 1-\mu_{ij} \right)\left( 1-\mu_{ia} \right)}} & \left( 1+\left( n_{i}-2 \right)\rho\right)\sum_{j=1}^{n_{i}} \begin{matrix} \frac{X_{ij}\mu_{ij}}{1-\mu_{ij}} \end{matrix}-\rho\sum_{j=1}^{n_{i}} \sum_{a\neq j} \frac{\left( X_{ij}+X_{ia} \right)\mu_{ij}\mu_{ia}}{\sqrt{\mu_{ij}\mu_{ia}\left( 1-\mu_{ij} \right)\left( 1-\mu_{ia} \right)}} \\ \left( 1+\left( n_{i}-2 \right)\rho\right)\sum_{j=1}^{n_{i}} \begin{matrix} \frac{X_{ij}\mu_{ij}}{1-\mu_{ij}} \end{matrix}-\rho\sum_{j=1}^{n_{i}} \sum_{a\neq j} \frac{\left( X_{ij}+X_{ia} \right)\mu_{ij}\mu_{ia}}{\sqrt{\mu_{ij}\mu_{ia}\left( 1-\mu_{ij} \right)\left( 1-\mu_{ia} \right)}} & \left( 1+\left( n_{i}-2 \right)\rho\right)\sum_{j=1}^{n_{i}} \begin{matrix} \frac{X_{ij}^{2}\mu_{ij}}{1-\mu_{ij}} \end{matrix}-2\rho\sum_{j=1}^{n_{i}} \sum_{a\neq j} \frac{X_{ij}X_{ia}\mu_{ij}\mu_{ia}}{\sqrt{\mu_{ij}\mu_{ia}\left( 1-\mu_{ij} \right)\left( 1-\mu_{ia} \right)}} \end{matrix} \right]$$

Note that when $n_{i}=1$, this equals $\left[ \begin{matrix} \frac{\mu_{i1}}{1-\mu_{i1}} & \frac{X_{i1}\mu_{i1}}{1-\mu_{i1}} \\ \frac{X_{i1}\mu_{i1}}{1-\mu_{i1}} & \frac{X_{i1}^{2}\mu_{i1}}{1-\mu_{i1}} \end{matrix} \right]$ and therefore this expression for $\mathbf{D}_{i}^{T}\mathbf{V}_{i}^{-1}\mathbf{D}_{i}$ holds for all $n_{i}\geq1$.

The first sum in each element of this matrix can be written as the sum over cluster members assigned to intervention plus the sum over the cluster members assigned to control (e.g. $\sum_{j=1}^{n_{i}} \frac{\mu_{ij}}{1-\mu_{ij}}$ can be rewritten as $\sum_{j=1|X_{ij}=1}^{n_{i}} \frac{\mu_{ij}}{1-\mu_{ij}}+\sum_{j=1|X_{ij}=0}^{n_{i}} \frac{\mu_{ij}}{1-\mu_{ij}}$). A similar approach can be used for the double sums in each element of the matrix, by rewriting these as the sums of paired terms where the two cluster members are both assigned to intervention, both assigned to control, and one to intervention and one to control. For example, $\sum_{j=1}^{n_{i}} \sum_{a\neq j} \frac{\mu_{ij}\mu_{ia}}{\sqrt{\mu_{ij}\mu_{ia}\left( 1-\mu_{ij} \right)\left( 1-\mu_{ia} \right)}}=\sum_{j=1|X_{ij}=1}^{n_{i}} \sum_{a\neq j|X_{ia}=1} \frac{\mu_{ij}\mu_{ia}}{\sqrt{\mu_{ij}\mu_{ia}\left( 1-\mu_{ij} \right)\left( 1-\mu_{ia} \right)}}+\sum_{j=1|X_{ij}=0}^{n_{i}} \sum_{a\neq j|X_{ia}=0} \frac{\mu_{ij}\mu_{ia}}{\sqrt{\mu_{ij}\mu_{ia}\left( 1-\mu_{ij} \right)\left( 1-\mu_{ia} \right)}}+\sum_{j=1|X_{ij}=1}^{n_{i}} \sum_{a\neq j|X_{ia}=0} \frac{\mu_{ij}\mu_{ia}}{\sqrt{\mu_{ij}\mu_{ia}\left( 1-\mu_{ij} \right)\left( 1-\mu_{ia} \right)}}$. The sum over pairs where both are assigned to intervention is a sum of $\binom{t_{i}}{2}=t_{i}(t_{i}-1)/2$ terms, the sum over pairs where both are assigned to control is a sum of $\binom{c_{i}}{2}=c_{i}(c_{i}-1)/2$ terms, and the sum over pairs with one member assigned to intervention and one to control is a sum of $t_{i}c_{i}$ terms.

$\Rightarrow\mathbf{D}_{i}^{T}\mathbf{V}_{i}^{-1}\mathbf{D}_{i}=\frac{1}{\left( 1-\rho\right)\left( 1+\left( n_{i}-1 \right)\rho\right)} \left[ \begin{matrix} a & b \\ b & c \end{matrix} \right]$ , where

$$a=\left( 1+\left( n_{i}-2 \right)\rho\right)\sum_{j=1}^{n_{i}} \begin{matrix} \frac{\mu_{ij}}{1-\mu_{ij}} \end{matrix}-2\rho\sum_{j=1}^{n_{i}} \sum_{a\neq j} \frac{\mu_{ij}\mu_{ia}}{\sqrt{\mu_{ij}\mu_{ia}\left( 1-\mu_{ij} \right)\left( 1-\mu_{ia} \right)}}$$

$$=\left( 1+\left( n_{i}-2 \right)\rho\right)\left[ \frac{\pi_{I}}{1-\pi_{I}}t_{i}+\frac{\pi_{C}}{1-\pi_{C}}c_{i} \right]-2\rho\left[ \frac{\pi_{I}^{2}}{\sqrt{\pi_{I}^{2}\left( 1-\pi_{I} \right)^{2}}}\frac{t_{i}\left( t_{i}-1 \right)}{2}+\frac{\pi_{C}^{2}}{\sqrt{\pi_{C}^{2}\left( 1-\pi_{C} \right)^{2}}}\frac{c_{i}\left( c_{i}-1 \right)}{2}+\frac{\pi_{I}\pi_{C}}{\sqrt{\pi_{I}\pi_{C}\left( 1-\pi_{I} \right)\left( 1-\pi_{C} \right)}}t_{i}c_{i} \right]$$

$$=\frac{\pi_{I}}{1-\pi_{I}}\left( 1+\left( n_{i}-t_{i}-1 \right)\rho\right)t_{i}+\frac{\pi_{C}}{1-\pi_{C}}\left( 1+\left( n_{i}-c_{i}-1 \right)\rho\right)c_{i}-\frac{2\pi_{I}\pi_{C}}{\sqrt{\pi_{I}\pi_{C}\left( 1-\pi_{I} \right)\left( 1-\pi_{C} \right)}}\rho t_{i}c_{i}$$

$$=\frac{\pi_{I}}{1-\pi_{I}}\left( 1+\left( c_{i}-1 \right)\rho\right)t_{i}+\frac{\pi_{C}}{1-\pi_{C}}\left( 1+\left( t_{i}-1 \right)\rho\right)c_{i}-\frac{2\pi_{I}\pi_{C}}{\sqrt{\pi_{I}\pi_{C}\left( 1-\pi_{I} \right)\left( 1-\pi_{C} \right)}}\rho t_{i}c_{i}$$

$$=\frac{\pi_{I}}{1-\pi_{I}}\left( 1-\rho\right)t_{i}+\frac{\pi_{C}}{1-\pi_{C}}\left( 1-\rho\right)c_{i}+\left[ \frac{\pi_{I}}{1-\pi_{I}}+\frac{\pi_{C}}{1-\pi_{C}}-\frac{2\pi_{I}\pi_{C}}{\sqrt{\pi_{I}\pi_{C}\left( 1-\pi_{I} \right)\left( 1-\pi_{C} \right)}} \right] \rho t_{i}c_{i}$$

$$b=\left( 1+\left( n_{i}-2 \right)\rho\right)\sum_{j=1}^{n_{i}} \begin{matrix} \frac{X_{ij}\mu_{ij}}{1-\mu_{ij}} \end{matrix}-\rho\sum_{j=1}^{n_{i}} \sum_{a\neq j} \frac{\left( X_{ij}+X_{ia} \right)\mu_{ij}\mu_{ia}}{\sqrt{\mu_{ij}\mu_{ia}\left( 1-\mu_{ij} \right)\left( 1-\mu_{ia} \right)}}$$

$$=\left( 1+\left( n_{i}-2 \right)\rho\right)\frac{\left( 1 \right)\pi_{I}}{1-\pi_{I}}t_{i}-\rho\left[ \frac{\left( 1+1 \right)\pi_{I}^{2}}{\sqrt{\pi_{I}^{2}\left( 1-\pi_{I} \right)^{2}}}\left( \frac{t_{i}\left( t_{i}-1 \right)}{2} \right)+\frac{\left( 1+0 \right)\pi_{I}\pi_{C}}{\sqrt{\pi_{I}\pi_{C}\left( 1-\pi_{I} \right)\left( 1-\pi_{C} \right)}}t_{i}c_{i} \right]$$

$$=\left( 1+\left( n_{i}-2 \right)\rho\right)\frac{\pi_{I}}{1-\pi_{I}}t_{i}-\frac{\pi_{C}}{1-\pi_{C}}\rho t_{i}\left( t_{i}-1 \right)-\frac{\pi_{I}\pi_{C}}{\sqrt{\pi_{I}\pi_{C}\left( 1-\pi_{I} \right)\left( 1-\pi_{C} \right)}}\rho t_{i}c_{i}$$

$$=\frac{\pi_{I}}{1-\pi_{I}}\left( t_{i}+\rho t_{i}c_{i}-{\rho t}_{i} \right)-\frac{\pi_{I}\pi_{C}}{\sqrt{\pi_{I}\pi_{C}\left( 1-\pi_{I} \right)\left( 1-\pi_{C} \right)}}\rho t_{i}c_{i} = \frac{\pi_{I}}{1-\pi_{I}}\left( 1-\rho\right)t_{i}+\left[ \frac{\pi_{I}}{1-\pi_{I}}-\frac{\pi_{I}\pi_{C}}{\sqrt{\pi_{I}\pi_{C}\left( 1-\pi_{I} \right)\left( 1-\pi_{C} \right)}} \right]\rho t_{i}c_{i}$$

$$c=\left( 1+\left( n_{i}-2 \right)\rho\right)\sum_{j=1}^{n_{i}} \begin{matrix} \frac{X_{ij}^{2}\mu_{ij}}{1-\mu_{ij}} \end{matrix}-2\rho\sum_{j=1}^{n_{i}} \sum_{a\neq j} \frac{X_{ij}X_{ia}\mu_{ij}\mu_{ia}}{\sqrt{\mu_{ij}\mu_{ia}\left( 1-\mu_{ij} \right)\left( 1-\mu_{ia} \right)}} = \left( 1+\left( n_{i}-2 \right)\rho\right)\frac{{\left( 1 \right)^{2}\pi}_{I}}{1-\pi_{I}}t_{i}-2\rho\left[ \frac{\left( 1 \right)\left( 1 \right)\pi_{I}^{2}}{\sqrt{\pi_{I}^{2}\left( 1-\pi_{I} \right)^{2}}}\frac{t_{i}\left( t_{i}-1 \right)}{2} \right]$$

$$=\left( 1+\left( n_{i}-2 \right)\rho\right)\frac{\pi_{I}}{1-\pi_{I}}t_{i}-\frac{\pi_{I}}{1-\pi_{I}}\rho t_{i}\left( t_{i}-1 \right) = \frac{\pi_{I}}{1-\pi_{I}}\left( 1+\left( c_{i}-1 \right)\rho\right)t_{i} = \frac{\pi_{I}}{1-\pi_{I}}\left( 1-\rho\right)t_{i}+\frac{\pi_{I}}{1-\pi_{I}}\rho t_{i}c_{i}$$

Sum over all clusters $i=1,\ldots, M$. Cluster $i$ is of size $n_{i}$and contains $t_{i}$ members assigned to treatment and $c_{i}$ members assigned to control.

$\sum_{i=1}^{M} \mathbf{D}_{i}^{T}\mathbf{V}_{i}^{-1}\mathbf{D}_{i}= \sum_{i=1}^{M} \left[ \frac{1}{\left( 1-\rho\right)\left( 1+\left( n_{i}-1 \right)\rho\right)}\left[ \begin{matrix} a & b \\ b & c \end{matrix} \right] \right]= \left[ \begin{matrix} A & B \\ B & C \end{matrix} \right]$ , where

$$A=\sum_{i=1}^{M} \frac{1}{\left( 1-\rho\right)\left( 1+\left( n_{i}-1 \right)\rho\right)}\left[ \frac{\pi_{I}}{1-\pi_{I}}\left( 1-\rho\right)t_{i}+\frac{\pi_{C}}{1-\pi_{C}}\left( 1-\rho\right)c_{i}+\left[ \frac{\pi_{I}}{1-\pi_{I}}+\frac{\pi_{C}}{1-\pi_{C}}-\frac{2\pi_{I}\pi_{C}}{\sqrt{\pi_{I}\pi_{C}\left( 1-\pi_{I} \right)\left( 1-\pi_{C} \right)}} \right] \rho t_{i}c_{i} \right]$$

$$=\frac{\pi_{I}}{1-\pi_{I}}\sum_{i=1}^{M} \frac{t_{i}}{\left( 1+\left( n_{i}-1 \right)\rho\right)}+\frac{\pi_{C}}{1-\pi_{C}}\sum_{i=1}^{M} \frac{c_{i}}{\left( 1+\left( n_{i}-1 \right)\rho\right)}+\left[ \frac{\pi_{I}}{1-\pi_{I}}+\frac{\pi_{C}}{1-\pi_{C}}-\frac{2\pi_{I}\pi_{C}}{\sqrt{\pi_{I}\pi_{C}\left( 1-\pi_{I} \right)\left( 1-\pi_{C} \right)}} \right]\frac{\rho}{1-\rho}\sum_{i=1}^{M} \frac{{t_{i}c}_{i}}{\left( 1+\left( n_{i}-1 \right)\rho\right)}$$

$$B=\sum_{i=1}^{M} \frac{1}{\left( 1-\rho\right)\left( 1+\left( n_{i}-1 \right)\rho\right)}\left[ \frac{\pi_{I}}{1-\pi_{I}}\left( 1-\rho\right)t_{i}+\left[ \frac{\pi_{I}}{1-\pi_{I}}-\frac{\pi_{I}\pi_{C}}{\sqrt{\pi_{I}\pi_{C}\left( 1-\pi_{I} \right)\left( 1-\pi_{C} \right)}} \right]\rho t_{i}c_{i} \right]$$

$$=\frac{\pi_{I}}{1-\pi_{I}}\sum_{i=1}^{M} \frac{t_{i}}{\left( 1+\left( n_{i}-1 \right)\rho\right)}+\left[ \frac{\pi_{I}}{1-\pi_{I}}-\frac{\pi_{I}\pi_{C}}{\sqrt{\pi_{I}\pi_{C}\left( 1-\pi_{I} \right)\left( 1-\pi_{C} \right)}} \right]\frac{\rho}{1-\rho}\sum_{i=1}^{M} \frac{{t_{i}c}_{i}}{\left( 1+\left( n_{i}-1 \right)\rho\right)}$$

$$C=\sum_{i=1}^{M} \frac{1}{\left( 1-\rho\right)\left( 1+\left( n_{i}-1 \right)\rho\right)}\left[ \frac{\pi_{I}}{1-\pi_{I}}\left( 1-\rho\right)t_{i}+\frac{\pi_{I}}{1-\pi_{I}}\rho t_{i}c_{i} \right] = \frac{\pi_{I}}{1-\pi_{I}}\sum_{i=1}^{M} \frac{t_{i}}{\left( 1+\left( n_{i}-1 \right)\rho\right)}+\frac{\pi_{I}}{1-\pi_{I}}\left( \frac{\rho}{1-\rho} \right)\sum_{i=1}^{M} \frac{{t_{i}c}_{i}}{\left( 1+\left( n_{i}-1 \right)\rho\right)}$$

$$\mathrm{cov} \left( \hat{\boldsymbol{\beta}} \right)=\left[ \sum_{i=1}^{M} \mathbf{D}_{i}^{T}\mathbf{V}_{i}^{-1}\mathbf{D}_{i} \right]^{-1}=\left[ \begin{matrix} A & B \\ B & C \end{matrix} \right]^{-1}=\frac{1}{AC-B^{2}}\left[ \begin{matrix} C & -B \\ -B & A \end{matrix} \right]$$

$$AC-B^{2}=\left[ \frac{\pi_{I}}{1-\pi_{I}}\sum_{i=1}^{M} \frac{t_{i}}{\left( 1+\left( n_{i}-1 \right)\rho\right)}+\frac{\pi_{C}}{1-\pi_{C}}\sum_{i=1}^{M} \frac{c_{i}}{\left( 1+\left( n_{i}-1 \right)\rho\right)}+\left[ \frac{\pi_{I}}{1-\pi_{I}}+\frac{\pi_{C}}{1-\pi_{C}}-\frac{2\pi_{I}\pi_{C}}{\sqrt{\pi_{I}\pi_{C}\left( 1-\pi_{I} \right)\left( 1-\pi_{C} \right)}} \right]\frac{\rho}{1-\rho}\sum_{i=1}^{M} \frac{{t_{i}c}_{i}}{\left( 1+\left( n_{i}-1 \right)\rho\right)} \right]\times\left[ \frac{\pi_{I}}{1-\pi_{I}}\sum_{i=1}^{M} \frac{t_{i}}{\left( 1+\left( n_{i}-1 \right)\rho\right)}+\frac{\pi_{I}}{1-\pi_{I}}\left( \frac{\rho}{1-\rho} \right)\sum_{i=1}^{M} \frac{{t_{i}c}_{i}}{\left( 1+\left( n_{i}-1 \right)\rho\right)} \right]-\left[ \frac{\pi_{I}}{1-\pi_{I}}\sum_{i=1}^{M} \frac{t_{i}}{\left( 1+\left( n_{i}-1 \right)\rho\right)}+\left[ \frac{\pi_{I}}{1-\pi_{I}}-\frac{\pi_{I}\pi_{C}}{\sqrt{\pi_{I}\pi_{C}\left( 1-\pi_{I} \right)\left( 1-\pi_{C} \right)}} \right]\frac{\rho}{1-\rho}\sum_{i=1}^{M} \frac{{t_{i}c}_{i}}{\left( 1+\left( n_{i}-1 \right)\rho\right)} \right]^{2}$$

$$=\frac{\pi_{I}\pi_{C}}{\left( 1-\pi_{I} \right)\left( 1-\pi_{C} \right)}\sum_{i=1}^{M} \frac{t_{i}}{\left( 1+\left( n_{i}-1 \right)\rho\right)}\sum_{i=1}^{M} \frac{c_{i}}{\left( 1+\left( n_{i}-1 \right)\rho\right)}+\frac{\pi_{I}\pi_{C}}{\left( 1-\pi_{I} \right)\left( 1-\pi_{C} \right)}\left( \frac{\rho}{1-\rho} \right)\sum_{i=1}^{M} \frac{c_{i}}{\left( 1+\left( n_{i}-1 \right)\rho\right)}\sum_{i=1}^{M} \frac{{t_{i}c}_{i}}{\left( 1+\left( n_{i}-1 \right)\rho\right)}+\left[ \frac{\pi_{I}^{2}}{\left( 1-\pi_{I} \right)^{2}}+\frac{\pi_{I}\pi_{C}}{\left( 1-\pi_{I} \right)\left( 1-\pi_{C} \right)}-\frac{2\pi_{I}^{2}\pi_{C}}{\left( 1-\pi_{I} \right)\sqrt{\pi_{I}\pi_{C}\left( 1-\pi_{I} \right)\left( 1-\pi_{C} \right)}}+\frac{\pi_{I}^{2}}{\left( 1-\pi_{I} \right)^{2}}-\frac{2\pi_{I}^{2}}{\left( 1-\pi_{I} \right)^{2}}+\frac{2\pi_{I}^{2}\pi_{C}}{\left( 1-\pi_{I} \right)\sqrt{\pi_{I}\pi_{C}\left( 1-\pi_{I} \right)\left( 1-\pi_{C} \right)}} \right]\left( \frac{\rho}{1-\rho} \right)\sum_{i=1}^{M} \frac{t_{i}}{\left( 1+\left( n_{i}-1 \right)\rho\right)}\sum_{i=1}^{M} \frac{{t_{i}c}_{i}}{\left( 1+\left( n_{i}-1 \right)\rho\right)}+\left[ \frac{\pi_{I}^{2}}{\left( 1-\pi_{I} \right)^{2}}+\frac{\pi_{I}\pi_{C}}{\left( 1-\pi_{I} \right)\left( 1-\pi_{C} \right)}-\frac{2\pi_{I}^{2}\pi_{C}}{\left( 1-\pi_{I} \right)\sqrt{\pi_{I}\pi_{C}\left( 1-\pi_{I} \right)\left( 1-\pi_{C} \right)}}-\frac{\pi_{I}^{2}}{\left( 1-\pi_{I} \right)^{2}}+\frac{2\pi_{I}^{2}\pi_{C}}{\left( 1-\pi_{I} \right)\sqrt{\pi_{I}\pi_{C}\left( 1-\pi_{I} \right)\left( 1-\pi_{C} \right)}}-\frac{\pi_{I}\pi_{C}}{\left( 1-\pi_{I} \right)\left( 1-\pi_{C} \right)} \right]\frac{\rho^{2}}{\left( 1-\rho\right)^{2}}\left[ \sum_{i=1}^{M} \frac{{t_{i}c}_{i}}{\left( 1+\left( n_{i}-1 \right)\rho\right)} \right]^{2}$$

$$=\frac{\pi_{I}\pi_{C}}{\left( 1-\pi_{I} \right)\left( 1-\pi_{C} \right)}\left[ \sum_{i=1}^{M} \frac{t_{i}}{\left( 1+\left( n_{i}-1 \right)\rho\right)}\sum_{i=1}^{M} \frac{c_{i}}{\left( 1+\left( n_{i}-1 \right)\rho\right)}+\frac{\rho}{1-\rho}\sum_{i=1}^{M} \frac{t_{i}}{\left( 1+\left( n_{i}-1 \right)\rho\right)}\sum_{i=1}^{M} \frac{{t_{i}c}_{i}}{\left( 1+\left( n_{i}-1 \right)\rho\right)}+\frac{\rho}{1-\rho}\sum_{i=1}^{M} \frac{c_{i}}{\left( 1+\left( n_{i}-1 \right)\rho\right)}\sum_{i=1}^{M} \frac{{t_{i}c}_{i}}{\left( 1+\left( n_{i}-1 \right)\rho\right)} \right]$$

$$\Rightarrow\mathrm{cov} \left( \hat{\boldsymbol{\beta}} \right)=$$

$$\frac{\left( 1-\pi_{I} \right)\left( 1-\pi_{C} \right)}{\pi_{I}\pi_{C}\left[ \sum_{i=1}^{M} \frac{t_{i}}{\left( 1+\left( n_{i}-1 \right)\rho\right)}\sum_{i=1}^{M} \frac{c_{i}}{\left( 1+\left( n_{i}-1 \right)\rho\right)}+\frac{\rho}{1-\rho}\sum_{i=1}^{M} \frac{t_{i}}{\left( 1+\left( n_{i}-1 \right)\rho\right)}\sum_{i=1}^{M} \frac{{t_{i}c}_{i}}{\left( 1+\left( n_{i}-1 \right)\rho\right)}+\frac{\rho}{1-\rho}\sum_{i=1}^{M} \frac{c_{i}}{\left( 1+\left( n_{i}-1 \right)\rho\right)}\sum_{i=1}^{M} \frac{{t_{i}c}_{i}}{\left( 1+\left( n_{i}-1 \right)\rho\right)} \right]}\times\left[ \begin{matrix} C & -B \\ -B & A \end{matrix} \right]$$

$$\mathrm{var}\left( \hat{\beta}_{1} \right)=$$

$$\frac{\left( 1-\pi_{I} \right)\left( 1-\pi_{C} \right)\left[ \frac{\pi_{I}}{1-\pi_{I}}\sum_{i=1}^{M} \frac{t_{i}}{\left( 1+\left( n_{i}-1 \right)\rho\right)}+\frac{\pi_{C}}{1-\pi_{C}}\sum_{i=1}^{M} \frac{c_{i}}{\left( 1+\left( n_{i}-1 \right)\rho\right)}+\left[ \frac{\pi_{I}}{1-\pi_{I}}+\frac{\pi_{C}}{1-\pi_{C}}-\frac{2\pi_{I}\pi_{C}}{\sqrt{\pi_{I}\pi_{C}\left( 1-\pi_{I} \right)\left( 1-\pi_{C} \right)}} \right]\frac{\rho}{1-\rho}\sum_{i=1}^{M} \frac{{t_{i}c}_{i}}{\left( 1+\left( n_{i}-1 \right)\rho\right)} \right]}{\pi_{I}\pi_{C}\left[ \sum_{i=1}^{M} \frac{t_{i}}{\left( 1+\left( n_{i}-1 \right)\rho\right)}\sum_{i=1}^{M} \frac{c_{i}}{\left( 1+\left( n_{i}-1 \right)\rho\right)}+\frac{\rho}{1-\rho}\sum_{i=1}^{M} \frac{t_{i}}{\left( 1+\left( n_{i}-1 \right)\rho\right)}\sum_{i=1}^{M} \frac{{t_{i}c}_{i}}{\left( 1+\left( n_{i}-1 \right)\rho\right)}+\frac{\rho}{1-\rho}\sum_{i=1}^{M} \frac{c_{i}}{\left( 1+\left( n_{i}-1 \right)\rho\right)}\sum_{i=1}^{M} \frac{{t_{i}c}_{i}}{\left( 1+\left( n_{i}-1 \right)\rho\right)} \right]}$$

$$=\frac{\pi_{I}\left( 1-\pi_{C} \right)\sum_{i=1}^{M} \frac{t_{i}}{\left( 1+\left( n_{i}-1 \right)\rho\right)}+\pi_{C}\left( 1-\pi_{I} \right)\sum_{i=1}^{M} \frac{c_{i}}{\left( 1+\left( n_{i}-1 \right)\rho\right)}+\left[ \pi_{I}\left( 1-\pi_{C} \right)+\pi_{C}\left( 1-\pi_{I} \right)-2\sqrt{\pi_{I}\pi_{C}\left( 1-\pi_{I} \right)\left( 1-\pi_{C} \right)} \right]\frac{\rho}{1-\rho}\sum_{i=1}^{M} \frac{{t_{i}c}_{i}}{\left( 1+\left( n_{i}-1 \right)\rho\right)}}{\pi_{I}\pi_{C}\left[ \sum_{i=1}^{M} \frac{t_{i}}{\left( 1+\left( n_{i}-1 \right)\rho\right)}\sum_{i=1}^{M} \frac{c_{i}}{\left( 1+\left( n_{i}-1 \right)\rho\right)}+\frac{\rho}{1-\rho}\sum_{i=1}^{M} \frac{t_{i}}{\left( 1+\left( n_{i}-1 \right)\rho\right)}\sum_{i=1}^{M} \frac{{t_{i}c}_{i}}{\left( 1+\left( n_{i}-1 \right)\rho\right)}+\frac{\rho}{1-\rho}\sum_{i=1}^{M} \frac{c_{i}}{\left( 1+\left( n_{i}-1 \right)\rho\right)}\sum_{i=1}^{M} \frac{{t_{i}c}_{i}}{\left( 1+\left( n_{i}-1 \right)\rho\right)} \right]}$$

$$\Rightarrow DEFF=$$

$$\frac{\pi_{I}\left( 1-\pi_{C} \right)\sum_{i=1}^{M} \frac{t_{i}}{\left( 1+\left( n_{i}-1 \right)\rho\right)}+\pi_{C}\left( 1-\pi_{I} \right)\sum_{i=1}^{M} \frac{c_{i}}{\left( 1+\left( n_{i}-1 \right)\rho\right)}+\left[ \pi_{I}\left( 1-\pi_{C} \right)+\pi_{C}\left( 1-\pi_{I} \right)-2\sqrt{\pi_{I}\pi_{C}\left( 1-\pi_{I} \right)\left( 1-\pi_{C} \right)} \right]\frac{\rho}{1-\rho}\sum_{i=1}^{M} \frac{{t_{i}c}_{i}}{\left( 1+\left( n_{i}-1 \right)\rho\right)}}{\pi_{I}\pi_{C}\left[ \sum_{i=1}^{M} \frac{t_{i}}{\left( 1+\left( n_{i}-1 \right)\rho\right)}\sum_{i=1}^{M} \frac{c_{i}}{\left( 1+\left( n_{i}-1 \right)\rho\right)}+\frac{\rho}{1-\rho}\sum_{i=1}^{M} \frac{t_{i}}{\left( 1+\left( n_{i}-1 \right)\rho\right)}\sum_{i=1}^{M} \frac{{t_{i}c}_{i}}{\left( 1+\left( n_{i}-1 \right)\rho\right)}+\frac{\rho}{1-\rho}\sum_{i=1}^{M} \frac{c_{i}}{\left( 1+\left( n_{i}-1 \right)\rho\right)}\sum_{i=1}^{M} \frac{{t_{i}c}_{i}}{\left( 1+\left( n_{i}-1 \right)\rho\right)} \right]} \times\frac{N\pi_{I}\pi_{C}}{2\left( \pi_{I}\left( 1-\pi_{C} \right)+\pi_{C}\left( 1-\pi_{I} \right) \right)}$$

$$=\frac{\pi_{I}\left( 1-\pi_{C} \right)\sum_{i=1}^{M} \frac{t_{i}}{\left( 1+\left( n_{i}-1 \right)\rho\right)}+\pi_{C}\left( 1-\pi_{I} \right)\sum_{i=1}^{M} \frac{c_{i}}{\left( 1+\left( n_{i}-1 \right)\rho\right)}+\left[ \pi_{I}\left( 1-\pi_{C} \right)+\pi_{C}\left( 1-\pi_{I} \right)-2\sqrt{\pi_{I}\pi_{C}\left( 1-\pi_{I} \right)\left( 1-\pi_{C} \right)} \right]\frac{\rho}{1-\rho}\sum_{i=1}^{M} \frac{{t_{i}c}_{i}}{\left( 1+\left( n_{i}-1 \right)\rho\right)}}{\sum_{i=1}^{M} \frac{t_{i}}{\left( 1+\left( n_{i}-1 \right)\rho\right)}\sum_{i=1}^{M} \frac{c_{i}}{\left( 1+\left( n_{i}-1 \right)\rho\right)}+\frac{\rho}{1-\rho}\sum_{i=1}^{M} \frac{t_{i}}{\left( 1+\left( n_{i}-1 \right)\rho\right)}\sum_{i=1}^{M} \frac{{t_{i}c}_{i}}{\left( 1+\left( n_{i}-1 \right)\rho\right)}+\frac{\rho}{1-\rho}\sum_{i=1}^{M} \frac{c_{i}}{\left( 1+\left( n_{i}-1 \right)\rho\right)}\sum_{i=1}^{M} \frac{{t_{i}c}_{i}}{\left( 1+\left( n_{i}-1 \right)\rho\right)}} \times\frac{N}{2\left( \pi_{I}\left( 1-\pi_{C} \right)+\pi_{C}\left( 1-\pi_{I} \right) \right)}$$

- - 1. Cluster randomisation

As shown in section 3.2.1:

$$\sum_{i=1}^{M} \frac{t_{i}}{\left( 1+\left( n_{i}-1 \right)\rho\right)}=\frac{N}{2}\sum_{k=1}^{K} {\frac{1}{\left( 1+\left( k-1 \right)\rho\right)}\gamma}_{k}$$

$$\sum_{i=1}^{M} \frac{c_{i}}{\left( 1+\left( n_{i}-1 \right)\rho\right)}=\frac{N}{2}\sum_{k=1}^{K} {\frac{1}{\left( 1+\left( k-1 \right)\rho\right)}\gamma}_{k}$$

$$\sum_{i=1}^{M} \frac{{t_{i}c}_{i}}{\left( 1+\left( n_{i}-1 \right)\rho\right)}=0$$

$$DEFF= \frac{\pi_{I}\left( 1-\pi_{C} \right)\frac{N}{2}\left( \sum_{k=1}^{K} {\frac{1}{\left( 1+\left( k-1 \right)\rho\right)}\gamma}_{k} \right)+\pi_{C}\left( 1-\pi_{I} \right)\frac{N}{2}\left( \sum_{k=1}^{K} {\frac{1}{\left( 1+\left( k-1 \right)\rho\right)}\gamma}_{k} \right)}{\left( \frac{N}{2}\sum_{k=1}^{K} {\frac{1}{\left( 1+\left( k-1 \right)\rho\right)}\gamma}_{k} \right)^{2}} \times\frac{N}{2\left( \pi_{I}\left( 1-\pi_{C} \right)+\pi_{C}\left( 1-\pi_{I} \right) \right)}$$

$$=\frac{\left( \pi_{I}\left( 1-\pi_{C} \right)+\pi_{C}\left( 1-\pi_{I} \right) \right)\left( \sum_{k=1}^{K} {\frac{1}{\left( 1+\left( k-1 \right)\rho\right)}\gamma}_{k} \right)}{\left( \sum_{k=1}^{K} {\frac{1}{\left( 1+\left( k-1 \right)\rho\right)}\gamma}_{k} \right)^{2}} \times\frac{1}{\left( \pi_{I}\left( 1-\pi_{C} \right)+\pi_{C}\left( 1-\pi_{I} \right) \right)} = \frac{1}{\sum_{k=1}^{K} {\frac{1}{\left( 1+\left( k-1 \right)\rho\right)}\gamma}_{k}} = \left[ \sum_{k=1}^{K} {\frac{1}{\left( 1+\left( k-1 \right)\rho\right)}\gamma}_{k} \right]^{-1}$$

- - 1. Individual randomisation

As shown in section 3.2.2:

$$\sum_{i=1}^{M} \frac{t_{i}}{\left( 1+\left( n_{i}-1 \right)\rho\right)}=\frac{N}{2}\sum_{k=1}^{K} \frac{1}{\left( 1+\left( k-1 \right)\rho\right)}\gamma_{k}$$

$$\sum_{i=1}^{M} \frac{c_{i}}{\left( 1+\left( n_{i}-1 \right)\rho\right)}=\frac{N}{2}\sum_{k=1}^{K} \frac{1}{\left( 1+\left( k-1 \right)\rho\right)}\gamma_{k}$$

$$\sum_{i=1}^{M} \frac{t_{i}c_{i}}{\left( 1+\left( n_{i}-1 \right)\rho\right)}=\frac{N}{4}\sum_{k=1}^{K} \frac{k-1}{\left( 1+\left( k-1 \right)\rho\right)}\gamma_{k}$$

$$DEFF=$$

$$\frac{\pi_{I}\left( 1-\pi_{C} \right)\left( \frac{N}{2} \right)\sum_{k=1}^{K} \frac{1}{\left( 1+\left( k-1 \right)\rho\right)}\gamma_{k}+\pi_{C}\left( 1-\pi_{I} \right)\left( \frac{N}{2} \right)\sum_{k=1}^{K} \frac{1}{\left( 1+\left( k-1 \right)\rho\right)}\gamma_{k}+\left[ \pi_{I}\left( 1-\pi_{C} \right)+\pi_{C}\left( 1-\pi_{I} \right)-2\sqrt{\pi_{I}\pi_{C}\left( 1-\pi_{I} \right)\left( 1-\pi_{C} \right)} \right]\frac{\rho}{1-\rho}\left( \frac{N}{4} \right)\sum_{k=1}^{K} \frac{k-1}{\left( 1+\left( k-1 \right)\rho\right)}\gamma_{k}}{\left( \frac{N}{2}\sum_{k=1}^{K} \frac{1}{\left( 1+\left( k-1 \right)\rho\right)}\gamma_{k} \right)^{2}+2\left( \left( \frac{\rho}{1-\rho} \right)\left( \frac{N}{2} \right)\sum_{k=1}^{K} \frac{1}{\left( 1+\left( k-1 \right)\rho\right)}\gamma_{k}\left( \frac{N}{4} \right)\sum_{k=1}^{K} \frac{k-1}{\left( 1+\left( k-1 \right)\rho\right)}\gamma_{k} \right)} \times\frac{N}{2\left( \pi_{I}\left( 1-\pi_{C} \right)+\pi_{C}\left( 1-\pi_{I} \right) \right)}$$

$$=\frac{\left( \pi_{I}\left( 1-\pi_{C} \right)+\pi_{C}\left( 1-\pi_{I} \right) \right)\sum_{k=1}^{K} \frac{1}{\left( 1+\left( k-1 \right)\rho\right)}\gamma_{k}+\frac{1}{2}\left[ \pi_{I}\left( 1-\pi_{C} \right)+\pi_{C}\left( 1-\pi_{I} \right)-2\sqrt{\pi_{I}\pi_{C}\left( 1-\pi_{I} \right)\left( 1-\pi_{C} \right)} \right]\left( \frac{\rho}{1-\rho} \right)\left( \sum_{k=1}^{K} \frac{k-1}{\left( 1+\left( k-1 \right)\rho\right)}\gamma_{k} \right)}{\left( \pi_{I}\left( 1-\pi_{C} \right)+\pi_{C}\left( 1-\pi_{I} \right) \right)\left( \sum_{k=1}^{K} \frac{1}{\left( 1+\left( k-1 \right)\rho\right)}\gamma_{k} \right)^{2}+\left( \frac{\rho}{1-\rho} \right)\left( \sum_{k=1}^{K} \frac{1}{\left( 1+\left( k-1 \right)\rho\right)}\gamma_{k} \right)\left( \sum_{k=1}^{K} \frac{k-1}{\left( 1+\left( k-1 \right)\rho\right)}\gamma_{k} \right)}$$

$$=\frac{\sum_{k=1}^{K} \frac{1}{\left( 1+\left( k-1 \right)\rho\right)}\gamma_{k}+\left( \frac{1}{2}-\frac{\sqrt{\pi_{I}\pi_{C}\left( 1-\pi_{I} \right)\left( 1-\pi_{C} \right)}}{\pi_{I}\left( 1-\pi_{C} \right)+\pi_{C}\left( 1-\pi_{I} \right)} \right)\left( \frac{\rho}{1-\rho} \right)\left( \sum_{k=1}^{K} \frac{k-1}{\left( 1+\left( k-1 \right)\rho\right)}\gamma_{k} \right)}{\left( \sum_{k=1}^{K} \frac{1}{\left( 1+\left( k-1 \right)\rho\right)}\gamma_{k} \right)^{2}+\left( \frac{\rho}{1-\rho} \right)\left( \sum_{k=1}^{K} \frac{1}{\left( 1+\left( k-1 \right)\rho\right)}\gamma_{k} \right)\left( \sum_{k=1}^{K} \frac{k-1}{\left( 1+\left( k-1 \right)\rho\right)}\gamma_{k} \right)}$$

# PROOFS OF COMBINATORIAL PROPERTIES

The binomial theorem states that $\left( x+a \right)^{k}=\sum_{d=0}^{k} \binom{k}{d}x^{d}a^{k-d}$ (Boros & Moll 2004).

Therefore, when $a=1$:

$\left( 1+x \right)^{k}=\sum_{d=0}^{k} \binom{k}{d}x^{d}$ ( P1 )

and when $x=a=1$:

$2^{k}=\sum_{d=0}^{k} \binom{k}{d}$ ( P2 )

Differentiating both sides of equation (P1) with respect to $x$, gives:

$k\left( 1+x \right)^{k-1}=\sum_{d=0}^{k} d\binom{k}{d}x^{d-1}$ ( P3 )

and when $x=1$:

$k2^{k-1}=\sum_{d=0}^{k} d\binom{k}{d}$ ( P4 )

Differentiating both sides of equation (P3) with respect to $x$, gives:

$k(k-1)\left( 1+x \right)^{k-2}=\sum_{d=0}^{k} d(d-1)\binom{k}{d}x^{d-2}$

and when $x=1$:

$k(k-1)2^{k-2}=\sum_{d=0}^{k} d(d-1)\binom{k}{d}$ ( P5 )

Summing equations (P4) and (P5):

$$k2^{k-1}+k\left( k-1 \right)2^{k-2}=\sum_{d=0}^{k} \left[ d\binom{k}{d}+d\left( d-1 \right)\binom{k}{d} \right]$$

$$\Rightarrow k2^{k-2}\left( 2+k-1 \right)=\sum_{d=0}^{k} d\left( 1+d-1 \right)\binom{k}{d}$$

 $\Rightarrow k(k+1)2^{k-2}=\sum_{d=0}^{k} d^{2}\binom{k}{d}$ ( P6 )

Using equations (P2) and (P4), we can show that:

$$\sum_{d=0}^{k} \left( k-d \right)\binom{k}{d}=k\sum_{d=0}^{k} \binom{k}{d}-\sum_{d=0}^{k} d\binom{k}{d}$$

$$=k2^{k}-k2^{k-1}$$

$$=k2^{k-1}\left( 2-1 \right)$$

 $=k2^{k-1}$ ( P7 )

Using equations (P4) and (P6), we can show that:

$$\sum_{d=0}^{k} d\left( k-d \right)\binom{k}{d}=k\sum_{d=0}^{k} d\binom{k}{d}-\sum_{d=0}^{k} d^{2}\binom{k}{d}$$

$$=k^{2}2^{k-1}-k\left( k+1 \right)2^{k-2}$$

$$=k2^{k-2}\left( 2k-k-1 \right)$$

 $=k\left( k-1 \right)2^{k-2}$ ( P8 )

Using equations (P2), (P4) and (P6), we can show that:

$$\sum_{d=0}^{k} \left( k-d \right)\left( k-d-1 \right)\binom{k}{d}=\left( k^{2}-k \right)\sum_{d=0}^{k} \binom{k}{d}-\left( 2k-1 \right)\sum_{d=0}^{k} d\binom{k}{d}+\sum_{d=0}^{k} d^{2}\binom{k}{d}$$

$$=\left( k^{2}-k \right)2^{k}-\left( 2k-1 \right)k2^{k-1}+k\left( k+1 \right)2^{k-2}$$

$$=k2^{k-2}\left( 4k-4-4k+2+k+1 \right)$$

 $=k\left( k-1 \right)2^{k-2}$ ( P9 )

Using equations (P4) and (P6), we can show that:

$$\sum_{d=0}^{k} d\left( 1+\left( k-d-1 \right)\rho\right)\binom{k}{d}=\left( 1+\rho k-\rho\right)\sum_{d=0}^{k} d\binom{k}{d}-\rho\sum_{d=0}^{k} d^{2}\binom{k}{d}$$

$$=\left( 1+\rho k-\rho\right)k2^{k-1}-\rho k\left( k+1 \right)2^{k-2}$$

$$=2^{k-2}k\left( 2+2\rho k-2\rho-\rho k-\rho\right)$$

$$=2^{k-2}k\left( 2+\rho k-3\rho\right)$$

 $=2^{k-2}k\left( \rho\left( k-3 \right)+2 \right)$ ( P10 )

# REFERENCES

Boros, G., Moll, V. *Irresistible integrals: symbolics, analysis, and experiments in the evaluation of integrals*. Cambridge, UK: Cambridge University Press; 2004.

McDaniel, L.S., Henderson, N.C., Rathouz, P.J. Fast pure R implementation of GEE: application of the Matrix package. *The R Journal*, 2013; 5(1): 181–187.
