# Supplementary material for: Sample Size Calculations for Partially Clustered Trials
Source: Stat Med. 2025 Jul 15;44(15-17):e70172. doi: 10.1002/sim.70172 (PMC12261973; doi:10.1002/sim.70172)
Supplement: Supplementary file 2 — File S2. Extra results. [file SIM-44-0-s004.docx]

**Supplementary File 2 – Extra results**

**Title:** Sample size calculations for partially clustered trials

**Authors:** Kylie M. Lange, Jessica Kasza, Thomas R. Sullivan, Lisa N. Yelland

**Supplementary tables and figures**

Supplementary Table 1: Design effects for a binary outcome with a log link by method of randomisation and GEE working correlation structure

Supplementary Table 2: Observed and expected design effects for a binary outcome with a log link

Supplementary Table 3: Observed and expected power for a binary outcome with a log link

Supplementary Figure 1: Design effects for a binary outcome with a logit link by randomisation method and GEE working correlation structure

Supplementary Figure 2: Design effects for a binary outcome with a logit link for varying odds ratios when $\boldsymbol{\pi}_{\boldsymbol{C}}$ = 0.1

Supplementary Figure 3: Design effects for a binary outcome with a log link by randomisation method and GEE working correlation structure

Supplementary Figure 4: Design effects for a binary outcome with log link for varying relative risks when $\pi_{C}$ = 0.4

Supplementary Figure 5: Design effects for a binary outcome with log link for varying relative risks when $\pi_{C}$ = 0.1

**Supplementary Table 1: Design effects for a binary outcome with a log link by method of randomisation and GEE working correlation structure**

| Randomisation method | Independence working correlation | Exchangeable working correlation |
| --- | --- | --- |
| Binary outcome – log link | | |
| Cluster | $1+\rho\sum_{k=1}^{K} {\left( k-1 \right)\gamma}_{k}$ | $\left[ \sum_{k=1}^{K} {\frac{1}{\left( 1+\left( k-1 \right)\rho\right)}\gamma}_{k} \right]^{-1}$ |
| Individual | $1+\rho\left[ \left( \frac{1}{2}-\frac{\sqrt{\pi_{I}\pi_{C}\left( 1-\pi_{I} \right)\left( 1-\pi_{C} \right)}}{\pi_{I}\left( 1-\pi_{C} \right)+\pi_{C}\left( 1-\pi_{I} \right)} \right)\sum_{k=1}^{K} \left( k-1 \right)\gamma_{k} \right]$ | $\frac{\sum_{k=1}^{K} \frac{1}{\left( 1+\left( k-1 \right)\rho\right)}\gamma_{k}+\left( \frac{1}{2}-\frac{\sqrt{\pi_{I}\pi_{C}\left( 1-\pi_{I} \right)\left( 1-\pi_{C} \right)}}{\pi_{I}\left( 1-\pi_{C} \right)+\pi_{C}\left( 1-\pi_{I} \right)} \right)\left( \frac{\rho}{1-\rho} \right)\left( \sum_{k=1}^{K} \frac{k-1}{\left( 1+\left( k-1 \right)\rho\right)}\gamma_{k} \right)}{\left( \sum_{k=1}^{K} \frac{1}{\left( 1+\left( k-1 \right)\rho\right)}\gamma_{k} \right)^{2}+\left( \frac{\rho}{1-\rho} \right)\left( \sum_{k=1}^{K} \frac{1}{\left( 1+\left( k-1 \right)\rho\right)}\gamma_{k} \right)\left( \sum_{k=1}^{K} \frac{k-1}{\left( 1+\left( k-1 \right)\rho\right)}\gamma_{k} \right)}$ |

Supplementary Table 2: Observed and expected design effects for a binary outcome with a log link

|  | | | | | GEE independence | | | GEE exchangeable | | |
| --- | --- | --- | --- | --- | --- | --- | --- | --- | --- | --- |
| Randomisation method | Sample size | Distribution of cluster sizes | ICC | Observed DEFF* | | Expected DEFF | Relative difference (%)* | Observed DEFF* | Expected DEFF | Relative difference (%)* |
| Cluster | 360 | Unequal cluster proportions | 0.2 | 1.19 | | 1.20 | -1.04 | 1.15 | 1.16 | -0.89 |
| Cluster | 360 | Unequal cluster proportions | 0.8 | 1.78 | | 1.80 | -0.84 | 1.43 | 1.44 | -0.68 |
| Cluster | 360 | Equal cluster proportions | 0.2 | 1.38 | | 1.40 | -1.31 | 1.35 | 1.37 | -1.05 |
| Cluster | 360 | Equal cluster proportions | 0.8 | 2.58 | | 2.60 | -0.66 | 2.24 | 2.25 | -0.66 |
| Cluster | 840 | Unequal cluster proportions | 0.2 | 1.19 | | 1.20 | -0.57 | 1.16 | 1.16 | -0.47 |
| Cluster | 840 | Unequal cluster proportions | 0.8 | 1.79 | | 1.80 | -0.32 | 1.44 | 1.44 | -0.22 |
| Cluster | 840 | Equal cluster proportions | 0.2 | 1.39 | | 1.40 | -0.53 | 1.36 | 1.37 | -0.45 |
| Cluster | 840 | Equal cluster proportions | 0.8 | 2.59 | | 2.60 | -0.25 | 2.25 | 2.25 | -0.17 |
| Individual | 360 | Unequal cluster proportions | 0.2 | 0.99 | | 1.00 | -0.90 | 0.96 | 0.97 | -1.02 |
| Individual | 360 | Unequal cluster proportions | 0.8 | 0.99 | | 1.01 | -2.03 | 0.45 | 0.46 | -2.00 |
| Individual | 360 | Equal cluster proportions | 0.2 | 0.99 | | 1.00 | -1.11 | 0.93 | 0.94 | -1.20 |
| Individual | 360 | Equal cluster proportions | 0.8 | 1.00 | | 1.02 | -2.33 | 0.32 | 0.33 | -2.85 |
| Individual | 840 | Unequal cluster proportions | 0.2 | 1.00 | | 1.00 | -0.40 | 0.96 | 0.97 | -0.39 |
| Individual | 840 | Unequal cluster proportions | 0.8 | 1.00 | | 1.01 | -0.89 | 0.46 | 0.46 | -1.39 |
| Individual | 840 | Equal cluster proportions | 0.2 | 1.00 | | 1.00 | -0.38 | 0.94 | 0.94 | -0.41 |
| Individual | 840 | Equal cluster proportions | 0.8 | 1.01 | | 1.02 | -1.15 | 0.33 | 0.33 | -1.40 |

* Median value across 10000 simulated datasets.

Clusters of size 1-4 were distributed in proportions (0.70, 0.15, 0.10, 0.05) in scenarios with unequal cluster proportions (corresponding to $\boldsymbol{\gamma}$ = (0.47, 0.20, 0.20, 0.13)), and (0.25, 0.25, 0.25, 0.25) in scenarios with equal cluster proportions (corresponding to $\boldsymbol{\gamma}$ = (0.1, 0.2, 0.3, 0.4)).

GEE = generalised estimating equation, DEFF = design effect, ICC = intracluster correlation coefficient.

Supplementary Table 3: Observed and expected power for a binary outcome with a log link

|  | | | | GEE independence | | | GEE exchangeable | | |
| --- | --- | --- | --- | --- | --- | --- | --- | --- | --- |
| Randomisation method | Sample size | Proportion of clusters of size 1-4 | ICC | Observed power | Expected power | Difference | Observed power | Expected power | Absolute difference |
| Cluster | 360 | Unequal cluster proportions | 0.2 | 44.39 | 44.67 | -0.28 | 45.20 | 45.80 | -0.60 |
| Cluster | 360 | Unequal cluster proportions | 0.8 | 31.60 | 31.97 | -0.37 | 37.90 | 38.45 | -0.55 |
| Cluster | 360 | Equal cluster proportions | 0.2 | 39.32 | 39.38 | -0.06 | 40.28 | 40.14 | 0.14 |
| Cluster | 360 | Equal cluster proportions | 0.8 | 22.11 | 23.66 | -1.55 | 24.96 | 26.58 | -1.62 |
| Cluster | 840 | Unequal cluster proportions | 0.2 | 78.69 | 79.64 | -0.95 | 80.14 | 80.85 | -0.71 |
| Cluster | 840 | Unequal cluster proportions | 0.8 | 61.99 | 62.44 | -0.45 | 71.42 | 72.07 | -0.65 |
| Cluster | 840 | Equal cluster proportions | 0.2 | 72.58 | 73.30 | -0.72 | 73.59 | 74.28 | -0.69 |
| Cluster | 840 | Equal cluster proportions | 0.8 | 46.80 | 47.40 | -0.60 | 52.41 | 53.03 | -0.62 |
| Individual | 360 | Unequal cluster proportions | 0.2 | 50.73 | 51.51 | -0.78 | 52.30 | 52.89 | -0.59 |
| Individual | 360 | Unequal cluster proportions | 0.8 | 51.72 | 51.22 | 0.50 | 90.28 | 83.75 | 6.53 |
| Individual | 360 | Equal cluster proportions | 0.2 | 50.98 | 51.41 | -0.43 | 53.84 | 54.01 | -0.17 |
| Individual | 360 | Equal cluster proportions | 0.8 | 50.50 | 50.85 | -0.35 | 99.49 | 93.37 | 6.12 |
| Individual | 840 | Unequal cluster proportions | 0.2 | 85.83 | 86.25 | -0.42 | 86.91 | 87.38 | -0.47 |
| Individual | 840 | Unequal cluster proportions | 0.8 | 85.80 | 86.01 | -0.21 | 99.98 | 99.44 | 0.54 |
| Individual | 840 | Equal cluster proportions | 0.2 | 84.90 | 86.17 | -1.27 | 86.98 | 88.25 | -1.27 |
| Individual | 840 | Equal cluster proportions | 0.8 | 86.15 | 85.69 | 0.46 | 100.00 | 99.96 | 0.04 |

Clusters of size 1-4 were distributed in proportions (0.70, 0.15, 0.10, 0.05) in scenarios with unequal cluster proportions (corresponding to $\boldsymbol{\gamma}$ = (0.47, 0.20, 0.20, 0.13)), and (0.25, 0.25, 0.25, 0.25) in scenarios with equal cluster proportions (corresponding to $\boldsymbol{\gamma}$ = (0.1, 0.2, 0.3, 0.4)).

GEE = generalised estimating equation, ICC = intracluster correlation coefficient.

Supplementary Figure 1: Design effects for a binary outcome with a logit link by randomisation method and GEE working correlation structure


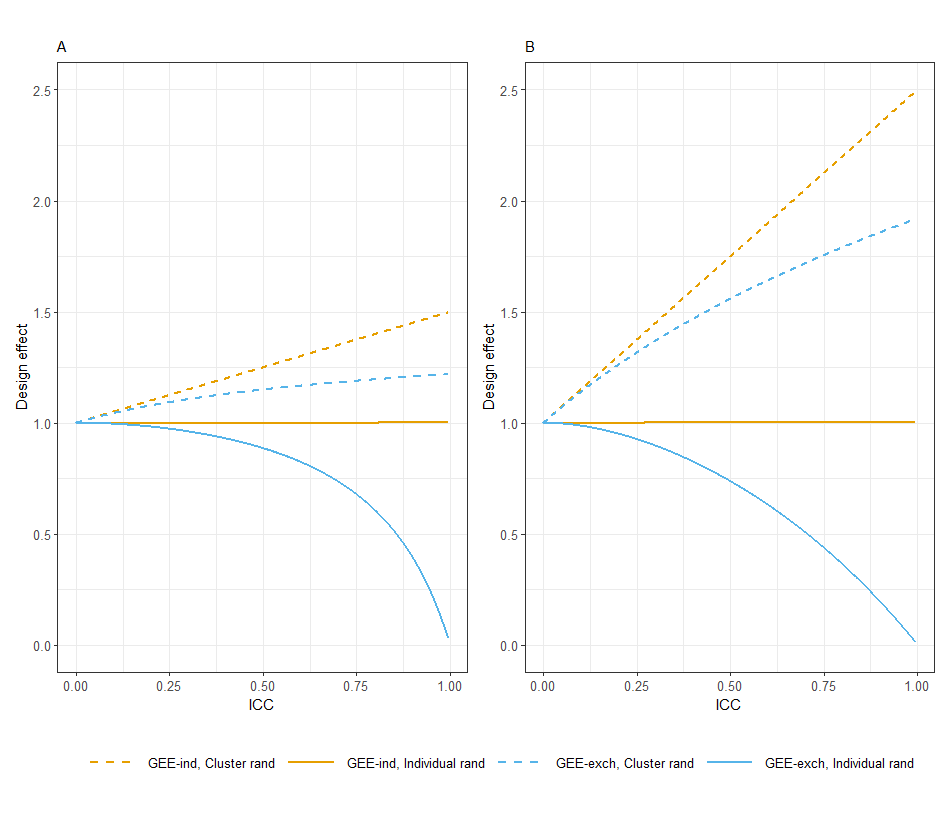


Figure shows the design effects for varying ICC with a maximum cluster size of 4, outcome prevalence of $\pi_{C}$= 0.4 and $\pi_{I}$= 0.3 (odds ratio = 0.64), and proportions of clusters of sizes 1-4 of (A) (0.70, 0.15, 0.10. 0.05) (corresponding to $\boldsymbol{\gamma}$ = (0.47, 0.20, 0.20, 0.13)) and (B) (0.25, 0.25, 0.25, 0.25) (corresponding to $\boldsymbol{\gamma}$ = (0.1, 0.2, 0.3, 0.4)). Design effects are shown for generalised estimating equations (GEEs) with an independence (GEE-ind) or exchangeable (GEE-exch) working correlation structure under cluster or individual randomisation of the clustered observations.

Supplementary Figure 2: Design effects for a binary outcome with a logit link for varying odds ratios when $\boldsymbol{\pi}_{\boldsymbol{c}}$= 0.1


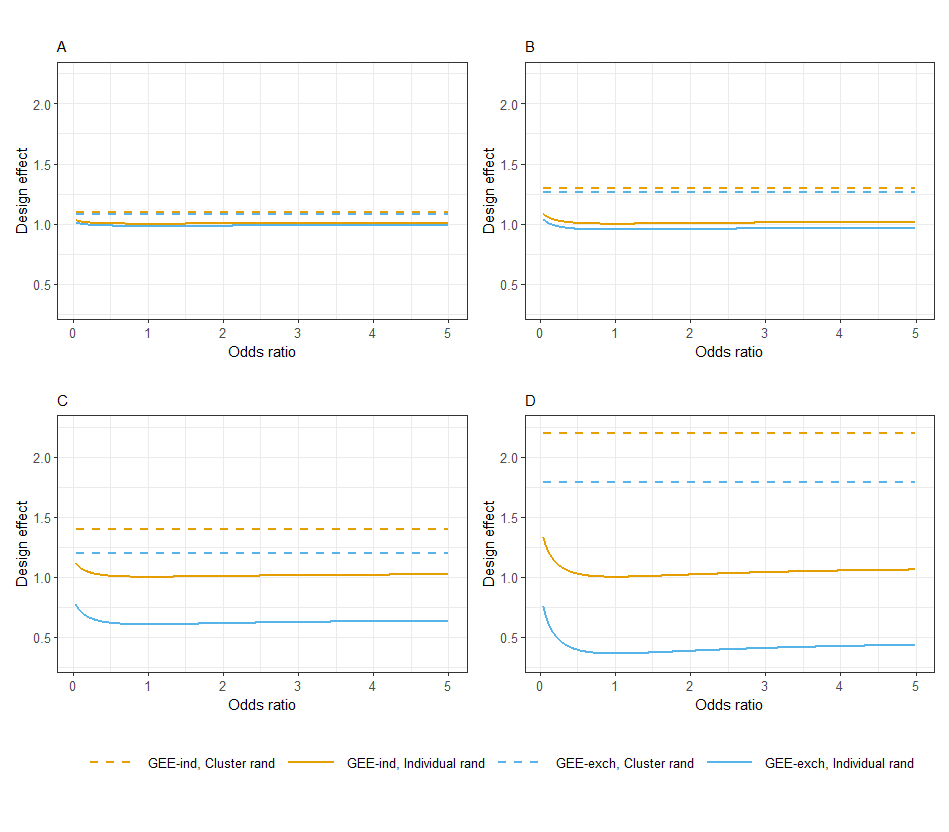


Figure shows the design effects with a maximum cluster size of 4, outcome prevalence in the control group of $\pi_{C}$= 0.1, outcome prevalence in the intervention group ranging from $\pi_{I}$= 0.005 to 0.36 to produce the varying odds ratio, and (A) proportions of clusters of sizes 1-4 of (0.70, 0.15, 0.10. 0.05) and ICC=0.2, (B) proportions of clusters of sizes 1-4 of (0.25, 0.25, 0.25, 0.25) and ICC=0.2, (C) proportions of clusters of sizes 1-4 of (0.70, 0.15, 0.10. 0.05) and ICC=0.8, (D) proportions of clusters of sizes 1-4 of (0.25, 0.25, 0.25, 0.25) and ICC=0.8. Design effects are shown for generalised estimating equations (GEEs) with an independence (GEE-ind) or exchangeable (GEE-exch) working correlation structure under cluster or individual randomisation of the clustered observations.

Supplementary Figure 3: Design effects for a binary outcome with a log link by randomisation method and GEE working correlation structure


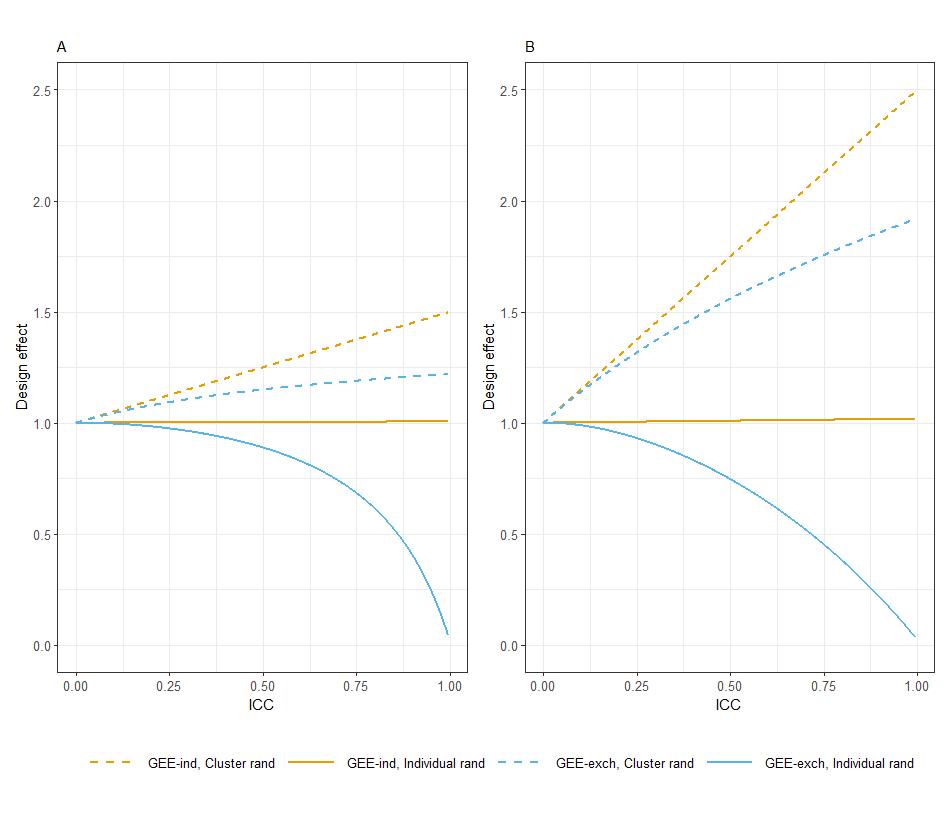


Figure shows the design effects for varying ICC with a maximum cluster size of 4, outcome prevalence of $\pi_{C}$= 0.4 and $\pi_{I}$= 0.3 (relative risk = 0.75), and proportions of clusters of sizes 1-4 of (A) (0.70, 0.15, 0.10. 0.05) (corresponding to $\boldsymbol{\gamma}$ = (0.47, 0.20, 0.20, 0.13)) and (B) (0.25, 0.25, 0.25, 0.25) (corresponding to $\boldsymbol{\gamma}$ = (0.1, 0.2, 0.3, 0.4)). Design effects are shown for generalised estimating equations (GEEs) with an independence (GEE-ind) or exchangeable (GEE-exch) working correlation structure under cluster or individual randomisation of the clustered observations.

Supplementary Figure 4: Design effects for a binary outcome with a log link for varying relative risks when $\boldsymbol{\pi}_{\boldsymbol{c}}$= 0.4


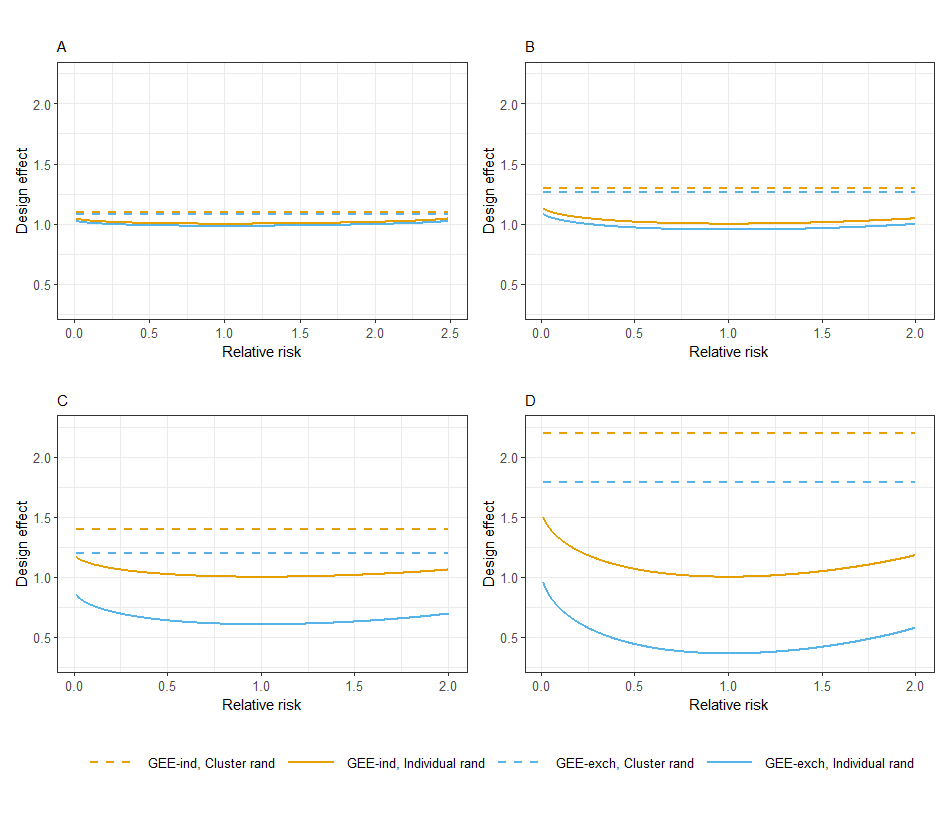


Figure shows the design effects for varying relative risk with a maximum cluster size of 4, outcome prevalence in the control group of $\pi_{C}$= 0.4, outcome prevalence in the intervention group ranging from $\pi_{I}$= 0.005 to 0.80 to produce the varying relative risk, and (A) proportions of clusters of sizes 1-4 of (0.70, 0.15, 0.10. 0.05) (corresponding to $\boldsymbol{\gamma}$ = (0.47, 0.20, 0.20, 0.13)) and ICC=0.2, (B) proportions of clusters of sizes 1-4 of (0.25, 0.25, 0.25, 0.25) (corresponding to $\boldsymbol{\gamma}$ = (0.1, 0.2, 0.3, 0.4)) and ICC=0.2, (C) proportions of clusters of sizes 1-4 of (0.70, 0.15, 0.10. 0.05) (corresponding to $\boldsymbol{\gamma}$ = (0.47, 0.20, 0.20, 0.13)) and ICC=0.8, (D) proportions of clusters of sizes 1-4 of (0.25, 0.25, 0.25, 0.25) (corresponding to $\boldsymbol{\gamma}$ = (0.1, 0.2, 0.3, 0.4)) and ICC=0.8. Design effects are shown for generalised estimating equations (GEEs) with an independence (GEE-ind) or exchangeable (GEE-exch) working correlation structure under cluster or individual randomisation of the clustered observations.

Supplementary Figure 5: Design effects for a binary outcome with a log link for varying relative risks when $\boldsymbol{\pi}_{\boldsymbol{c}}$= 0.1


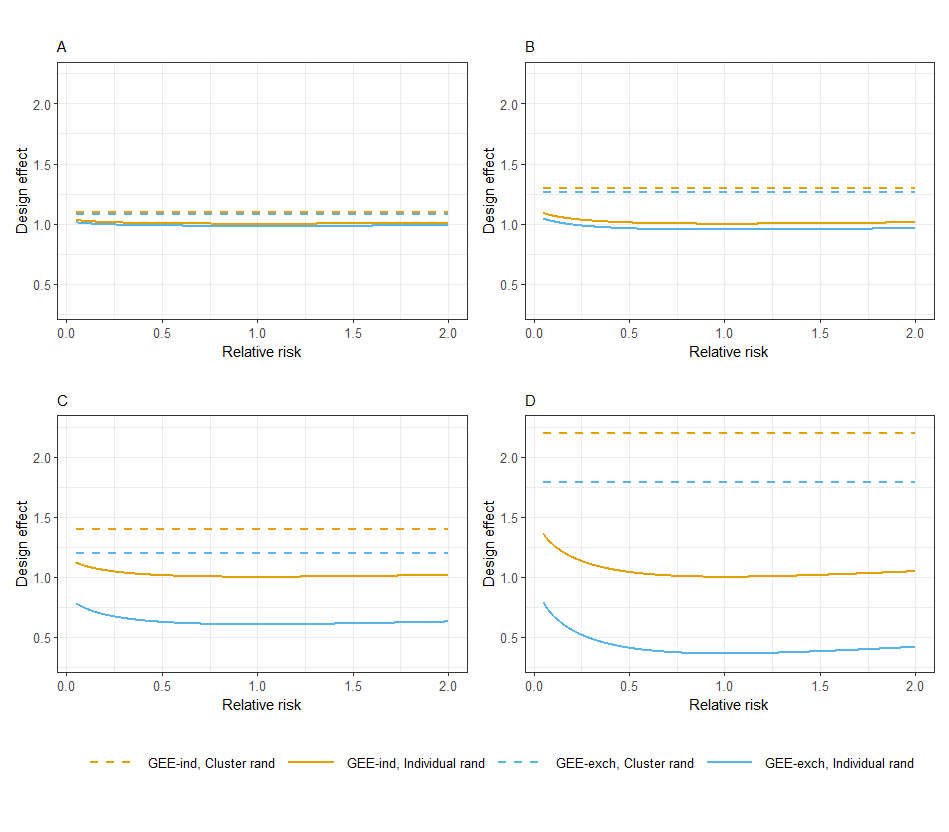


Figure shows the design effects for varying relative risk with a maximum cluster size of 4, outcome prevalence in the control group of $\pi_{C}$= 0.1, outcome prevalence in the intervention group ranging from $\pi_{I}$= 0.005 to 0.20 to produce the varying relative risk, and (A) proportions of clusters of sizes 1-4 of (0.70, 0.15, 0.10. 0.05) (corresponding to $\boldsymbol{\gamma}$ = (0.47, 0.20, 0.20, 0.13)) and ICC=0.2, (B) proportions of clusters of sizes 1-4 of (0.25, 0.25, 0.25, 0.25) (corresponding to $\boldsymbol{\gamma}$ = (0.1, 0.2, 0.3, 0.4)) and ICC=0.2, (C) proportions of clusters of sizes 1-4 of (0.70, 0.15, 0.10. 0.05) (corresponding to $\boldsymbol{\gamma}$ = (0.47, 0.20, 0.20, 0.13)) and ICC=0.8, (D) proportions of clusters of sizes 1-4 of (0.25, 0.25, 0.25, 0.25) (corresponding to $\boldsymbol{\gamma}$ = (0.1, 0.2, 0.3, 0.4)) and ICC=0.8. Design effects are shown for generalised estimating equations (GEEs) with an independence (GEE-ind) or exchangeable (GEE-exch) working correlation structure under cluster or individual randomisation of the clustered observations.
