# Supplementary material for: Sample Size Calculations for Partially Clustered Trials
Source: Stat Med. 2025 Jul 15;44(15-17):e70172. doi: 10.1002/sim.70172 (PMC12261973; doi:10.1002/sim.70172)
Supplement: Supplementary file 3 — File S3. Sensitivity analyses. [file SIM-44-0-s003.docx]

**Supplementary File 3 – Sensitivity analyses**

**Title:** Sample size calculations for partially clustered trials

**Authors:** Kylie M. Lange, Jessica Kasza, Thomas R. Sullivan, Lisa N. Yelland

# Sensitivity analysis 1:

The simulation study was repeated by relaxing the assumption of treatment balance within cluster size. For cluster randomisation, an equal number of clusters were assigned to each treatment group without stratification by cluster size. This resulted in the treatment groups being balanced overall on average, but the assumptions of overall treatment group balance and treatment group balance within cluster size were not guaranteed in individual datasets. For individual randomisation, treatment groups were balanced overall for each dataset, but not within cluster size.

Results are presented in Supplementary Tables 4 – 9.

# Sensitivity analysis 2:

In a second sensitivity analysis, instead of the number of clusters of each size being constant across replicated datasets for each scenario, the $M\_k$ were generated randomly from a multinomial distribution with the total number of clusters ($M$) fixed at the same values used in the main simulation study, and probabilities equal to $\boldsymbol{\delta}=(\delta_{1}, \ldots, \delta_{K})$. The total number of observations ($N$) therefore varied across datasets, but equalled the sample size used in the main simulation study on average.

Results are presented in Supplementary Tables 10 – 15.

Supplementary Table 4: Observed and expected design effects for a continuous outcome, without treatment balance within cluster size

|  | | | | | GEE independence | | | GEE exchangeable | | |
| --- | --- | --- | --- | --- | --- | --- | --- | --- | --- | --- |
| Randomisation method | Sample size | Distribution of cluster sizes | ICC | Observed DEFF* | | Expected DEFF | Relative difference (%)* | Observed DEFF* | Expected DEFF | Relative difference (%)* |
| Cluster | 240 | Unequal cluster proportions | 0.2 | 1.17 | | 1.20 | -2.47 | 1.14 | 1.16 | -2.11 |
| Cluster | 240 | Unequal cluster proportions | 0.8 | 1.75 | | 1.80 | -3.04 | 1.43 | 1.44 | -0.71 |
| Cluster | 240 | Equal cluster proportions | 0.2 | 1.36 | | 1.40 | -2.76 | 1.33 | 1.37 | -2.49 |
| Cluster | 240 | Equal cluster proportions | 0.8 | 2.54 | | 2.60 | -2.30 | 2.22 | 2.25 | -1.33 |
| Cluster | 600 | Unequal cluster proportions | 0.2 | 1.19 | | 1.20 | -1.04 | 1.15 | 1.16 | -0.80 |
| Cluster | 600 | Unequal cluster proportions | 0.8 | 1.78 | | 1.80 | -1.29 | 1.44 | 1.44 | -0.24 |
| Cluster | 600 | Equal cluster proportions | 0.2 | 1.38 | | 1.40 | -1.22 | 1.35 | 1.37 | -1.04 |
| Cluster | 600 | Equal cluster proportions | 0.8 | 2.58 | | 2.60 | -0.94 | 2.24 | 2.25 | -0.51 |
| Individual | 240 | Unequal cluster proportions | 0.2 | 0.98 | | 1.00 | -1.59 | 0.95 | 0.97 | -1.64 |
| Individual | 240 | Unequal cluster proportions | 0.8 | 0.97 | | 1.00 | -2.57 | 0.48 | 0.45 | 7.76 |
| Individual | 240 | Equal cluster proportions | 0.2 | 0.98 | | 1.00 | -2.38 | 0.91 | 0.94 | -2.48 |
| Individual | 240 | Equal cluster proportions | 0.8 | 0.96 | | 1.00 | -3.58 | 0.31 | 0.31 | -0.60 |
| Individual | 600 | Unequal cluster proportions | 0.2 | 0.99 | | 1.00 | -0.62 | 0.96 | 0.97 | -0.62 |
| Individual | 600 | Unequal cluster proportions | 0.8 | 0.99 | | 1.00 | -0.95 | 0.46 | 0.45 | 3.13 |
| Individual | 600 | Equal cluster proportions | 0.2 | 0.99 | | 1.00 | -0.97 | 0.93 | 0.94 | -0.92 |
| Individual | 600 | Equal cluster proportions | 0.8 | 0.98 | | 1.00 | -1.66 | 0.31 | 0.31 | 0.09 |

* Median value across 10000 simulated datasets.

Clusters of size 1-4 were distributed in proportions (0.70, 0.15, 0.10, 0.05) in scenarios with unequal cluster proportions (corresponding to $\boldsymbol{\gamma}$ = (0.47, 0.20, 0.20, 0.13)), and (0.25, 0.25, 0.25, 0.25) in scenarios with equal cluster proportions (corresponding to $\boldsymbol{\gamma}$ = (0.1, 0.2, 0.3, 0.4)).

GEE = generalised estimating equation, DEFF = design effect, ICC = intracluster correlation coefficient

.

Supplementary Table 5: Observed and expected power for a continuous outcome, without treatment balance within cluster size

|  | | | | GEE independence | | | GEE exchangeable | | |
| --- | --- | --- | --- | --- | --- | --- | --- | --- | --- |
| Randomisation method | Sample size | Distribution of cluster sizes | ICC | Observed power | Expected power | Absolute difference | Observed power | Expected power | Absolute difference |
| Cluster | 240 | Unequal cluster proportions | 0.2 | 42.78 | 42.05 | 0.73 | 44.33 | 43.14 | 1.19 |
| Cluster | 240 | Unequal cluster proportions | 0.8 | 31.57 | 29.94 | 1.63 | 37.07 | 36.10 | 0.97 |
| Cluster | 240 | Equal cluster proportions | 0.2 | 39.21 | 36.99 | 2.22 | 40.03 | 37.71 | 2.32 |
| Cluster | 240 | Equal cluster proportions | 0.8 | 24.12 | 22.10 | 2.02 | 27.05 | 24.85 | 2.20 |
| Cluster | 600 | Unequal cluster proportions | 0.2 | 80.29 | 79.67 | 0.62 | 81.69 | 80.88 | 0.81 |
| Cluster | 600 | Unequal cluster proportions | 0.8 | 63.34 | 62.39 | 0.95 | 72.37 | 72.06 | 0.31 |
| Cluster | 600 | Equal cluster proportions | 0.2 | 74.30 | 73.30 | 1.00 | 75.37 | 74.28 | 1.09 |
| Cluster | 600 | Equal cluster proportions | 0.8 | 47.94 | 47.25 | 0.69 | 53.33 | 52.92 | 0.41 |
| Individual | 240 | Unequal cluster proportions | 0.2 | 49.66 | 48.76 | 0.90 | 50.97 | 50.11 | 0.86 |
| Individual | 240 | Unequal cluster proportions | 0.8 | 50.07 | 48.76 | 1.31 | 78.50 | 82.20 | -3.70 |
| Individual | 240 | Equal cluster proportions | 0.2 | 50.21 | 48.76 | 1.45 | 52.49 | 51.32 | 1.17 |
| Individual | 240 | Equal cluster proportions | 0.8 | 51.14 | 48.76 | 2.38 | 93.47 | 93.48 | -0.01 |
| Individual | 600 | Unequal cluster proportions | 0.2 | 87.08 | 86.37 | 0.71 | 88.54 | 87.50 | 1.04 |
| Individual | 600 | Unequal cluster proportions | 0.8 | 86.54 | 86.37 | 0.17 | 99.19 | 99.54 | -0.35 |
| Individual | 600 | Equal cluster proportions | 0.2 | 86.93 | 86.37 | 0.56 | 88.87 | 88.46 | 0.41 |
| Individual | 600 | Equal cluster proportions | 0.8 | 86.87 | 86.37 | 0.50 | 99.98 | 99.98 | 0.00 |

Clusters of size 1-4 were distributed in proportions (0.70, 0.15, 0.10, 0.05) in scenarios with unequal cluster proportions (corresponding to $\boldsymbol{\gamma}$ = (0.47, 0.20, 0.20, 0.13)), and (0.25, 0.25, 0.25, 0.25) in scenarios with equal cluster proportions (corresponding to $\boldsymbol{\gamma}$ = (0.1, 0.2, 0.3, 0.4)).

GEE = generalised estimating equation, ICC = intracluster correlation coefficient

**Supplementary Table 6: Observed and expected design effects for a binary outcome with a logit link, without treatment balance within cluster size**

|  | | | | GEE independence | | | GEE exchangeable | | |
| --- | --- | --- | --- | --- | --- | --- | --- | --- | --- |
| Randomisation method | Sample size | Distribution of cluster sizes | ICC | Observed DEFF* | Expected DEFF | Relative difference (%)* | Observed DEFF* | Expected DEFF | Relative difference (%)* |
| Cluster | 360 | Unequal cluster proportions | 0.2 | 1.19 | 1.20 | -1.03 | 1.16 | 1.16 | -0.62 |
| Cluster | 360 | Unequal cluster proportions | 0.8 | 1.78 | 1.80 | -0.98 | 1.43 | 1.44 | -0.45 |
| Cluster | 360 | Equal cluster proportions | 0.2 | 1.38 | 1.40 | -1.36 | 1.35 | 1.37 | -1.12 |
| Cluster | 360 | Equal cluster proportions | 0.8 | 2.58 | 2.60 | -0.81 | 2.24 | 2.25 | -0.58 |
| Cluster | 840 | Unequal cluster proportions | 0.2 | 1.19 | 1.20 | -0.47 | 1.16 | 1.16 | -0.28 |
| Cluster | 840 | Unequal cluster proportions | 0.8 | 1.79 | 1.80 | -0.50 | 1.44 | 1.44 | -0.19 |
| Cluster | 840 | Equal cluster proportions | 0.2 | 1.39 | 1.40 | -0.48 | 1.36 | 1.37 | -0.41 |
| Cluster | 840 | Equal cluster proportions | 0.8 | 2.59 | 2.60 | -0.29 | 2.25 | 2.25 | -0.19 |
| Individual | 360 | Unequal cluster proportions | 0.2 | 0.99 | 1.00 | -0.55 | 0.96 | 0.97 | -0.67 |
| Individual | 360 | Unequal cluster proportions | 0.8 | 0.99 | 1.00 | -0.96 | 0.45 | 0.45 | 0.18 |
| Individual | 360 | Equal cluster proportions | 0.2 | 0.99 | 1.00 | -1.10 | 0.93 | 0.94 | -1.33 |
| Individual | 360 | Equal cluster proportions | 0.8 | 0.98 | 1.00 | -2.09 | 0.30 | 0.31 | -2.73 |
| Individual | 840 | Unequal cluster proportions | 0.2 | 1.00 | 1.00 | -0.20 | 0.96 | 0.97 | -0.29 |
| Individual | 840 | Unequal cluster proportions | 0.8 | 1.00 | 1.00 | -0.41 | 0.45 | 0.45 | 0.05 |
| Individual | 840 | Equal cluster proportions | 0.2 | 1.00 | 1.00 | -0.32 | 0.93 | 0.94 | -0.40 |
| Individual | 840 | Equal cluster proportions | 0.8 | 1.00 | 1.00 | -0.66 | 0.31 | 0.31 | -1.10 |

* Median value across 10000 simulated datasets.

Clusters of size 1-4 were distributed in proportions (0.70, 0.15, 0.10, 0.05) in scenarios with unequal cluster proportions (corresponding to $\boldsymbol{\gamma}$ = (0.47, 0.20, 0.20, 0.13)), and (0.25, 0.25, 0.25, 0.25) in scenarios with equal cluster proportions (corresponding to $\boldsymbol{\gamma}$ = (0.1, 0.2, 0.3, 0.4)).

GEE = generalised estimating equation, DEFF = design effect, ICC = intracluster correlation coefficient.

**Supplementary Table 7: Observed and expected power for a binary outcome with a logit link, without treatment balance within cluster size**

|  | | | | GEE independence | | | GEE exchangeable | | |
| --- | --- | --- | --- | --- | --- | --- | --- | --- | --- |
| Randomisation method | Sample size | Distribution of cluster sizes | ICC | Observed power | Expected power | Absolute difference | Observed power | Expected power | Absolute difference |
| Cluster | 360 | Unequal cluster proportions | 0.2 | 44.28 | 44.05 | 0.23 | 45.55 | 45.17 | 0.38 |
| Cluster | 360 | Unequal cluster proportions | 0.8 | 32.24 | 31.43 | 0.81 | 38.09 | 37.86 | 0.23 |
| Cluster | 360 | Equal cluster proportions | 0.2 | 39.34 | 38.79 | 0.55 | 39.92 | 39.54 | 0.38 |
| Cluster | 360 | Equal cluster proportions | 0.8 | 23.54 | 23.20 | 0.34 | 25.81 | 26.09 | -0.28 |
| Cluster | 840 | Unequal cluster proportions | 0.2 | 79.29 | 79.15 | 0.14 | 80.65 | 80.38 | 0.27 |
| Cluster | 840 | Unequal cluster proportions | 0.8 | 62.25 | 61.82 | 0.43 | 71.84 | 71.50 | 0.34 |
| Cluster | 840 | Equal cluster proportions | 0.2 | 72.74 | 72.75 | -0.01 | 73.82 | 73.73 | 0.09 |
| Cluster | 840 | Equal cluster proportions | 0.8 | 46.66 | 46.77 | -0.11 | 52.74 | 52.39 | 0.35 |
| Individual | 360 | Unequal cluster proportions | 0.2 | 51.57 | 50.95 | 0.62 | 52.81 | 52.34 | 0.47 |
| Individual | 360 | Unequal cluster proportions | 0.8 | 51.68 | 50.93 | 0.75 | 90.31 | 84.18 | 6.13 |
| Individual | 360 | Equal cluster proportions | 0.2 | 51.26 | 50.94 | 0.32 | 53.89 | 53.57 | 0.32 |
| Individual | 360 | Equal cluster proportions | 0.8 | 52.22 | 50.89 | 1.33 | 99.42 | 94.52 | 4.90 |
| Individual | 840 | Unequal cluster proportions | 0.2 | 86.37 | 85.93 | 0.44 | 87.56 | 87.08 | 0.48 |
| Individual | 840 | Unequal cluster proportions | 0.8 | 86.43 | 85.91 | 0.52 | 99.97 | 99.49 | 0.48 |
| Individual | 840 | Equal cluster proportions | 0.2 | 86.54 | 85.92 | 0.62 | 88.43 | 88.05 | 0.38 |
| Individual | 840 | Equal cluster proportions | 0.8 | 85.67 | 85.88 | -0.21 | 100.00 | 99.98 | 0.02 |

Clusters of size 1-4 were distributed in proportions (0.70, 0.15, 0.10, 0.05) in scenarios with unequal cluster proportions (corresponding to $\boldsymbol{\gamma}$ = (0.47, 0.20, 0.20, 0.13)), and (0.25, 0.25, 0.25, 0.25) in scenarios with equal cluster proportions (corresponding to $\boldsymbol{\gamma}$ = (0.1, 0.2, 0.3, 0.4)).

GEE = generalised estimating equation, ICC = intracluster correlation coefficient.

Supplementary Table 8: Observed and expected design effects for a binary outcome with a log link, without treatment balance within cluster size

|  | | | | | GEE independence | | | GEE exchangeable | | |
| --- | --- | --- | --- | --- | --- | --- | --- | --- | --- | --- |
| Randomisation method | Sample size | Distribution of cluster sizes | ICC | Observed DEFF* | | Expected DEFF | Relative difference (%)* | Observed DEFF* | Expected DEFF | Relative difference (%)* |
| Cluster | 360 | Unequal cluster proportions | 0.2 | 1.19 | | 1.20 | -1.15 | 1.15 | 1.16 | -0.91 |
| Cluster | 360 | Unequal cluster proportions | 0.8 | 1.78 | | 1.80 | -1.08 | 1.43 | 1.44 | -0.84 |
| Cluster | 360 | Equal cluster proportions | 0.2 | 1.38 | | 1.40 | -1.41 | 1.35 | 1.37 | -1.28 |
| Cluster | 360 | Equal cluster proportions | 0.8 | 2.58 | | 2.60 | -0.86 | 2.23 | 2.25 | -0.86 |
| Cluster | 840 | Unequal cluster proportions | 0.2 | 1.19 | | 1.20 | -0.45 | 1.16 | 1.16 | -0.40 |
| Cluster | 840 | Unequal cluster proportions | 0.8 | 1.79 | | 1.80 | -0.52 | 1.44 | 1.44 | -0.37 |
| Cluster | 840 | Equal cluster proportions | 0.2 | 1.39 | | 1.40 | -0.55 | 1.36 | 1.37 | -0.44 |
| Cluster | 840 | Equal cluster proportions | 0.8 | 2.59 | | 2.60 | -0.29 | 2.24 | 2.25 | -0.31 |
| Individual | 360 | Unequal cluster proportions | 0.2 | 1.00 | | 1.00 | -0.58 | 0.96 | 0.97 | -0.61 |
| Individual | 360 | Unequal cluster proportions | 0.8 | 1.00 | | 1.01 | -0.86 | 0.46 | 0.46 | -0.49 |
| Individual | 360 | Equal cluster proportions | 0.2 | 0.99 | | 1.00 | -1.11 | 0.93 | 0.94 | -1.29 |
| Individual | 360 | Equal cluster proportions | 0.8 | 1.00 | | 1.02 | -1.86 | 0.32 | 0.33 | -3.13 |
| Individual | 840 | Unequal cluster proportions | 0.2 | 1.00 | | 1.00 | -0.23 | 0.97 | 0.97 | -0.20 |
| Individual | 840 | Unequal cluster proportions | 0.8 | 1.01 | | 1.01 | -0.37 | 0.46 | 0.46 | -0.32 |
| Individual | 840 | Equal cluster proportions | 0.2 | 1.00 | | 1.00 | -0.32 | 0.94 | 0.94 | -0.30 |
| Individual | 840 | Equal cluster proportions | 0.8 | 1.01 | | 1.02 | -0.56 | 0.33 | 0.33 | -1.33 |

* Median value across 10000 simulated datasets.

Clusters of size 1-4 were distributed in proportions (0.70, 0.15, 0.10, 0.05) in scenarios with unequal cluster proportions (corresponding to $\boldsymbol{\gamma}$ = (0.47, 0.20, 0.20, 0.13)), and (0.25, 0.25, 0.25, 0.25) in scenarios with equal cluster proportions (corresponding to $\boldsymbol{\gamma}$ = (0.1, 0.2, 0.3, 0.4)).

GEE = generalised estimating equation, DEFF = design effect, ICC = intracluster correlation coefficient.

Supplementary Table 9: Observed and expected power for a binary outcome with a log link, without treatment balance within cluster size

|  | | | | GEE independence | | | GEE exchangeable | | |
| --- | --- | --- | --- | --- | --- | --- | --- | --- | --- |
| Randomisation method | Sample size | Distribution of cluster sizes | ICC | Observed power | Expected power | Absolute difference | Observed power | Expected power | Absolute difference |
| Cluster | 360 | Unequal cluster proportions | 0.2 | 43.83 | 44.67 | -0.84 | 45.01 | 45.80 | -0.79 |
| Cluster | 360 | Unequal cluster proportions | 0.8 | 31.50 | 31.97 | -0.47 | 37.34 | 38.45 | -1.11 |
| Cluster | 360 | Equal cluster proportions | 0.2 | 38.55 | 39.38 | -0.83 | 39.34 | 40.14 | -0.80 |
| Cluster | 360 | Equal cluster proportions | 0.8 | 22.58 | 23.66 | -1.08 | 25.09 | 26.58 | -1.49 |
| Cluster | 840 | Unequal cluster proportions | 0.2 | 79.11 | 79.64 | -0.53 | 80.51 | 80.85 | -0.34 |
| Cluster | 840 | Unequal cluster proportions | 0.8 | 61.98 | 62.44 | -0.46 | 71.44 | 72.07 | -0.63 |
| Cluster | 840 | Equal cluster proportions | 0.2 | 72.45 | 73.30 | -0.85 | 73.65 | 74.28 | -0.63 |
| Cluster | 840 | Equal cluster proportions | 0.8 | 46.17 | 47.40 | -1.23 | 52.13 | 53.03 | -0.90 |
| Individual | 360 | Unequal cluster proportions | 0.2 | 50.94 | 51.51 | -0.57 | 52.33 | 52.89 | -0.56 |
| Individual | 360 | Unequal cluster proportions | 0.8 | 50.95 | 51.22 | -0.27 | 89.87 | 83.75 | 6.12 |
| Individual | 360 | Equal cluster proportions | 0.2 | 50.55 | 51.41 | -0.86 | 53.16 | 54.01 | -0.85 |
| Individual | 360 | Equal cluster proportions | 0.8 | 51.08 | 50.85 | 0.23 | 99.39 | 93.37 | 6.02 |
| Individual | 840 | Unequal cluster proportions | 0.2 | 86.27 | 86.25 | 0.02 | 87.41 | 87.38 | 0.03 |
| Individual | 840 | Unequal cluster proportions | 0.8 | 86.11 | 86.01 | 0.10 | 99.97 | 99.44 | 0.53 |
| Individual | 840 | Equal cluster proportions | 0.2 | 86.39 | 86.17 | 0.22 | 88.30 | 88.25 | 0.05 |
| Individual | 840 | Equal cluster proportions | 0.8 | 85.39 | 85.69 | -0.30 | 100.00 | 99.96 | 0.04 |

Clusters of size 1-4 were distributed in proportions (0.70, 0.15, 0.10, 0.05) in scenarios with unequal cluster proportions (corresponding to $\boldsymbol{\gamma}$ = (0.47, 0.20, 0.20, 0.13)), and (0.25, 0.25, 0.25, 0.25) in scenarios with equal cluster proportions (corresponding to $\boldsymbol{\gamma}$ = (0.1, 0.2, 0.3, 0.4)).

GEE = generalised estimating equation, ICC = intracluster correlation coefficient.

Supplementary Table 10: Observed and expected design effects for a continuous outcome, with a varying number of clusters of each size

|  | | | | | GEE independence | | | GEE exchangeable | | |
| --- | --- | --- | --- | --- | --- | --- | --- | --- | --- | --- |
| Randomisation method | Number of clusters | Distribution of cluster sizes | ICC | Observed DEFF* | | Expected DEFF | Relative difference (%)* | Observed DEFF* | Expected DEFF | Relative difference (%)* |
| Cluster | 160 | Unequal cluster proportions | 0.2 | 1.17 | | 1.20 | -2.51 | 1.14 | 1.16 | -2.14 |
| Cluster | 160 | Unequal cluster proportions | 0.8 | 1.75 | | 1.80 | -2.80 | 1.43 | 1.44 | -1.04 |
| Cluster | 96 | Equal cluster proportions | 0.2 | 1.36 | | 1.40 | -2.93 | 1.33 | 1.37 | -2.62 |
| Cluster | 96 | Equal cluster proportions | 0.8 | 2.54 | | 2.60 | -2.27 | 2.23 | 2.25 | -1.14 |
| Cluster | 400 | Unequal cluster proportions | 0.2 | 1.19 | | 1.20 | -0.97 | 1.15 | 1.16 | -0.84 |
| Cluster | 400 | Unequal cluster proportions | 0.8 | 1.78 | | 1.80 | -1.27 | 1.44 | 1.44 | -0.31 |
| Cluster | 240 | Equal cluster proportions | 0.2 | 1.38 | | 1.40 | -1.18 | 1.35 | 1.37 | -1.02 |
| Cluster | 240 | Equal cluster proportions | 0.8 | 2.58 | | 2.60 | -0.85 | 2.24 | 2.25 | -0.52 |
| Individual | 160 | Unequal cluster proportions | 0.2 | 0.98 | | 1.00 | -2.06 | 0.94 | 0.97 | -2.18 |
| Individual | 160 | Unequal cluster proportions | 0.8 | 0.96 | | 1.00 | -4.26 | 0.48 | 0.45 | 6.87 |
| Individual | 96 | Equal cluster proportions | 0.2 | 0.97 | | 1.00 | -2.75 | 0.91 | 0.94 | -2.77 |
| Individual | 96 | Equal cluster proportions | 0.8 | 0.95 | | 1.00 | -5.16 | 0.31 | 0.31 | -1.65 |
| Individual | 400 | Unequal cluster proportions | 0.2 | 0.99 | | 1.00 | -0.80 | 0.96 | 0.97 | -0.70 |
| Individual | 400 | Unequal cluster proportions | 0.8 | 0.98 | | 1.00 | -1.94 | 0.46 | 0.45 | 2.60 |
| Individual | 240 | Equal cluster proportions | 0.2 | 0.99 | | 1.00 | -0.89 | 0.93 | 0.94 | -0.89 |
| Individual | 240 | Equal cluster proportions | 0.8 | 0.98 | | 1.00 | -2.06 | 0.31 | 0.31 | -0.51 |

* Median value across 10000 simulated datasets.

Clusters of size 1-4 were distributed in proportions (0.70, 0.15, 0.10, 0.05) in scenarios with unequal cluster proportions (corresponding to $\boldsymbol{\gamma}$ = (0.47, 0.20, 0.20, 0.13)), and (0.25, 0.25, 0.25, 0.25) in scenarios with equal cluster proportions (corresponding to $\boldsymbol{\gamma}$ = (0.1, 0.2, 0.3, 0.4)).

GEE = generalised estimating equation, DEFF = design effect, ICC = intracluster correlation coefficient

Supplementary Table 11: Observed and expected power for a continuous outcome, with a varying number of clusters of each size

|  | | | | GEE independence | | | GEE exchangeable | | |
| --- | --- | --- | --- | --- | --- | --- | --- | --- | --- |
| Randomisation method | Number of clusters | Distribution of cluster sizes | ICC | Observed power | Expected power | Absolute difference | Observed power | Expected power | Absolute difference |
| Cluster | 160 | Unequal cluster proportions | 0.2 | 43.76 | 42.05 | 1.71 | 44.79 | 43.14 | 1.65 |
| Cluster | 160 | Unequal cluster proportions | 0.8 | 30.90 | 29.94 | 0.96 | 36.50 | 36.10 | 0.40 |
| Cluster | 96 | Equal cluster proportions | 0.2 | 39.73 | 36.99 | 2.74 | 40.48 | 37.71 | 2.77 |
| Cluster | 96 | Equal cluster proportions | 0.8 | 23.66 | 22.10 | 1.56 | 25.97 | 24.85 | 1.12 |
| Cluster | 400 | Unequal cluster proportions | 0.2 | 80.06 | 79.67 | 0.39 | 81.33 | 80.88 | 0.45 |
| Cluster | 400 | Unequal cluster proportions | 0.8 | 63.40 | 62.39 | 1.01 | 72.85 | 72.06 | 0.79 |
| Cluster | 240 | Equal cluster proportions | 0.2 | 74.24 | 73.30 | 0.94 | 75.26 | 74.28 | 0.98 |
| Cluster | 240 | Equal cluster proportions | 0.8 | 47.97 | 47.25 | 0.72 | 53.56 | 52.92 | 0.64 |
| Individual | 160 | Unequal cluster proportions | 0.2 | 49.86 | 48.76 | 1.10 | 51.34 | 50.11 | 1.23 |
| Individual | 160 | Unequal cluster proportions | 0.8 | 51.65 | 48.76 | 2.89 | 79.05 | 82.20 | -3.15 |
| Individual | 96 | Equal cluster proportions | 0.2 | 50.86 | 48.76 | 2.10 | 52.95 | 51.32 | 1.63 |
| Individual | 96 | Equal cluster proportions | 0.8 | 52.02 | 48.76 | 3.26 | 93.78 | 93.48 | 0.30 |
| Individual | 400 | Unequal cluster proportions | 0.2 | 85.97 | 86.37 | -0.40 | 86.96 | 87.50 | -0.54 |
| Individual | 400 | Unequal cluster proportions | 0.8 | 87.05 | 86.37 | 0.68 | 99.27 | 99.54 | -0.27 |
| Individual | 240 | Equal cluster proportions | 0.2 | 86.36 | 86.37 | -0.01 | 88.46 | 88.46 | -0.00 |
| Individual | 240 | Equal cluster proportions | 0.8 | 86.46 | 86.37 | 0.09 | 99.97 | 99.98 | -0.01 |

Clusters of size 1-4 were distributed in proportions (0.70, 0.15, 0.10, 0.05) in scenarios with unequal cluster proportions (corresponding to $\boldsymbol{\gamma}$ = (0.47, 0.20, 0.20, 0.13)), and (0.25, 0.25, 0.25, 0.25) in scenarios with equal cluster proportions (corresponding to $\boldsymbol{\gamma}$ = (0.1, 0.2, 0.3, 0.4)).

GEE = generalised estimating equation, ICC = intracluster correlation coefficient

**Supplementary Table 12: Observed and expected design effects for a binary outcome with a logit link, with a varying number of clusters of each size**

|  | | | | GEE independence | | | GEE exchangeable | | |
| --- | --- | --- | --- | --- | --- | --- | --- | --- | --- |
| Randomisation method | Number of clusters | Distribution of cluster sizes | ICC | Observed DEFF* | Expected DEFF | Relative difference (%)* | Observed DEFF* | Expected DEFF | Relative difference (%)* |
| Cluster | 240 | Unequal cluster proportions | 0.2 | 1.19 | 1.20 | -1.01 | 1.16 | 1.16 | -0.56 |
| Cluster | 240 | Unequal cluster proportions | 0.8 | 1.78 | 1.80 | -0.93 | 1.44 | 1.44 | -0.27 |
| Cluster | 144 | Equal cluster proportions | 0.2 | 1.38 | 1.40 | -1.50 | 1.35 | 1.37 | -1.23 |
| Cluster | 144 | Equal cluster proportions | 0.8 | 2.58 | 2.60 | -0.69 | 2.24 | 2.25 | -0.51 |
| Cluster | 560 | Unequal cluster proportions | 0.2 | 1.19 | 1.20 | -0.43 | 1.16 | 1.16 | -0.27 |
| Cluster | 560 | Unequal cluster proportions | 0.8 | 1.79 | 1.80 | -0.44 | 1.44 | 1.44 | -0.20 |
| Cluster | 336 | Equal cluster proportions | 0.2 | 1.39 | 1.40 | -0.77 | 1.36 | 1.37 | -0.59 |
| Cluster | 336 | Equal cluster proportions | 0.8 | 2.59 | 2.60 | -0.30 | 2.25 | 2.25 | -0.18 |
| Individual | 240 | Unequal cluster proportions | 0.2 | 0.99 | 1.00 | -0.98 | 0.96 | 0.97 | -1.09 |
| Individual | 240 | Unequal cluster proportions | 0.8 | 0.98 | 1.00 | -2.35 | 0.45 | 0.45 | -1.11 |
| Individual | 144 | Equal cluster proportions | 0.2 | 0.99 | 1.00 | -1.41 | 0.92 | 0.94 | -1.71 |
| Individual | 144 | Equal cluster proportions | 0.8 | 0.98 | 1.00 | -2.30 | 0.30 | 0.31 | -3.07 |
| Individual | 560 | Unequal cluster proportions | 0.2 | 1.00 | 1.00 | -0.38 | 0.96 | 0.97 | -0.35 |
| Individual | 560 | Unequal cluster proportions | 0.8 | 0.99 | 1.00 | -0.87 | 0.45 | 0.45 | -0.45 |
| Individual | 336 | Equal cluster proportions | 0.2 | 0.99 | 1.00 | -0.64 | 0.93 | 0.94 | -0.80 |
| Individual | 336 | Equal cluster proportions | 0.8 | 0.99 | 1.00 | -1.05 | 0.31 | 0.31 | -1.25 |

* Median value across 10000 simulated datasets.

Clusters of size 1-4 were distributed in proportions (0.70, 0.15, 0.10, 0.05) in scenarios with unequal cluster proportions (corresponding to $\boldsymbol{\gamma}$ = (0.47, 0.20, 0.20, 0.13)), and (0.25, 0.25, 0.25, 0.25) in scenarios with equal cluster proportions (corresponding to $\boldsymbol{\gamma}$ = (0.1, 0.2, 0.3, 0.4)).

GEE = generalised estimating equation, DEFF = design effect, ICC = intracluster correlation coefficient

.

**Supplementary Table 13: Observed and expected power for a binary outcome with a logit link, with a varying number of clusters of each size**

|  | | | | GEE independence | | | GEE exchangeable | | |
| --- | --- | --- | --- | --- | --- | --- | --- | --- | --- |
| Randomisation method | Number of clusters | Distribution of cluster sizes | ICC | Observed power | Expected power | Absolute difference | Observed power | Expected power | Absolute difference |
| Cluster | 240 | Unequal cluster proportions | 0.2 | 44.55 | 44.05 | 0.50 | 45.80 | 45.17 | 0.63 |
| Cluster | 240 | Unequal cluster proportions | 0.8 | 31.90 | 31.43 | 0.47 | 38.13 | 37.86 | 0.27 |
| Cluster | 144 | Equal cluster proportions | 0.2 | 39.35 | 38.79 | 0.56 | 40.37 | 39.54 | 0.83 |
| Cluster | 144 | Equal cluster proportions | 0.8 | 24.54 | 23.20 | 1.34 | 27.47 | 26.09 | 1.38 |
| Cluster | 560 | Unequal cluster proportions | 0.2 | 79.87 | 79.15 | 0.72 | 81.19 | 80.38 | 0.81 |
| Cluster | 560 | Unequal cluster proportions | 0.8 | 62.26 | 61.82 | 0.44 | 71.91 | 71.50 | 0.41 |
| Cluster | 336 | Equal cluster proportions | 0.2 | 72.95 | 72.75 | 0.20 | 73.97 | 73.73 | 0.24 |
| Cluster | 336 | Equal cluster proportions | 0.8 | 46.49 | 46.77 | -0.28 | 52.32 | 52.39 | -0.07 |
| Individual | 240 | Unequal cluster proportions | 0.2 | 52.04 | 50.95 | 1.09 | 53.50 | 52.34 | 1.16 |
| Individual | 240 | Unequal cluster proportions | 0.8 | 52.41 | 50.93 | 1.48 | 90.41 | 84.18 | 6.23 |
| Individual | 144 | Equal cluster proportions | 0.2 | 51.47 | 50.94 | 0.53 | 54.04 | 53.57 | 0.47 |
| Individual | 144 | Equal cluster proportions | 0.8 | 52.59 | 50.89 | 1.70 | 99.27 | 94.52 | 4.75 |
| Individual | 560 | Unequal cluster proportions | 0.2 | 86.12 | 85.93 | 0.19 | 87.30 | 87.08 | 0.22 |
| Individual | 560 | Unequal cluster proportions | 0.8 | 86.12 | 85.91 | 0.21 | 99.96 | 99.49 | 0.47 |
| Individual | 336 | Equal cluster proportions | 0.2 | 85.75 | 85.92 | -0.17 | 88.07 | 88.05 | 0.02 |
| Individual | 336 | Equal cluster proportions | 0.8 | 85.82 | 85.88 | -0.06 | 100.00 | 99.98 | 0.02 |

Clusters of size 1-4 were distributed in proportions (0.70, 0.15, 0.10, 0.05) in scenarios with unequal cluster proportions (corresponding to $\boldsymbol{\gamma}$ = (0.47, 0.20, 0.20, 0.13)), and (0.25, 0.25, 0.25, 0.25) in scenarios with equal cluster proportions (corresponding to $\boldsymbol{\gamma}$ = (0.1, 0.2, 0.3, 0.4)).

GEE = generalised estimating equation, ICC = intracluster correlation coefficient.

Supplementary Table 14: Observed and expected design effects for a binary outcome with a log link, with a varying number of clusters of each size

|  | | | | | GEE independence | | | GEE exchangeable | | |
| --- | --- | --- | --- | --- | --- | --- | --- | --- | --- | --- |
| Randomisation method | Number of clusters | Distribution of cluster sizes | ICC | Observed DEFF* | | Expected DEFF | Relative difference (%)* | Observed DEFF* | Expected DEFF | Relative difference (%)* |
| Cluster | 240 | Unequal cluster proportions | 0.2 | 1.19 | | 1.20 | -1.05 | 1.15 | 1.16 | -0.81 |
| Cluster | 240 | Unequal cluster proportions | 0.8 | 1.78 | | 1.80 | -0.97 | 1.44 | 1.44 | -0.39 |
| Cluster | 144 | Equal cluster proportions | 0.2 | 1.38 | | 1.40 | -1.58 | 1.35 | 1.37 | -1.35 |
| Cluster | 144 | Equal cluster proportions | 0.8 | 2.58 | | 2.60 | -0.73 | 2.24 | 2.25 | -0.59 |
| Cluster | 560 | Unequal cluster proportions | 0.2 | 1.19 | | 1.20 | -0.47 | 1.16 | 1.16 | -0.41 |
| Cluster | 560 | Unequal cluster proportions | 0.8 | 1.79 | | 1.80 | -0.43 | 1.44 | 1.44 | -0.19 |
| Cluster | 336 | Equal cluster proportions | 0.2 | 1.39 | | 1.40 | -0.81 | 1.36 | 1.37 | -0.68 |
| Cluster | 336 | Equal cluster proportions | 0.8 | 2.59 | | 2.60 | -0.30 | 2.25 | 2.25 | -0.18 |
| Individual | 240 | Unequal cluster proportions | 0.2 | 0.99 | | 1.00 | -0.99 | 0.96 | 0.97 | -0.91 |
| Individual | 240 | Unequal cluster proportions | 0.8 | 0.99 | | 1.01 | -2.18 | 0.45 | 0.46 | -1.53 |
| Individual | 144 | Equal cluster proportions | 0.2 | 0.99 | | 1.00 | -1.35 | 0.93 | 0.94 | -1.52 |
| Individual | 144 | Equal cluster proportions | 0.8 | 1.00 | | 1.02 | -2.10 | 0.32 | 0.33 | -3.13 |
| Individual | 560 | Unequal cluster proportions | 0.2 | 1.00 | | 1.00 | -0.41 | 0.97 | 0.97 | -0.33 |
| Individual | 560 | Unequal cluster proportions | 0.8 | 1.00 | | 1.01 | -0.83 | 0.46 | 0.46 | -0.74 |
| Individual | 336 | Equal cluster proportions | 0.2 | 1.00 | | 1.00 | -0.58 | 0.94 | 0.94 | -0.67 |
| Individual | 336 | Equal cluster proportions | 0.8 | 1.01 | | 1.02 | -1.02 | 0.33 | 0.33 | -1.06 |

* Median value across 10000 simulated datasets.

Clusters of size 1-4 were distributed in proportions (0.70, 0.15, 0.10, 0.05) in scenarios with unequal cluster proportions (corresponding to $\boldsymbol{\gamma}$ = (0.47, 0.20, 0.20, 0.13)), and (0.25, 0.25, 0.25, 0.25) in scenarios with equal cluster proportions (corresponding to $\boldsymbol{\gamma}$ = (0.1, 0.2, 0.3, 0.4)).

GEE = generalised estimating equation, DEFF = design effect, ICC = intracluster correlation coefficient

.

Supplementary Table 15: Observed and expected power for a binary outcome with a log link, with a varying number of clusters of each size

|  | | | | GEE independence | | | GEE exchangeable | | |
| --- | --- | --- | --- | --- | --- | --- | --- | --- | --- |
| Randomisation method | Number of clusters | Distribution of cluster sizes | ICC | Observed power | Expected power | Absolute difference | Observed power | Expected power | Absolute difference |
| Cluster | 240 | Unequal cluster proportions | 0.2 | 43.91 | 44.67 | -0.76 | 45.23 | 45.80 | -0.57 |
| Cluster | 240 | Unequal cluster proportions | 0.8 | 30.98 | 31.97 | -0.99 | 37.29 | 38.45 | -1.16 |
| Cluster | 144 | Equal cluster proportions | 0.2 | 38.81 | 39.38 | -0.57 | 39.70 | 40.14 | -0.44 |
| Cluster | 144 | Equal cluster proportions | 0.8 | 23.43 | 23.66 | -0.23 | 26.45 | 26.58 | -0.13 |
| Cluster | 560 | Unequal cluster proportions | 0.2 | 79.66 | 79.64 | 0.02 | 81.09 | 80.85 | 0.24 |
| Cluster | 560 | Unequal cluster proportions | 0.8 | 61.90 | 62.44 | -0.54 | 71.60 | 72.07 | -0.47 |
| Cluster | 336 | Equal cluster proportions | 0.2 | 72.71 | 73.30 | -0.59 | 73.76 | 74.28 | -0.52 |
| Cluster | 336 | Equal cluster proportions | 0.8 | 45.97 | 47.40 | -1.43 | 51.84 | 53.03 | -1.19 |
| Individual | 240 | Unequal cluster proportions | 0.2 | 51.45 | 51.51 | -0.06 | 53.04 | 52.89 | 0.15 |
| Individual | 240 | Unequal cluster proportions | 0.8 | 51.54 | 51.22 | 0.32 | 89.97 | 83.75 | 6.22 |
| Individual | 144 | Equal cluster proportions | 0.2 | 50.75 | 51.41 | -0.66 | 53.33 | 54.01 | -0.68 |
| Individual | 144 | Equal cluster proportions | 0.8 | 51.36 | 50.85 | 0.51 | 99.21 | 93.37 | 5.84 |
| Individual | 560 | Unequal cluster proportions | 0.2 | 86.01 | 86.25 | -0.24 | 87.20 | 87.38 | -0.18 |
| Individual | 560 | Unequal cluster proportions | 0.8 | 85.97 | 86.01 | -0.04 | 99.96 | 99.44 | 0.52 |
| Individual | 336 | Equal cluster proportions | 0.2 | 85.52 | 86.17 | -0.65 | 87.87 | 88.25 | -0.38 |
| Individual | 336 | Equal cluster proportions | 0.8 | 85.39 | 85.69 | -0.30 | 100.00 | 99.96 | 0.04 |

Clusters of size 1-4 were distributed in proportions (0.70, 0.15, 0.10, 0.05) in scenarios with unequal cluster proportions (corresponding to $\boldsymbol{\gamma}$ = (0.47, 0.20, 0.20, 0.13)), and (0.25, 0.25, 0.25, 0.25) in scenarios with equal cluster proportions (corresponding to $\boldsymbol{\gamma}$ = (0.1, 0.2, 0.3, 0.4)).

GEE = generalised estimating equation, ICC = intracluster correlation coefficient.
